# Brain Morphometry Estimation: From Hours to Seconds Using Deep Learning

**DOI:** 10.3389/fneur.2020.00244

**Published:** 2020-04-08

**Authors:** Michael Rebsamen, Yannick Suter, Roland Wiest, Mauricio Reyes, Christian Rummel

**Affiliations:** ^1^Support Center for Advanced Neuroimaging (SCAN), University Institute of Diagnostic and Interventional Neuroradiology, University of Bern, Inselspital, Bern University Hospital, Bern, Switzerland; ^2^Graduate School for Cellular and Biomedical Sciences, University of Bern, Bern, Switzerland; ^3^Insel Data Science Center, Inselspital, Bern University Hospital, Bern, Switzerland; ^4^ARTORG Center for Biomedical Research, University of Bern, Bern, Switzerland

**Keywords:** human brain morphometry, MRI, deep learning, epilepsy, cortical thickness

## Abstract

**Motivation:** Brain morphometry from magnetic resonance imaging (MRI) is a promising neuroimaging biomarker for the non-invasive diagnosis and monitoring of neurodegenerative and neurological disorders. Current tools for brain morphometry often come with a high computational burden, making them hard to use in clinical routine, where time is often an issue. We propose a deep learning-based approach to predict the volumes of anatomically delineated subcortical regions of interest (ROI), and mean thicknesses and curvatures of cortical parcellations directly from T1-weighted MRI. Advantages are the timely availability of results while maintaining a clinically relevant accuracy.

**Materials and Methods:** An anonymized dataset of 574 subjects (443 healthy controls and 131 patients with epilepsy) was used for the supervised training of a convolutional neural network (CNN). A *silver-standard* ground truth was generated with FreeSurfer 6.0.

**Results:** The CNN predicts a total of 165 morphometric measures directly from raw MR images. Analysis of the results using intraclass correlation coefficients showed, in general, good correlation with FreeSurfer generated ground truth data, with some of the regions nearly reaching human inter-rater performance (ICC > 0.75). Cortical thicknesses predicted by the CNN showed cross-sectional annual age-related gray matter atrophy rates both globally (thickness change of −0.004 mm/year) and regionally in agreement with the literature. A statistical test to dichotomize patients with epilepsy from healthy controls revealed similar effect sizes for structures affecting all subtypes as reported in a large-scale epilepsy study.

**Conclusions:** We demonstrate the general feasibility of using deep learning to estimate human brain morphometry directly from T1-weighted MRI within seconds. A comparison of the results to other publications shows accuracies of comparable magnitudes for the subcortical volumes and cortical thicknesses.

## 1. Introduction

Magnetic resonance imaging (MRI) is the method of choice for non-invasive assessments of brain structure. Clinicians use MRI for diagnosis, disease monitoring, and therapy control in a wide range of neurological and neurogenerative disorders like e.g., epilepsy, multiple sclerosis, Alzheimer's, Parkinson's, or Huntington's disease, which are often associated with structural changes of the brain (1). Structural MRI including high-resolution T1-weighted (T1w) imaging is part of today's protocol recommendations for many of these disorders (2–4). Beyond visual assessment by trained experts, quantitative brain morphometry is gaining increasingly more attention for medical applications. Precise and automatic reconstruction of structures from MRI is still a topic of active research. Commonly used methods are voxel-based morphometry (VBM) (5) and surface-based analysis (SBA) (6).

A variety of morphometric parameters have been proposed. Three of the most frequently used parameters are the volumes of anatomically delineated regions of interest (ROIs), and the thickness and the curvature of the cortical band. Volumes are either reported in physical units as *mm*^3^ or *cm*^3^, or as fractions of the intracranial volume. Total gray matter (GM) volume is known to decrease with aging (7), which can regionally or globally be accelerated by neurodegenerative diseases (8, 9). Atrophy of brain tissue is generally accompanied by enlarged ventricles and increased volume of cortical (sulcal) cerebrospinal fluid (CSF) that sustains the brain within the skull (10).

Cortical thickness is the distance in *mm* between the white matter (WM) surface (i.e., the interface between GM and WM) and pial surface (i.e., the interface between GM and CSF). The overall mean thickness of the healthy human cerebral cortex is about 2.5 mm, with regional variations between 1 and 4.5 mm (11). A multitude of geometrical definitions for the curvature of a surface exist (12). The mean curvature, as an extrinsic measure for the folding of the cortex (13), roughly corresponds to the inverse of the radius of a sphere fitted to the surface and is measured in *mm*^−1^. Both, thickness and curvature of the cortex, can be reported per vertex on a reconstructed surface mesh or as ROI-wide averages (parcellations). In the interest of readability, we here use the terms thickness and curvature to refer to their parcellation-wise averages.

Large-scale studies of brain morphometry are only possible if morphometric parameters are available for a large number of MR images, with high accuracy and in a reproducible manner. However, manual segmentation and measurements are extremely labor intensive, prone to errors, and good intra- and inter-rater reproducibility depends on task-specific training (14). Software for automatic or semi-automatic extraction of brain morphometry from MRI is available and includes tools such as FreeSurfer (15), FSL (16), ANTs (17), NeuroQuant (18), and IBASPM (19). Among these morphometry tools, FreeSurfer is the most comprehensive, as it provides many metrics, including direct measures of volumes and cortical thickness and curvature.

In a large-scale, multi-center study by the ENIGMA consortium (20), significant structural changes in the brains of epilepsy patients have been identified recently (21). When compared to a cohort of healthy controls, altered subcortical volumes and reduced cortical thickness in distinct regions were observed. The feasibility of applying morphometry tools to individual patients and to support clinical diagnostics has been shown (22) by comparing personalized morphometric analysis to a normative database adjusted for confounding factors like age and sex.

Brain morphometry is expected to become an essential quantitative neuroimaging biomarker (23). Although currently mainly used in the academic realm, it has great potential to complement today's predominantly qualitative visual assessments of MRI by neuroradiologists. If morphometry is to be used for diagnostics of individual patients in daily clinical practice, the timely availability becomes crucial. Today's state of the art tools for the automatic determination of brain morphometry often come with a high computational burden (~10 h with FreeSurfer), heavily hampering their use in clinical routine, where time is often an issue.

The adoption of deep learning in medical image analysis has increased rapidly over the past years. In current research projects, it has even become the method of first choice for many tasks. In a review of recent studies that use deep learning in medical image analysis (24), MRI was the most frequently used imaging modality, and the brain the most prominent organ. While the vast majority of tasks concern image segmentation and classification, applications of deep learning for regression (prediction) of morphometry in medical image applications are still rare, especially for brain MRI. Technically, convolutional neural networks (CNNs) (25) are the most prevalent architectures for image analysis. Despite the 3D nature of MRI, many methods still use 2D convolutions. Input is often fed patch- or slice-wise into the networks, partially motivated by limited computational resources and the lack of large-scale training data (26). The increase of power and memory of modern GPUs has the potential to change this, though.

A regression problem leveraging the full 3D MRI volume using a CNN was proposed by Cole et al. (27), where they successfully predicted brain age directly from raw MRI with a mean absolute error of <5 years, i.e., much smaller than the age range of available datasets. Deep learning has been used to directly estimate the wall thickness of the ventricular myocardium from a sequence of cardiac images (28). The authors made use of both, the spatial and temporal information, by combining a CNN and a recurrent neural network (RNN). Directly classifying neurological diseases is another popular challenge that is being tackled by deep learning, mainly for Alzheimer's disease (29–31) where a large public dataset is available from the Alzheimer's Disease Neuroimaging Initiative (ADNI) (32).

Regarding brain anatomy, promising results in the application of deep learning-based models were observed for the segmentation of tissue classes and subcortical structures (33–38). The challenge of having access to enough labeled data for training is addressed by semi-supervised (39) and unsupervised (40) approaches or data augmentation strategies simulating diverse pulse sequences (41). While these segmentation-based methods enable calculation of volumes in a timely fashion, none of them provide thickness or curvature measures of the cortex. Graph convolutional networks (GCN) have been used (42, 43) to parcellate the surface of the cerebral cortex. For calculating the cortical thickness, alternative methods like Laplace equations (44) or registration-based solutions (45) have been proposed. Recently, *FastSurfer* was proposed as an optimized FreeSurfer pipeline, reducing the runtime to about 1.7 h, which is primarily achieved by a deep learning-based whole brain segmentation and a faster surface reconstruction and spherical mapping using marching cube and Laplace eigenfunctions (46).

A classical machine learning approach for brain morphometry estimation from MRI was proposed by Suter et al. (47), using a Random Forest to directly estimate cortical thickness and curvature, both on a per voxel and parcellation level. As a limitation, their approach still depended on the first part of the FreeSurfer pipeline to pre-process the data before feeding it into the model. Including feature extraction, this required about 30 min to predict the morphometric parameters of a single subject.

**Figure 1:**
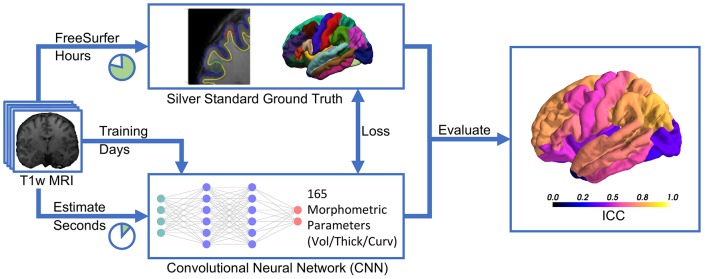
Deep learning-based estimation of brain morphometry directly from T1-weighted MRI, making results available within seconds.

Recent advances in deep learning for image analysis motivated us to propose a deep learning-based approach for direct estimation (regression) of brain morphometry from MRI. We hypothesized that a neural network can directly predict the volumes of anatomically delineated subcortical ROI, and mean thicknesses and curvatures of cortical parcellations. Advantages would be the availability of results within seconds while maintaining a clinically relevant accuracy (see Figure 1). While deep learning-based methods are increasingly used for fast brain anatomy segmentation, this is—to the best of our knowledge—the first application to directly regress morphometric measures of the cortex.

This paper is structured as follows: after a description of the data, their pre-processing, the network architecture and the evaluation metrics in the methods section, we first analyze the predictions in terms of correlation coefficients against a *silver-standard* ground truth. The relevance of our predictions beyond correlation is assessed via a group comparison of epilepsy patients with healthy controls approximating the worldwide recognized ENIGMA study, and an analysis of cross-sectional age-related cortical GM atrophy rates. Finally, we contrast the results to the literature and analyze the reliability by means of rescan tests.

## 2. Materials and Methods

### 2.1. Data

The data for this project were used in previous studies (22, 48) by the Bern University Hospital (Inselspital). The dataset consists of anonymized, high-resolution isotropic T1-weighted MR images, acquired at the Inselspital on two 3T MR scanners (Magnetom Trio and Verio, Siemens, Erlangen, Germany). Images were acquired in sagittal direction and MRI protocols were either MDEFT (49), standard 3D MP-RAGE (50), MP-RAGE according to the recommendations of the Alzheimer's Disease Neuroimaging Initiative (51) or MP-RAGE optimized for gray-white contrast (52). Detailed sequence parameters can be found in the Supplementary Material of Rummel et al. (48).

Only age, sex, scanner, and sequence are known from the anonymized data. Both healthy controls (*n* = 443) and patients with epilepsy (*n* = 131) are included in the dataset. The age range across all subjects is from 6 to 84 years. The demographic distribution of the subsets is shown in Table 1.

**Table 1:** Demographic information of the subjects and its distribution to the three datasets.

	**Healthy controls**			**Epilepsy**		
	***n***	**Mean age (±SD)**	**% Male**	***n***	**Mean age (±SD)**	**% Male**
Train	336	34.5 ± 20.2	44.3	102	35.0 ± 14.4	48.0
Validate	35	30.9 ± 21.3	37.1	11	30.3 ± 13.0	45.5
Test	72	31.5 ± 12.9	38.9	18	33.9 ± 16.2	44.4
Overall	443	33.7 ± 19.3	42.9	131	34.4 ± 14.5	47.3

*Age in years*.

The dataset contains a certain number of re-scans, i.e., for some healthy controls more than one MRI is available (48) in intervals not longer than 2 years. All MR images of these subjects were intentionally assigned to the test set to enable robustness tests. Since all these subjects are within the age range of 21–41 years, this results in a lower standard deviation of the age in the test set. The remaining subjects were randomly distributed among the three sets.

#### 2.1.1. FreeSurfer

Due to the lack of a gold-standard ground truth for brain morphometry, we used FreeSurfer to generate a *silver-standard* ground truth in this project. FreeSurfer (FS) (15) is a freely available software package for the analysis of neuroimaging data.

To obtain the volumes of anatomical brain segmentations, FreeSurfer performs a whole brain segmentation of subcortical and ventricular structures, assigning a label to each voxel (53). The SBA is derived from a geometric model of the cortical surface (6). SBA measures are available per vertex or averaged for ROI for which the cortex is parcellated and mapped to a brain atlas.

An automatic reconstruction of a topologically correct surface for the highly folded brain cortex is an extraordinarily difficult task. A breakthrough in the development of FreeSurfer was to use a combination of both the pial and the gray/white matter boundaries along with volume intensities to achieve an anatomically accurate surface representation. This iterative process of topological corrections is computationally expensive and the most time-consuming part in the whole FreeSurfer pipeline. It is owed to this high-resolution surface mesh that allows measurements of cortical thickness with submillimeter accuracy, which is necessary to characterize subtle cortical atrophy in diseases (11).

The accuracy and reliability of FreeSurfer have been investigated multiple times, e.g., by comparing the results with manual segmentation by experts (54–56), by performing scan-rescan studies (57, 58), or through comparison with other tools (59). FreeSurfer's output may be influenced by the image acquisition setup like scanner manufacturer, field strength, and protocols (60), but also the version of FreeSurfer, and even the underlying hardware and operating system, are known to influence the results when applied to the same MR image (61).

#### 2.1.2. Ground Truth Generation

A *silver-standard* ground truth for the cortical and subcortical morphometrics was generated with FreeSurfer 6.0 (recon-all) running on CentOS Linux, release 6.9. Average processing time was 11.3±3.3 h per MR image. Subcortical volumes in *mm*^3^ for 29 ROI were extracted from the segmentation statistics (aseg.stats) (53). The volume of the corpus callosum was calculated by summing up its five sub-regions (anterior, mid-anterior, central, mid-posterior, and posterior). Cortical thicknesses in *mm* and curvatures in *mm*^−1^ were extracted from the surface statistics (lh.aparc.stats, rh.aparc.stats) as their parcellation-wise averages defined by the Desikan-Killiany (DK) atlas (62), resulting in 34 ROIs per hemisphere.

The reliability of the FreeSurfer output depends on previous steps in the processing pipeline, mainly the tissue segmentation and surface reconstruction. Errors therein may lead to significant deviations. As a simple automatic quality check to detect likely erroneous large outlier, the output from FreeSurfer was fed into an existing pipeline for automated morphometric analysis developed by Rummel et al. (48). The pipeline reported an unusually high number of significantly abnormal regions for 17 subjects which were removed from the dataset. One additional subject was removed after visual inspection due to a severely distorted white matter mask from FreeSurfer.

#### 2.1.3. Data Pre-processing

Pre-processing of the raw MR images for deep learning included the following steps: The brain mask from the FreeSurfer output was used for skull-stripping the original T1w image. This anonymized image was then re-sampled and cropped to 256 × 256 × 256 voxels with a size of 1 *mm*^3^ (mri_convert) in order to have a common input size across all subjects. The voxel intensities of each image were re-scaled into the range 0–4,095 to account for intensity variations between different images. Last, the center of mass from all foreground voxels was moved to the center of the image to facilitate data augmentation described below.

### 2.2. Convolutional Neural Network Architecture

The scaffold for the development of the custom network architecture for brain morphometry was to some extent inspired by AlexNet (63), the winner of the 2012 ImageNet Large Scale Visual Recognition Challenge (ILSVRC) (64). Motivated by the volumetric nature of MR images, we use 3D convolutions on the full input volume instead of 2D with three input channels in AlexNet. Further modifications include a reduction by two convolution layers, adjustments in the fully connected layers to account for different sizes, and a regression output. This results in a network architecture with a total of six layers, as depicted in Table 2. Accordingly, the receptive field after the last pooling layer is 209 in all three dimensions.

**Table 2:** Architecture of the CNN for brain morphometry.

**Layer**	**Kernel**	**Stride**	**Filters**	**Output size**	**Activation function**
Input	–	–	–	256 × 256 × 256	–
Conv3D	11 × 11 × 11	3	144	86 × 86 × 86 × 144	ReLU
MaxPool	3 × 3 × 3	2	–	42 × 42 × 42 × 144	–
Conv3D	5 × 5 × 5	2	192	21 × 21 × 21 × 192	ReLU
MaxPool	3 × 3 × 3	2	–	10 × 10 × 10 × 192	–
Conv3D	5 × 5 × 5	1	192	10 × 10 × 10 × 192	ReLU
MaxPool	3 × 3 × 3	2	–	4 × 4 × 4 × 192	–
FC	–	–	–	374	ReLU
FC	–	–	–	192	–
FC	–	–	–	165	–

Dropout (0.4) is applied after the last MaxPool layer and after first FC layer.

*A bias is added to the first convolutional and all fully connected layers. Conv3D, 3D convolution; FC, fully connected layer; ReLU, rectified linear unit*.

The total number of trainable parameters in the network is 9 467 877, about half of them being in the convolutional layers. The weights of the convolutional kernels are initialized randomly according to the Xavier Uniform Initializer (65). All variables of the fully connected layers and the bias are zero-initialized.

The mean squared error (MSE) objective function is minimized using Adam (66) as gradient-based optimizer with an empirically determined initial learning rate of 10^−5^. With a batch size of 6, the training of one epoch consists of 73 steps and requires about 3 min to complete.

The model was implemented in Python using Tensorflow 1.8 (67). Training was performed on a NVIDIA Titan Xp GPU with 12 GB memory. During training, the accuracy was periodically evaluated on the validation set. The model of the best epoch, measured in terms of mean *R*^2^ across all regression morphometrics, was kept for early stopping.

We found the following data augmentation strategy allows the model to be trained for more epochs before the onset of overfitting: The skull-stripped input image was randomly translated by up to ±15 voxel in a randomly selected dimension, followed by three consecutive 90° rotations around a random principal axis. Besides artificially increasing the amount of training data, this has the positive side effect of enabling the model to process images in an arbitrary orientation. These transformations are computationally inexpensive and can be performed for the (pre-fetched) next batch on the CPU while calculations of the current batch are running on the GPU.

### 2.3. Evaluation

Several metrics exist to evaluate the correlation and reliability of a regression model. For direct comparison with others, we report the results for all three metrics mentioned below in the Supplementary Material.

The *coefficient of determination*, denoted *R*^2^, is an indicator for the goodness of fit of a linear regression model:

(1) $$\begin{array}{l} R^{2}=1-\frac{\sum_{i\;=1}^{N}{\left(y_{i}-g_{i}\right)}^{2}}{\sum_{i\;=1}^{N}{\left(g_{i}-\bar{g}\right)}^{2}} \end{array}$$

where *y*_*i*_ is the prediction for the *ith* sample, *g*_*i*_ the *silver-standard* ground truth and ḡ the sample mean for *N* samples.

The *Pearson correlation coefficient*, denoted *r* when applied to a sample, measures the linear correlation of two variables:

(2) $$\begin{array}{l} r=\frac{cov\left(y,g\right)}{\sigma_{y}\sigma_{g}} \end{array}$$

where σ is the standard deviation of the prediction and *silver-standard* ground truth, respectively. Pearson's *r* is less susceptible to large outlier than *R*^2^.

A fixed bias remains unrecognized by Pearson's *r* (e.g., reports a perfect correlation of 1 for *y* = 2*g* or *y* = *g*+1). Therefore we employed the intraclass correlation coefficient (ICC) along with a 95% confidence interval as primary quantitative metric to assess the reliability of the predictions (68). Reflecting both degree of correlation and agreement between measurements, ICC is widely used in medicine to measure intra- and inter-rater performance as well as for the evaluation of test-retest experiments. In its original form, ICC is defined as the ratio of true variance ($\sigma_{g}^{2}$) to true variance plus error variance ($\sigma_{\epsilon }^{2}$):

(3) $$\begin{array}{l} ICC=\frac{\sigma_{g}^{2}}{\sigma_{g}^{2}+\sigma_{\epsilon }^{2}} \end{array}$$

Modern definitions use sample mean squares from analysis of variance (ANOVA). Various assumptions lead to slightly different forms of ICC (69). By following the guideline from Koo and Li (70), the appropriate form for our task is *two-way mixed effects, absolute agreement, single rater/measurement* also known as:

(4) $$\begin{array}{l} ICC\left(2,1\right)=\frac{MS_{R}-MS_{E}}{MS_{R}+\frac{1}{N}\left(MS_{C}-MS_{E}\right)} \end{array}$$

where *MS*_*R*_ = mean square for rows, *MS*_*E*_ = mean square for error and *MS*_*C*_ = mean square for columns from ANOVA. However, some papers lack a clear definition of which ICC was used exactly, making one-to-one comparisons more difficult.

Rules of thumb for the interpretation of ICC in the context of clinical significance are given by Cicchetti et al. (71):

- Less than 0.40 : poor
- Between 0.40 and 0.59 : fair
- Between 0.60 and 0.74 : good
- Between 0.75 and 1.00 : excellent

All three evaluation metrics yield values below 0 for negative correlation or poor agreement, 0 for no correlation, e.g., a model just predicting the average expected outcome, and gradually become 1 for perfect correlation. The metrics were calculated in *R* (72) with the additional package *irr* (73) for ICC.

Besides simple correlation plots and the quantitative metrics described above, we further analyzed the predictions qualitatively using Bland-Altman plots (74) by plotting the differences against the means of the two methods (75). *Studying the difference rather than the agreement* is a recommended (76) analysis technique if a new method is to be compared to an existing, well-established method and the underlying true values are actually unknown (as in our case with brain morphometry and FreeSurfer as the established method).

### 2.4. Clinical Significance - Patients With Epilepsy

A widely used application of brain morphometry in clinical research is the statistical comparison of two different groups in a population. To explore the efficacy of our deep learning-based approach beyond purely technical metrics, we assessed to which degree we could replicate the findings of such a research study with the morphometrics estimated by the CNN.

In a large-scale study (21), including more than 2,000 patient cases, the ENIGMA consortium assessed structural brain abnormalities in patients with epilepsy. Among the findings were increased volumes of the lateral ventricle bilaterally, decreased volumes of the thalamus and globus pallidus from the right hemisphere, and a reduced mean thickness of the precentral gyrus and paracentral lobule bilaterally in patients with epilepsy when compared to a group of healthy controls. Only the aforementioned eight metrics showed statistically significant deviations in all four epilepsy subgroups examined by the study. Our dataset contains patients with epilepsy from all four subgroups, but the sample size does not allow for stratification into small subgroups. The baseline from ENIGMA is, therefore, the “All epilepsies” phenotype. Effect sizes adjusted for age and sex to compare healthy controls vs. patients with epilepsy were calculated using Cohen's *d*, implemented in the *R* package *effsize* (77). Statistical significance was determined with a one-sided *t*-test (*p* < 0.05).

To increase the sample size for the test, we created three additional train/validate/test splits of the dataset, each with a unique set of subjects in the validation and test set (non-exhaustive cross validation). Models were trained (as described in section 2.2) independently of each other using these sets. The combined predictions from the four resulting test sets yield a sample of 274 healthy controls and 86 patients with epilepsy. Although our population is much smaller than in ENIGMA (1,727 healthy controls and 2,149 patients with epilepsy), a comparison using the effect size is valid as this statistical test is not confounded by the sample size.

### 2.5. Age-Related Cortical Gray Matter Atrophy

The overall cortical thickness is known to decrease with normal aging (7). This age-related atrophy varies regionally (78). We assessed whether this trend is recognizable in the predictions from the CNN on the whole cohort of controls and patients. The age effect on the predicted thicknesses was analyzed in *R* by fitting a general linear model, both globally for the whole brain (all parcellations averaged) and regionally for each parcellation. In order to account for multiple tests, the significance level was Bonferroni corrected with a factor of 68 (number of parcellations in both hemispheres).

The results were compared to the study of Lemaitre et al. (78) in which a similar cohort (216 participants with a mean age of 39.8±16.5 years) was analyzed for age-related regional morphometric changes.

### 2.6. Reliability by Rescan Tests

Due to the lack of a gold-standard ground truth, we should not solely rely on the accuracy to judge on the performance of a method. Reliability is another important quality feature. Repeated measurements of the same subject should ideally yield similar values, or in our case, different MRI from the same subject should report similar results. For nine subjects, between three and six scans are available in the dataset. Since these rescans were acquired within a time frame of maximum 2 years, we assume only minor structural changes in the brain occurred during this time. Hence we assume an unchanged ground truth and assessed the reliability by means of evaluating the standard deviation of the morphometrics predicted by the CNN.

## 3. Results

**Figure 2:**
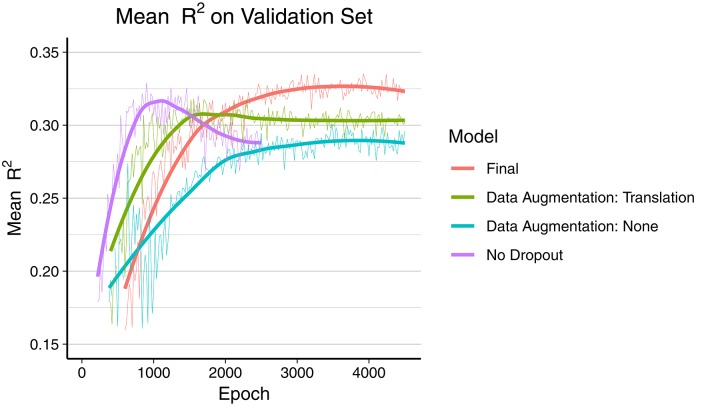
Comparison of overfitting behavior of four different models during training. Mean *R*^2^ of all 165 regression metrics is calculated every 20th epoch. The thick line is the smoothed curve of the individual data points (thin line). Final model: Dropout and data augmentation with rotations and translations. Green and blue model include dropout.

The final model was trained during 7 days over 4,500 epochs, with the best mean *R*^2^ score on the validation set reached at epoch 3,920 (early stopping). As depicted in Figure 2, the final model using dropout and data augmentation required more training steps to converge. Both translations and rotations contributed to reduce overfitting and to achieve a higher *R*^2^. Dropout roughly tripled the number of epochs required to converge. About 15% of the performance gain, in terms of mean *R*^2^, was attributed to data augmentation. The corresponding metrics on the training data can be found in Figure S1 (Supplementary Material), showing earlier convergence without data augmentation.

All results below are from the evaluation on the test set consisting of 90 subjects, as described in section 2.1. The total runtime required for predicting all 165 morphometrics for these 90 subjects was 698 s, which is less than 8 s for a single MR image. This included all necessary pre-processing steps of which re-sampling to unit volume and isovoxel took most of the time, whereas passing the data through the CNN on the GPU was below 1 s.

**Figure 3:**
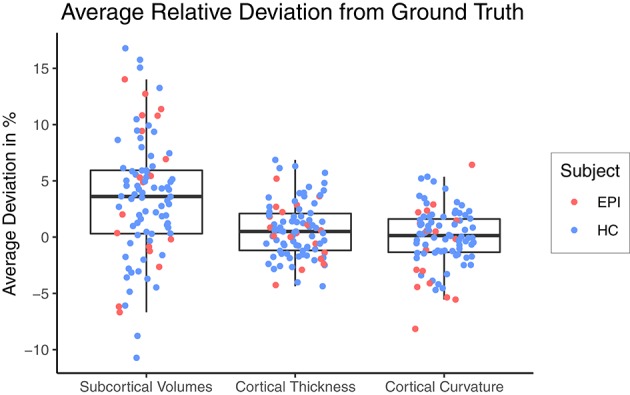
Relative deviation from *silver-standard* ground truth (CNN – FS), averaged across all regions of interest per category for each subject. HC, healthy controls; EPI, patients with epilepsy.

Figure 3 shows a Box-and-Whiskers plot of the averaged relative error for each category. The mean relative deviations from *silver-standard* ground truth were below 5% for all three categories (volume = 3.43±5.41%, thickness = 0.63±2.44%, curvature = 0.02±2.58%). The subsequent sections report and analyze the accuracy of the individual predictions for each of the three categories.

### 3.1. Subcortical Volume

**Figure 4:**
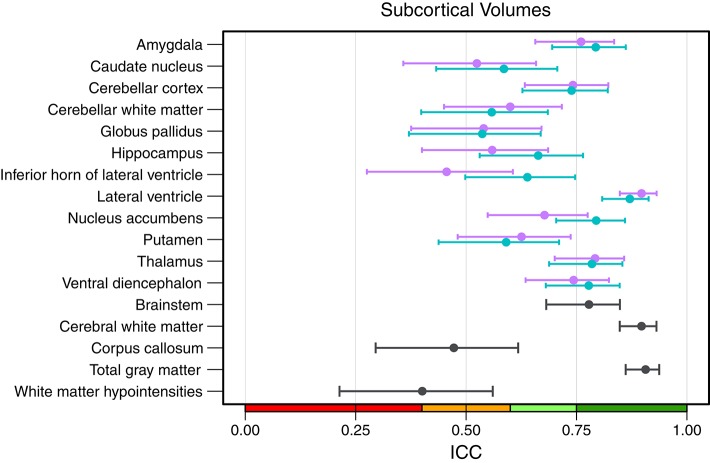
Intraclass correlation coefficients with 95% confidence intervals for all subcortical volumes. Purple, right hemisphere; green, left hemisphere. Color scale indicate poor (red), fair (orange), good (light-green), excellent (green) ICC.

An overview of all intraclass correlation coefficients along with 95% confidence intervals is shown in Figure 4 and detailed numbers are reported in Table S1 (Supplementary Material). Intraclass correlation coefficients were excellent (ICC above 0.75) for 11 out of 29 predicted volumes, good (ICC 0.60–0.74) for 7, and fair (ICC 0.40–0.59) for the remaining 11 volumes. The highest scores were reached for the volumes of total gray matter (ICC = 0.91), cerebral white matter (0.90), and left (0.87) and right lateral ventricle (0.90). Also excellent ICCs were reached for amygdala (left = 0.79, right = 0.76), thalamus (left/right = 0.79), left nucleus accumbens (0.79), brainstem (0.78), and left ventral diencephalon (0.78). Scores on the lower end were reported for the volumes of white matter hypointensities (0.40), right inferior horn of lateral ventricle (0.46) and corpus callosum (0.47). The mean ICC over all volumes was 0.68 (left hemisphere = 0.69, right hemisphere = 0.66). The ICCs were not significantly related to the size of the structures (*r* = 0.247, *p* = 0.215).

**Figure 5:**
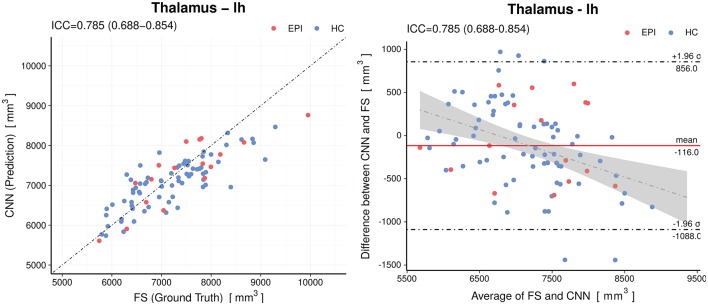
Accuracy of the predictions for the volumes of the left thalamus. Left: Correlation plot, Right: Bland–Altman plot. The CNN model shows a small (−116*mm*^3^) bias and a slight tendency to overestimate smaller volumes and underestimate the larger. The gray shaded area represents the 95% confidence interval of the regression line. Horizontal dashed lines delineate the 95% confidence intervals indicating the likelihood of individual measures to be within ±1.96 standard deviations. HC, healthy controls; EPI, patients with epilepsy.

When analyzing individual estimations using Bland-Altman plots, we observe a tendency of the CNN to have overestimated smaller volumes and underestimated the larger (see Figure 5 for an example of the left thalamus). The red horizontal line representing the mean difference between prediction and *silver-standard* ground truth was close to zero (the relative mean difference was below 3.2% for all structures except for the white matter hypointensities and inferior horn of lateral ventricles). This suggests only a small bias is present. The regression lines in the correlation plots were not as steep as 45° (perfect correlation) for most of the volumes, which is an indication the CNN was not able to fully capture the variance of the *silver-standard* ground truth. Correlation and Bland-Altman plots for all subcortical volumes are listed in the Supplementary Material.

### 3.2. Cortical Thickness and Curvature

**Figure 6:**
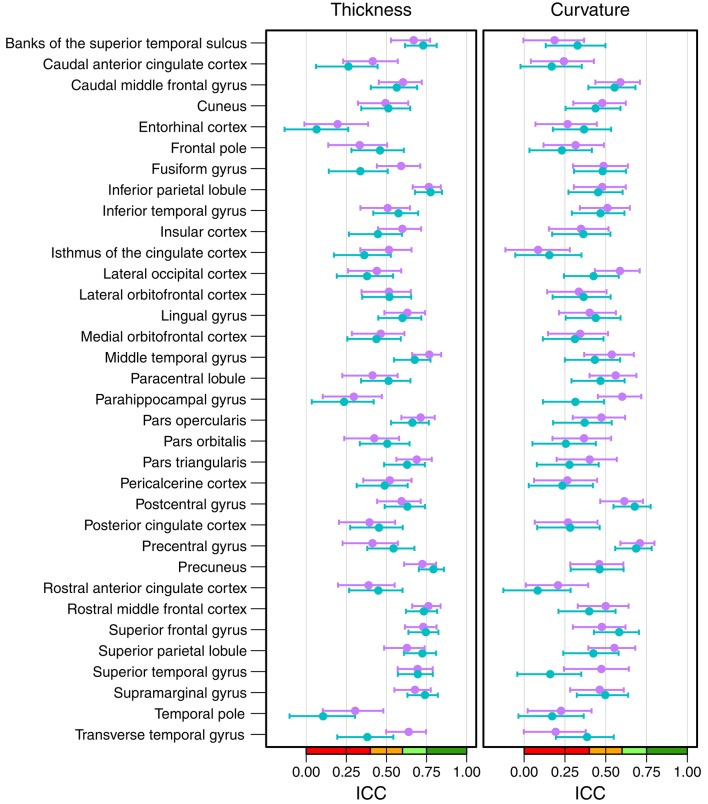
Intraclass correlation coefficients with 95% confidence intervals for cortical thickness (left) and cortical curvature (right). Purple, right hemisphere; green, left hemisphere. Color scale indicate poor (red), fair (orange), good (light-green), excellent (green) ICC.

Figure 6 shows the intraclass correlation coefficients of all cortical parcellations (detailed numbers are reported in Table S2). For the cortical thickness, the mean ICC of all 68 parcellations was 0.53 (left hemisphere = 0.52, right hemisphere = 0.54). An excellent ICC was reached for 5 parcellations, namely the thickness of left precuneus (0.79), left (0.78) and right inferior parietal lobule (0.76), right middle temporal gyrus (0.77), and right rostral middle frontal cortex (0.76). Good ICCs included 21 parcellations, fair 28, and poor 14. The lowest scores for the thickness were found for the left (0.06) and right entorhinal cortex (0.20) and left temporal pole (0.10).

The mean ICC of the cortical curvatures was 0.39 (left hemisphere = 0.38, right hemisphere = 0.41). No parcellations reached an excellent ICC for the cortical curvature, the 5 parcellations with a good ICC were: left (0.69) and right precentral gyrus (0.71), left (0.68) and right postcentral gyrus (0.61), and right parahippocampal gyrus (0.60). Fair ICCs were reached for 30 and poor for 33 parcellations. The lowest scores were found in the area of the cingulate cortex: left rostral anterior cingulate cortex (0.08), and left (0.16) and right isthmus of the cingulate cortex (0.08).

**Figure 7:**
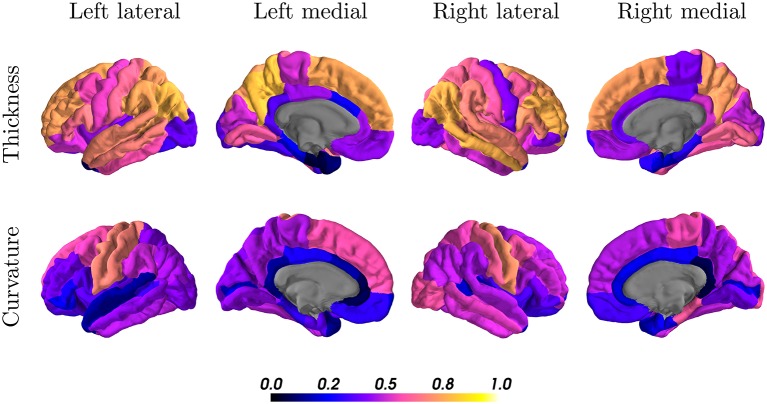
Intraclass correlation coefficients of all cortical regions for thickness (first row) and curvature (second row) superimposed on a standard brain. Color scales indicate poor (black, blue) to excellent (orange, yellow) ICC.

When looking at the anatomical location, we observed the best results in the parietal and frontal lobes, both for thickness and curvature (see Figure 7). For the cortical thickness, the mean ICC per lobes were: parietal (left = 0.73, right = 0.68), frontal (left = 0.57, right = 0.55), occipital (left = 0.50, right = 0.52), and temporal (left = 0.42, right = 0.52). For the cortical curvature, the results were in the same order with slightly lower scores, namely: parietal (left = 0.50, right = 0.51), frontal (left = 0.41, right = 0.46), occipital (left = 0.38, right = 0.43), and temporal (left = 0.35, right = 0.39).

### 3.3. Patients With Epilepsy

The predictions from the CNN were used to perform a population study equivalent to ENIGMA (21), dichotomizing epilepsy from healthy controls. Effect size differences between epilepsy and healthy control groups are shown in Table 3. The first column replicates the numbers from the ENIGMA epilepsy study. Cohen's *d* for the CNN and FreeSurfer were calculated on the combined test dataset of 274 subjects.

**Table 3:** Effect size differences between patients with epilepsy and healthy controls.

**Structure**	**ENIGMA (21)**	**CNN**		**FreeSurfer**
	**Cohen's *d***	**Cohen's *d***	***P*-value**	**Cohen's *d***
Lateral ventricle (lh)	0.288	**0.261**	2.66 × 10^−2^	0.145
Lateral ventricle (rh)	0.268	**0.282**	1.86 × 10^−2^	0.245
Thalamus (rh)	−0.368	0.136	1.79 × 10^−1^	0.051
Globus pallidus (rh)	−0.316	0.250	3.70 × 10^−2^	0.055
Paracentral lobule (lh)	−0.311	−**0.382**	6.37 × 10^−4^	−0.421
Paracentral lobule (rh)	−0.315	−**0.279**	1.09 × 10^−2^	−0.270
Precentral gyrus (lh)	−0.384	−**0.303**	1.26 × 10^−2^	−0.363
Precentral gyrus (rh)	−0.399	−**0.341**	4.31 × 10^−3^	−0.121

*lh, left hemisphere; rh, right hemisphere; bold numbers, effect size of CNN in agreement with ENIGMA. P−value: statistical significance of a one−sided t−test comparing the two groups*.

In agreement with the findings from ENIGMA, the predictions from the CNN showed statistically significant (*p* < 0.05) positive effect sizes for the volume of the lateral ventricles and negative effect sizes for the mean thickness of the paracentral lobules and precentral gyri bilaterally. Contrary to ENIGMA, the result showed an increased volume of the right globus pallidus for patients with epilepsy. No statistically significant effect size was found for the volume of the right thalamus. For the two deviating structures, both the predictions from the CNN and FreeSurfer fail to replicate the findings from ENIGMA.

### 3.4. Age-Related Cortical Gray Matter Atrophy

**Figure 8:**
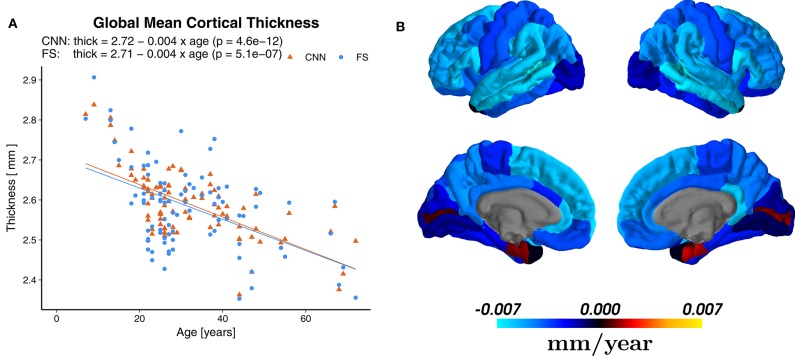
**(A)** Effect of age on the global mean cortical thickness (all parcellations averaged), both for the predictions of the CNN and FreeSurfer (FS). **(B)** Regional age-related cortical thinning. Regression maps based on the predictions from CNN superimposed on a standard brain. Color scale indicates change of thickness in mm/year (blue for reductions and red for increases), which can be compared to Figure 2b of Lemaitre et al. (78).

Linear regression revealed a statistically significant cross-sectional age-related reduction in global mean cortical thickness (*r* = −0.65, *p* = 4.6 × 10^−12^) with an overall effect of 0.004±0.002 mm per year (average ± SD), see Figure 8A. The regional distribution of the age effects can be seen in Figure 8B. Predominant reductions were observed in the frontal (average −0.0049±0.0020 mm/year) and parietal (−0.0047±0.0008 mm/year) lobes and less in the temporal (−0.0037±0.0029 mm/year) lobe. In the occipital lobe, the age-dependent thickness change was considerably smaller (−0.0009±0.0012 mm/year).

**Figure 9:**
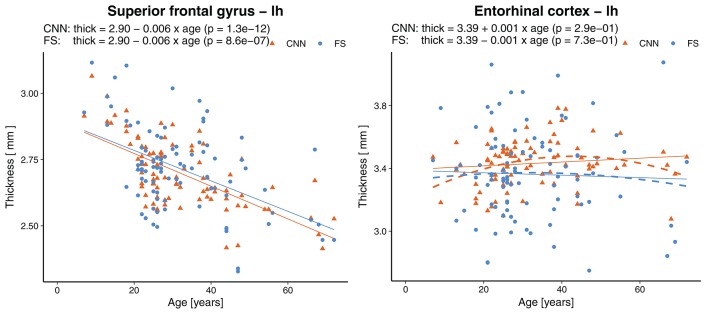
Effect of age on the thickness of the superior frontal gyrus (left) and entorhinal cortex (right). Dashed lines represent second order polynomial age trends. FS, FreeSurfer.

Statistically significant (*p* < 0.0007, Bonferroni corrected) age-related reductions were seen not only globally, but also on most (55/68) of the individual parcellations. Figure 9 shows an example of the superior frontal gyrus from the left hemisphere. A list of all thickness vs. age plots can be found in the Supplementary Material. A decreasing thickness was observed for all parcellations except the pericalcerine and entorhinal cortex. The linear age trend for the entorhinal cortex was slightly positive. When fitting a quadratic model (dashed line in Figure 9 right), we observed an increased thickness with age until a peak around 45 years followed by a decrease again. This observation is consistent with the finding of Hasan et al. (79). They have identified the same pattern for the entorhinal cortex with a peak thickness at about 44 years in a large cohort of 1,660 participants.

### 3.5. Comparison With Others

The accuracy and reliability of morphometric measures from MRI have been subject to various studies, both for automatic methods and manual segmentation. A comparison of our results to metrics reported by others is shown in Table 4. Selected structures include some of the most frequently investigated subcortical volumes and the cortical thickness of all parcellations in the parietal lobe.

**Table 4:** Comparison of results to metrics reported by others.

	**CNN vs.FS**		**FIRST vs.** **Manual**	**IBASPM vs.** **Manual**	**NeuroQuant vs.** **FS**	**Inter-observer**	**FS corr.vs.FS**	**FS Test-Retest**	**FS Test-Retest**	**FIRST Test-Retest**
			**(55)**	**(56)**	**(59)**	(54)		**(57)**	(58)	
**Structure**	***r***	**ICC**	***r***	***r***	***r***	**ICC**	**ICC**	**ICC**	**ICC**	**ICC**
Amygdala (lh)	0.83	0.79	0.24		0.86	0.72	-0.10	0.68	**0.87**	0.75
Amygdala (rh)	0.81	0.76	0.24		0.85	**0.89**	-0.17	0.68	0.82	0.52
Hippocampus (lh)	0.70	0.66	0.66	0.59	0.82	0.76	0.64	0.60	**0.98**	0.93
Hippocampus (rh)	0.60	0.56	0.66	0.49	0.81	0.63	0.54	0.60	**0.94**	0.86
Globus pallidus (lh)	0.61	0.54			0.21			0.79	0.92	**0.93**
Globus pallidus (rh)	0.59	0.54			0.36			0.79	**0.91**	0.89
Thalamus (lh)	0.82	0.79			0.67			0.83	**0.98**	**0.98**
Thalamus (rh)	0.83	0.79			0.79			0.83	**0.98**	**0.98**
Inferior parietal (lh)		**0.78**						0.64		
Inferior parietal (rh)		0.76						**0.84**		
Postcentral (lh)		0.63						**0.85**		
Postcentral (rh)		0.59						**0.87**		
Precuneus (lh)		**0.79**						0.73		
Precuneus (rh)		0.72						**0.78**		
Superior parietal (lh)		0.72						**0.76**		
Superior parietal (rh)		0.63						**0.85**		
Supramarginal (lh)		**0.74**						0.70		
Supramarginal (rh)		0.68						**0.80**		

First eight rows: selected subcortical volumes.

*Last ten rows: cortical thicknesses of parietal lobe. Bold numbers highlight the best ICC for each row. r, Pearson's r; ICC, Intraclass correlation coefficient; FS, FreeSurfer; lh, left hemisphere; rh, right hemisphere*.

Morey et al. (55) compared automatic measurement by FSL/FIRST of the hippocampus and amygdala to expert hand tracing. A single expert rater with experience segmented the structures in MR images from 20 participants. The authors reported the numbers only combined for both hemispheres. With the CNN, we observed significantly better correlations for the amygdala (left = 0.83, right = 0.81 vs. FIRST = 0.24) and comparable results for the hippocampi.

Similar, Tae et al. (56) compared IBASPM to manual segmentations of hippocampi. The authors reported both Pearson's *r* and ICC. However, they used a different form of ICC (equivalent to Cronbach's alpha) measuring consistency and not agreement. Hence we are comparing using Pearson's *r*. With a dataset of 41 subjects consisting of controls and patients with chronic major depressive disorder, they reported lower correlations (left = 0.59, right = 0.49) than our CNN (left = 0.70, right = 0.60).

The FDA approved software NeuroQuant was compared to FreeSurfer by Ochs et al. (59). Initially developed as a commercial version of FreeSurfer, NeuroQuant meanwhile uses an independent code base and relies on a different probabilistic atlas. A total of 60 MRI scans (20 healthy, 20 Alzheimer's disease patients, and 20 mild traumatically brain-injured patients) were processed by both tools. The authors reported higher correlations for the volumes of the amygdalae and hippocampi, but lower correlations for the globus pallidi and thalami.

Using MR images from former professional football players, Guenette et al. (54) compared volumes of selected brain regions based on fully automated labels from FreeSurfer to manually corrected labels. Two trained raters manually corrected the labels from FreeSurfer in 108 subjects, followed by a review of a neuroanatomist. To assess inter-observer performance, 10 randomly chosen subjects were independently corrected by a third trained rater. Intraclass correlation coefficients for the inter-observer performance were generally higher compared to our CNN, except for the left amygdala (CNN = 0.79, inter-observer = 0.72). However, ICCs for the fully automated vs. manually corrected volumes were slightly lower for the hippocampus and significantly lower for the amygdala where the authors even reported negative values. Since correlation coefficients for the combined amygdala-hippocampal complex were good, the authors suspect a deviating definition of the border between the amygdala and hippocampus in FreeSurfer's atlas.

The test-retest reliability of FreeSurfer was assessed by Madan and Kensinger (57). Thirty young volunteers (20–30 years old) were scanned ten times within a 1-month period. The MR images were processed with FreeSurfer 5.3.0, and the reliability measured using ICC (both hemispheres combined for subcortical volumes). In agreement with our findings, they generally observed less reliable measures of the cortical thickness in the temporal lobe. Compared to the results of our CNN, ICCs for subcortical volumes were higher for the globus pallidi, thalami, and right hippocampus, but lower for the left hippocampus and both amygdalae. For the cortical thickness in the parietal lobe, they reported a higher ICC for seven parcellations and a lower ICC for three parcellations. An other test-retest experiment by Morey et al. (58) using four rescans for each of the 23 healthy subjects revealed higher ICCs with FS and FSL/FIRST for subcortical volumes.

### 3.6. Reliability

**Figure 10:**
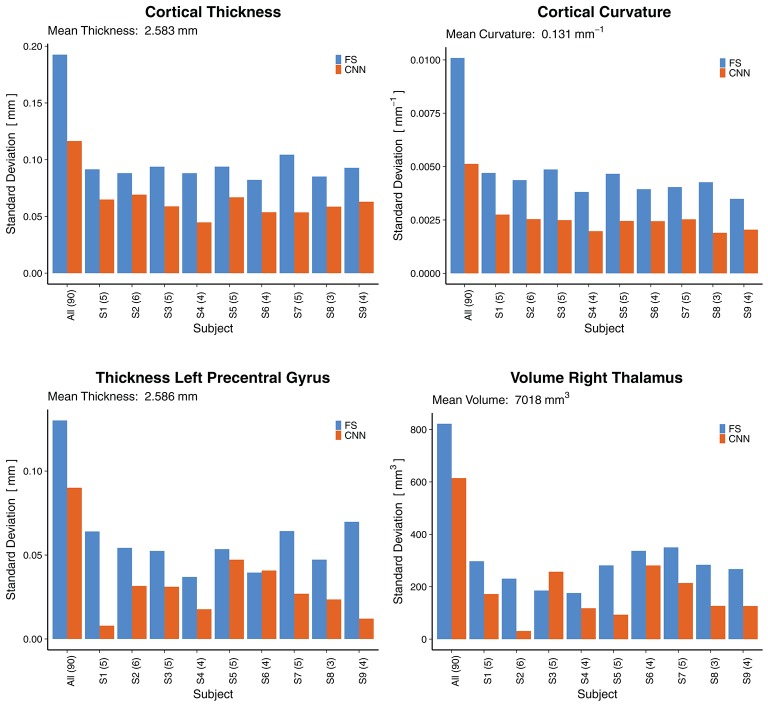
Reliability by means of rescan tests: Standard deviations (SD) across all 90 scans (leftmost bars) and for the nine subjects with available rescans. In parenthesis: Number of scans per subject. First row: SD for the cortical thickness and curvature, averaged over all 68 cortical parcellations. Second row: SD for two selected morphometrics. FS, FreeSurfer.

To assess the reliability of the method, we analyzed the predictions where several rescans of the same subject are available. Figure 10 shows the standard deviations (SD) across all 90 scans (leftmost bars) followed by the SD across rescans within each of the nine subjects separately. For the cortical thickness and curvature, the SD are reported as an average of all 68 parcellations. A general observation is that the SD across all 90 scans were lower for the CNN (±0.116 *mm* and ±0.005 *mm*^−1^ for thickness and curvature, respectively) than for FreeSurfer (±0.193 *mm*, ±0.010 *mm*^−1^). This suggests the CNN is unable to fully capture the inter-subject variance. Partially, this is probably due to some of the less accurate parcellations (they show less variance with a bias toward the mean), lowering the averaged SD.

When looking at selected morphometrics individually (second row in Figure 10, selected structures of interest for epilepsy), the SD of the CNN was closer to the one from FS. For the rescans, SD from the CNN were lower than those from FreeSurfer for all nine subjects, some significantly. A good to excellent accuracy for the volume of the right thalamus (ICC = 0.79 within *CI*_95%_ 0.70–0.86) comes along with good reliability for the rescans (SD below 4.1% for all subjects). As an example, the CNN predicted the following volumes for the right thalamus from the six scans of subject *S2*: 7,079, 7,066, 7,028, 7,010, 7,021, 7,003 *mm*^3^. This corresponds to an average of 7,035 *mm*^3^ and a standard deviation of 31 *mm*^3^. Whereas FreeSurfer reported an average volume of 7,011 *mm*^3^ with a standard deviation of 230 *mm*^3^ for the scans of the same subject. Corresponding reliability plots for the remaining structures can be found in the Supplementary Material.

## 4. Discussion

We have used data from 574 subjects, processed with FreeSurfer, for the supervised training of a CNN to predict brain morphometry from MRI. The customized CNN predicts a total of 165 morphometric measures (subcortical volumes, and cortical thicknesses and curvatures) directly from minimally pre-processed (skull-stripped) T1w MR images, without the need of prior image registration nor segmentation, enabling results to be available within seconds. With 438 samples in the training set, which is considered to be on the lower end for successfully training a deep learning model (80, 81), a simple data augmentation strategy of translations and rotations further improved the accuracy. Besides quantitative evaluations of the results, we have shown methods to assess the clinical relevance of the achieved accuracy (sections 3.3, 3.4 and 3.6) beyond correlation coefficients.

### 4.1. Convolutional Neural Network Architecture

Our aim of directly regressing all morphometric measures requires passing the entire 3D volume as input into the network, ruling out slice- or patch-based strategies. The large input size consequently constrains the network to simpler architectures, or otherwise would require special infrastructure to train large networks with high-resolution input (82). We have not performed an extensive architecture search, but explored different directions within the given constraints and found the proposed architecture suitable for the task to demonstrate the feasibility. Besides optimizing the network architecture, further improvements could be achieved by leveraging recent developments in how to deal with sparse or noisy labels in medical image analysis (83) of which semi- or self-supervised learning might be promising strategies (84).

The chosen data augmentation is effective, while still computationally efficient. Arbitrary rotations would require resampling, which is computationally expensive and might cause unwanted artifacts. Future work should also investigate contrast-related data augmentation techniques (random scale and shift of intensity distributions) to make the network more robust to scanner and sequence variations (85).

### 4.2. Evaluation

We consider intraclass correlation coefficients (ICC) to be the best suited quantitative evaluation metric for the given task, as it measures both, degree of correlation and agreement. Nevertheless, its interpretation is non-trivial. As we can infer from the general definition of ICC (ratio of true variance to true variance plus error variance), a low ICC could also relate to a lack of variability among subjects (70). Consequently, absolute values of ICC between categories should be compared with care, e.g., between subcortical volumes (naturally higher inter-subject variance) and cortical curvatures (lower inter-subject variance). Instead, the results should be contrasted with other established methods.

A fair, good or excellent ICC [according (71)] was reached for all 29 subcortical volumes and the vast majority (54 out of 68) of the cortical thicknesses. The reliability of the predictions for the cortical curvatures is questionable, with only about half of them (35/68) being in the range of fair and above. For the cortical structures, the lowest ICC were found in the temporal lobe, an observation that is also reported by Madan et al. in a reliability evaluation of FreeSurfer (57).

As we can see from the correlation plots, the CNN model was unable to capture the full variance of the *silver-standard* ground truth (trend toward the mean expected outcome). This observation is a known challenge in regression tasks (86) which are inevitably prone to the “*regression toward the mean*” effect (87) when optimizing a model by minimizing its prediction errors. The Bland-Altman plots revealed only a small bias from zero, but a tendency of the model to overestimate smaller values and underestimate the larger ones.

### 4.3. Patients With Epilepsy

Using morphometry predicted by the CNN, structural changes between healthy controls and patients with epilepsy were observed in our dataset, similar to the findings from the ENIGMA epilepsy study (21). Effect size differences were consistent for six out of eight regions. In case of the two deviating results for the right thalamus and globus pallidus, FreeSurfer is not in agreement with the findings from ENIGMA either. The cause is unknown, but might be related to the type of epilepsies in our dataset.

### 4.4. Age-Related Cortical Gray Matter Atrophy

Age-related gray matter atrophy is an extensively studied aspect of brain morphometry. Based on the predicted cortical thicknesses, a linear regression model revealed a statistically significant change of −0.004 mm/year in global average thickness for the population in our test set. Exactly the same value has been reported by Lemaitre et al. (78). Regionally, we found age-related atrophy to be less pronounced in the parcellations of the temporal lobe, which is in agreement with the literature (7, 78, 88). The cortical thickness of the entorhinal cortex was classified as less reliable from an ICC point of view, yet its age trend suggests a better correlation. A linear model suggested a slightly increasing thickness over the lifespan. A closer examination with a quadratic model revealed a remarkably similar pattern to what has been reported by Hasan et al. (79), namely an increasing thickness until around 45 years followed by a decrease again. It is worth highlighting again, that the age of the subjects is not part of the input data for the CNN.

### 4.5. Comparison With Others

No method can reasonably achieve a 100% accuracy for the given problem (MRI being a surrogate for the underlying anatomy, with a limited resolution and partial volume effects). Therefore, comparing a new method to well-established methods is common practice. We have contrasted the results to publications covering a variety of evaluation methods, such as manual tracing by experts, scan-rescan studies, and comparisons among different tools. The selected subcortical volumes and cortical thicknesses of the parietal lobe showed quite comparable magnitudes of intraclass correlation coefficients. Human inter-rater reliability for segmentation of hippocampi was reported (89) to be in the range of *ICC* = 0.73−0.85, which is considered as a reasonable upper bound on the accuracy of automated segmentation by Stein et al. (90). A comparison to other recently proposed fast methods (section 1) is not directly possible as these are either segmentation methods reporting the spatial overlap with Dice coefficients, or evaluation metrics for parcellation-wise averages are not available.

### 4.6. Limitations and Outlook

The lack of a gold-standard ground truth is one of the major challenges. Supervised training of a model with ground truth data generated by another method (in this case FreeSurfer) always leads to a bias toward the results from the tool, rather than the (unknown) true underlying values. The evaluation is limited to a comparison with the other method, in which the new model is unable to be superior to the baseline by definition. Furthermore, although FreeSurfer is a well-established and thoroughly validated tool, it is not immune to errors (in rare cases producing exceptionally large outliers). We have not performed any systematic quality control of the FreeSurfer output, such as visual inspection of the pial and white matter boundaries, neither on the training nor the test set.

Although we used data acquired on two different scanners, with four different MRI protocols, they are all from the same center (Inselspital). We have no indication how well the trained model would generalize to data from other centers. On one hand, morphometric measures derived from traditional voxel-based morphometry (VBM) are also known to be biased to site-specific variations (91). On the other hand, deep learning has shown its ability to generalize toward a range of acquisition settings in MRI (92). To what extent this applies to brain morphometry remains to be investigated. Although the data comprised of both healthy controls and patients with epilepsy, the behavior of the model on pathologies not present in the training data is unknown.

Despite progress to improve the interpretability of deep learning (93), deep neural networks are still considered, to a large extent, as black boxes (94). The difficulty to understand their decision-making-process poses a challenge in its adoption for medical applications (95), especially for direct classification and regression tasks. Future work should address the lack of visual inspection options for quality control, particularly for cortical thickness and curvature measures. For volumetric information of tissue classes and subcortical structures, a segmentation algorithm is probably still the preferred approach as it facilitates a visual verification of the results.

The efficacy of a deep learning-based approach for brain morphometry for clinical applications has yet to be shown, ideally on an individual patient level. We plan to further evaluate this novel approach along with other established and emerging morphometry methods on a larger scale, with a broader dataset from several centers including different neurodegenerative diseases.

## 5. Conclusions

We have shown the general feasibility of using deep learning to estimate human brain morphometry directly from MRI within seconds. To the best of our knowledge, this is currently the fastest reported solution to obtain subcortical and cortical morphometric measures from MRI. A trained CNN predicts a total of 165 morphometric measures within seconds, compared to several hours of traditional methods.

Analysis of the results using intraclass correlation coefficients and Bland-Altman plots showed, in general, good correlation with FreeSurfer generated *silver-standard* ground truth data. Some of the regions (namely subcortical volumes and cortical thicknesses in the parietal lobe) nearly reached human inter-observer performance.

Besides a good rescan reliability, further indications support the hypothesis of reaching an accuracy to be clinically relevant. Namely, (1) replication of the findings from the large-scale ENIGMA study to detect structural morphometric changes in patients with epilepsy, (2) observed cross-sectional annual age-related gray matter atrophy rates both globally and regionally in agreement with literature, and (3) contrasting the results with other publications reporting accuracies of comparable magnitudes.

## Data Availability Statement

The datasets used for this study cannot be made publicly available. The experiments were performed with data from patients and healthy controls of the Bern University Hospital. All study participants signed informed consent for the use of their data for research. However, this does not include permission to make the raw data publicly available. Code may be shared upon direct request.

## Ethics Statement

This study was carried out in accordance with the recommendations of Kantonale Ethikkommission Bern with written informed consent from all subjects. All subjects gave written informed consent in accordance with the Declaration of Helsinki. The protocol was approved by the Kantonale Ethikkommission Bern (protocol 2017-00697). Written informed consent to participate in this study was provided by the participants legal guardian/next of kin.

## Author Contributions

RW and MRey: conceive the project idea. MReb, CR, and YS: design of experiments. MReb: perform experiments, data analysis, and manuscript drafting. CR: manuscript revision. MReb, YS, and CR: result interpretation. All authors reviewed and approved the final version of the manuscript.

### Conflict of Interest

The authors declare that the research was conducted in the absence of any commercial or financial relationships that could be construed as a potential conflict of interest.
