# Supplementary material for: Brain Morphometry Estimation: From Hours to Seconds Using Deep Learning
Source: Front Neurol. 2020 Apr 8;11:244. doi: 10.3389/fneur.2020.00244 (PMC7156625; doi:10.3389/fneur.2020.00244)
Supplement: Supplementary file 1 [file Data_Sheet_1.PDF]

Supplementary Materials for Brain Morphometry Estimation: from Hours to Seconds using Deep Learning

Michael Rebsamen[1,2], Yannick Suter[3], Roland Wiest[1], Mauricio Reyes[3,4], and Christian Rummel[1]

[1]Support Center for Advanced Neuroimaging (SCAN), University Institute of Diagnostic and Interventional Neuroradiology, University of Bern, Inselspital, Bern University Hospital, Bern, Switzerland
[2]Graduate School for Cellular and Biomedical Sciences, University of Bern, Bern, Switzerland
[3]Insel Data Science Center, Inselspital, Bern University Hospital, Bern, Switzerland
[4]ARTORG Center for Biomedical Research, University of Bern, Bern, Switzerland

Contents

1 Model Training **2**

2 Results for all Morphometrics **2**

3 Plots for all Morphometrics **4**
3.1 Correlation and Bland-Altman Plots for Subcortical Volume 4
3.2 Correlation and Bland-Altman Plots for Cortical Thickness 14
3.3 Correlation and Bland-Altman Plots for Cortical Curvature 37
3.4 Cortical Thickness against Age Plots 60

4 Reliability **72**
4.1 Reliability Plots for Subcortical Volume 72
4.2 Reliability Plots for Cortical Thickness 77
4.3 Reliability Plots for Cortical Curvature 89

1 Model Training

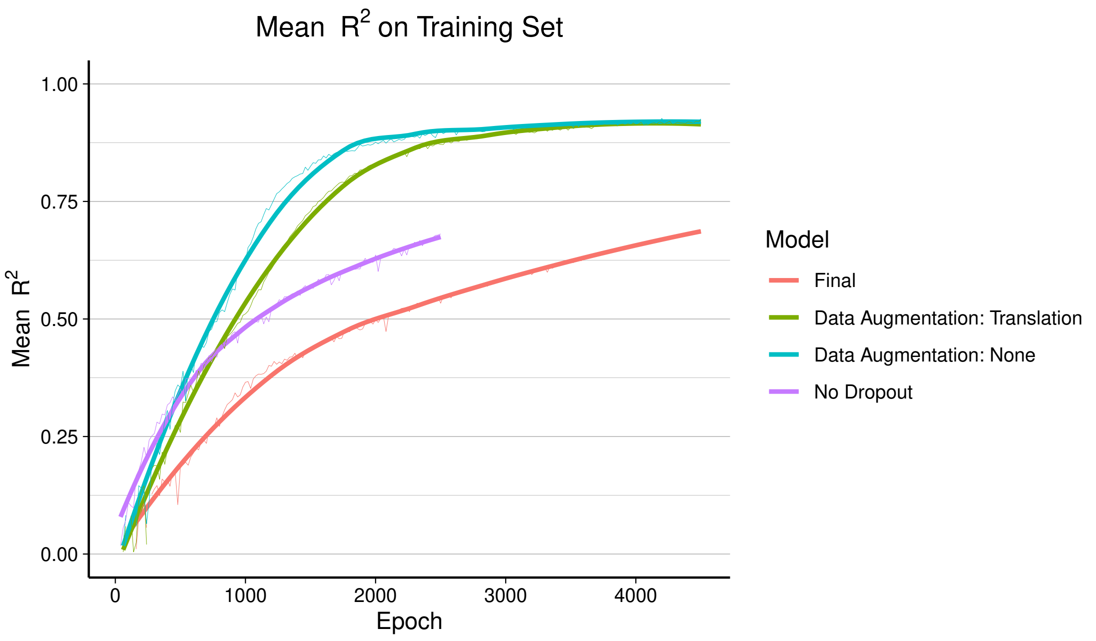

Figure S1: Mean R^2 of all 165 regression metrics on the **training** data. The thick line is the smoothed curve of the data points evaluated every 20th epoch (thin line). Final model: Dropout and data augmentation with rotations and translations. Green and blue model include dropout.

2 Results for all Morphometrics

In the tables below, all numbers are rounded to two decimals for space reasons. The categorization of ICC in the main manuscript is based on raw numbers (e.g. ICC of right cerebellar white matter = 0.59965, hence categorized as *fair*).

Table S1: Result Overview Subcortex. All results in terms of R squared (R^2), Pearson's (r) and intraclass correlation coefficient (ICC) with 95% confidence interval (CI), both for left (lh) and right (rh) hemisphere.

		R^2		r		ICC (95% CI)	
		lh	rh	lh	rh	lh	rh
Subcortical Volumes	Amygdala	0.66	0.64	0.83	0.81	0.79 (0.69-0.86)	0.76 (0.66-0.84)
	Caudate nucleus	0.43	0.38	0.67	0.64	0.59 (0.43-0.71)	0.52 (0.36-0.66)
	Cerebellar cortex	0.54	0.57	0.76	0.76	0.74 (0.63-0.82)	0.74 (0.63-0.82)
	Cerebellar white matter	0.40	0.41	0.66	0.65	0.56 (0.40-0.69)	0.60 (0.45-0.72)
	Globus pallidus	0.38	0.34	0.61	0.59	0.54 (0.37-0.67)	0.54 (0.38-0.67)
	Hippocampus	0.47	0.35	0.70	0.60	0.66 (0.53-0.76)	0.56 (0.40-0.69)
	Inferior horn of lateral ventricle	0.43	0.25	0.66	0.51	0.64 (0.50-0.75)	0.46 (0.28-0.61)
	Lateral ventricle	0.77	0.84	0.88	0.93	0.87 (0.81-0.91)	0.90 (0.85-0.93)
	Nucleus accumbens	0.65	0.45	0.81	0.69	0.79 (0.70-0.86)	0.68 (0.55-0.78)
	Putamen	0.47	0.50	0.71	0.73	0.59 (0.44-0.71)	0.63 (0.48-0.74)
	Thalamus	0.65	0.67	0.82	0.83	0.79 (0.69-0.85)	0.79 (0.70-0.86)
	Ventral diencephalon	0.66	0.60	0.82	0.77	0.78 (0.68-0.85)	0.74 (0.63-0.82)
	Brainstem	0.65		0.81		0.78 (0.68-0.85)	
	Cerebral white matter	0.84		0.93		0.90 (0.85-0.93)	
	Corpus callosum	0.33		0.59		0.47 (0.30-0.62)	
	Total gray matter	0.84		0.92		0.91 (0.86-0.94)	
	White matter hypointensities	0.25		0.51		0.40 (0.21-0.56)	

Table S2: Result Overview Cortex. All results in terms of R squared (R^2), Pearson's (r) and intraclass correlation coefficient (ICC) with 95% confidence interval (CI), both for left (lh) and right (rh) hemisphere.

		R^2		r		ICC (95% CI)	
		lh	rh	lh	rh	lh	rh
Cortical Thickness	Banks of the superior temporal sulcus	0.55	0.39	0.75	0.69	0.73 (0.62-0.81)	0.67 (0.53-0.77)
	Caudal anterior cingulate cortex	0.13	0.17	0.37	0.47	0.26 (0.06-0.44)	0.41 (0.23-0.57)
	Caudal middle frontal gyrus	0.36	0.37	0.63	0.64	0.56 (0.40-0.69)	0.60 (0.45-0.72)
	Cuneus	0.33	0.30	0.60	0.56	0.51 (0.34-0.65)	0.49 (0.32-0.64)
	Entorhinal cortex	-0.23	0.05	0.08	0.28	0.06 (-0.13-0.26)	0.20 (-0.01-0.39)
	Frontal pole	0.30	0.12	0.56	0.47	0.46 (0.28-0.61)	0.33 (0.14-0.50)
	Fusiform gyrus	0.13	0.41	0.40	0.66	0.34 (0.14-0.51)	0.59 (0.44-0.71)
	Inferior parietal lobule	0.65	0.60	0.82	0.78	0.78 (0.68-0.85)	0.76 (0.66-0.84)
	Inferior temporal gyrus	0.34	0.32	0.62	0.57	0.57 (0.42-0.70)	0.51 (0.34-0.65)
	Insular cortex	0.26	0.43	0.52	0.66	0.45 (0.27-0.60)	0.60 (0.45-0.72)
	Isthmus of the cingulate cortex	0.17	0.31	0.47	0.62	0.36 (0.17-0.53)	0.52 (0.34-0.66)
	Lateral occipital cortex	0.19	0.25	0.47	0.54	0.38 (0.19-0.54)	0.44 (0.26-0.59)
	Lateral orbitofrontal cortex	0.33	0.38	0.58	0.63	0.52 (0.35-0.65)	0.51 (0.34-0.65)
	Lingual gyrus	0.42	0.49	0.65	0.71	0.60 (0.45-0.72)	0.63 (0.49-0.74)
	Medial orbitofrontal cortex	0.17	0.30	0.48	0.55	0.44 (0.26-0.59)	0.46 (0.28-0.61)
	Middle temporal gyrus	0.43	0.58	0.69	0.78	0.68 (0.55-0.77)	0.77 (0.66-0.84)
	Paracentral lobule	0.32	0.25	0.57	0.50	0.51 (0.34-0.65)	0.41 (0.23-0.57)
	Parahippocampal gyrus	0.10	0.12	0.35	0.43	0.24 (0.03-0.42)	0.30 (0.10-0.47)
	Pars opercularis	0.49	0.47	0.70	0.71	0.66 (0.53-0.76)	0.71 (0.59-0.80)
	Pars orbitalis	0.38	0.21	0.63	0.47	0.51 (0.33-0.64)	0.42 (0.24-0.58)
	Pars triangularis	0.40	0.51	0.65	0.72	0.63 (0.49-0.74)	0.69 (0.56-0.78)
	Pericalcerine cortex	0.33	0.35	0.57	0.61	0.49 (0.31-0.63)	0.52 (0.36-0.66)
	Postcentral gyrus	0.47	0.46	0.70	0.69	0.63 (0.49-0.74)	0.59 (0.44-0.71)
	Posterior cingulate cortex	0.30	0.12	0.57	0.47	0.45 (0.27-0.60)	0.39 (0.20-0.55)
	Precentral gyrus	0.32	0.23	0.58	0.49	0.54 (0.38-0.67)	0.41 (0.23-0.57)
	Precuneus	0.68	0.59	0.84	0.78	0.79 (0.70-0.86)	0.72 (0.61-0.81)
	Rostral anterior cingulate cortex	0.28	0.23	0.53	0.49	0.45 (0.27-0.60)	0.39 (0.20-0.55)
	Rostral middle frontal cortex	0.56	0.60	0.75	0.78	0.73 (0.62-0.82)	0.76 (0.66-0.84)
	Superior frontal gyrus	0.58	0.55	0.77	0.75	0.74 (0.64-0.82)	0.73 (0.62-0.81)
	Superior parietal lobule	0.61	0.49	0.79	0.72	0.72 (0.61-0.81)	0.63 (0.49-0.74)
	Superior temporal gyrus	0.53	0.56	0.73	0.76	0.70 (0.57-0.79)	0.69 (0.57-0.79)
	Supramarginal gyrus	0.58	0.49	0.77	0.71	0.74 (0.63-0.82)	0.68 (0.55-0.78)
	Temporal pole	-0.02	0.20	0.17	0.45	0.10 (-0.10-0.30)	0.30 (0.10-0.48)
	Transverse temporal gyrus	0.16	0.50	0.46	0.72	0.38 (0.19-0.54)	0.64 (0.50-0.75)
Cortical Curvature	Banks of the superior temporal sulcus	0.20	-0.06	0.47	0.34	0.33 (0.13-0.50)	0.19 (-0.00-0.37)
	Caudal anterior cingulate cortex	-0.04	0.04	0.29	0.31	0.17 (-0.02-0.35)	0.24 (0.04-0.43)
	Caudal middle frontal gyrus	0.40	0.40	0.65	0.63	0.55 (0.39-0.68)	0.59 (0.44-0.71)
	Cuneus	0.30	0.36	0.55	0.63	0.44 (0.25-0.59)	0.48 (0.30-0.62)
	Entorhinal cortex	0.18	0.12	0.45	0.40	0.37 (0.18-0.53)	0.27 (0.07-0.45)
	Frontal pole	0.05	0.17	0.32	0.44	0.23 (0.03-0.41)	0.32 (0.12-0.49)
	Fusiform gyrus	0.26	0.16	0.53	0.54	0.48 (0.31-0.62)	0.49 (0.30-0.64)
	Inferior parietal lobule	0.29	0.28	0.53	0.56	0.45 (0.27-0.60)	0.48 (0.30-0.62)
	Inferior temporal gyrus	0.28	0.33	0.55	0.60	0.47 (0.29-0.61)	0.51 (0.34-0.65)
	Insular cortex	0.16	0.15	0.43	0.41	0.36 (0.17-0.53)	0.35 (0.15-0.52)
	Isthmus of the cingulate cortex	-0.06	-0.15	0.19	0.12	0.16 (-0.05-0.35)	0.08 (-0.11-0.28)
	Lateral occipital cortex	0.19	0.43	0.48	0.66	0.43 (0.24-0.58)	0.59 (0.43-0.71)
	Lateral orbitofrontal cortex	0.18	0.13	0.46	0.41	0.36 (0.17-0.53)	0.33 (0.14-0.50)
	Lingual gyrus	0.32	0.25	0.59	0.50	0.44 (0.25-0.59)	0.40 (0.21-0.56)
	Medial orbitofrontal cortex	0.15	0.21	0.41	0.46	0.31 (0.11-0.49)	0.34 (0.15-0.51)
	Middle temporal gyrus	0.24	0.35	0.49	0.64	0.43 (0.25-0.59)	0.54 (0.37-0.67)
	Paracentral lobule	0.28	0.40	0.54	0.64	0.47 (0.29-0.62)	0.56 (0.40-0.69)
	Parahippocampal gyrus	0.13	0.49	0.39	0.75	0.31 (0.12-0.49)	0.60 (0.45-0.72)
	Pars opercularis	0.29	0.30	0.58	0.56	0.37 (0.18-0.54)	0.47 (0.30-0.62)
	Pars orbitalis	0.16	0.22	0.41	0.47	0.26 (0.05-0.44)	0.37 (0.17-0.53)
	Pars triangularis	0.19	0.21	0.47	0.58	0.28 (0.08-0.46)	0.40 (0.20-0.57)
	Pericalcerine cortex	-0.02	0.01	0.27	0.30	0.23 (0.03-0.42)	0.26 (0.06-0.45)
	Postcentral gyrus	0.51	0.46	0.72	0.68	0.68 (0.55-0.78)	0.61 (0.47-0.73)
	Posterior cingulate cortex	0.17	0.12	0.41	0.36	0.28 (0.08-0.46)	0.27 (0.06-0.45)
	Precentral gyrus	0.51	0.55	0.72	0.75	0.69 (0.56-0.78)	0.71 (0.59-0.80)
	Precuneus	0.27	0.30	0.55	0.56	0.46 (0.28-0.61)	0.46 (0.28-0.61)
	Rostral anterior cingulate cortex	-0.02	0.07	0.14	0.36	0.08 (-0.13-0.28)	0.21 (0.01-0.39)
	Rostral middle frontal cortex	0.15	0.29	0.44	0.56	0.40 (0.21-0.56)	0.50 (0.33-0.64)
	Superior frontal gyrus	0.34	0.28	0.60	0.53	0.58 (0.43-0.70)	0.48 (0.30-0.62)
	Superior parietal lobule	0.20	0.30	0.48	0.58	0.42 (0.24-0.58)	0.55 (0.39-0.68)
	Superior temporal gyrus	-0.10	0.19	0.20	0.60	0.16 (-0.04-0.35)	0.47 (0.24-0.64)
	Supramarginal gyrus	0.29	0.34	0.54	0.60	0.50 (0.32-0.64)	0.46 (0.28-0.61)
	Temporal pole	0.04	-0.07	0.26	0.25	0.17 (-0.03-0.36)	0.23 (0.02-0.41)
	Transverse temporal gyrus	0.28	-0.01	0.54	0.29	0.39 (0.19-0.55)	0.19 (-0.00-0.38)

3 Plots for all Morphometrics

Listed below are correlation and Bland-Altman plots for all morphometrics. Intraclass correlation coefficients (ICC) with 95% confidence intervals are reported in the subtitles. The last section shows the predictions of the cortical thickness together with ground truth plotted against age.
lh: left hemisphere, rh: right hemisphere, HC: healthy controls, EPI: patients with epilepsy.

3.1 Correlation and Bland-Altman Plots for Subcortical Volume

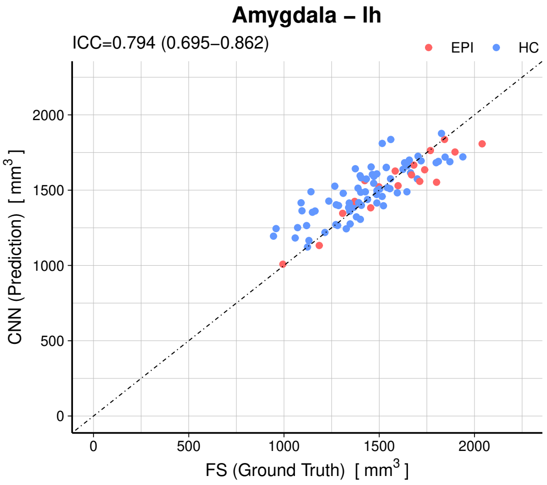

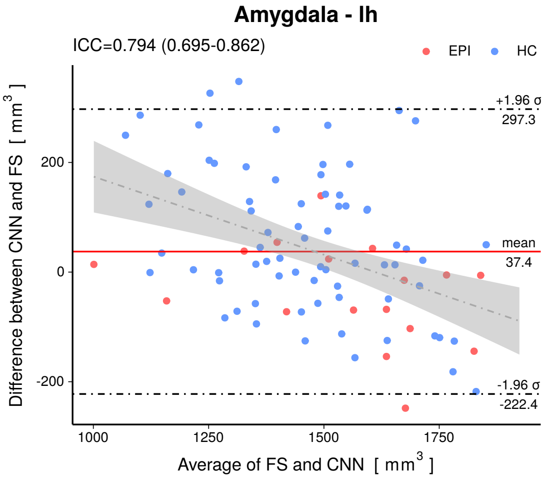

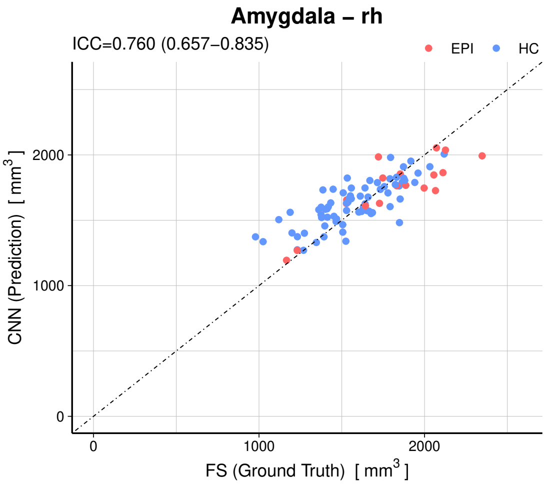

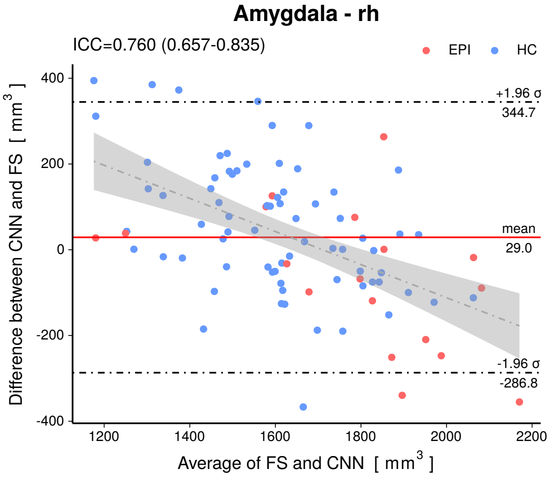

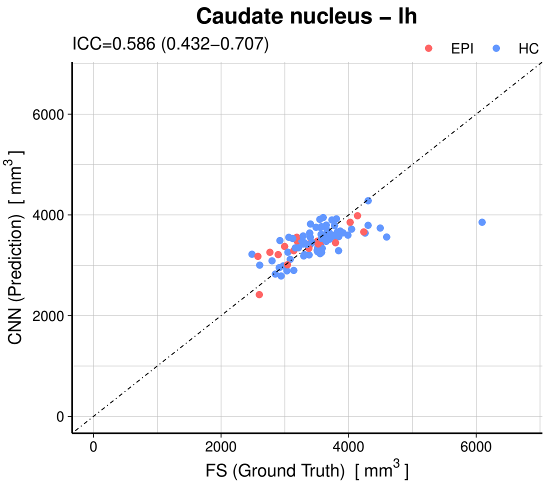
Caudate nucleus – lh
ICC=0.586 (0.432–0.707)
EPI
HC
CNN (Prediction) [mm3]
FS (Ground Truth) [mm3]
0
2000
4000
6000

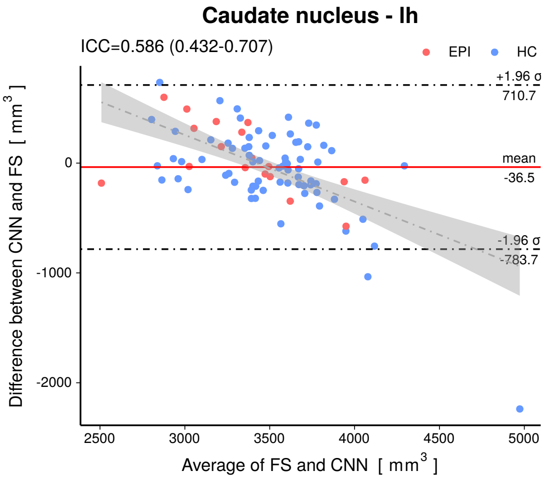
Caudate nucleus - lh
ICC=0.586 (0.432-0.707)
EPI
HC
+1.96 σ
710.7
mean
-36.5
-1.96 σ
-783.7
Difference between CNN and FS [mm3]
Average of FS and CNN [mm3]
2500
3000
3500
4000
4500
5000
0
-1000
-2000

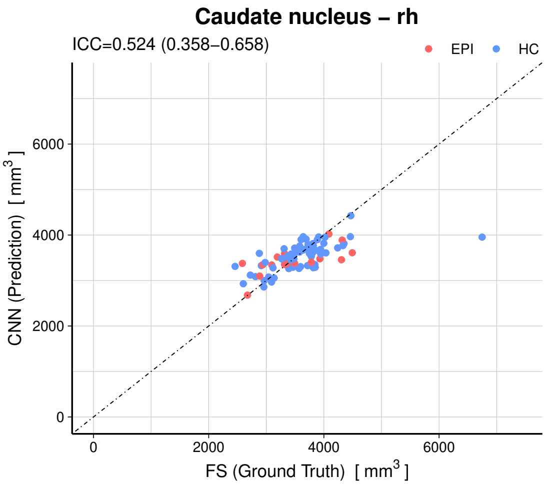
Caudate nucleus – rh
ICC=0.524 (0.358–0.658)
EPI
HC
CNN (Prediction) [mm3]
FS (Ground Truth) [mm3]
0
2000
4000
6000

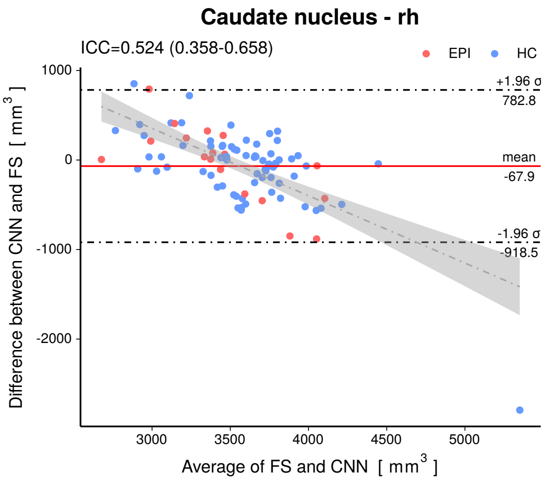
Caudate nucleus - rh
ICC=0.524 (0.358-0.658)
EPI
HC
+1.96 σ
782.8
mean
-67.9
-1.96 σ
-918.5
Difference between CNN and FS [mm3]
Average of FS and CNN [mm3]
3000
3500
4000
4500
5000
1000
0
-1000
-2000

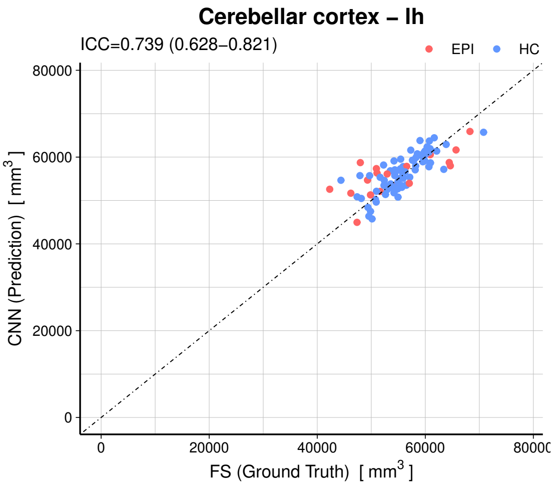
Cerebellar cortex – lh
ICC=0.739 (0.628–0.821)
EPI
HC
CNN (Prediction) [mm3]
FS (Ground Truth) [mm3]
0
20000
40000
60000
80000

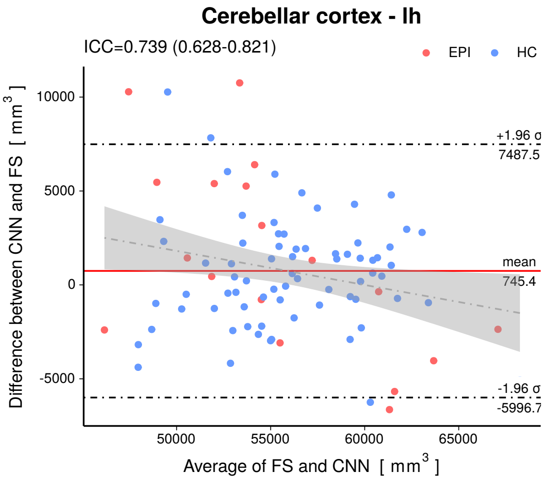
Cerebellar cortex - lh
ICC=0.739 (0.628-0.821)
EPI
HC
+1.96 σ
7487.5
mean
745.4
-1.96 σ
-5996.7
Difference between CNN and FS [mm3]
Average of FS and CNN [mm3]
50000
55000
60000
65000
10000
5000
0
-5000

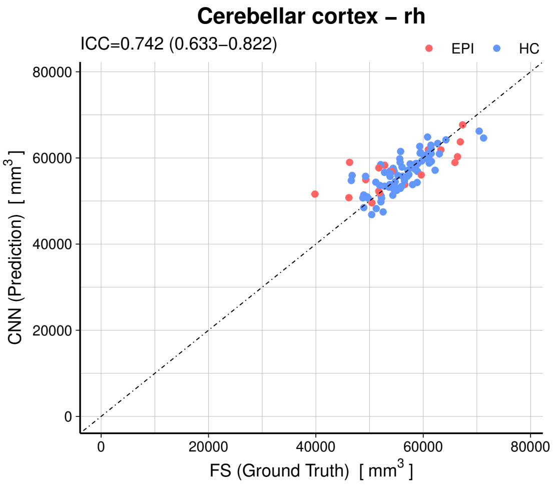
Cerebellar cortex – rh
ICC=0.742 (0.633–0.822)
EPI
HC
CNN (Prediction) [mm3]
FS (Ground Truth) [mm3]

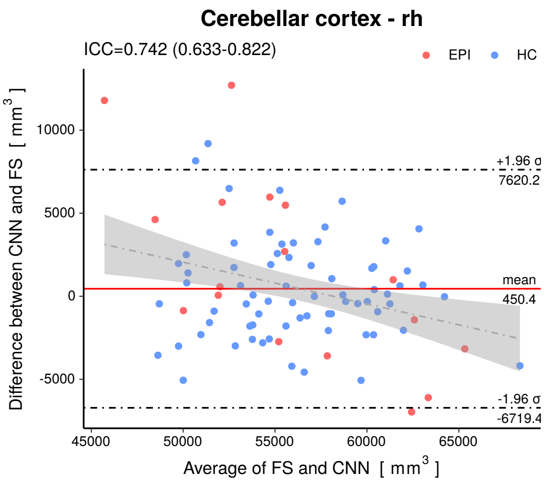
Cerebellar cortex - rh
ICC=0.742 (0.633-0.822)
EPI
HC
+1.96 σ
7620.2
mean
450.4
-1.96 σ
-6719.4
Difference between CNN and FS [mm3]
Average of FS and CNN [mm3]

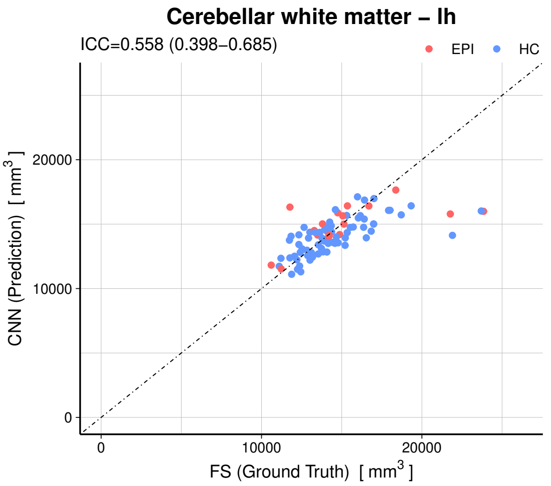
Cerebellar white matter – lh
ICC=0.558 (0.398–0.685)
EPI
HC
CNN (Prediction) [mm3]
FS (Ground Truth) [mm3]

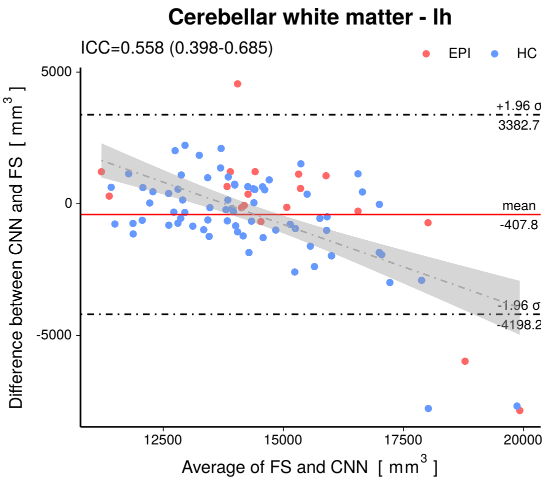
Cerebellar white matter - lh
ICC=0.558 (0.398-0.685)
EPI
HC
+1.96 σ
3382.7
mean
-407.8
-1.96 σ
-4198.2
Difference between CNN and FS [mm3]
Average of FS and CNN [mm3]

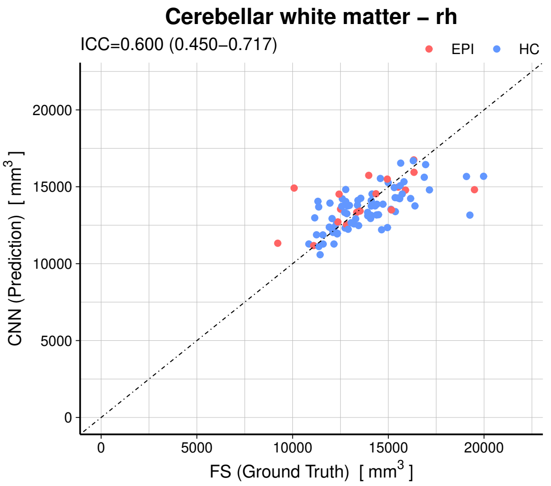
Cerebellar white matter – rh
ICC=0.600 (0.450–0.717)
EPI
HC
CNN (Prediction) [mm3]
FS (Ground Truth) [mm3]

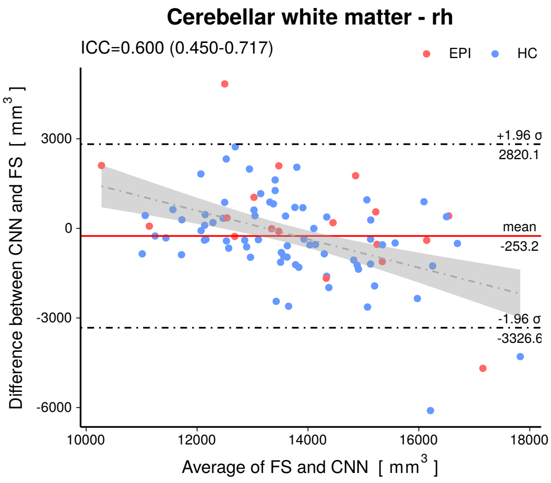
Cerebellar white matter - rh
ICC=0.600 (0.450-0.717)
EPI
HC
+1.96 σ
2820.1
mean
-253.2
-1.96 σ
-3326.6
Difference between CNN and FS [mm3]
Average of FS and CNN [mm3]

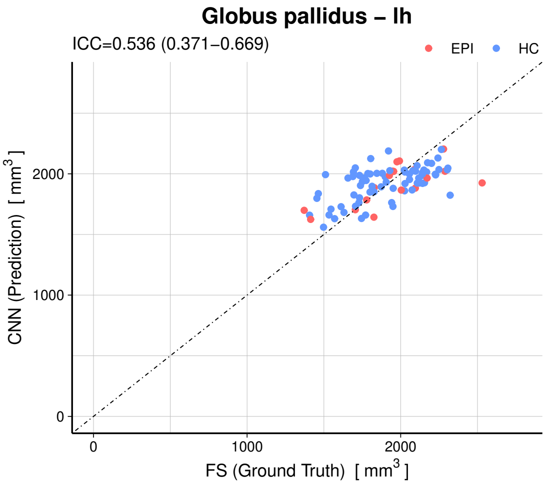
Globus pallidus – lh
ICC=0.536 (0.371–0.669)
EPI
HC
CNN (Prediction) [mm3]
FS (Ground Truth) [mm3]
0
1000
2000

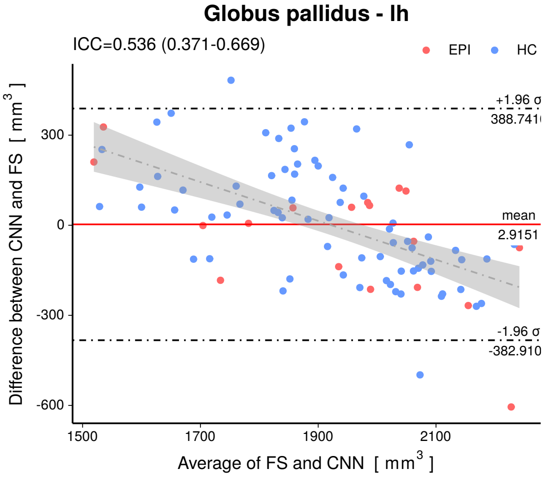
Globus pallidus - lh
ICC=0.536 (0.371-0.669)
EPI
HC
+1.96 σ
388.741
mean
2.9151
-1.96 σ
-382.910
Difference between CNN and FS [mm3]
Average of FS and CNN [mm3]
300
0
-300
-600
1500
1700
1900
2100

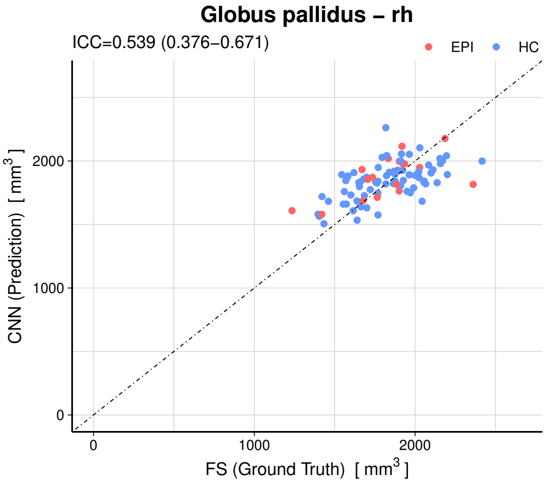
Globus pallidus – rh
ICC=0.539 (0.376–0.671)
EPI
HC
CNN (Prediction) [mm3]
FS (Ground Truth) [mm3]
0
1000
2000

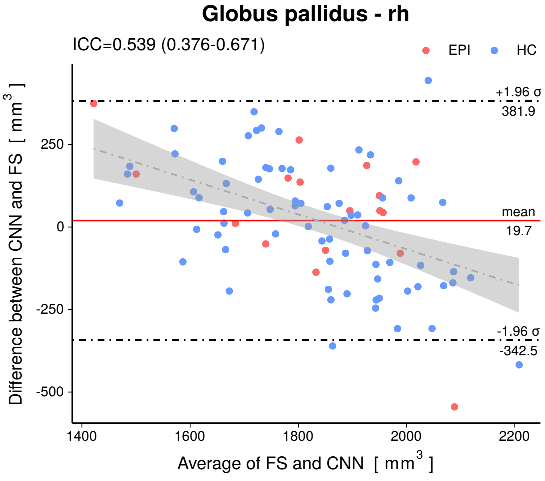
Globus pallidus - rh
ICC=0.539 (0.376-0.671)
EPI
HC
+1.96 σ
381.9
mean
19.7
-1.96 σ
-342.5
Difference between CNN and FS [mm3]
Average of FS and CNN [mm3]
250
0
-250
-500
1400
1600
1800
2000
2200

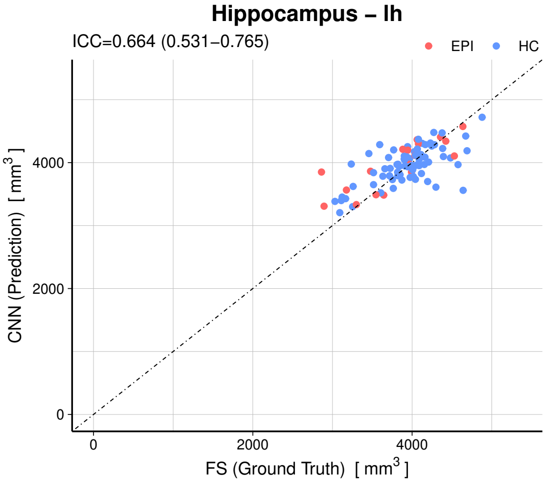
Hippocampus – lh
ICC=0.664 (0.531–0.765)
EPI
HC
CNN (Prediction) [mm3]
FS (Ground Truth) [mm3]
0
2000
4000

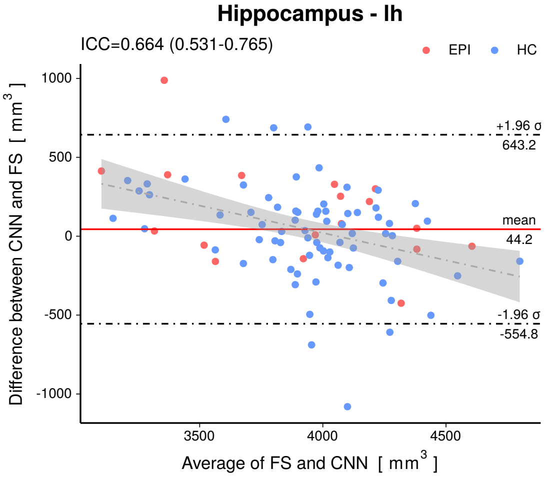
Hippocampus - lh
ICC=0.664 (0.531-0.765)
EPI
HC
+1.96 σ
643.2
mean
44.2
-1.96 σ
-554.8
Difference between CNN and FS [mm3]
Average of FS and CNN [mm3]
1000
500
0
-500
-1000
3500
4000
4500

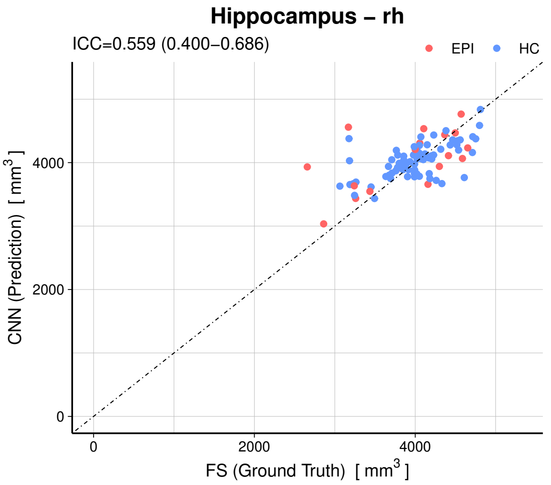
Hippocampus – rh
ICC=0.559 (0.400–0.686)
EPI
HC
CNN (Prediction) [mm3]
FS (Ground Truth) [mm3]
0
2000
4000

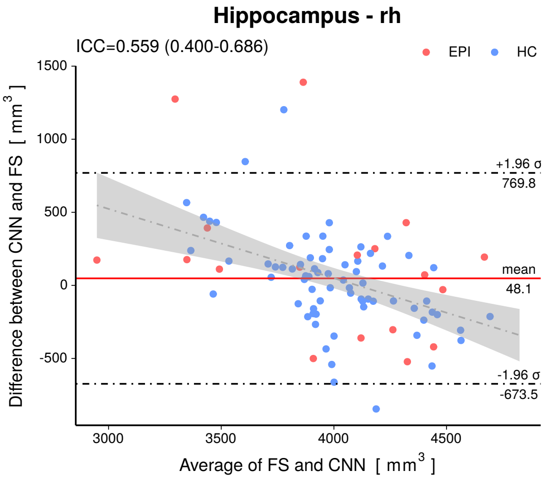
Hippocampus - rh
ICC=0.559 (0.400-0.686)
EPI
HC
Difference between CNN and FS [mm3]
Average of FS and CNN [mm3]
+1.96 σ
769.8
mean
48.1
-1.96 σ
-673.5
1500
1000
500
0
-500
3000
3500
4000
4500

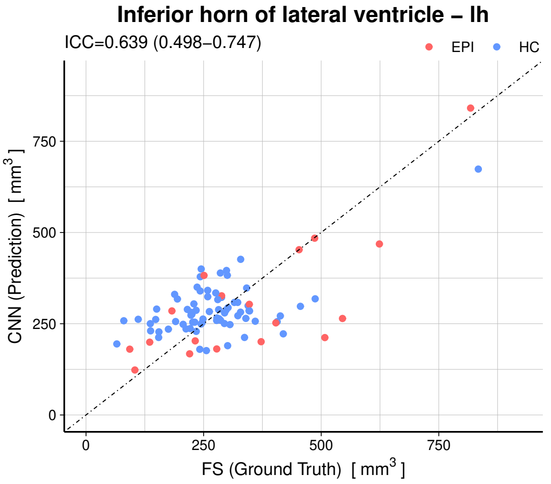
Inferior horn of lateral ventricle – lh
ICC=0.639 (0.498–0.747)
EPI
HC
CNN (Prediction) [mm3]
FS (Ground Truth) [mm3]
0
250
500
750

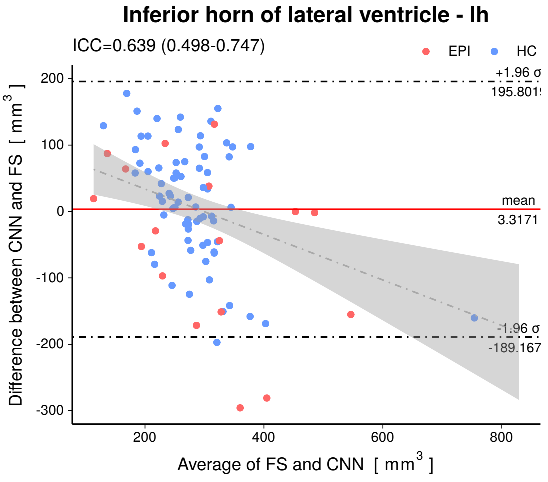
Inferior horn of lateral ventricle - lh
ICC=0.639 (0.498-0.747)
EPI
HC
Difference between CNN and FS [mm3]
Average of FS and CNN [mm3]
+1.96 σ
195.801
mean
3.3171
-1.96 σ
-189.167
200
100
0
-100
-200
-300
200
400
600
800

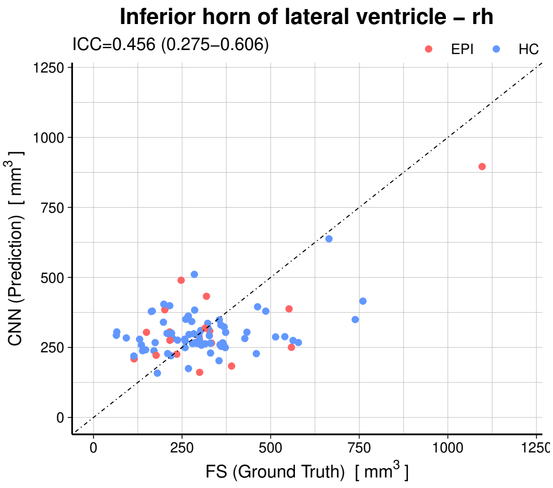
Inferior horn of lateral ventricle – rh
ICC=0.456 (0.275–0.606)
EPI
HC
CNN (Prediction) [mm3]
FS (Ground Truth) [mm3]
0
250
500
750
1000
1250

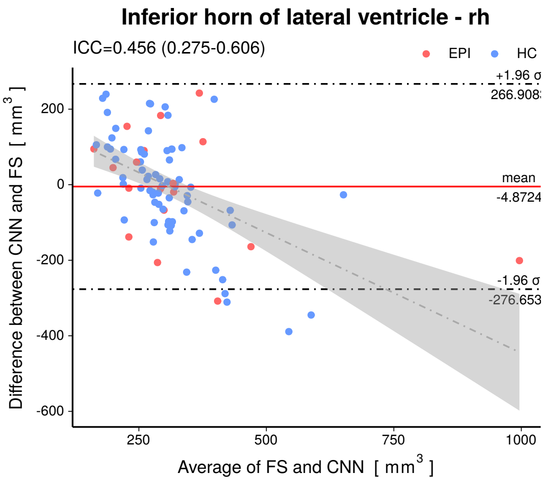
Inferior horn of lateral ventricle - rh
ICC=0.456 (0.275-0.606)
EPI
HC
Difference between CNN and FS [mm3]
Average of FS and CNN [mm3]
+1.96 σ
266.908
mean
-4.8724
-1.96 σ
-276.653
200
0
-200
-400
-600
250
500
750
1000

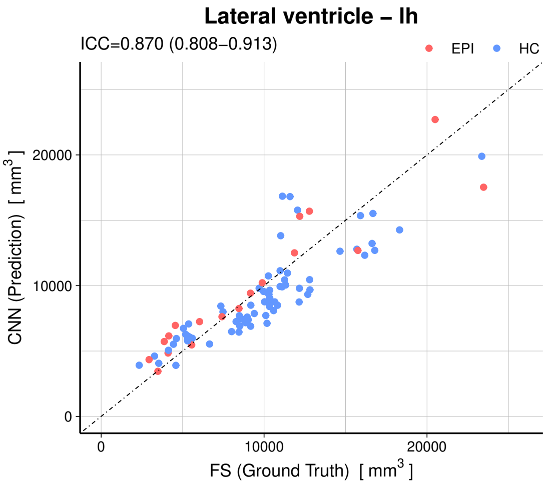
Lateral ventricle – lh
ICC=0.870 (0.808–0.913)
EPI
HC
CNN (Prediction) [mm3]
FS (Ground Truth) [mm3]
0
10000
20000

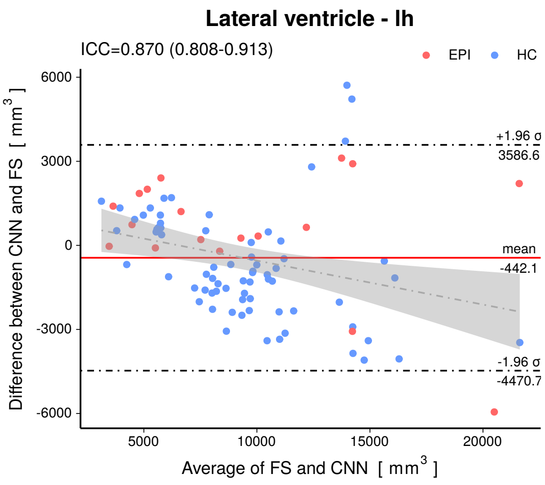
Lateral ventricle - lh
ICC=0.870 (0.808-0.913)
EPI
HC
+1.96 σ
3586.6
mean
-442.1
-1.96 σ
-4470.7
Difference between CNN and FS [mm3]
Average of FS and CNN [mm3]
6000
3000
0
-3000
-6000
5000
10000
15000
20000

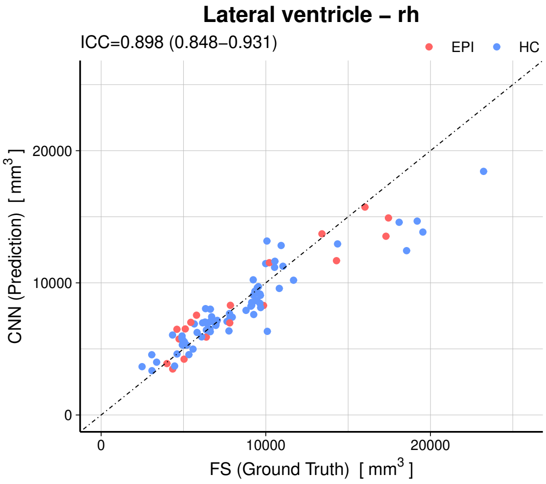
Lateral ventricle – rh
ICC=0.898 (0.848–0.931)
EPI
HC
CNN (Prediction) [mm3]
FS (Ground Truth) [mm3]
0
10000
20000

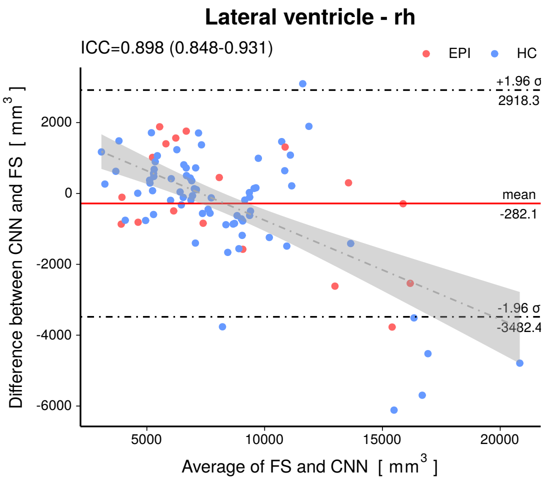
Lateral ventricle - rh
ICC=0.898 (0.848-0.931)
EPI
HC
+1.96 σ
2918.3
mean
-282.1
-1.96 σ
-3482.4
Difference between CNN and FS [mm3]
Average of FS and CNN [mm3]
2000
0
-2000
-4000
-6000
5000
10000
15000
20000

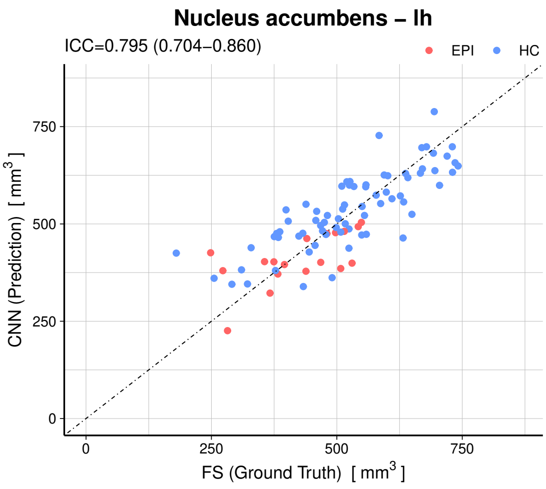
Nucleus accumbens – lh
ICC=0.795 (0.704–0.860)
EPI
HC
CNN (Prediction) [mm3]
FS (Ground Truth) [mm3]
0
250
500
750

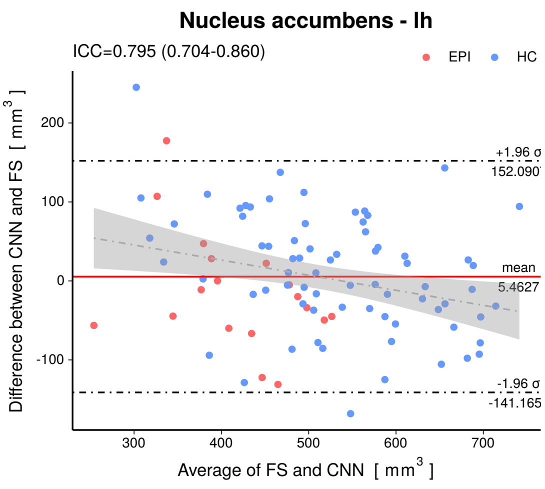
Nucleus accumbens - lh
ICC=0.795 (0.704-0.860)
EPI
HC
+1.96 σ
152.090
mean
5.4627
-1.96 σ
-141.165
Difference between CNN and FS [mm3]
Average of FS and CNN [mm3]
200
100
0
-100
300
400
500
600
700

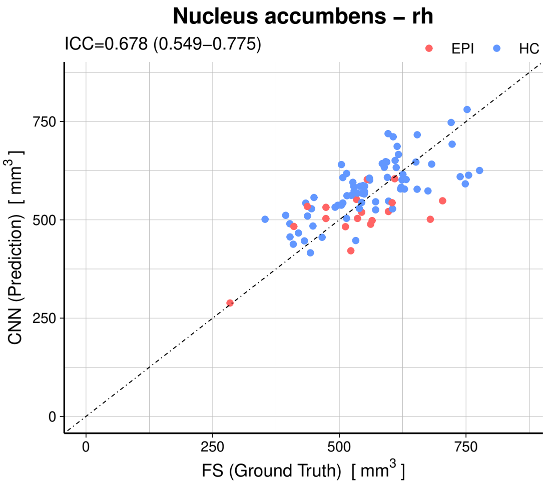
Nucleus accumbens – rh
ICC=0.678 (0.549–0.775)
EPI
HC
CNN (Prediction) [mm^3]
FS (Ground Truth) [mm^3]

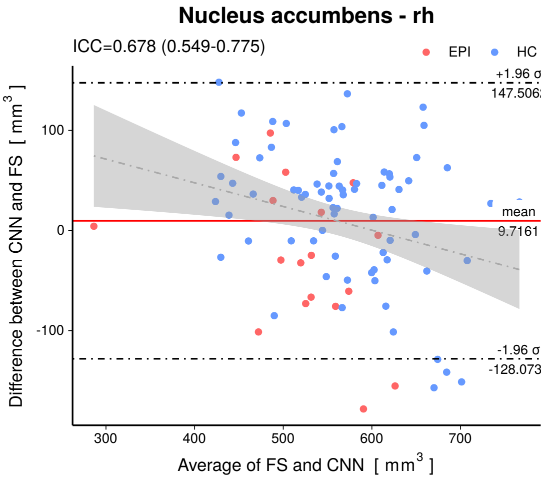
Nucleus accumbens - rh
ICC=0.678 (0.549-0.775)
EPI
HC
+1.96 σ
147.506
mean
9.7161
-1.96 σ
-128.073
Difference between CNN and FS [mm^3]
Average of FS and CNN [mm^3]

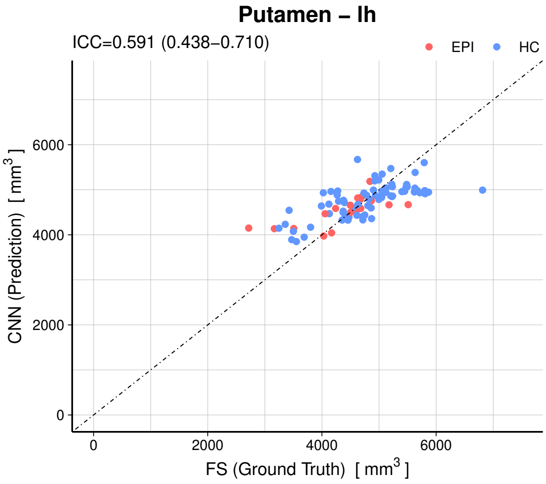
Putamen – lh
ICC=0.591 (0.438–0.710)
EPI
HC
CNN (Prediction) [mm^3]
FS (Ground Truth) [mm^3]

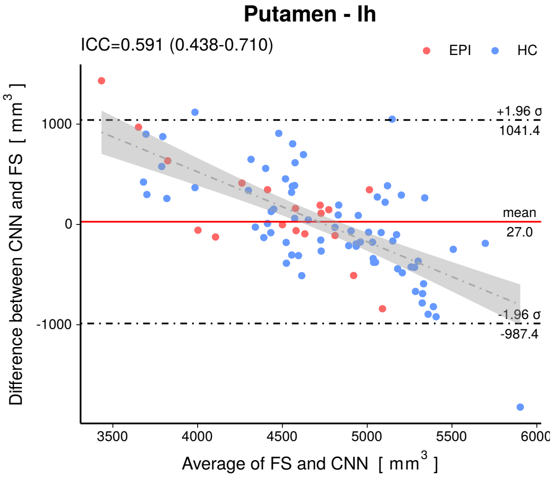
Putamen - lh
ICC=0.591 (0.438-0.710)
EPI
HC
+1.96 σ
1041.4
mean
27.0
-1.96 σ
-987.4
Difference between CNN and FS [mm^3]
Average of FS and CNN [mm^3]

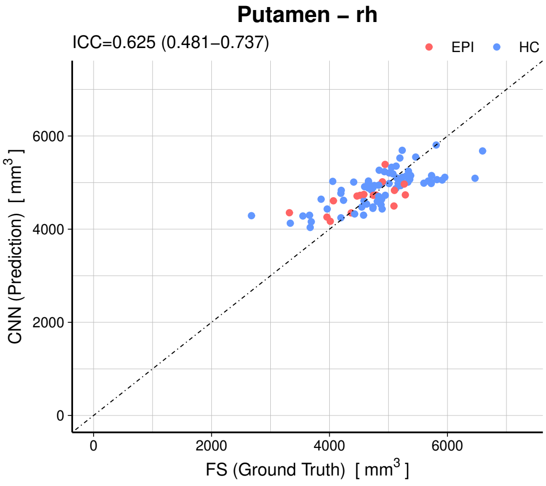
Putamen – rh
ICC=0.625 (0.481–0.737)
EPI
HC
CNN (Prediction) [mm^3]
FS (Ground Truth) [mm^3]

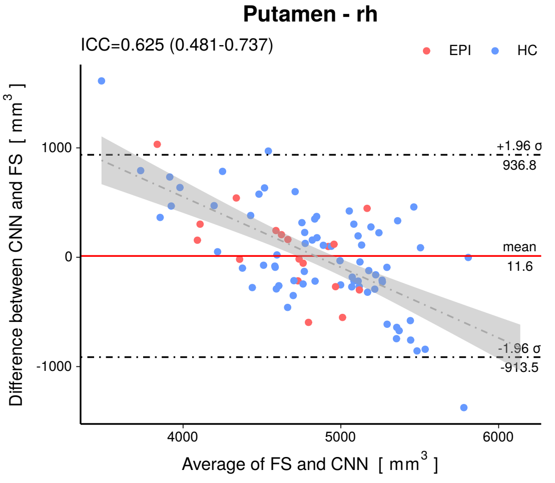
Putamen - rh
ICC=0.625 (0.481-0.737)
EPI
HC
+1.96 σ
936.8
mean
11.6
-1.96 σ
-913.5
Difference between CNN and FS [mm^3]
Average of FS and CNN [mm^3]

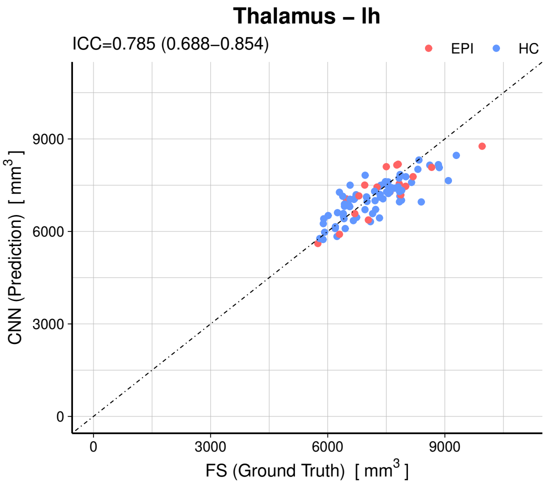

Thalamus – lh
ICC=0.785 (0.688–0.854)
EPI
HC
CNN (Prediction) [mm3]
FS (Ground Truth) [mm3]

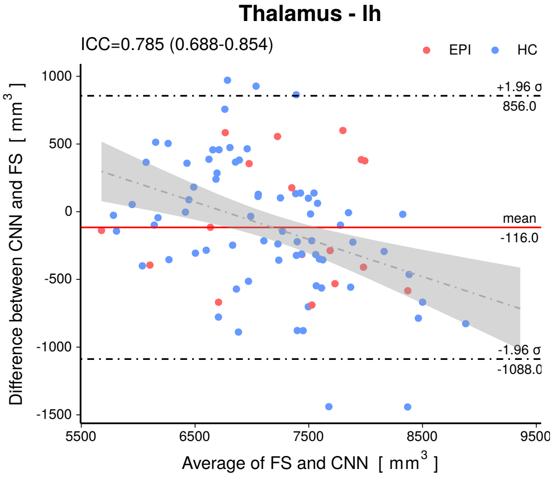

Thalamus - lh
ICC=0.785 (0.688-0.854)
EPI
HC
+1.96 σ
856.0
mean
-116.0
-1.96 σ
-1088.0
Difference between CNN and FS [mm3]
Average of FS and CNN [mm3]

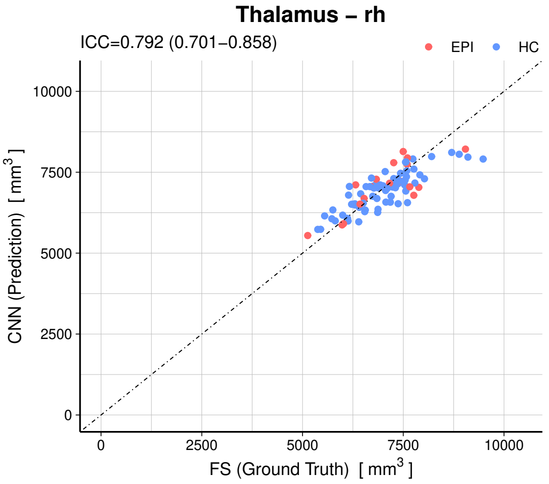

Thalamus – rh
ICC=0.792 (0.701–0.858)
EPI
HC
CNN (Prediction) [mm3]
FS (Ground Truth) [mm3]

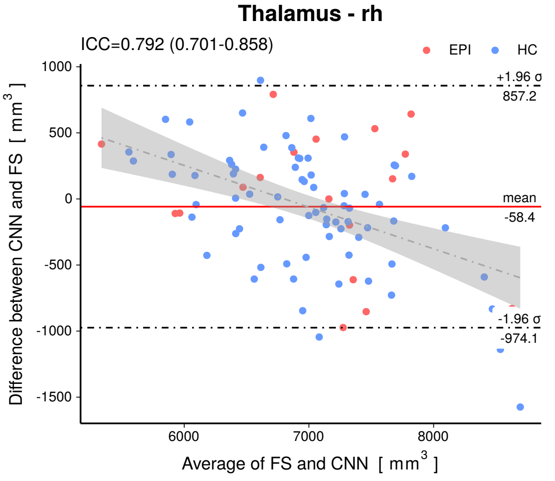

Thalamus - rh
ICC=0.792 (0.701-0.858)
EPI
HC
+1.96 σ
857.2
mean
-58.4
-1.96 σ
-974.1
Difference between CNN and FS [mm3]
Average of FS and CNN [mm3]

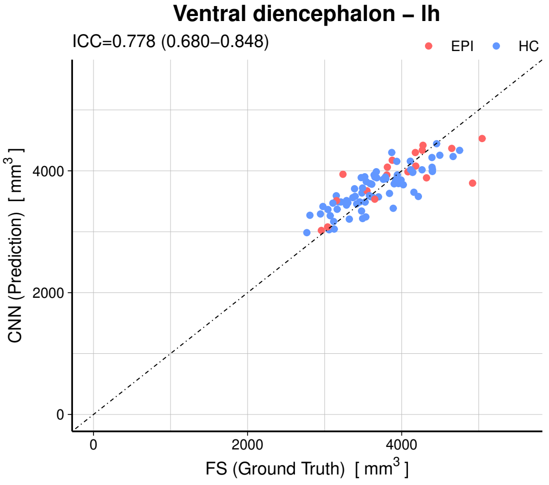

Ventral diencephalon – lh
ICC=0.778 (0.680–0.848)
EPI
HC
CNN (Prediction) [mm3]
FS (Ground Truth) [mm3]

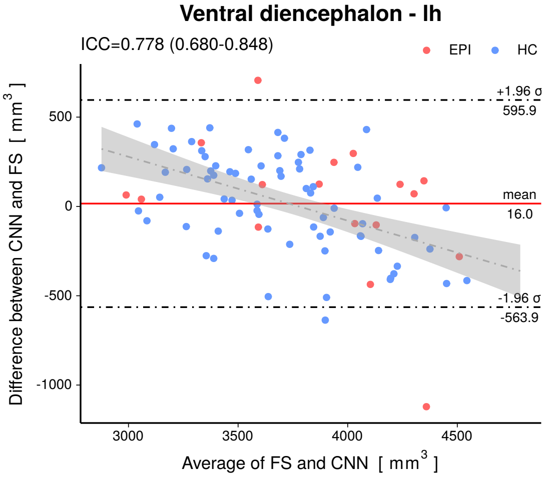

Ventral diencephalon - lh
ICC=0.778 (0.680-0.848)
EPI
HC
+1.96 σ
595.9
mean
16.0
-1.96 σ
-563.9
Difference between CNN and FS [mm3]
Average of FS and CNN [mm3]

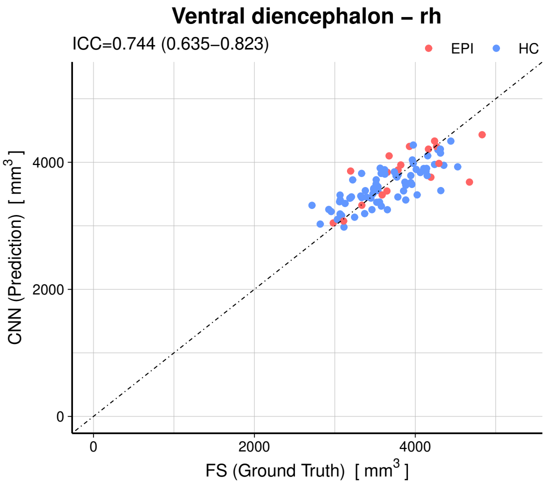
Ventral diencephalon – rh
ICC=0.744 (0.635–0.823)
EPI
HC
CNN (Prediction) [mm3]
FS (Ground Truth) [mm3]
0
2000
4000
0
2000
4000

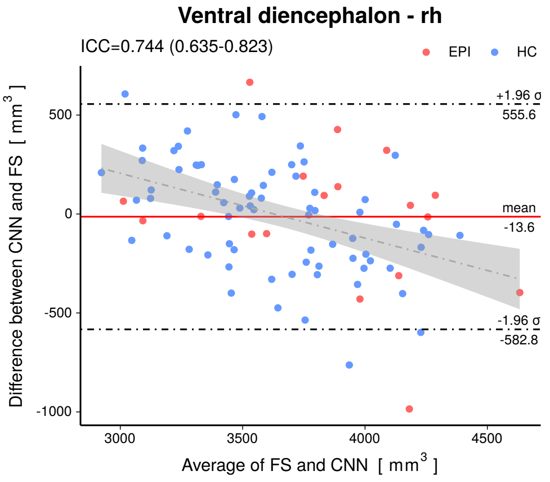
Ventral diencephalon - rh
ICC=0.744 (0.635-0.823)
EPI
HC
+1.96 σ
555.6
mean
-13.6
-1.96 σ
-582.8
Difference between CNN and FS [mm3]
Average of FS and CNN [mm3]
500
0
-500
-1000
3000
3500
4000
4500

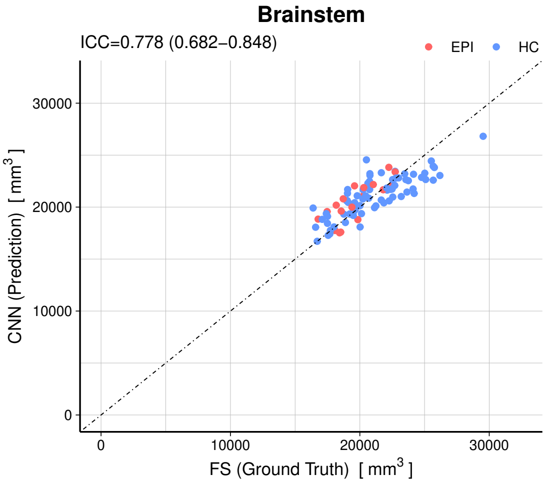
Brainstem
ICC=0.778 (0.682–0.848)
EPI
HC
CNN (Prediction) [mm3]
FS (Ground Truth) [mm3]
30000
20000
10000
0
0
10000
20000
30000

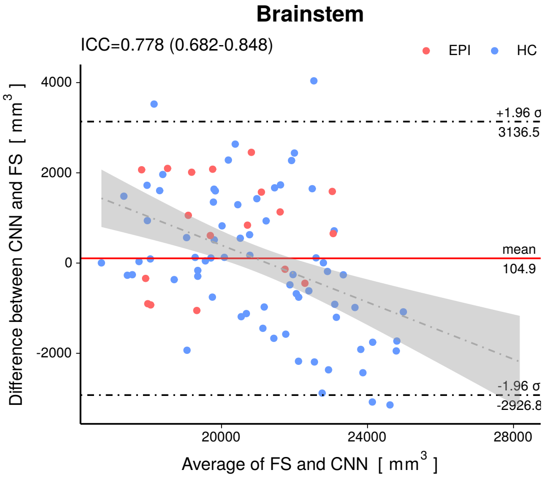
Brainstem
ICC=0.778 (0.682-0.848)
EPI
HC
+1.96 σ
3136.5
mean
104.9
-1.96 σ
-2926.8
Difference between CNN and FS [mm3]
Average of FS and CNN [mm3]
4000
2000
0
-2000
20000
24000
28000

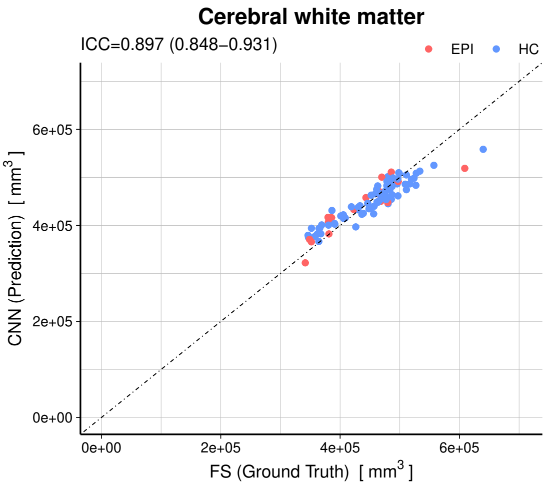
Cerebral white matter
ICC=0.897 (0.848–0.931)
EPI
HC
CNN (Prediction) [mm3]
FS (Ground Truth) [mm3]
6e+05
4e+05
2e+05
0e+00
0e+00
2e+05
4e+05
6e+05

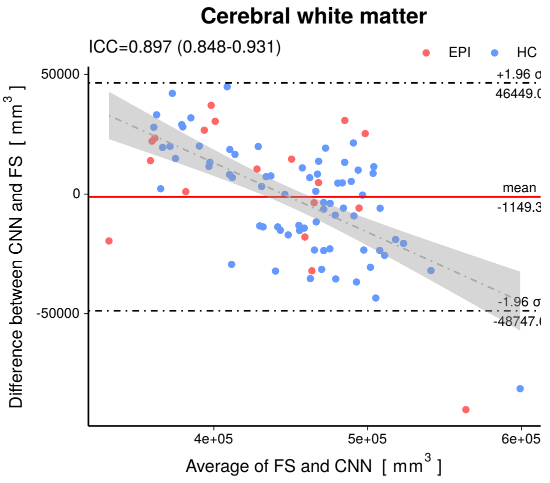
Cerebral white matter
ICC=0.897 (0.848-0.931)
EPI
HC
+1.96 σ
mean
-1.96 σ
Difference between CNN and FS [mm3]
Average of FS and CNN [mm3]
50000
0
-50000
4e+05
5e+05
6e+05

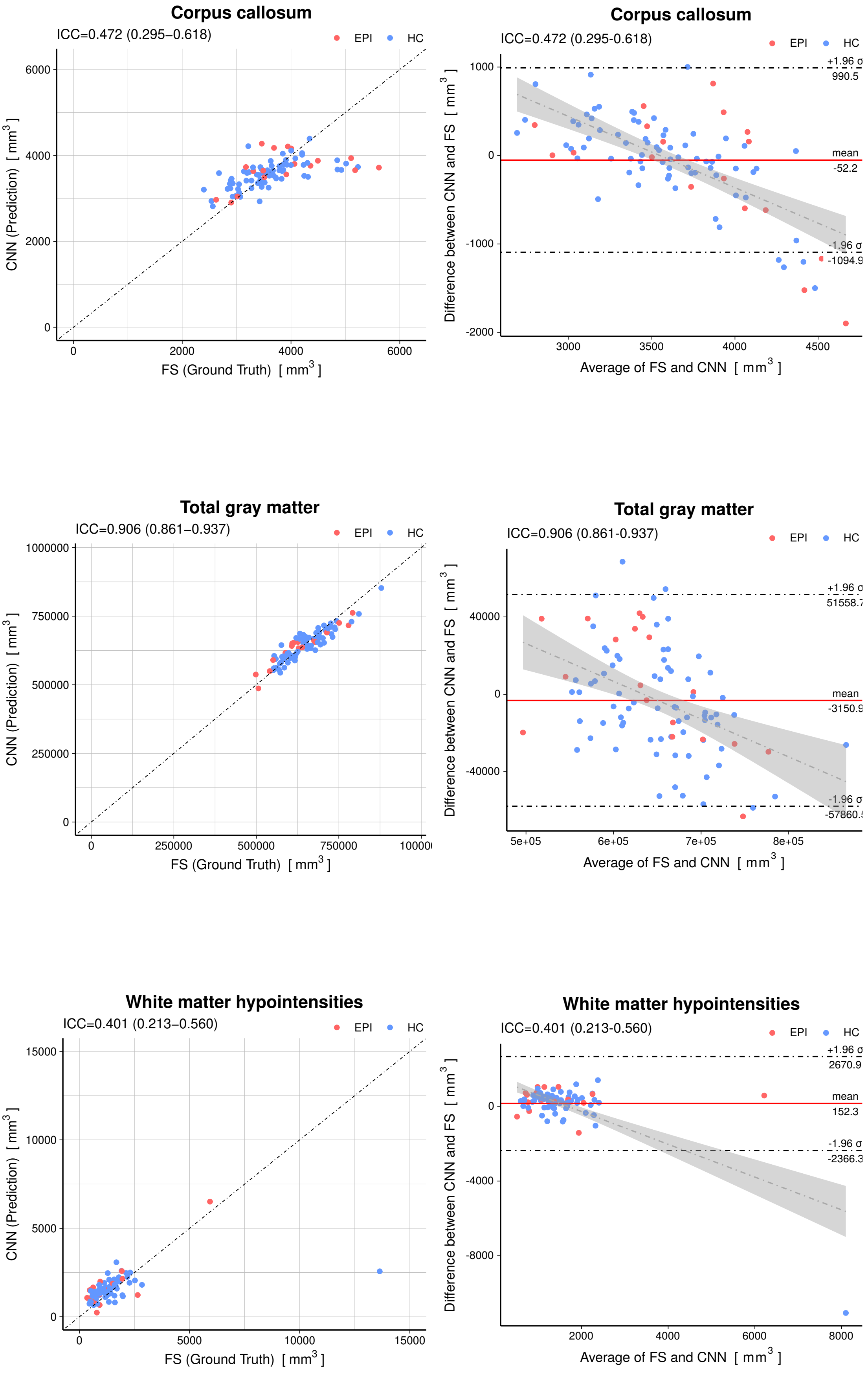
Corpus callosum
ICC=0.472 (0.295–0.618)
EPI
HC
CNN (Prediction) [mm3]
FS (Ground Truth) [mm3]
Corpus callosum
ICC=0.472 (0.295-0.618)
+1.96 σ
990.5
mean
-52.2
-1.96 σ
-1094.9
Difference between CNN and FS [mm3]
Average of FS and CNN [mm3]
Total gray matter
ICC=0.906 (0.861–0.937)
Total gray matter
ICC=0.906 (0.861-0.937)
+1.96 σ
51558.7
mean
-3150.9
-1.96 σ
-57860.5
White matter hypointensities
ICC=0.401 (0.213–0.560)
White matter hypointensities
ICC=0.401 (0.213-0.560)
+1.96 σ
2670.9
mean
152.3
-1.96 σ
-2366.3

3.2 Correlation and Bland-Altman Plots for Cortical Thickness

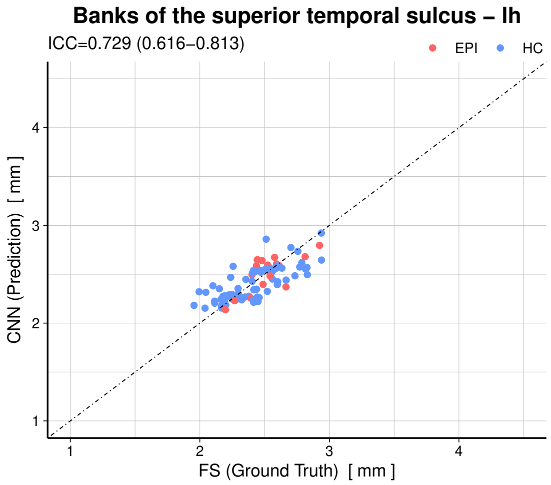

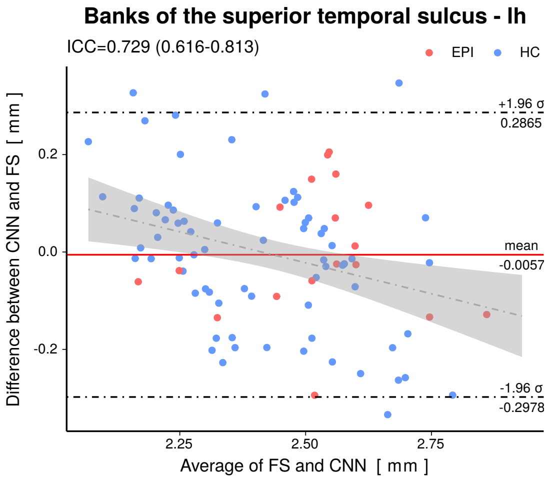

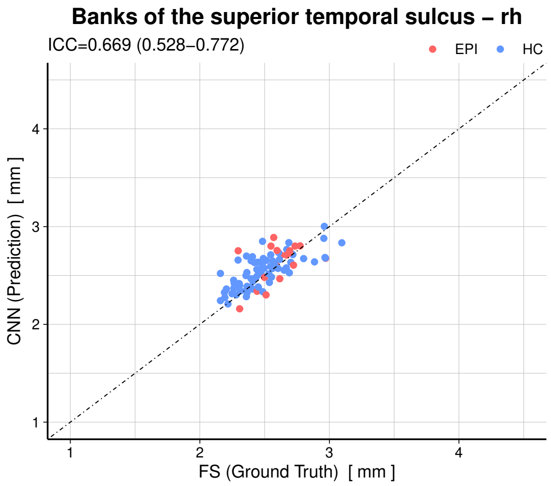

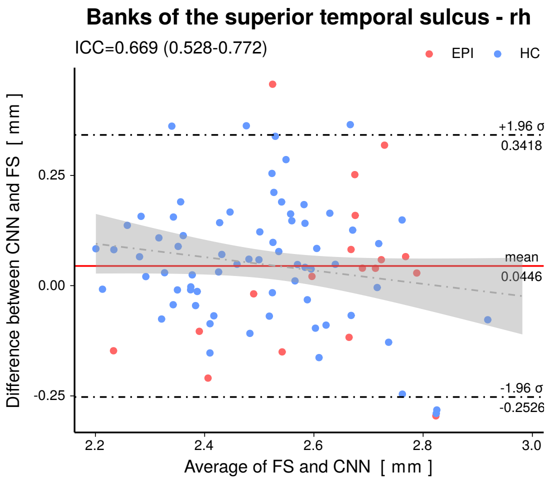

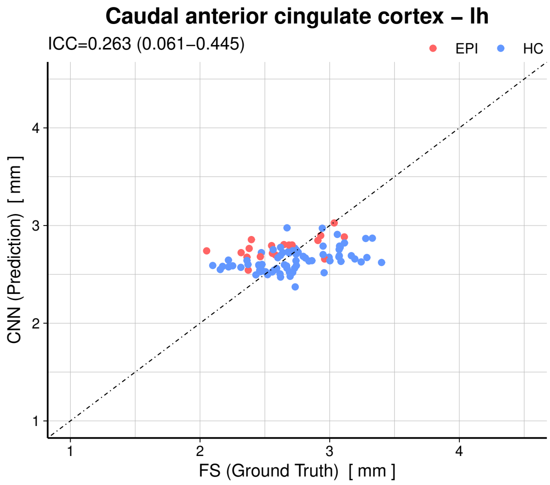

Caudal anterior cingulate cortex – lh
ICC=0.263 (0.061–0.445)
EPI
HC
CNN (Prediction) [mm]
FS (Ground Truth) [mm]

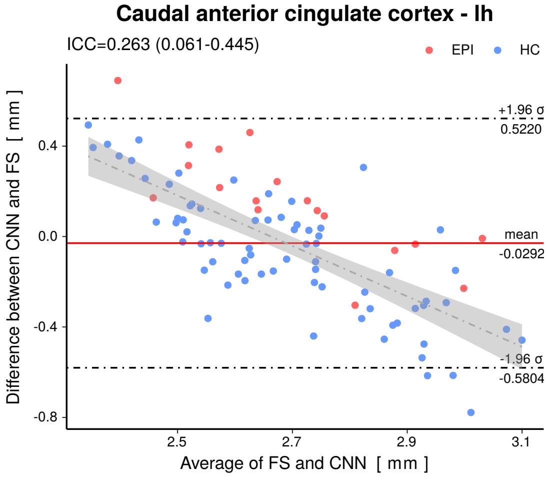

Caudal anterior cingulate cortex - lh
ICC=0.263 (0.061-0.445)
EPI
HC
+1.96 σ
0.5220
mean
-0.0292
-1.96 σ
-0.5804
Difference between CNN and FS [mm]
Average of FS and CNN [mm]

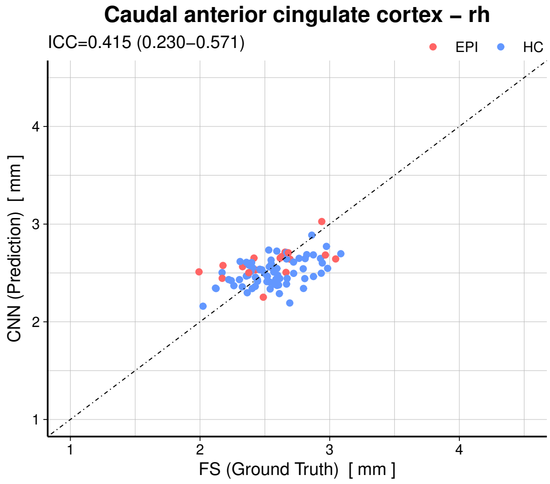

Caudal anterior cingulate cortex – rh
ICC=0.415 (0.230–0.571)
EPI
HC
CNN (Prediction) [mm]
FS (Ground Truth) [mm]

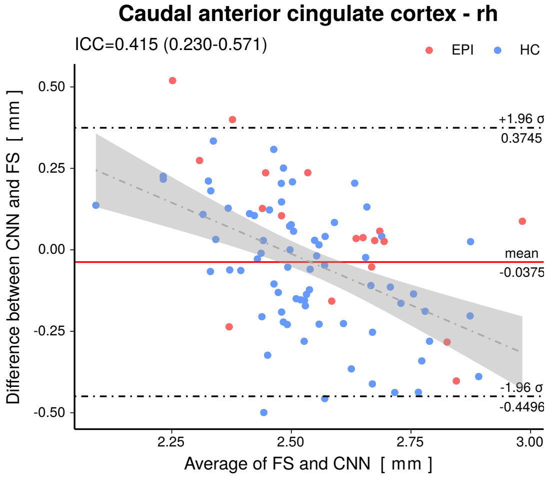

Caudal anterior cingulate cortex - rh
ICC=0.415 (0.230-0.571)
EPI
HC
+1.96 σ
0.3745
mean
-0.0375
-1.96 σ
-0.4496
Difference between CNN and FS [mm]
Average of FS and CNN [mm]

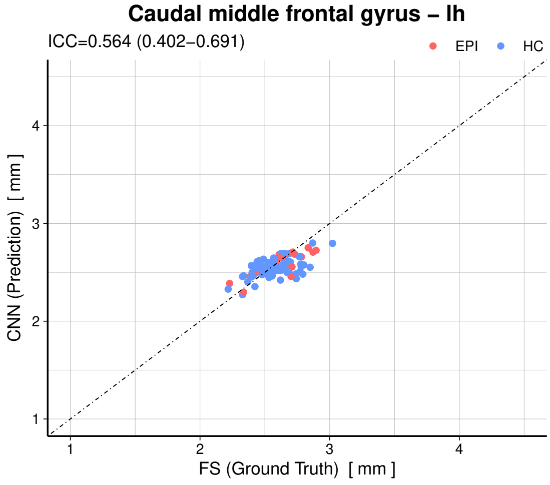

Caudal middle frontal gyrus – lh
ICC=0.564 (0.402–0.691)
EPI
HC
CNN (Prediction) [mm]
FS (Ground Truth) [mm]

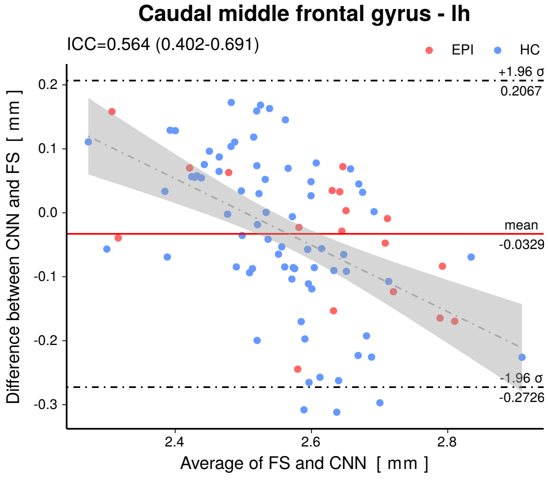

Caudal middle frontal gyrus - lh
ICC=0.564 (0.402-0.691)
EPI
HC
+1.96 σ
0.2067
mean
-0.0329
-1.96 σ
-0.2726
Difference between CNN and FS [mm]
Average of FS and CNN [mm]

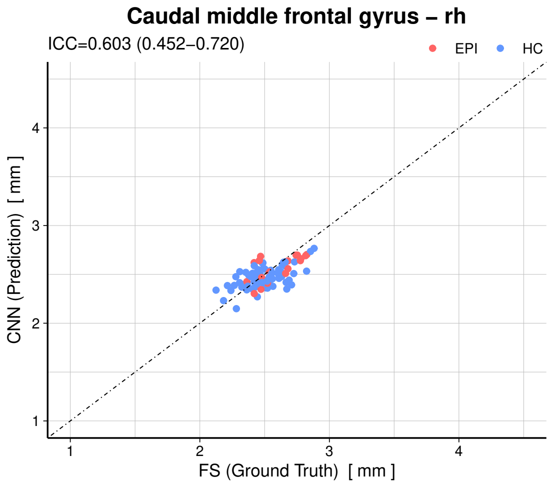
Caudal middle frontal gyrus – rh
ICC=0.603 (0.452–0.720)
EPI
HC
CNN (Prediction) [mm]
FS (Ground Truth) [mm]

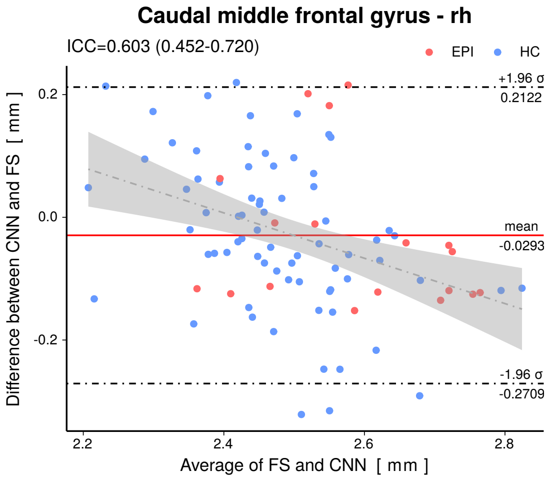
Caudal middle frontal gyrus - rh
ICC=0.603 (0.452-0.720)
EPI
HC
+1.96 σ
0.2122
mean
-0.0293
-1.96 σ
-0.2709
Difference between CNN and FS [mm]
Average of FS and CNN [mm]

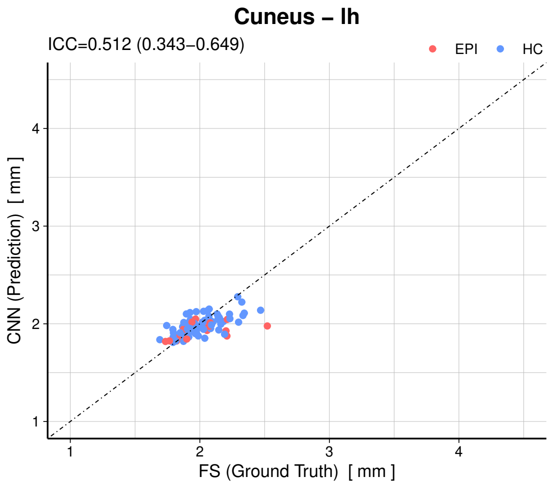
Cuneus – lh
ICC=0.512 (0.343–0.649)
EPI
HC
CNN (Prediction) [mm]
FS (Ground Truth) [mm]

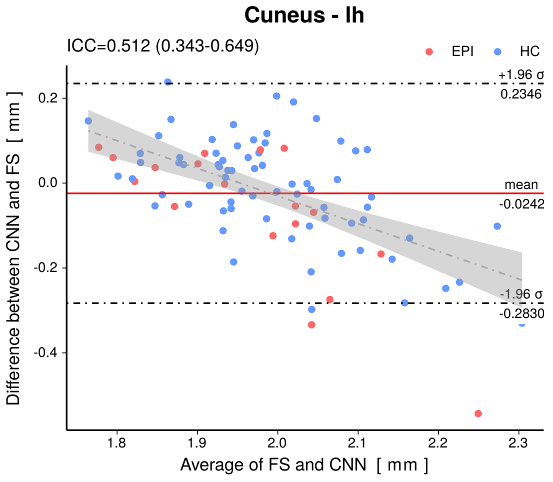
Cuneus - lh
ICC=0.512 (0.343-0.649)
EPI
HC
+1.96 σ
0.2346
mean
-0.0242
-1.96 σ
-0.2830
Difference between CNN and FS [mm]
Average of FS and CNN [mm]

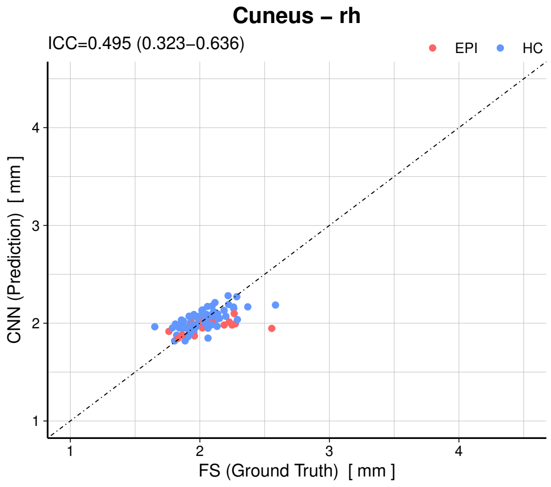
Cuneus – rh
ICC=0.495 (0.323–0.636)
EPI
HC
CNN (Prediction) [mm]
FS (Ground Truth) [mm]

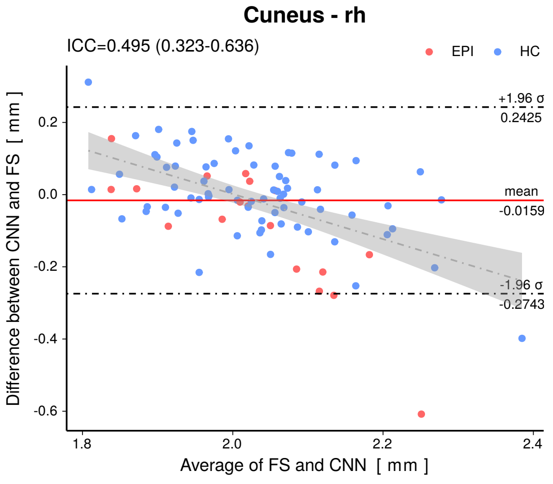
Cuneus - rh
ICC=0.495 (0.323-0.636)
EPI
HC
+1.96 σ
0.2425
mean
-0.0159
-1.96 σ
-0.2743
Difference between CNN and FS [mm]
Average of FS and CNN [mm]

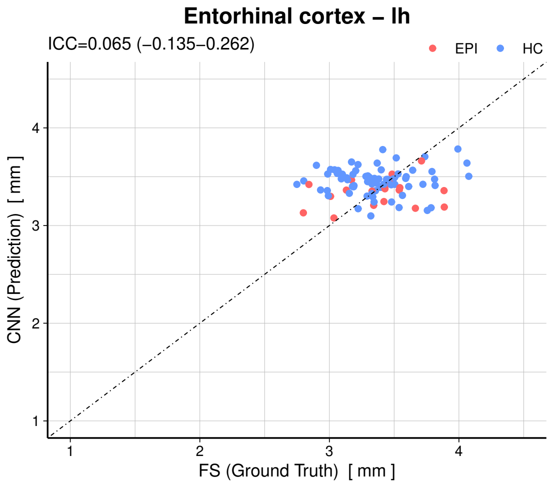
Entorhinal cortex – lh
ICC=0.065 (–0.135–0.262)
EPI
HC
CNN (Prediction) [mm]
FS (Ground Truth) [mm]
1
2
3
4

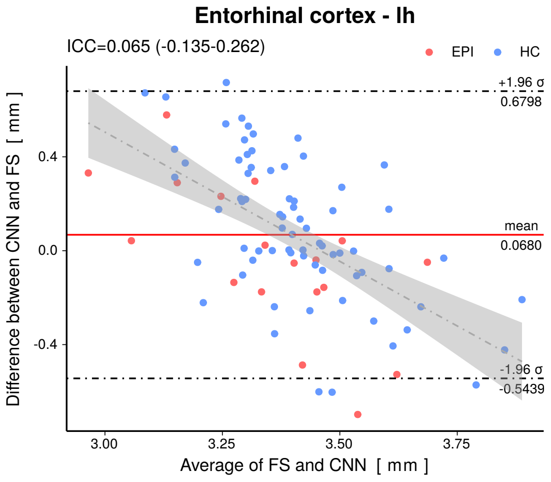
Entorhinal cortex - lh
ICC=0.065 (-0.135-0.262)
EPI
HC
+1.96 σ
0.6798
mean
0.0680
-1.96 σ
-0.5439
0.4
0.0
-0.4
3.00
3.25
3.50
3.75
Difference between CNN and FS [mm]
Average of FS and CNN [mm]

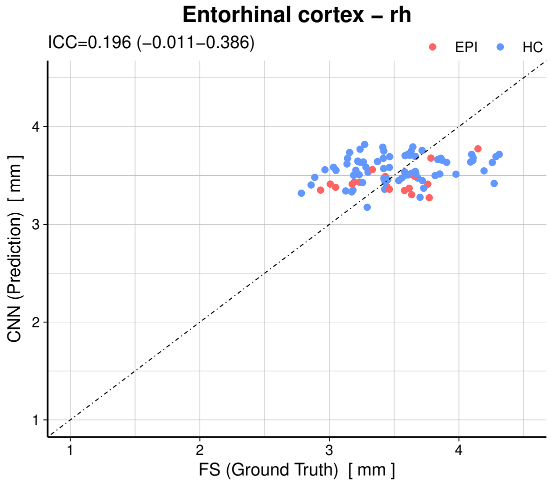
Entorhinal cortex – rh
ICC=0.196 (–0.011–0.386)
EPI
HC
CNN (Prediction) [mm]
FS (Ground Truth) [mm]
1
2
3
4

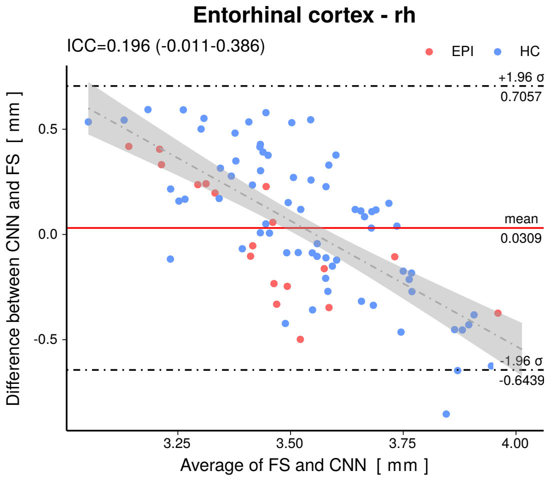
Entorhinal cortex - rh
ICC=0.196 (-0.011-0.386)
EPI
HC
+1.96 σ
0.7057
mean
0.0309
-1.96 σ
-0.6439
0.5
0.0
-0.5
3.25
3.50
3.75
4.00
Difference between CNN and FS [mm]
Average of FS and CNN [mm]

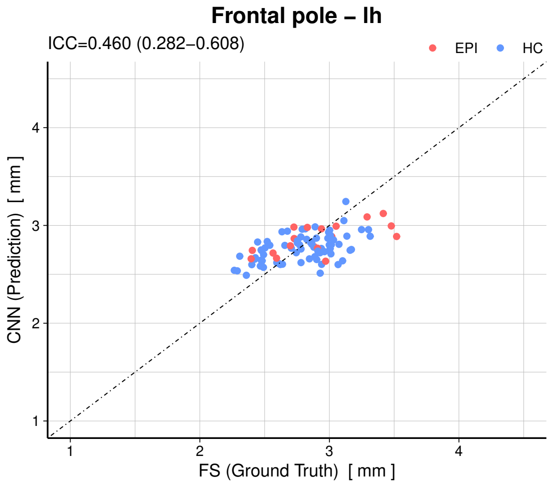
Frontal pole – lh
ICC=0.460 (0.282–0.608)
EPI
HC
CNN (Prediction) [mm]
FS (Ground Truth) [mm]
1
2
3
4

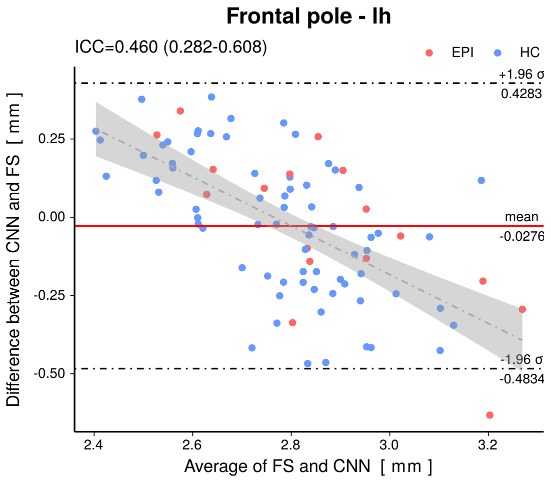
Frontal pole - lh
ICC=0.460 (0.282-0.608)
EPI
HC
+1.96 σ
0.4283
mean
-0.0276
-1.96 σ
-0.4834
0.25
0.00
-0.25
-0.50
2.4
2.6
2.8
3.0
3.2
Difference between CNN and FS [mm]
Average of FS and CNN [mm]

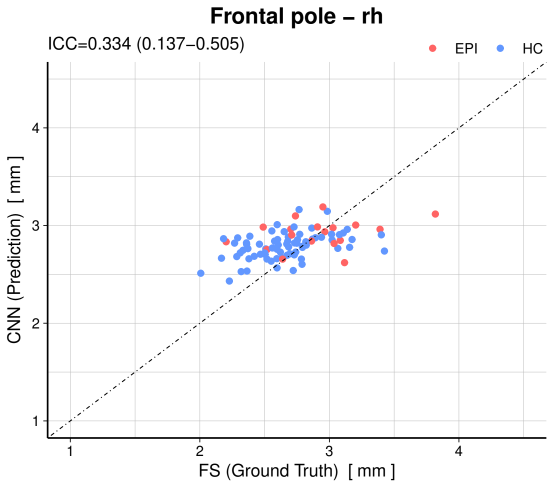

Frontal pole – rh
ICC=0.334 (0.137–0.505)
EPI
HC
CNN (Prediction) [mm]
FS (Ground Truth) [mm]

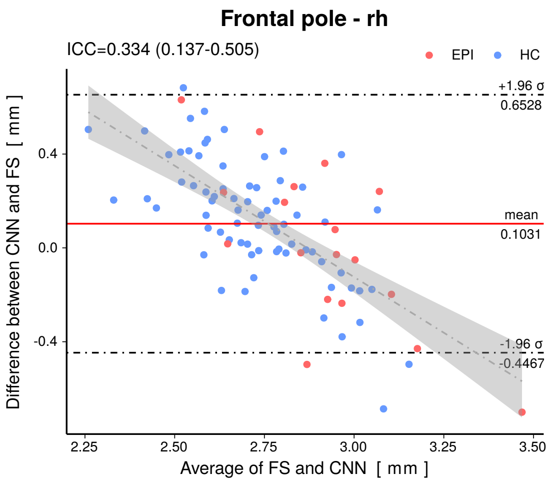

Frontal pole - rh
ICC=0.334 (0.137-0.505)
EPI
HC
+1.96 σ
0.6528
mean
0.1031
-1.96 σ
-0.4467
Difference between CNN and FS [mm]
Average of FS and CNN [mm]

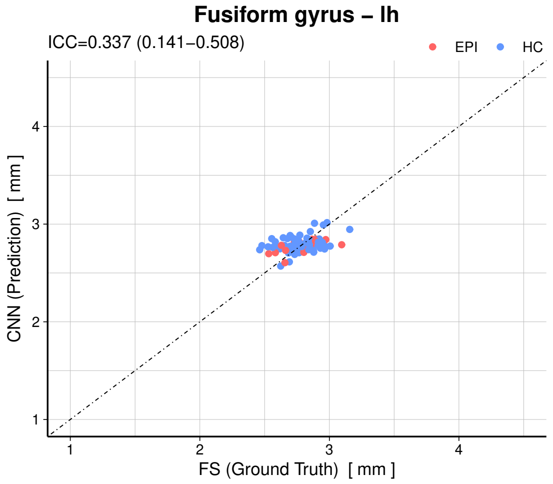

Fusiform gyrus – lh
ICC=0.337 (0.141–0.508)
EPI
HC
CNN (Prediction) [mm]
FS (Ground Truth) [mm]

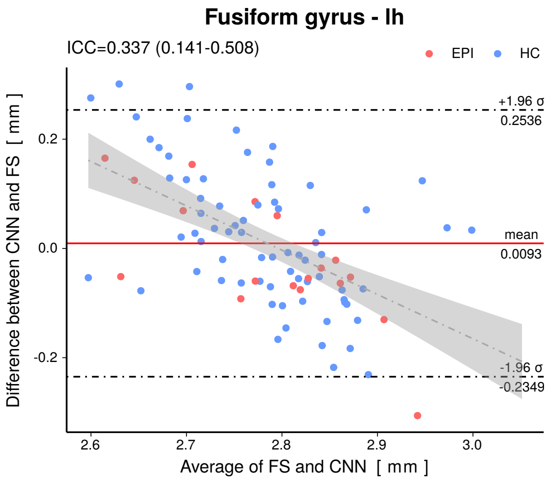

Fusiform gyrus - lh
ICC=0.337 (0.141-0.508)
EPI
HC
+1.96 σ
0.2536
mean
0.0093
-1.96 σ
-0.2349
Difference between CNN and FS [mm]
Average of FS and CNN [mm]

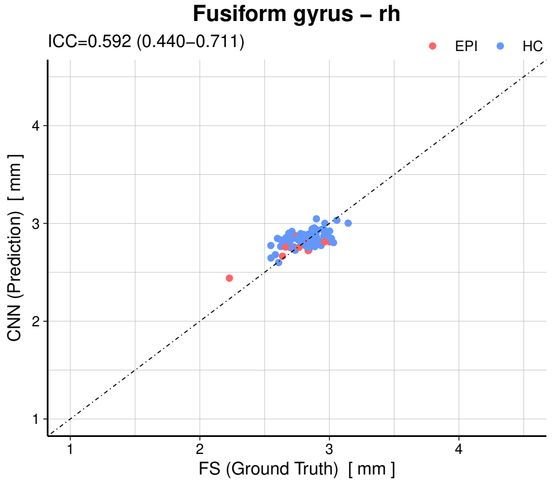

Fusiform gyrus – rh
ICC=0.592 (0.440–0.711)
EPI
HC
CNN (Prediction) [mm]
FS (Ground Truth) [mm]

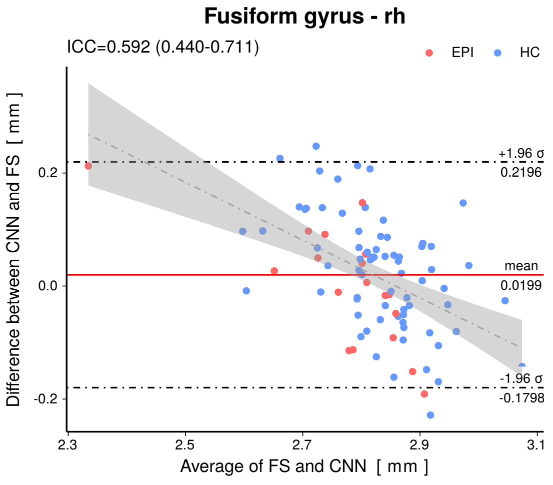

Fusiform gyrus - rh
ICC=0.592 (0.440-0.711)
EPI
HC
+1.96 σ
0.2196
mean
0.0199
-1.96 σ
-0.1798
Difference between CNN and FS [mm]
Average of FS and CNN [mm]

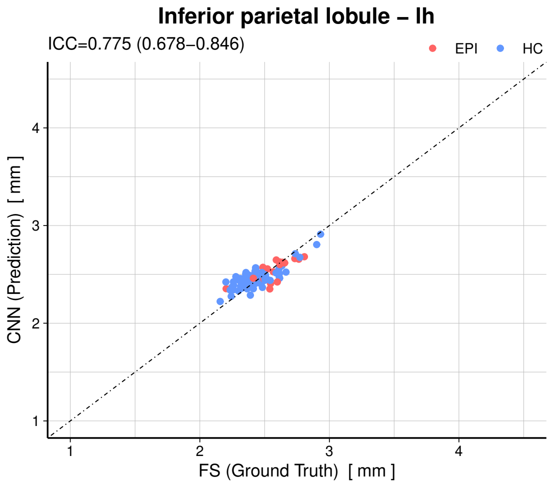
Inferior parietal lobule – lh
ICC=0.775 (0.678–0.846)
EPI
HC
CNN (Prediction) [mm]
FS (Ground Truth) [mm]

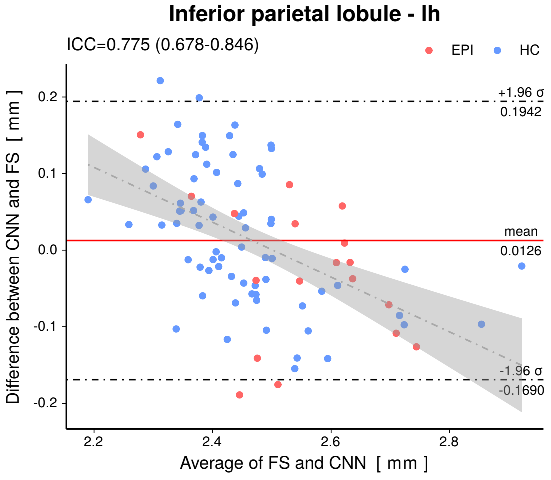
Inferior parietal lobule - lh
ICC=0.775 (0.678-0.846)
EPI
HC
+1.96 σ
0.1942
mean
0.0126
-1.96 σ
-0.1690
Difference between CNN and FS [mm]
Average of FS and CNN [mm]

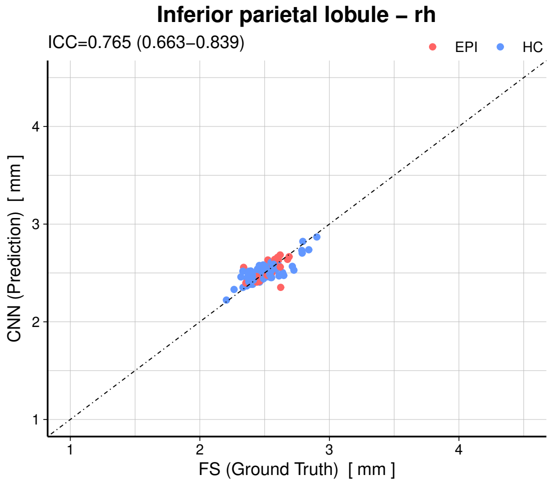
Inferior parietal lobule – rh
ICC=0.765 (0.663–0.839)
EPI
HC
CNN (Prediction) [mm]
FS (Ground Truth) [mm]

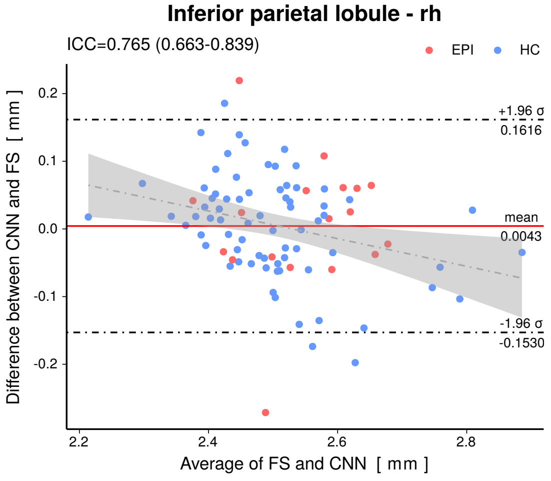
Inferior parietal lobule - rh
ICC=0.765 (0.663-0.839)
EPI
HC
+1.96 σ
0.1616
mean
0.0043
-1.96 σ
-0.1530
Difference between CNN and FS [mm]
Average of FS and CNN [mm]

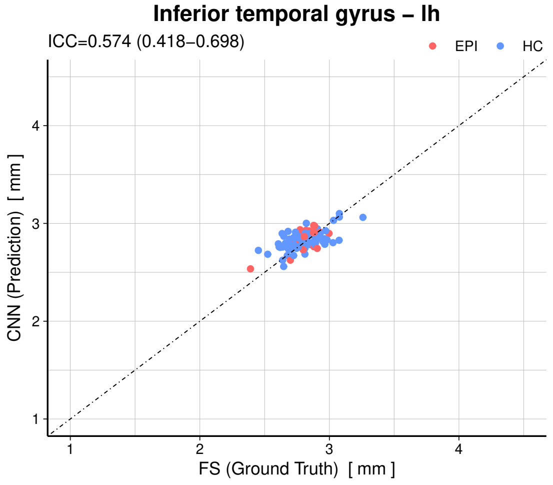
Inferior temporal gyrus – lh
ICC=0.574 (0.418–0.698)
EPI
HC
CNN (Prediction) [mm]
FS (Ground Truth) [mm]

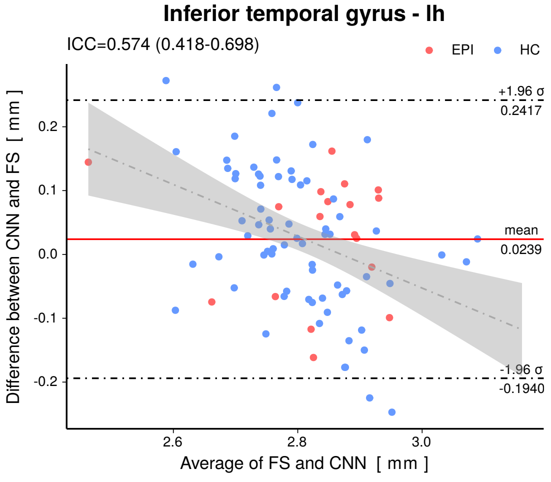
Inferior temporal gyrus - lh
ICC=0.574 (0.418-0.698)
EPI
HC
+1.96 σ
0.2417
mean
0.0239
-1.96 σ
-0.1940
Difference between CNN and FS [mm]
Average of FS and CNN [mm]

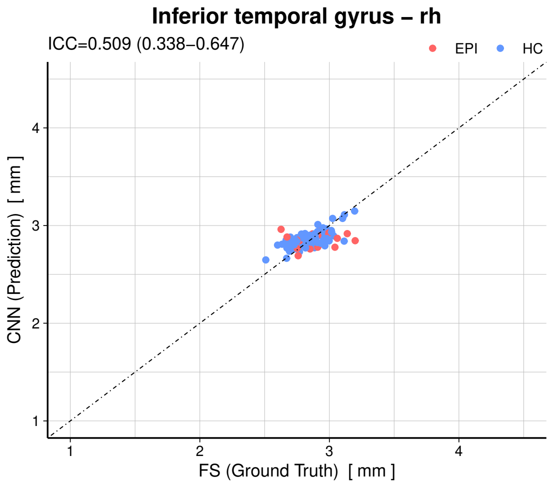
Inferior temporal gyrus – rh
ICC=0.509 (0.338–0.647)
EPI
HC
CNN (Prediction) [mm]
FS (Ground Truth) [mm]

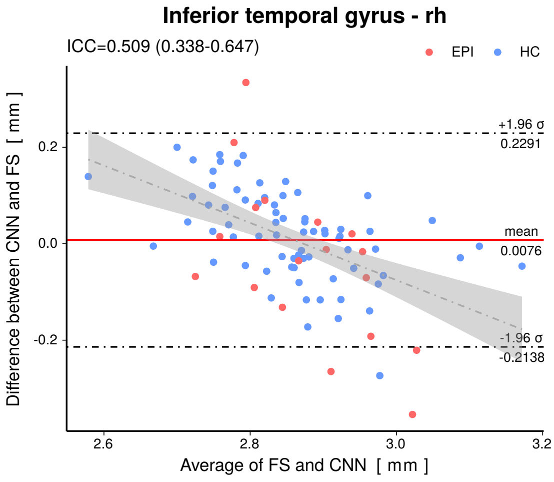
Inferior temporal gyrus - rh
ICC=0.509 (0.338-0.647)
EPI
HC
+1.96 σ
0.2291
mean
0.0076
-1.96 σ
-0.2138
Difference between CNN and FS [mm]
Average of FS and CNN [mm]

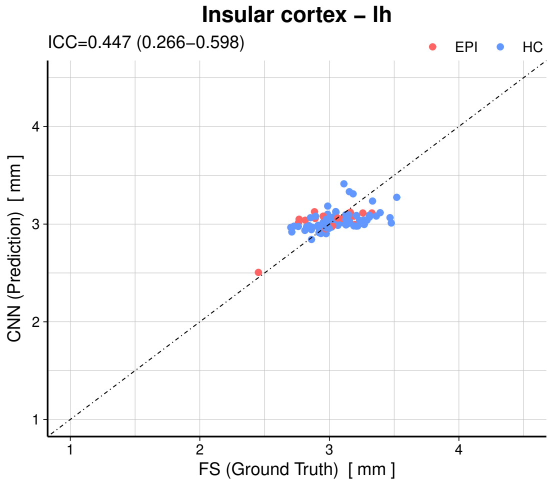
Insular cortex – lh
ICC=0.447 (0.266–0.598)
EPI
HC
CNN (Prediction) [mm]
FS (Ground Truth) [mm]

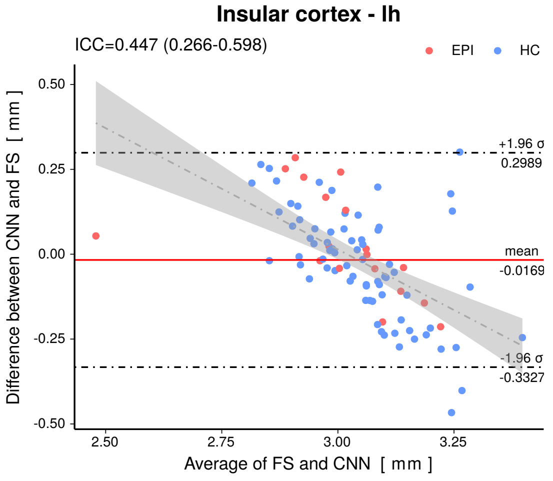
Insular cortex - lh
ICC=0.447 (0.266-0.598)
EPI
HC
+1.96 σ
0.2989
mean
-0.0169
-1.96 σ
-0.3327
Difference between CNN and FS [mm]
Average of FS and CNN [mm]

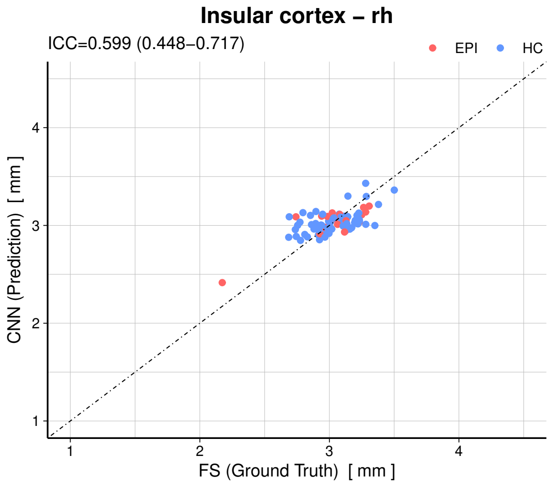
Insular cortex – rh
ICC=0.599 (0.448–0.717)
EPI
HC
CNN (Prediction) [mm]
FS (Ground Truth) [mm]

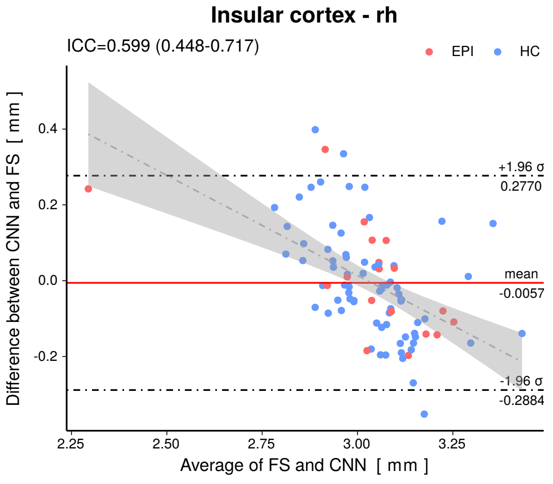
Insular cortex - rh
ICC=0.599 (0.448-0.717)
EPI
HC
+1.96 σ
0.2770
mean
-0.0057
-1.96 σ
-0.2884
Difference between CNN and FS [mm]
Average of FS and CNN [mm]

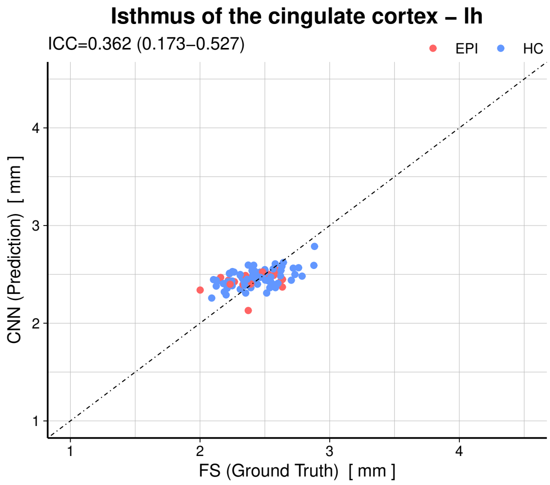
Isthmus of the cingulate cortex – lh
ICC=0.362 (0.173–0.527)
EPI
HC
CNN (Prediction) [mm]
FS (Ground Truth) [mm]

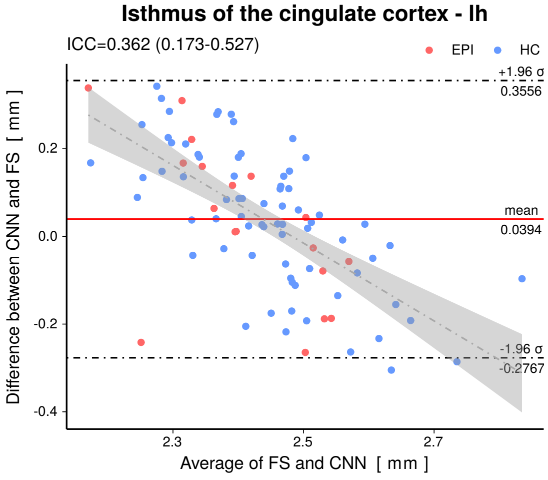
Isthmus of the cingulate cortex - lh
ICC=0.362 (0.173-0.527)
EPI
HC
+1.96 σ
0.3556
mean
0.0394
-1.96 σ
-0.2767
Difference between CNN and FS [mm]
Average of FS and CNN [mm]

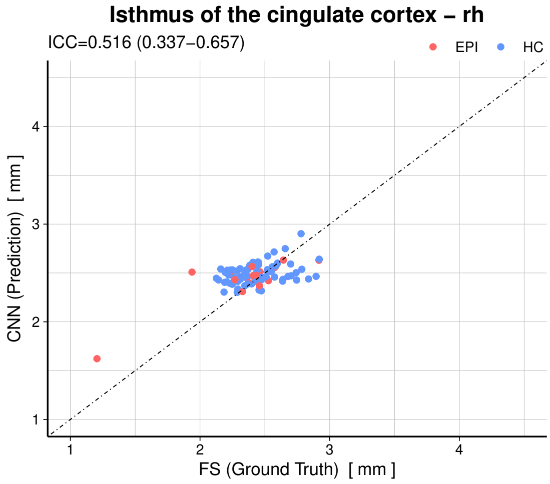
Isthmus of the cingulate cortex – rh
ICC=0.516 (0.337–0.657)
EPI
HC
CNN (Prediction) [mm]
FS (Ground Truth) [mm]

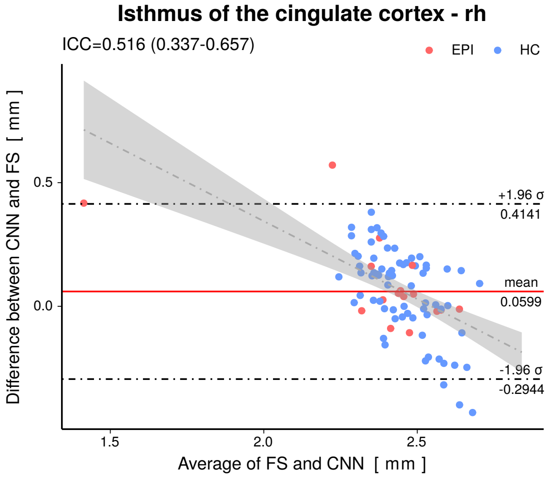
Isthmus of the cingulate cortex - rh
ICC=0.516 (0.337-0.657)
EPI
HC
+1.96 σ
0.4141
mean
0.0599
-1.96 σ
-0.2944
Difference between CNN and FS [mm]
Average of FS and CNN [mm]

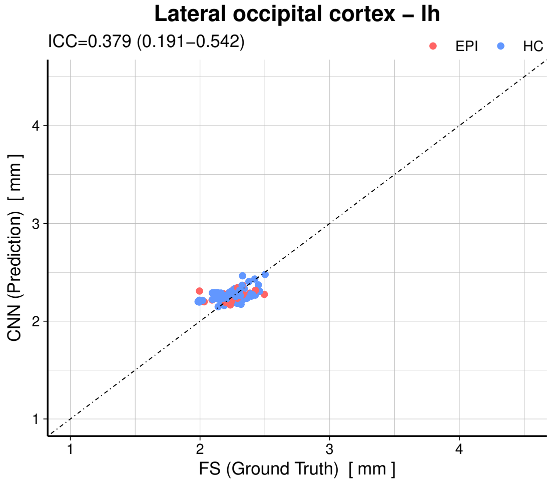
Lateral occipital cortex – lh
ICC=0.379 (0.191–0.542)
EPI
HC
CNN (Prediction) [mm]
FS (Ground Truth) [mm]

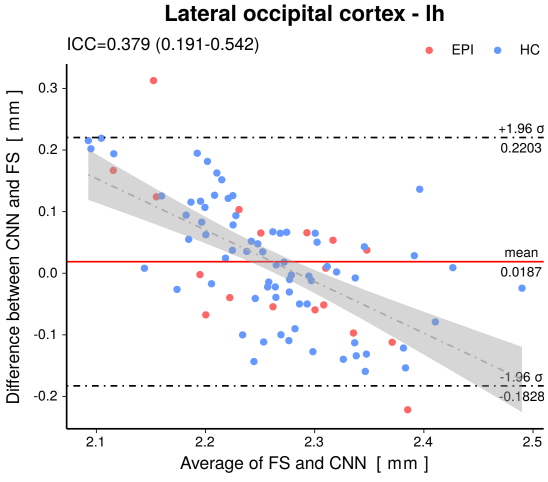
Lateral occipital cortex - lh
ICC=0.379 (0.191-0.542)
EPI
HC
+1.96 σ
0.2203
mean
0.0187
-1.96 σ
-0.1828
Difference between CNN and FS [mm]
Average of FS and CNN [mm]

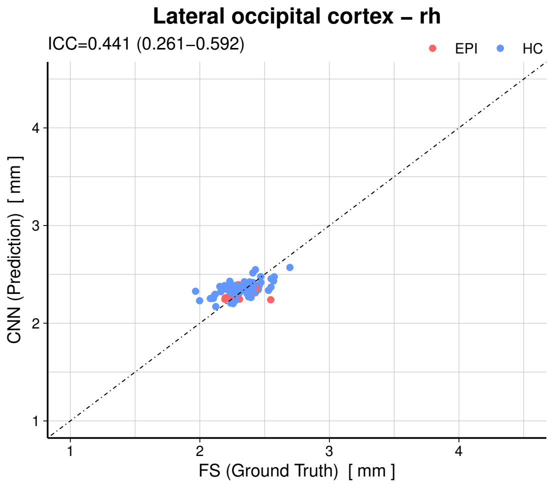
Lateral occipital cortex – rh
ICC=0.441 (0.261–0.592)
EPI
HC
CNN (Prediction) [mm]
FS (Ground Truth) [mm]

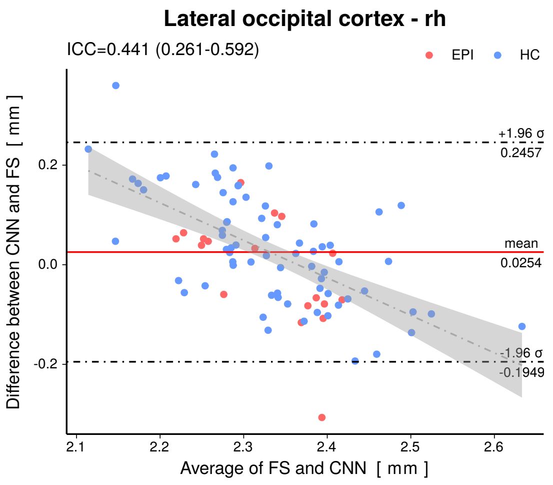
Lateral occipital cortex - rh
ICC=0.441 (0.261-0.592)
EPI
HC
+1.96 σ
0.2457
mean
0.0254
-1.96 σ
-0.1949
Difference between CNN and FS [mm]
Average of FS and CNN [mm]

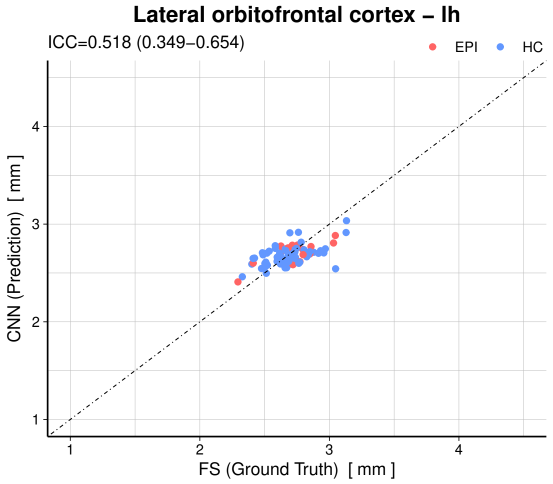
Lateral orbitofrontal cortex – lh
ICC=0.518 (0.349–0.654)
EPI
HC
CNN (Prediction) [mm]
FS (Ground Truth) [mm]

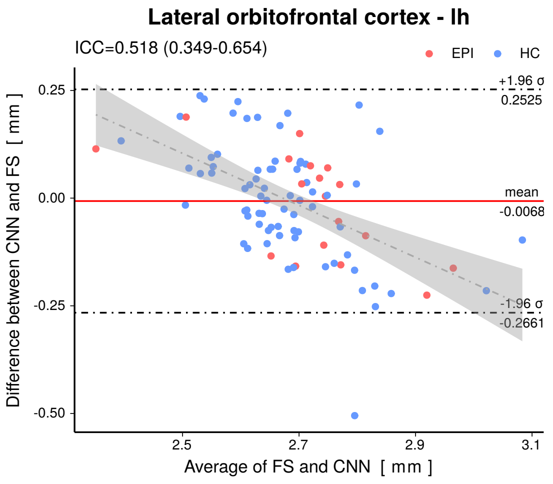
Lateral orbitofrontal cortex - lh
ICC=0.518 (0.349-0.654)
EPI
HC
+1.96 σ
0.2525
mean
-0.0068
-1.96 σ
-0.2661
Difference between CNN and FS [mm]
Average of FS and CNN [mm]

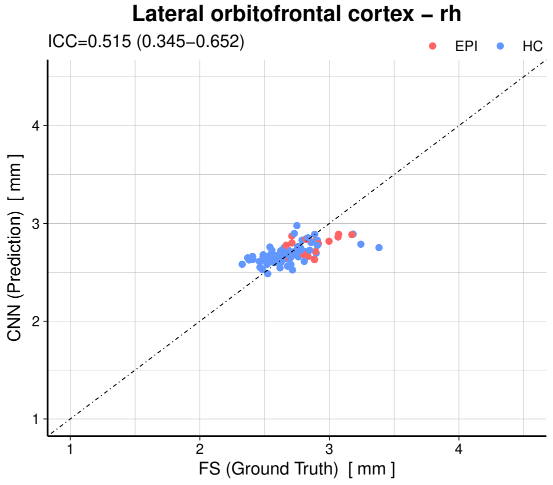
Lateral orbitofrontal cortex – rh
ICC=0.515 (0.345–0.652)
EPI
HC
CNN (Prediction) [mm]
FS (Ground Truth) [mm]

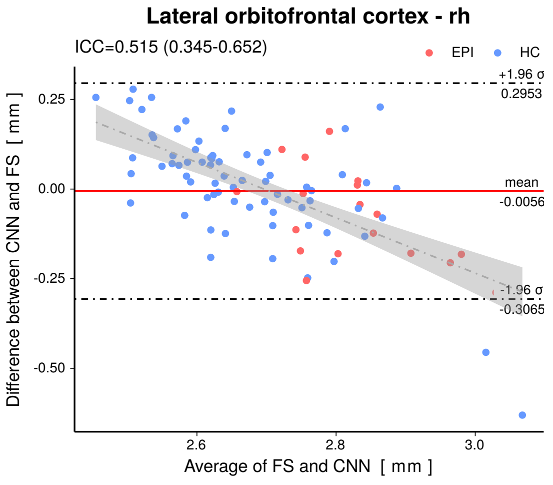
Lateral orbitofrontal cortex - rh
ICC=0.515 (0.345-0.652)
EPI
HC
+1.96 σ
0.2953
mean
-0.0056
-1.96 σ
-0.3065
Difference between CNN and FS [mm]
Average of FS and CNN [mm]

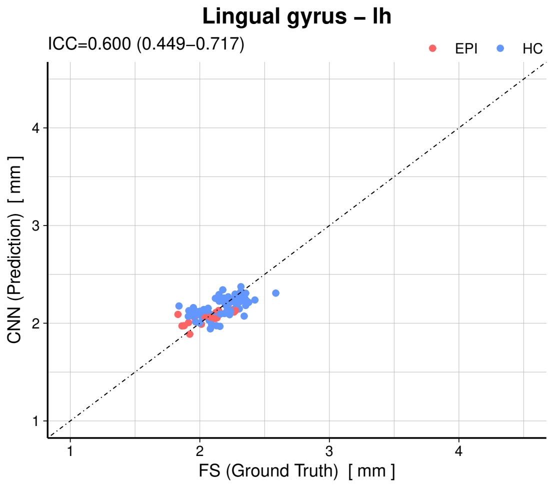
Lingual gyrus – lh
ICC=0.600 (0.449–0.717)
EPI
HC
CNN (Prediction) [mm]
FS (Ground Truth) [mm]

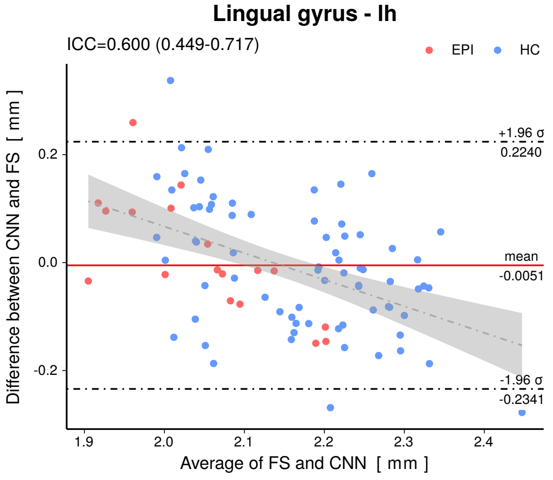
Lingual gyrus - lh
ICC=0.600 (0.449-0.717)
EPI
HC
+1.96 σ
0.2240
mean
-0.0051
-1.96 σ
-0.2341
Difference between CNN and FS [mm]
Average of FS and CNN [mm]

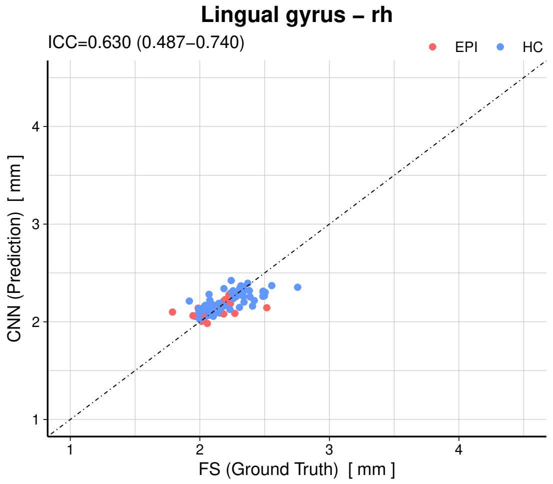
Lingual gyrus – rh
ICC=0.630 (0.487–0.740)
EPI
HC
CNN (Prediction) [mm]
FS (Ground Truth) [mm]

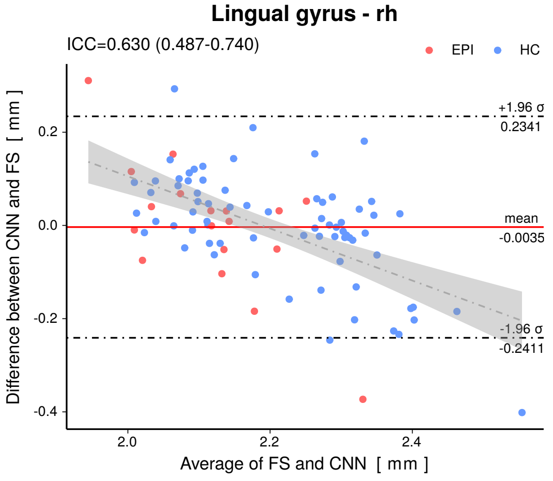
Lingual gyrus - rh
ICC=0.630 (0.487-0.740)
EPI
HC
+1.96 σ
0.2341
mean
-0.0035
-1.96 σ
-0.2411
Difference between CNN and FS [mm]
Average of FS and CNN [mm]

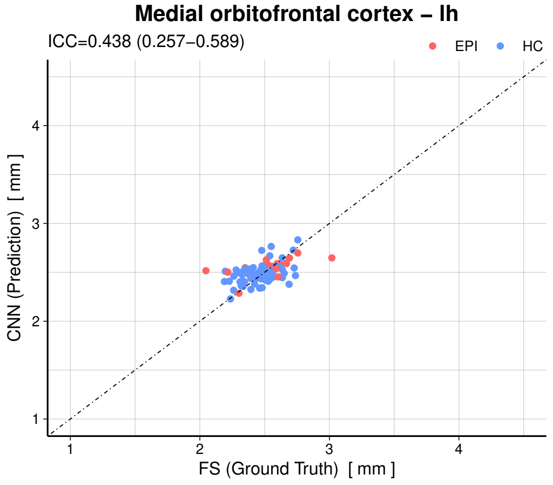
Medial orbitofrontal cortex – lh
ICC=0.438 (0.257–0.589)
EPI
HC
CNN (Prediction) [mm]
FS (Ground Truth) [mm]

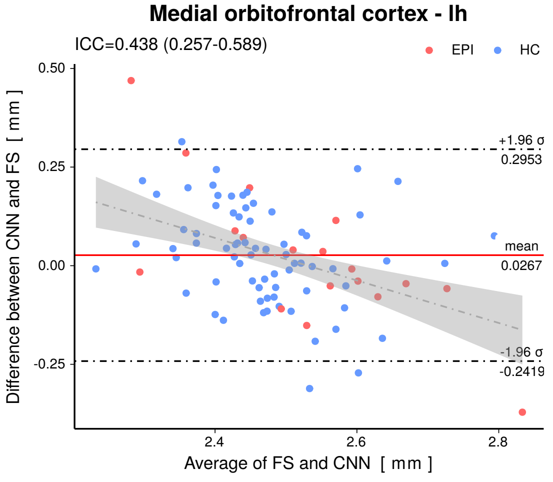
Medial orbitofrontal cortex - lh
ICC=0.438 (0.257-0.589)
EPI
HC
+1.96 σ
0.2953
mean
0.0267
-1.96 σ
-0.2419
Difference between CNN and FS [mm]
Average of FS and CNN [mm]

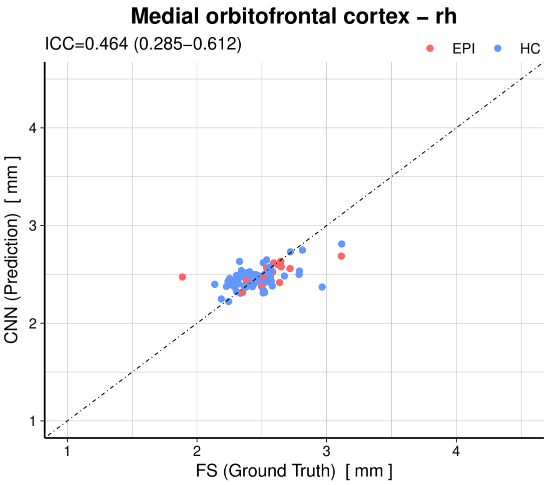
Medial orbitofrontal cortex – rh
ICC=0.464 (0.285–0.612)
EPI
HC
CNN (Prediction) [mm]
FS (Ground Truth) [mm]

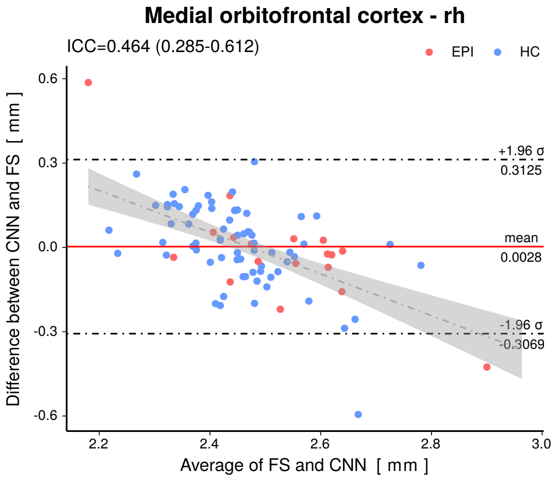
Medial orbitofrontal cortex - rh
ICC=0.464 (0.285-0.612)
EPI
HC
+1.96 σ
0.3125
mean
0.0028
-1.96 σ
-0.3069
Difference between CNN and FS [mm]
Average of FS and CNN [mm]

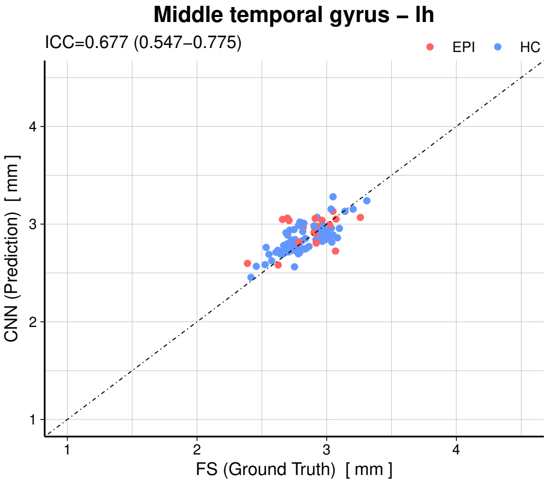
Middle temporal gyrus – lh
ICC=0.677 (0.547–0.775)
EPI
HC
CNN (Prediction) [mm]
FS (Ground Truth) [mm]

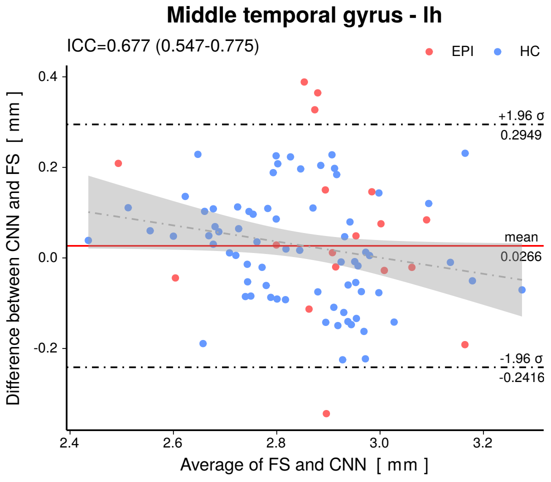
Middle temporal gyrus - lh
ICC=0.677 (0.547-0.775)
EPI
HC
+1.96 σ
0.2949
mean
0.0266
-1.96 σ
-0.2416
Difference between CNN and FS [mm]
Average of FS and CNN [mm]

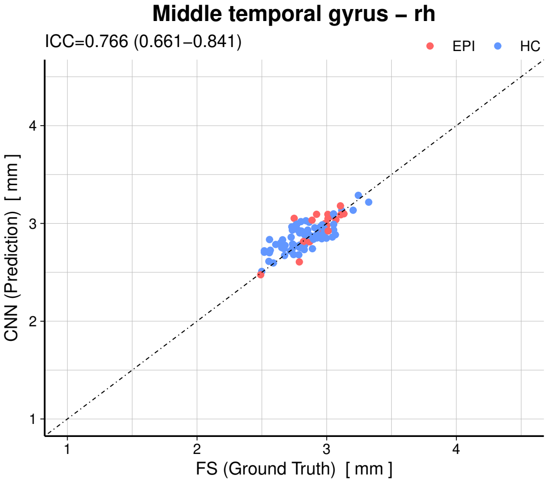
Middle temporal gyrus – rh
ICC=0.766 (0.661–0.841)
EPI
HC
CNN (Prediction) [mm]
FS (Ground Truth) [mm]

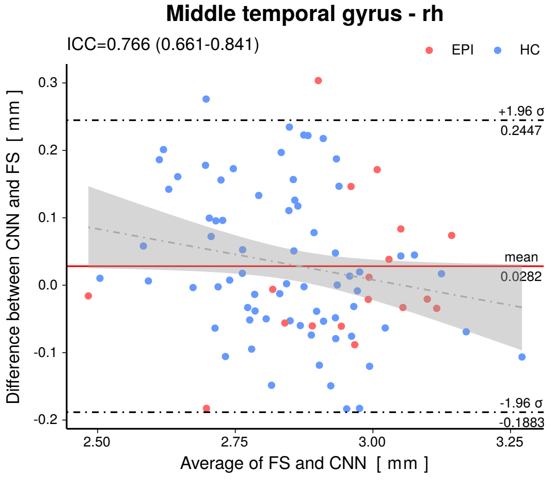
Middle temporal gyrus - rh
ICC=0.766 (0.661-0.841)
EPI
HC
+1.96 σ
0.2447
mean
0.0282
-1.96 σ
-0.1883
Difference between CNN and FS [mm]
Average of FS and CNN [mm]

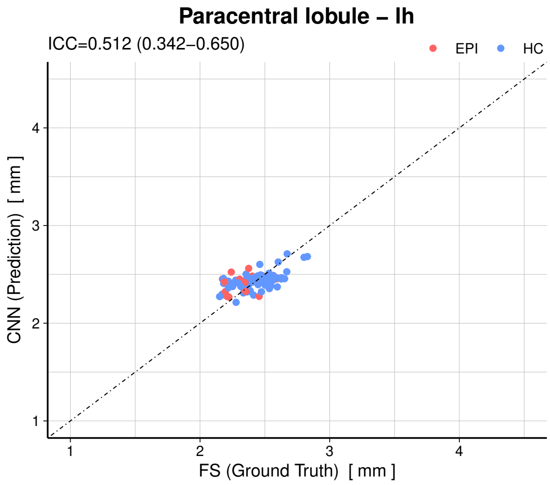
Paracentral lobule – lh
ICC=0.512 (0.342–0.650)
EPI
HC
CNN (Prediction) [mm]
FS (Ground Truth) [mm]

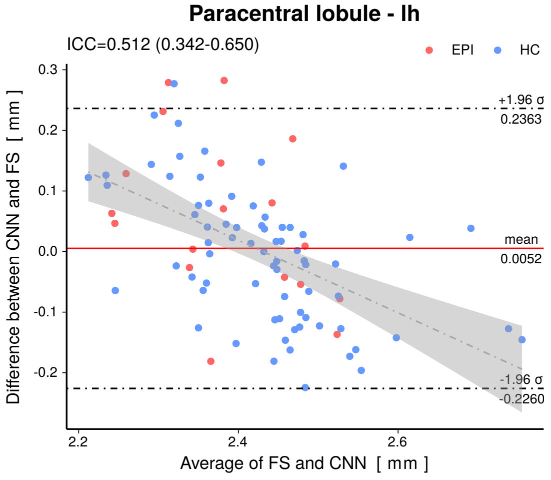
Paracentral lobule - lh
ICC=0.512 (0.342-0.650)
EPI
HC
+1.96 σ
0.2363
mean
0.0052
-1.96 σ
-0.2260
Difference between CNN and FS [mm]
Average of FS and CNN [mm]

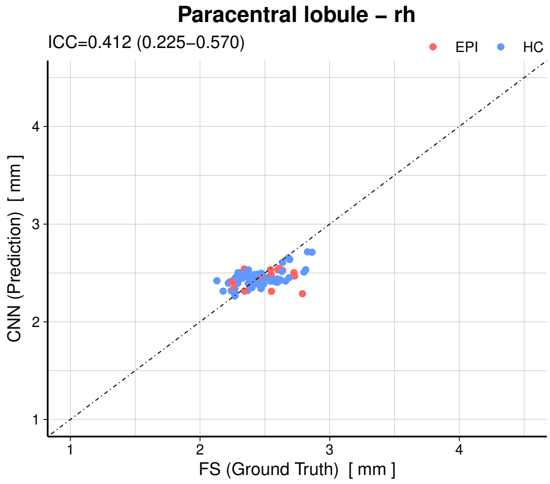
Paracentral lobule – rh
ICC=0.412 (0.225–0.570)
EPI
HC
CNN (Prediction) [mm]
FS (Ground Truth) [mm]

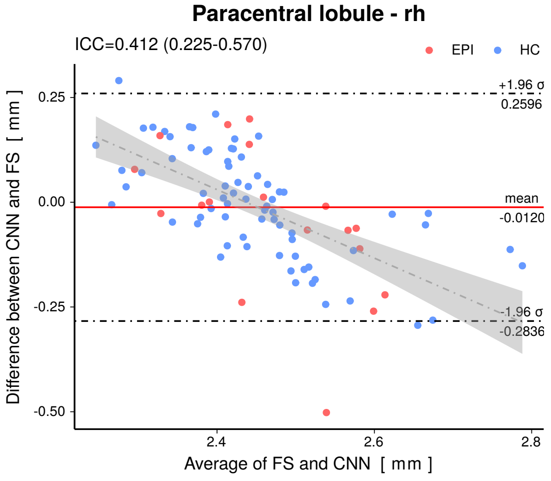
Paracentral lobule - rh
ICC=0.412 (0.225-0.570)
EPI
HC
+1.96 σ
0.2596
mean
-0.0120
-1.96 σ
-0.2836
Difference between CNN and FS [mm]
Average of FS and CNN [mm]

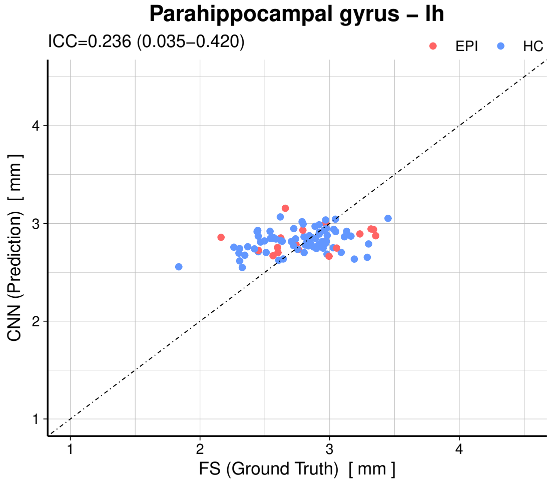
Parahippocampal gyrus – lh
ICC=0.236 (0.035–0.420)
EPI
HC
CNN (Prediction) [mm]
FS (Ground Truth) [mm]

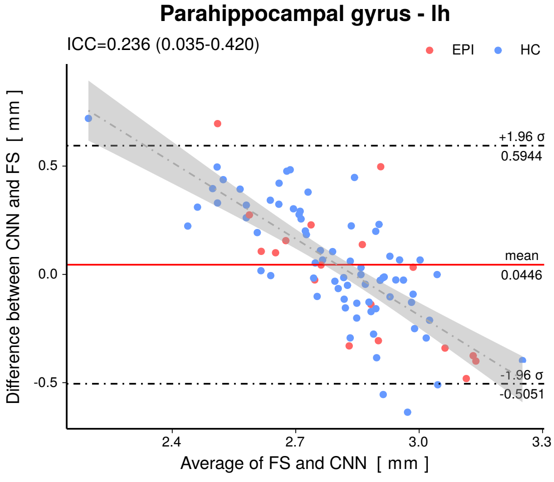
Parahippocampal gyrus - lh
ICC=0.236 (0.035-0.420)
EPI
HC
+1.96 σ
0.5944
mean
0.0446
-1.96 σ
-0.5051
Difference between CNN and FS [mm]
Average of FS and CNN [mm]

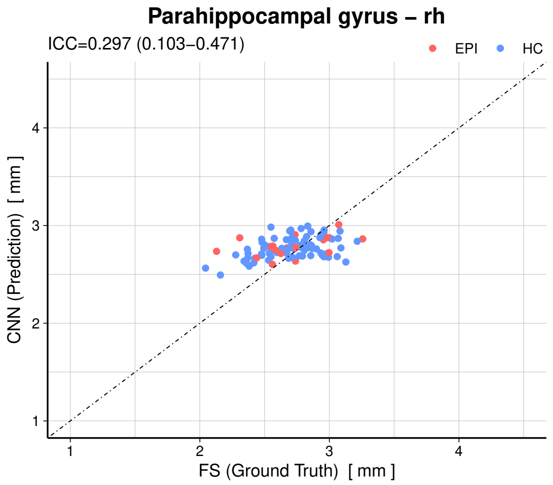
Parahippocampal gyrus – rh
ICC=0.297 (0.103–0.471)
EPI
HC
CNN (Prediction) [mm]
FS (Ground Truth) [mm]
1
2
3
4

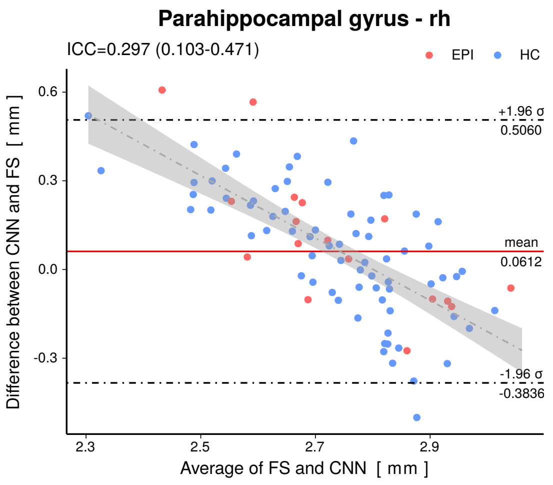
Parahippocampal gyrus - rh
ICC=0.297 (0.103-0.471)
EPI
HC
+1.96 σ
0.5060
mean
0.0612
-1.96 σ
-0.3836
0.6
0.3
0.0
-0.3
2.3
2.5
2.7
2.9
Difference between CNN and FS [mm]
Average of FS and CNN [mm]

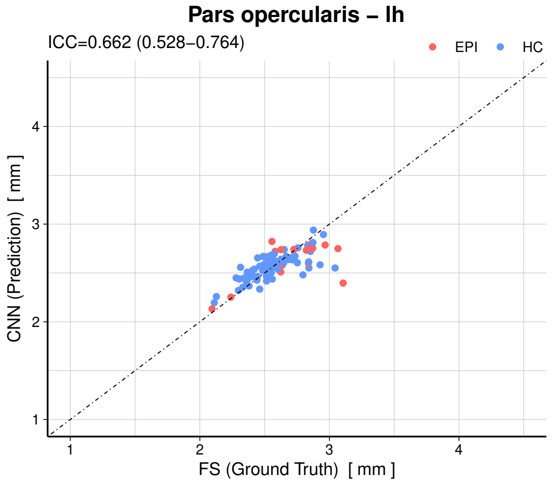
Pars opercularis – lh
ICC=0.662 (0.528–0.764)
EPI
HC
CNN (Prediction) [mm]
FS (Ground Truth) [mm]
1
2
3
4

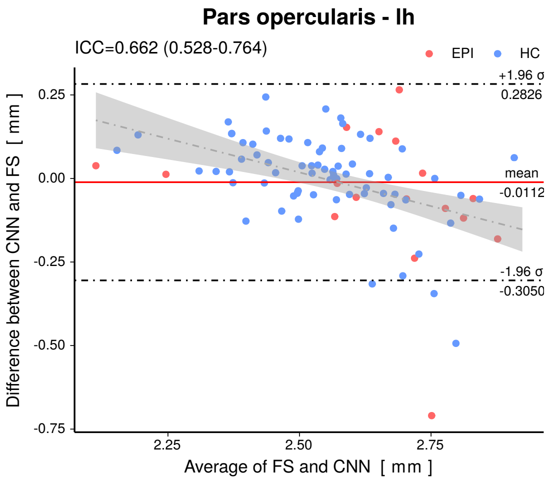
Pars opercularis - lh
ICC=0.662 (0.528-0.764)
EPI
HC
+1.96 σ
0.2826
mean
-0.0112
-1.96 σ
-0.3050
0.25
0.00
-0.25
-0.50
-0.75
2.25
2.50
2.75
Difference between CNN and FS [mm]
Average of FS and CNN [mm]

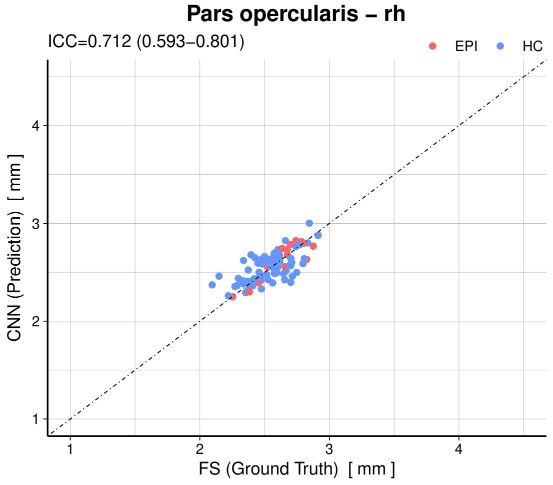
Pars opercularis – rh
ICC=0.712 (0.593–0.801)
EPI
HC
CNN (Prediction) [mm]
FS (Ground Truth) [mm]
1
2
3
4

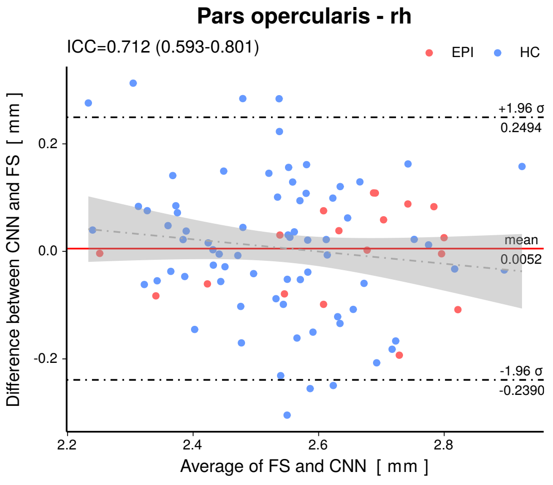
Pars opercularis - rh
ICC=0.712 (0.593-0.801)
EPI
HC
+1.96 σ
0.2494
mean
0.0052
-1.96 σ
-0.2390
0.2
0.0
-0.2
2.2
2.4
2.6
2.8
Difference between CNN and FS [mm]
Average of FS and CNN [mm]

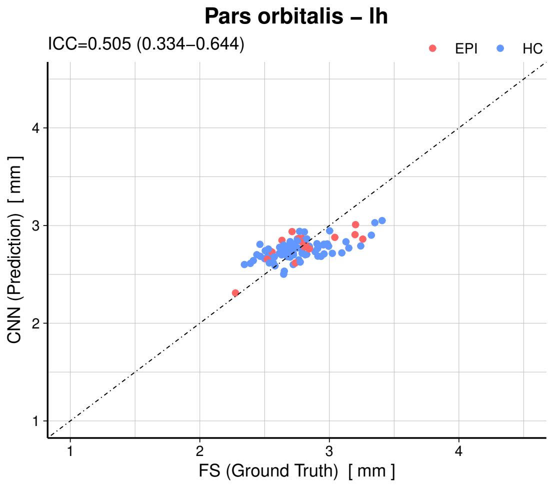
Pars orbitalis – lh
ICC=0.505 (0.334–0.644)
EPI
HC
CNN (Prediction) [mm]
FS (Ground Truth) [mm]

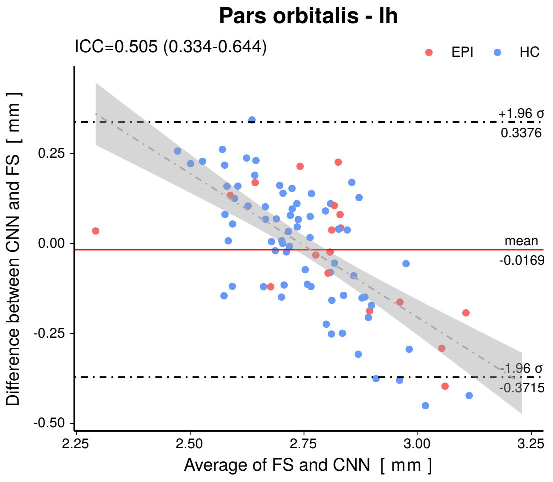
Pars orbitalis - lh
ICC=0.505 (0.334-0.644)
EPI
HC
+1.96 σ
0.3376
mean
-0.0169
-1.96 σ
-0.3715
Difference between CNN and FS [mm]
Average of FS and CNN [mm]

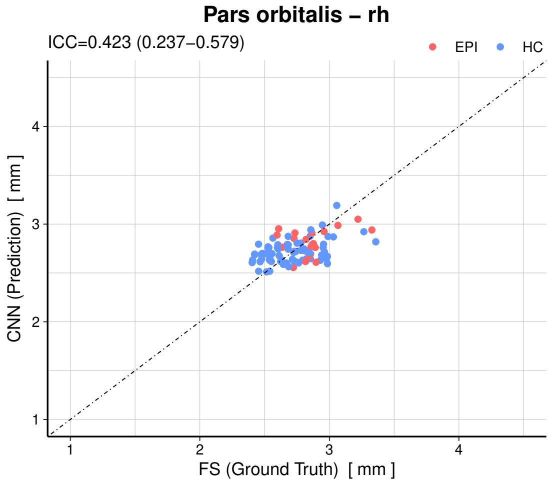
Pars orbitalis – rh
ICC=0.423 (0.237–0.579)
EPI
HC
CNN (Prediction) [mm]
FS (Ground Truth) [mm]

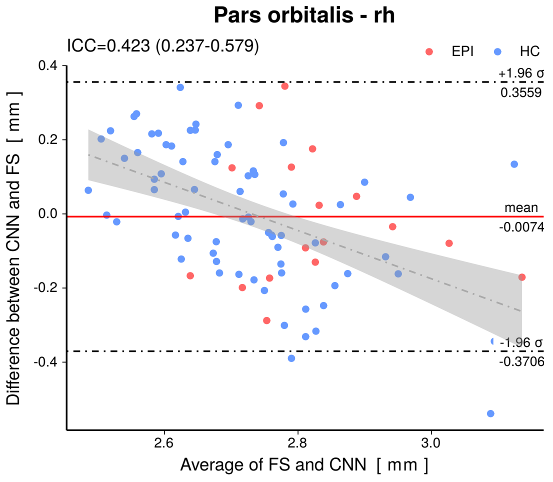
Pars orbitalis - rh
ICC=0.423 (0.237-0.579)
EPI
HC
+1.96 σ
0.3559
mean
-0.0074
-1.96 σ
-0.3706
Difference between CNN and FS [mm]
Average of FS and CNN [mm]

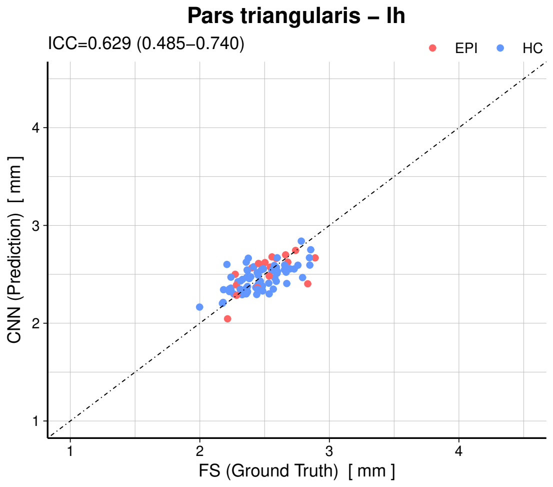
Pars triangularis – lh
ICC=0.629 (0.485–0.740)
EPI
HC
CNN (Prediction) [mm]
FS (Ground Truth) [mm]

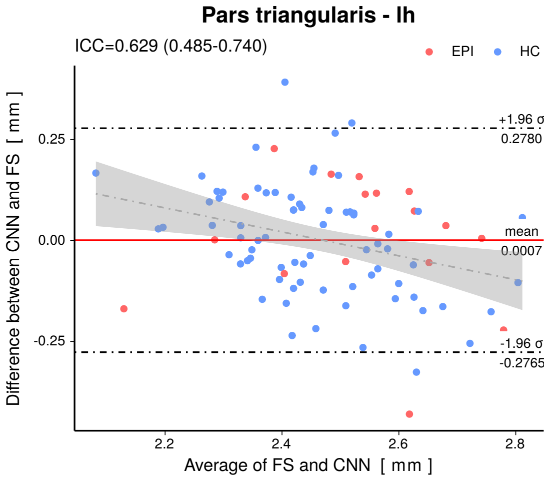
Pars triangularis - lh
ICC=0.629 (0.485-0.740)
EPI
HC
+1.96 σ
0.2780
mean
0.0007
-1.96 σ
-0.2765
Difference between CNN and FS [mm]
Average of FS and CNN [mm]

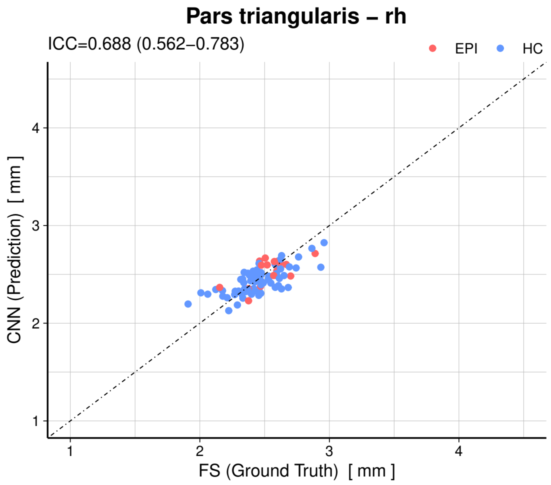
Pars triangularis – rh
ICC=0.688 (0.562–0.783)
EPI
HC
CNN (Prediction) [mm]
FS (Ground Truth) [mm]

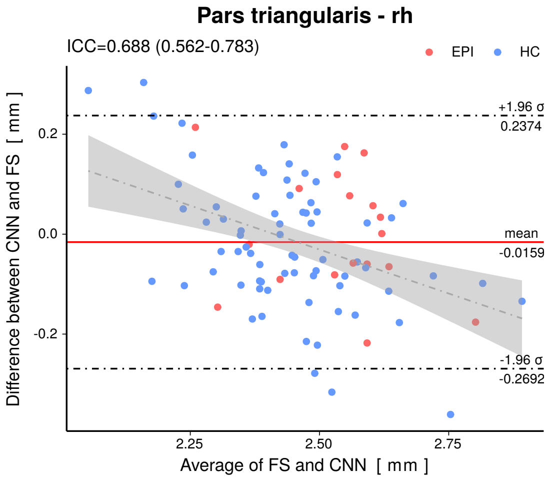
Pars triangularis - rh
ICC=0.688 (0.562-0.783)
EPI
HC
+1.96 σ
0.2374
mean
-0.0159
-1.96 σ
-0.2692
Difference between CNN and FS [mm]
Average of FS and CNN [mm]

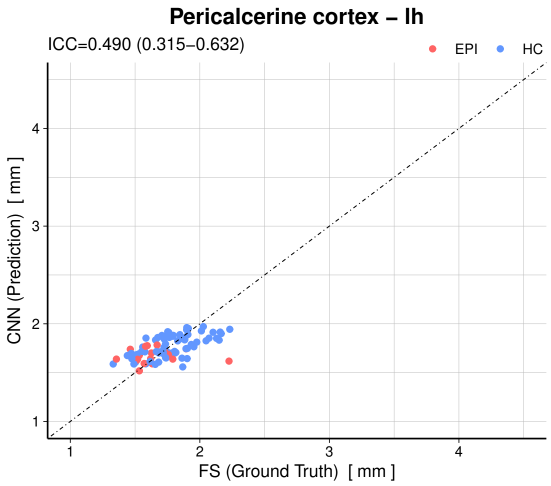
Pericalcerine cortex – lh
ICC=0.490 (0.315–0.632)
EPI
HC
CNN (Prediction) [mm]
FS (Ground Truth) [mm]

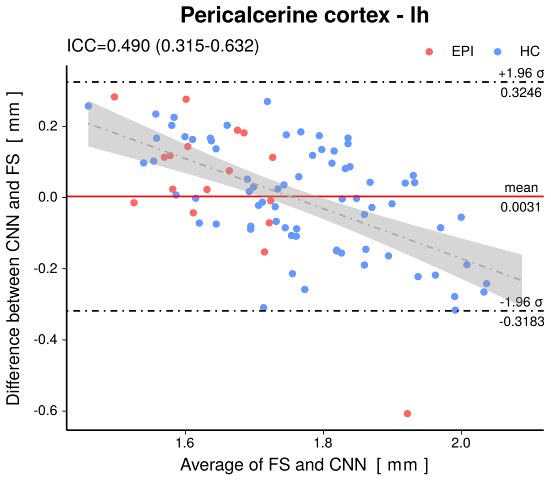
Pericalcerine cortex - lh
ICC=0.490 (0.315-0.632)
EPI
HC
+1.96 σ
0.3246
mean
0.0031
-1.96 σ
-0.3183
Difference between CNN and FS [mm]
Average of FS and CNN [mm]

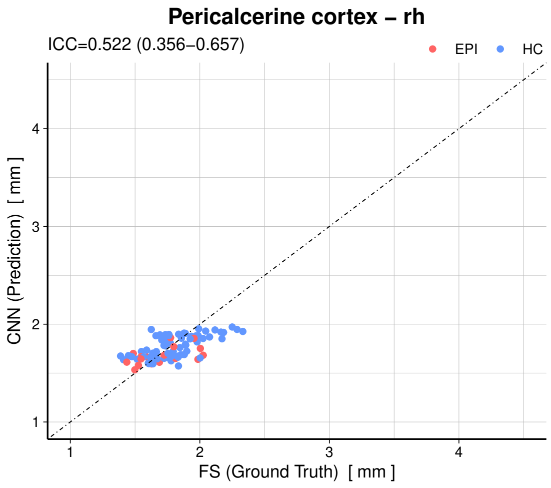
Pericalcerine cortex – rh
ICC=0.522 (0.356–0.657)
EPI
HC
CNN (Prediction) [mm]
FS (Ground Truth) [mm]

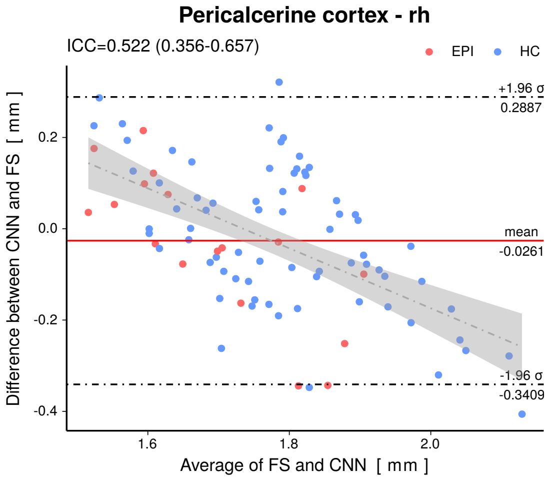
Pericalcerine cortex - rh
ICC=0.522 (0.356-0.657)
EPI
HC
+1.96 σ
0.2887
mean
-0.0261
-1.96 σ
-0.3409
Difference between CNN and FS [mm]
Average of FS and CNN [mm]

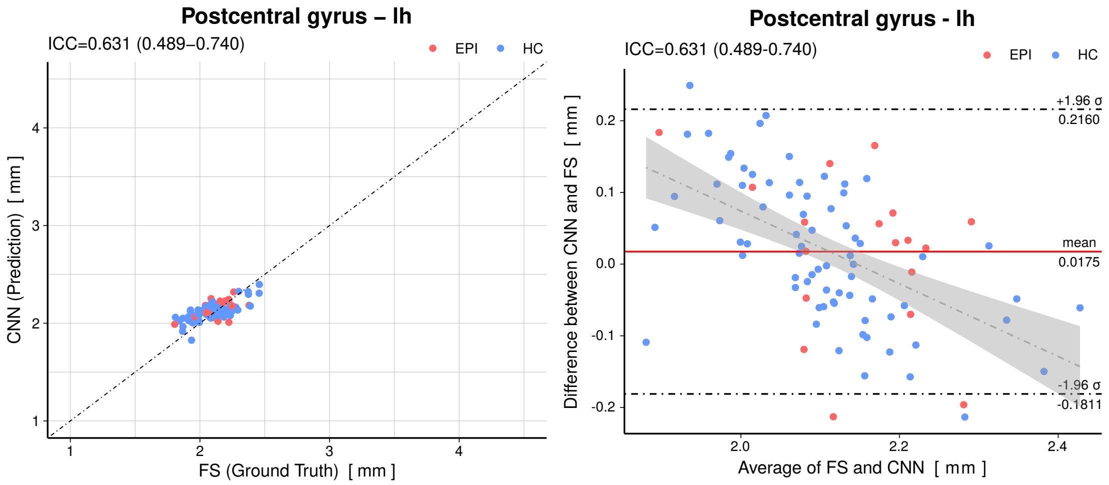
Postcentral gyrus – lh
ICC=0.631 (0.489–0.740)
EPI HC
CNN (Prediction) [mm]
FS (Ground Truth) [mm]
Postcentral gyrus - lh
ICC=0.631 (0.489-0.740)
+1.96 σ
0.2160
mean
0.0175
-1.96 σ
-0.1811
Difference between CNN and FS [mm]
Average of FS and CNN [mm]

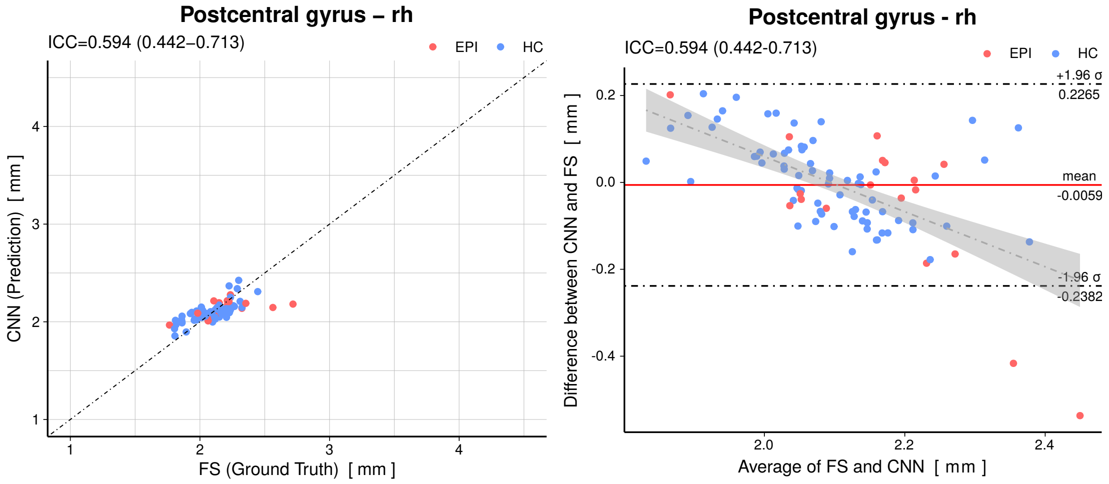
Postcentral gyrus – rh
ICC=0.594 (0.442–0.713)
EPI HC
CNN (Prediction) [mm]
FS (Ground Truth) [mm]
Postcentral gyrus - rh
ICC=0.594 (0.442-0.713)
+1.96 σ
0.2265
mean
-0.0059
-1.96 σ
-0.2382
Difference between CNN and FS [mm]
Average of FS and CNN [mm]

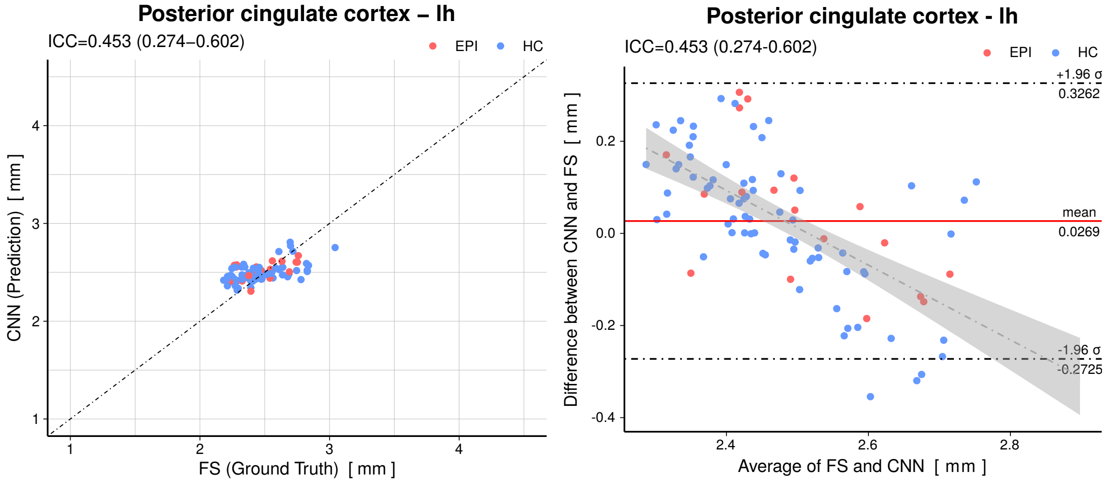
Posterior cingulate cortex – lh
ICC=0.453 (0.274–0.602)
EPI HC
CNN (Prediction) [mm]
FS (Ground Truth) [mm]
Posterior cingulate cortex - lh
ICC=0.453 (0.274-0.602)
+1.96 σ
0.3262
mean
0.0269
-1.96 σ
-0.2725
Difference between CNN and FS [mm]
Average of FS and CNN [mm]

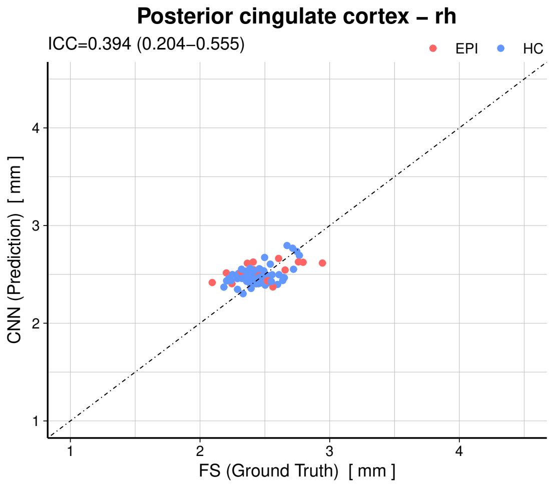
Posterior cingulate cortex – rh
ICC=0.394 (0.204–0.555)
EPI
HC
CNN (Prediction) [mm]
FS (Ground Truth) [mm]

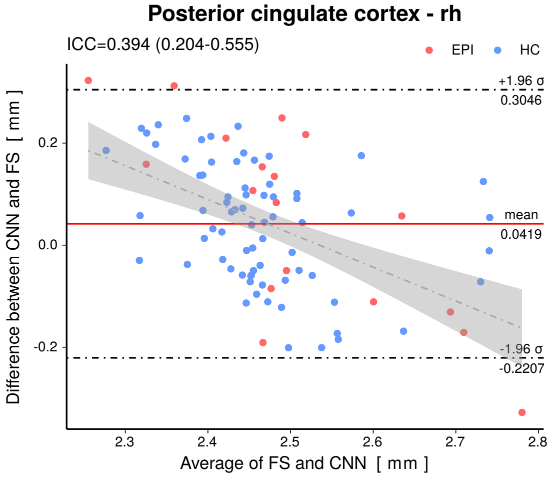
Posterior cingulate cortex - rh
ICC=0.394 (0.204-0.555)
EPI
HC
+1.96 σ
0.3046
mean
0.0419
-1.96 σ
-0.2207
Difference between CNN and FS [mm]
Average of FS and CNN [mm]

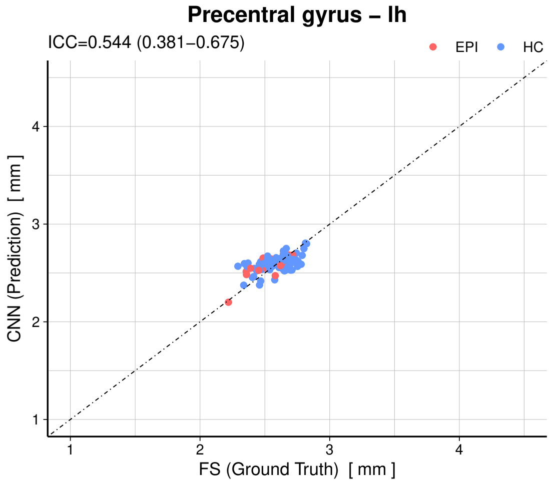
Precentral gyrus – lh
ICC=0.544 (0.381–0.675)
EPI
HC
CNN (Prediction) [mm]
FS (Ground Truth) [mm]

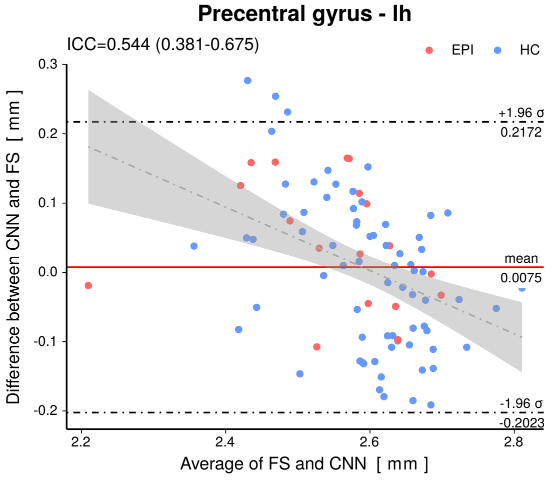
Precentral gyrus - lh
ICC=0.544 (0.381-0.675)
EPI
HC
+1.96 σ
0.2172
mean
0.0075
-1.96 σ
-0.2023
Difference between CNN and FS [mm]
Average of FS and CNN [mm]

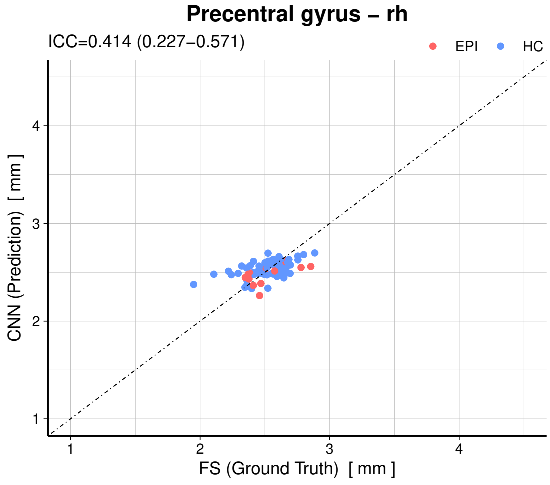
Precentral gyrus – rh
ICC=0.414 (0.227–0.571)
EPI
HC
CNN (Prediction) [mm]
FS (Ground Truth) [mm]

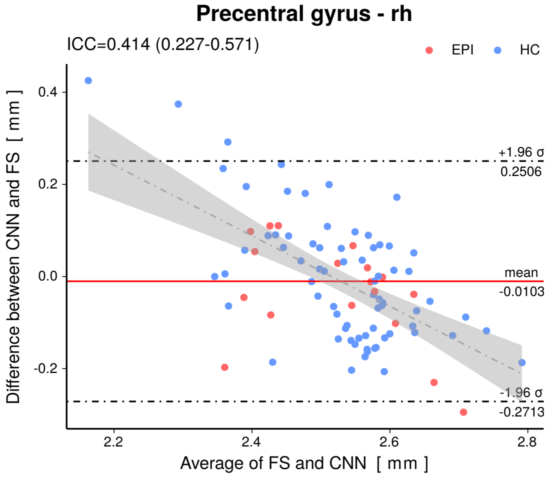
Precentral gyrus - rh
ICC=0.414 (0.227-0.571)
EPI
HC
+1.96 σ
0.2506
mean
-0.0103
-1.96 σ
-0.2713
Difference between CNN and FS [mm]
Average of FS and CNN [mm]

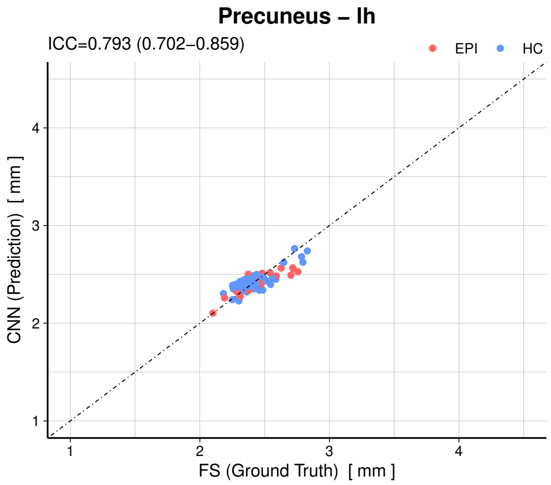
Precuneus – lh
ICC=0.793 (0.702–0.859)
EPI
HC
CNN (Prediction) [mm]
FS (Ground Truth) [mm]

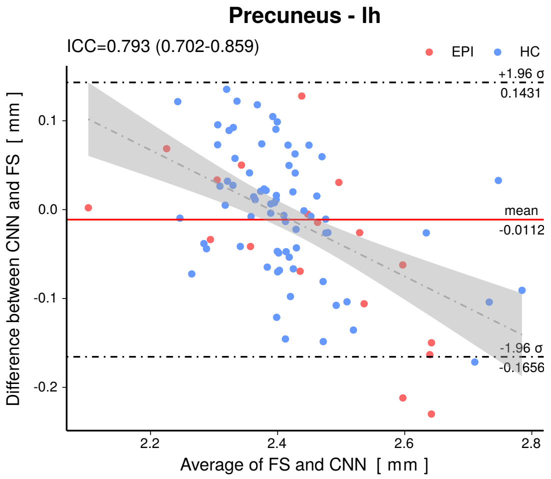
Precuneus - lh
ICC=0.793 (0.702-0.859)
EPI
HC
+1.96 σ
0.1431
mean
-0.0112
-1.96 σ
-0.1656
Difference between CNN and FS [mm]
Average of FS and CNN [mm]

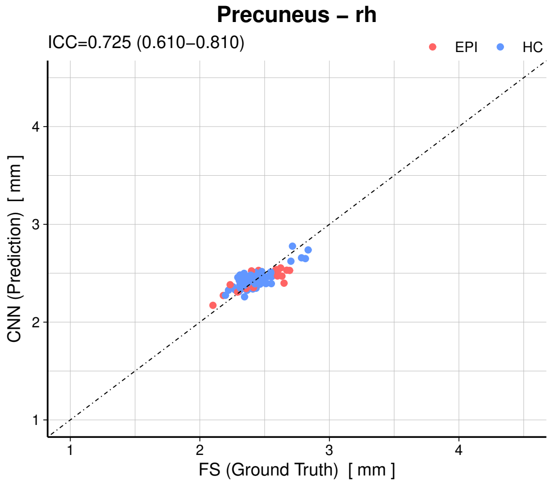
Precuneus – rh
ICC=0.725 (0.610–0.810)
EPI
HC
CNN (Prediction) [mm]
FS (Ground Truth) [mm]

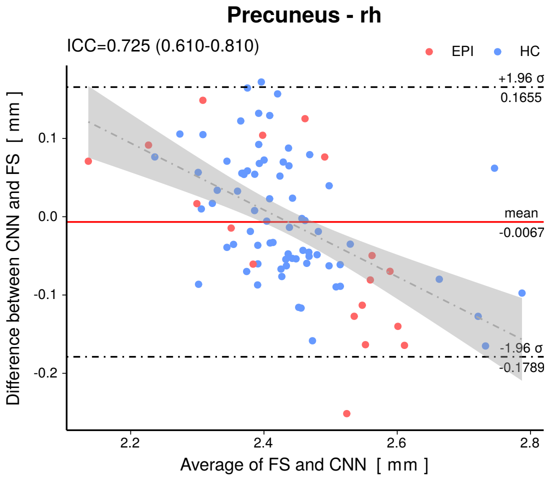
Precuneus - rh
ICC=0.725 (0.610-0.810)
EPI
HC
+1.96 σ
0.1655
mean
-0.0067
-1.96 σ
-0.1789
Difference between CNN and FS [mm]
Average of FS and CNN [mm]

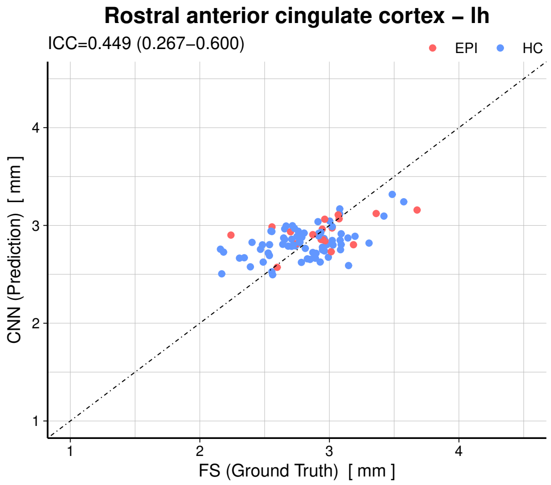
Rostral anterior cingulate cortex – lh
ICC=0.449 (0.267–0.600)
EPI
HC
CNN (Prediction) [mm]
FS (Ground Truth) [mm]

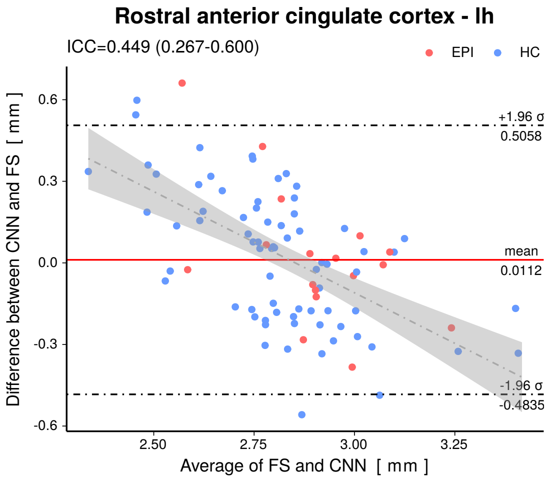
Rostral anterior cingulate cortex - lh
ICC=0.449 (0.267-0.600)
EPI
HC
+1.96 σ
0.5058
mean
0.0112
-1.96 σ
-0.4835
Difference between CNN and FS [mm]
Average of FS and CNN [mm]

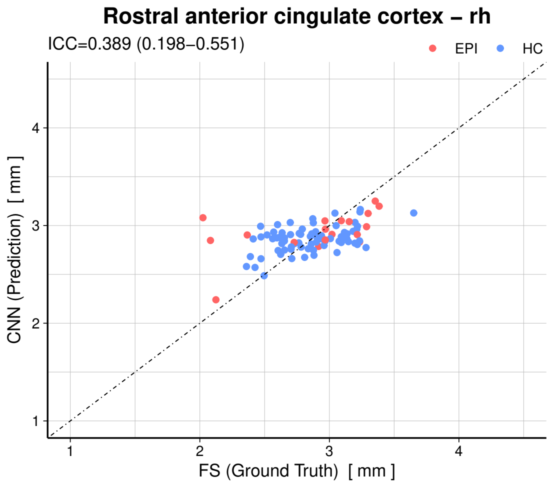
Rostral anterior cingulate cortex – rh
ICC=0.389 (0.198–0.551)
EPI
HC
CNN (Prediction) [mm]
FS (Ground Truth) [mm]

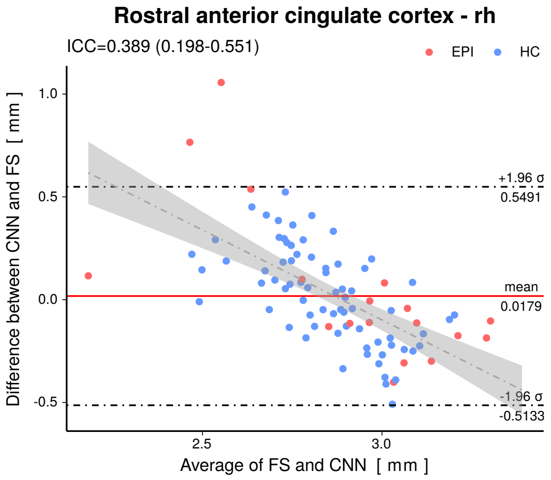
Rostral anterior cingulate cortex - rh
ICC=0.389 (0.198-0.551)
EPI
HC
+1.96 σ
0.5491
mean
0.0179
-1.96 σ
-0.5133
Difference between CNN and FS [mm]
Average of FS and CNN [mm]

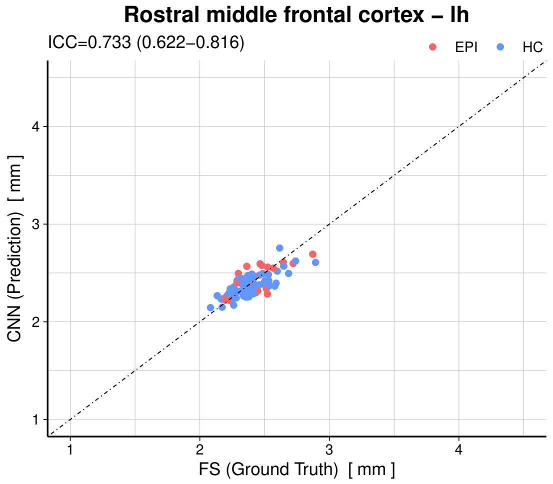
Rostral middle frontal cortex – lh
ICC=0.733 (0.622–0.816)
EPI
HC
CNN (Prediction) [mm]
FS (Ground Truth) [mm]

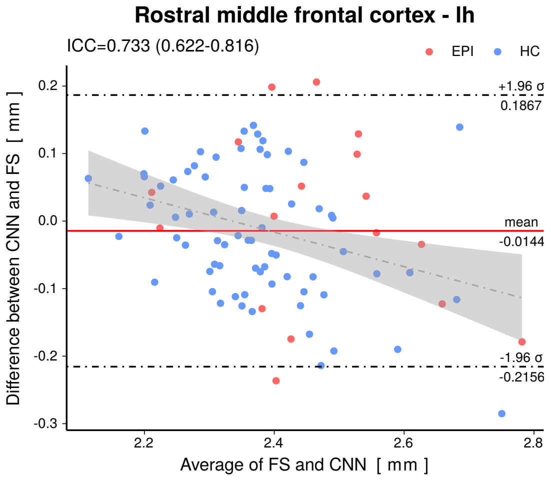
Rostral middle frontal cortex - lh
ICC=0.733 (0.622-0.816)
EPI
HC
+1.96 σ
0.1867
mean
-0.0144
-1.96 σ
-0.2156
Difference between CNN and FS [mm]
Average of FS and CNN [mm]

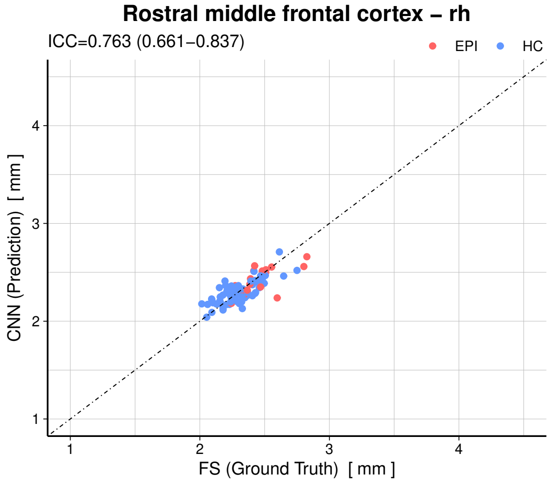
Rostral middle frontal cortex – rh
ICC=0.763 (0.661–0.837)
EPI
HC
CNN (Prediction) [mm]
FS (Ground Truth) [mm]

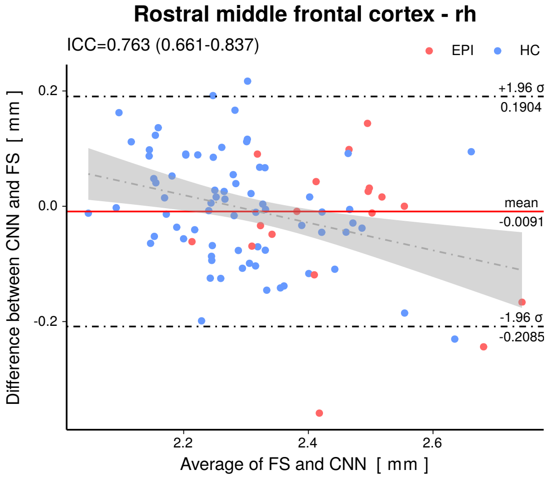
Rostral middle frontal cortex - rh
ICC=0.763 (0.661-0.837)
EPI
HC
+1.96 σ
0.1904
mean
-0.0091
-1.96 σ
-0.2085
Difference between CNN and FS [mm]
Average of FS and CNN [mm]

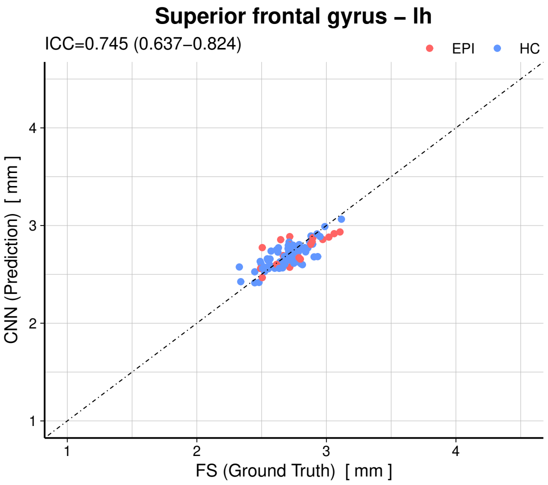
Superior frontal gyrus – lh
ICC=0.745 (0.637–0.824)
EPI
HC
CNN (Prediction) [mm]
FS (Ground Truth) [mm]

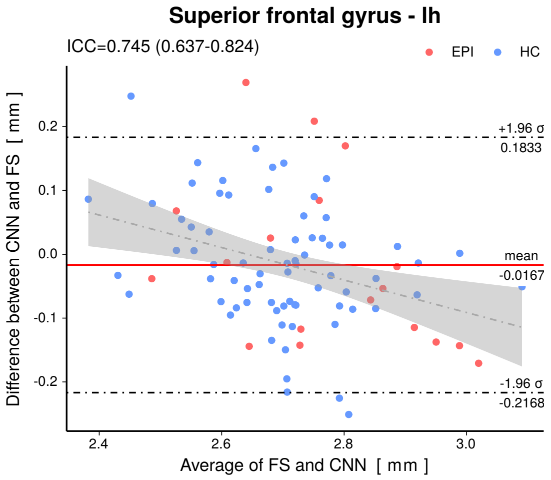
Superior frontal gyrus - lh
ICC=0.745 (0.637-0.824)
EPI
HC
+1.96 σ
0.1833
mean
-0.0167
-1.96 σ
-0.2168
Difference between CNN and FS [mm]
Average of FS and CNN [mm]

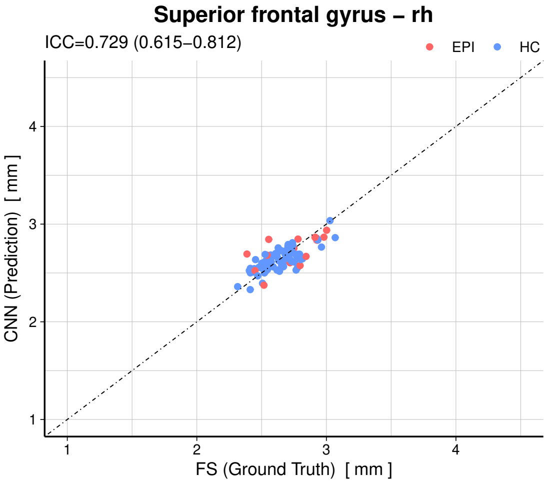
Superior frontal gyrus – rh
ICC=0.729 (0.615–0.812)
EPI
HC
CNN (Prediction) [mm]
FS (Ground Truth) [mm]

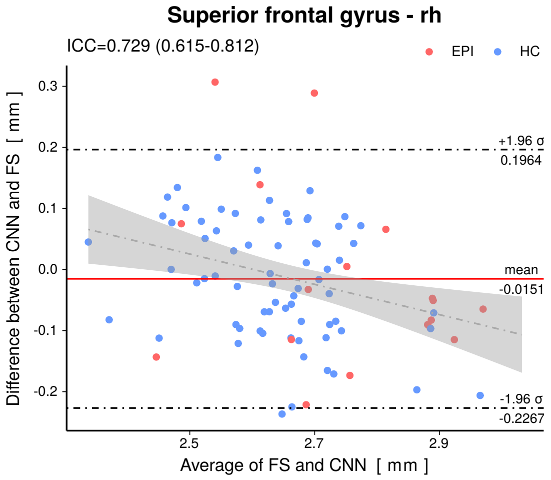
Superior frontal gyrus - rh
ICC=0.729 (0.615-0.812)
EPI
HC
+1.96 σ
0.1964
mean
-0.0151
-1.96 σ
-0.2267
Difference between CNN and FS [mm]
Average of FS and CNN [mm]

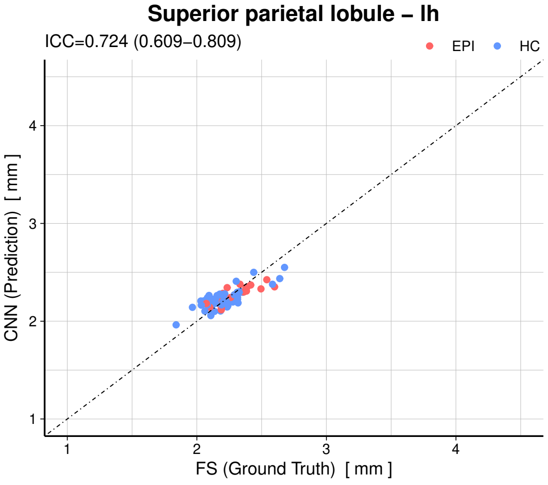
Superior parietal lobule – lh
ICC=0.724 (0.609–0.809)
EPI
HC
CNN (Prediction) [mm]
FS (Ground Truth) [mm]

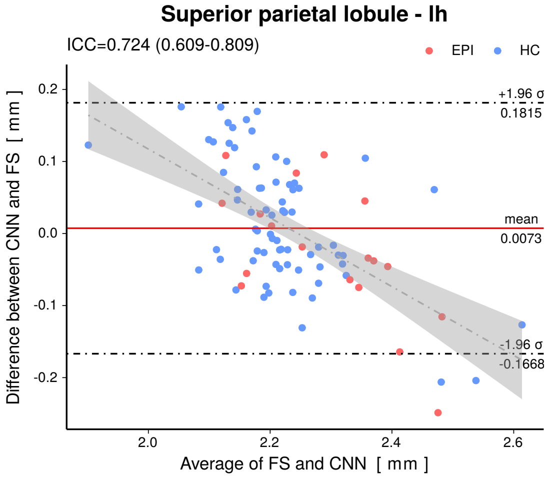
Superior parietal lobule - lh
ICC=0.724 (0.609-0.809)
EPI
HC
+1.96 σ
0.1815
mean
0.0073
-1.96 σ
-0.1668
Difference between CNN and FS [mm]
Average of FS and CNN [mm]

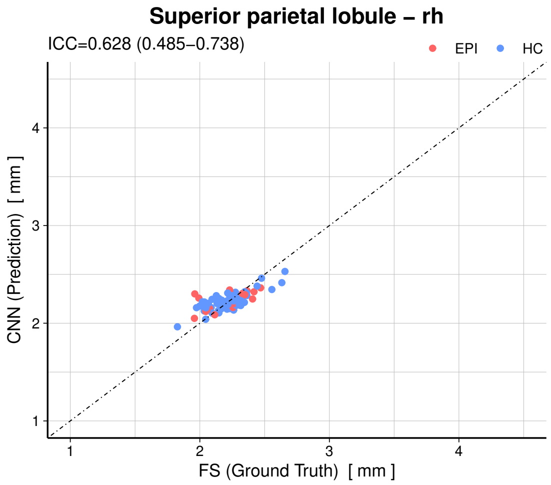
Superior parietal lobule – rh
ICC=0.628 (0.485–0.738)
EPI
HC
CNN (Prediction) [mm]
FS (Ground Truth) [mm]

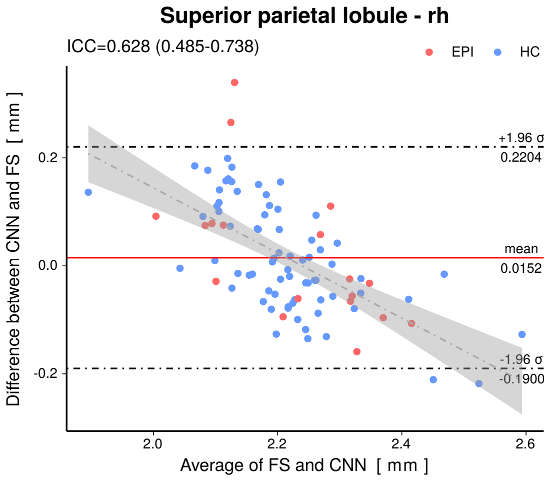
Superior parietal lobule - rh
ICC=0.628 (0.485-0.738)
EPI
HC
+1.96 σ
0.2204
mean
0.0152
-1.96 σ
-0.1900
Difference between CNN and FS [mm]
Average of FS and CNN [mm]

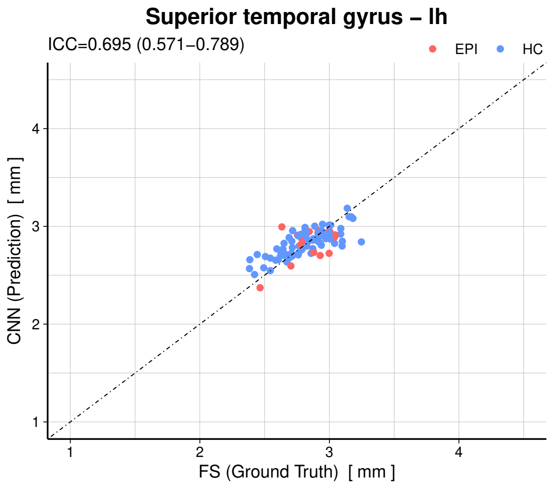
Superior temporal gyrus – lh
ICC=0.695 (0.571–0.789)
EPI
HC
CNN (Prediction) [mm]
FS (Ground Truth) [mm]

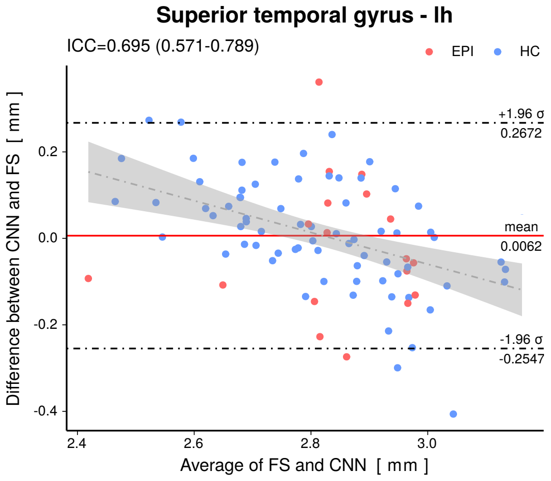
Superior temporal gyrus - lh
ICC=0.695 (0.571-0.789)
EPI
HC
+1.96 σ
0.2672
mean
0.0062
-1.96 σ
-0.2547
Difference between CNN and FS [mm]
Average of FS and CNN [mm]

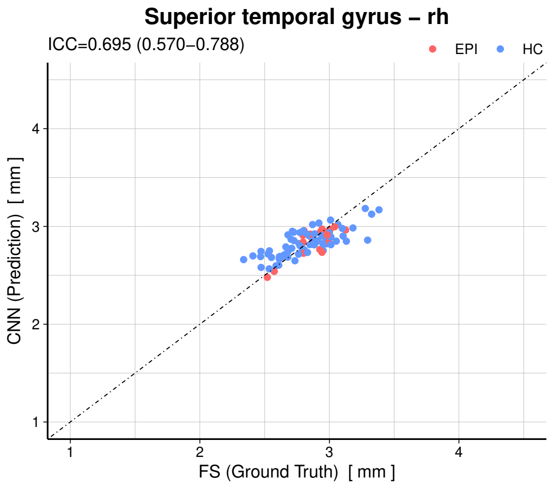
Superior temporal gyrus – rh
ICC=0.695 (0.570–0.788)
EPI
HC
CNN (Prediction) [mm]
FS (Ground Truth) [mm]

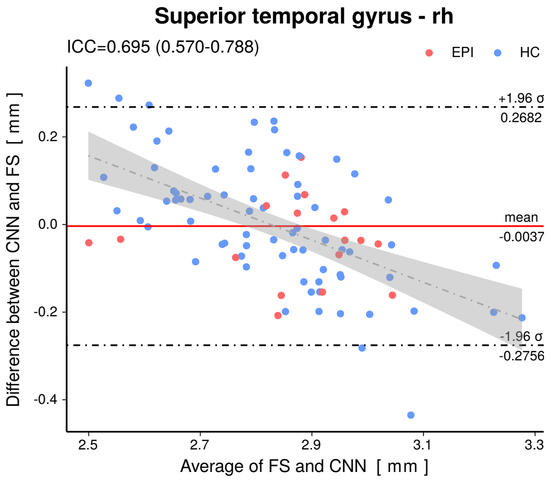
Superior temporal gyrus - rh
ICC=0.695 (0.570-0.788)
EPI
HC
+1.96 σ
0.2682
mean
-0.0037
-1.96 σ
-0.2756
Difference between CNN and FS [mm]
Average of FS and CNN [mm]

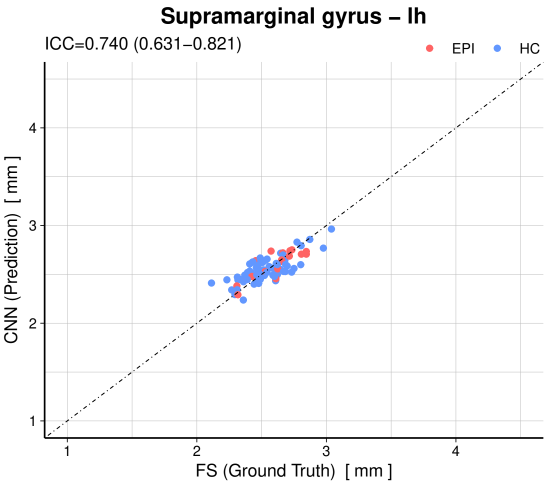
Supramarginal gyrus – lh
ICC=0.740 (0.631–0.821)
EPI
HC
CNN (Prediction) [mm]
FS (Ground Truth) [mm]

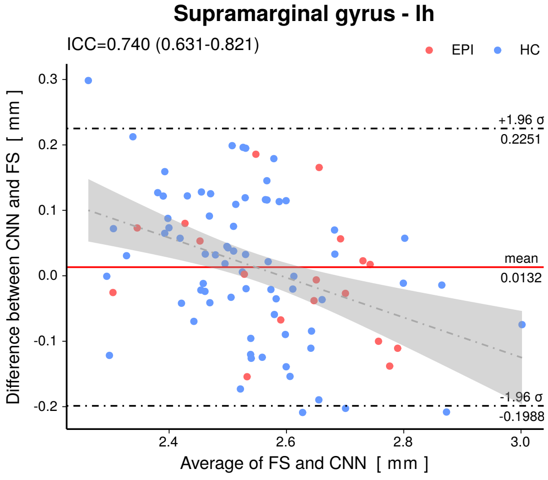
Supramarginal gyrus - lh
ICC=0.740 (0.631-0.821)
EPI
HC
+1.96 σ
0.2251
mean
0.0132
-1.96 σ
-0.1988
Difference between CNN and FS [mm]
Average of FS and CNN [mm]

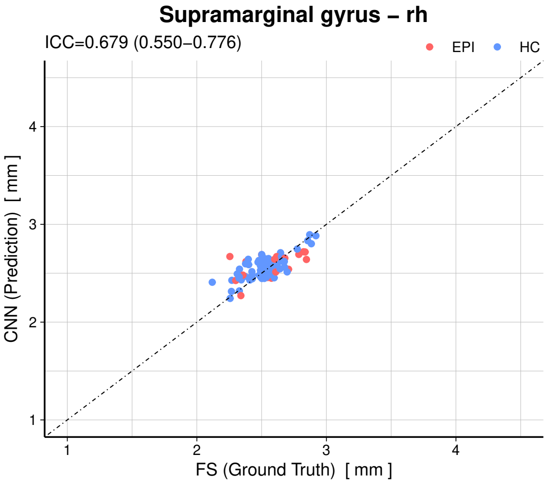
Supramarginal gyrus – rh
ICC=0.679 (0.550–0.776)
EPI
HC
CNN (Prediction) [mm]
FS (Ground Truth) [mm]

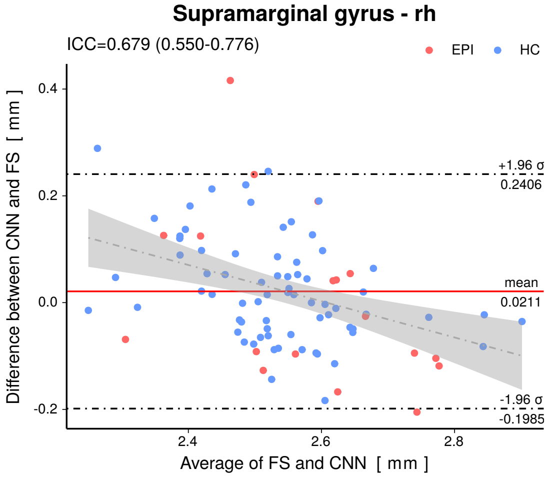
Supramarginal gyrus - rh
ICC=0.679 (0.550-0.776)
EPI
HC
+1.96 σ
0.2406
mean
0.0211
-1.96 σ
-0.1985
Difference between CNN and FS [mm]
Average of FS and CNN [mm]

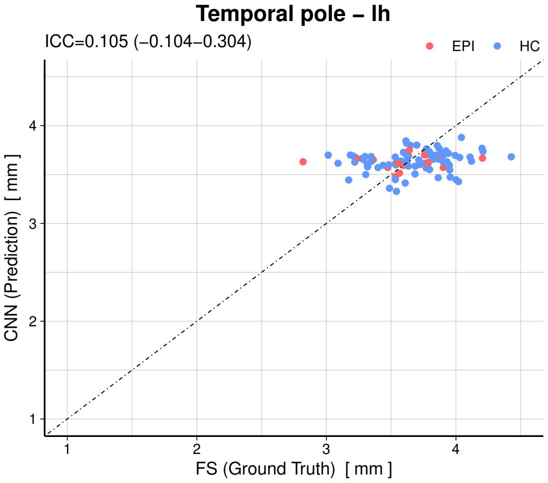
Temporal pole – lh
ICC=0.105 (−0.104–0.304)
EPI
HC
CNN (Prediction) [mm]
FS (Ground Truth) [mm]

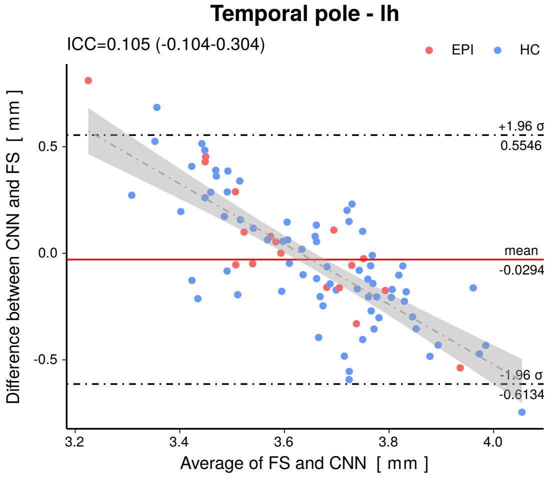
Temporal pole - lh
ICC=0.105 (-0.104-0.304)
EPI
HC
+1.96 σ
0.5546
mean
-0.0294
-1.96 σ
-0.6134
Difference between CNN and FS [mm]
Average of FS and CNN [mm]

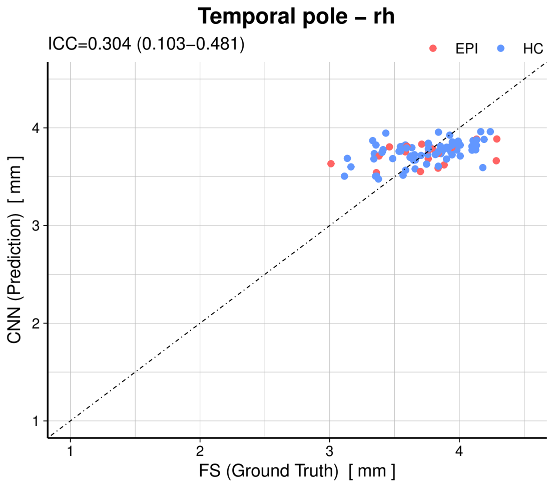
Temporal pole – rh
ICC=0.304 (0.103–0.481)
EPI
HC
CNN (Prediction) [mm]
FS (Ground Truth) [mm]

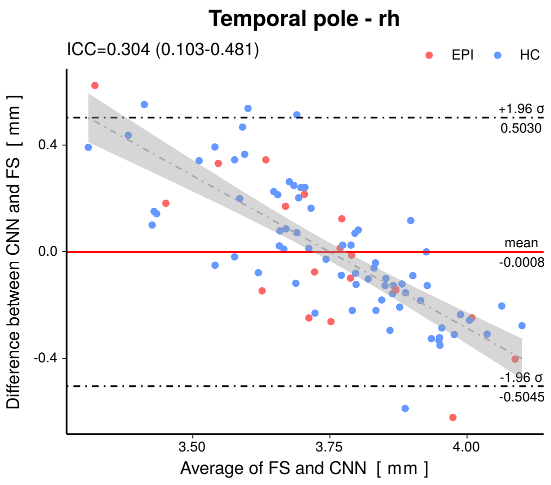
Temporal pole - rh
ICC=0.304 (0.103-0.481)
EPI
HC
+1.96 σ
0.5030
mean
-0.0008
-1.96 σ
-0.5045
Difference between CNN and FS [mm]
Average of FS and CNN [mm]

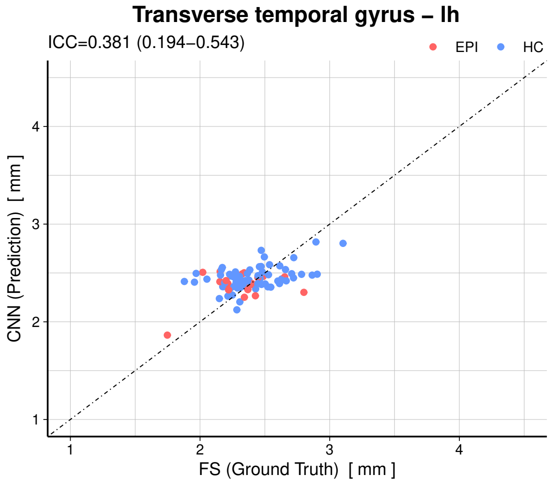
Transverse temporal gyrus – lh
ICC=0.381 (0.194–0.543)
EPI
HC
CNN (Prediction) [mm]
FS (Ground Truth) [mm]

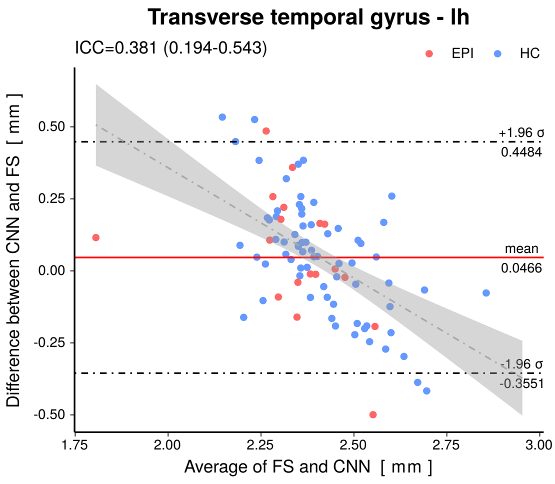
Transverse temporal gyrus - lh
ICC=0.381 (0.194-0.543)
EPI
HC
+1.96 σ
0.4484
mean
0.0466
-1.96 σ
-0.3551
Difference between CNN and FS [mm]
Average of FS and CNN [mm]

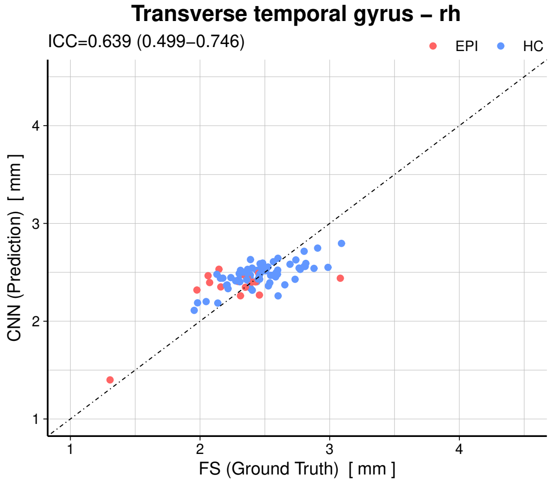
Transverse temporal gyrus – rh
ICC=0.639 (0.499–0.746)
EPI
HC
CNN (Prediction) [mm]
FS (Ground Truth) [mm]

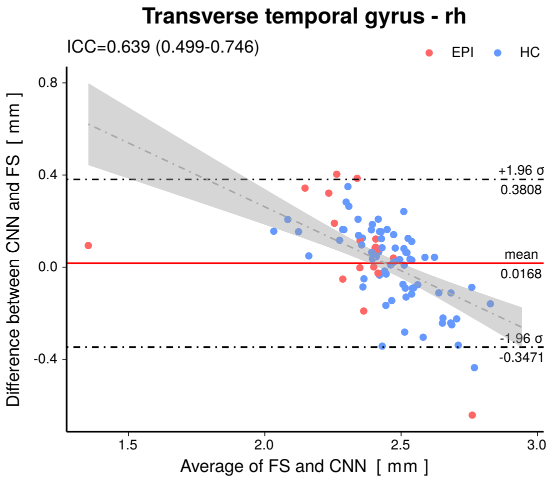
Transverse temporal gyrus - rh
ICC=0.639 (0.499-0.746)
EPI
HC
+1.96 σ
0.3808
mean
0.0168
-1.96 σ
-0.3471
Difference between CNN and FS [mm]
Average of FS and CNN [mm]

3.3 Correlation and Bland-Altman Plots for Cortical Curvature

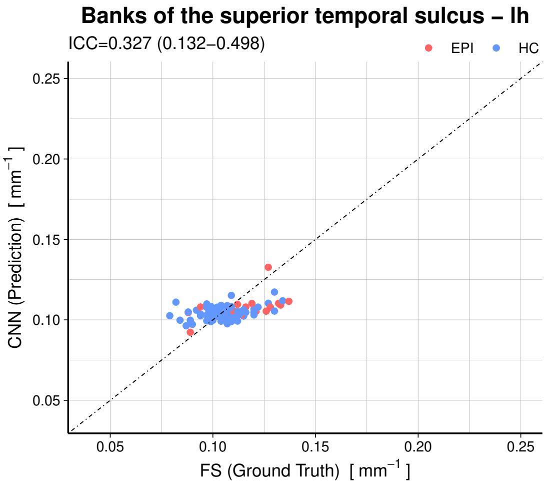

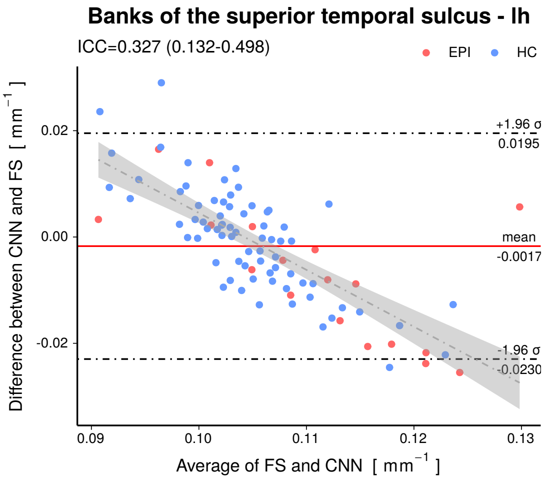

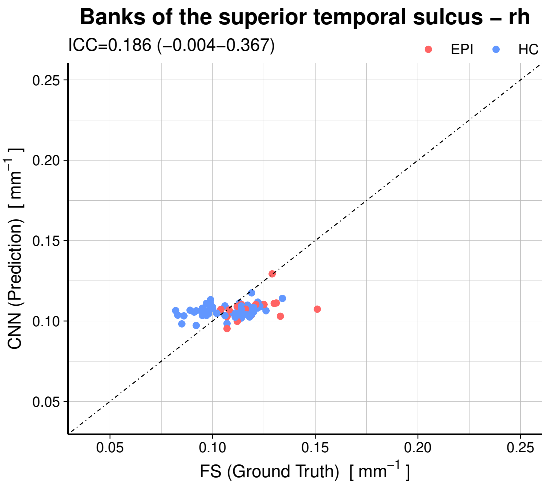

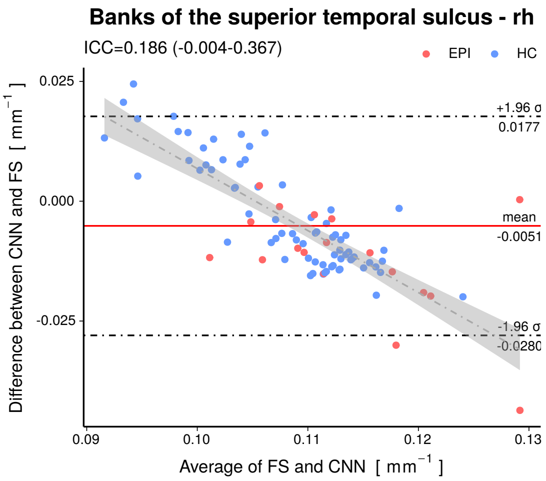

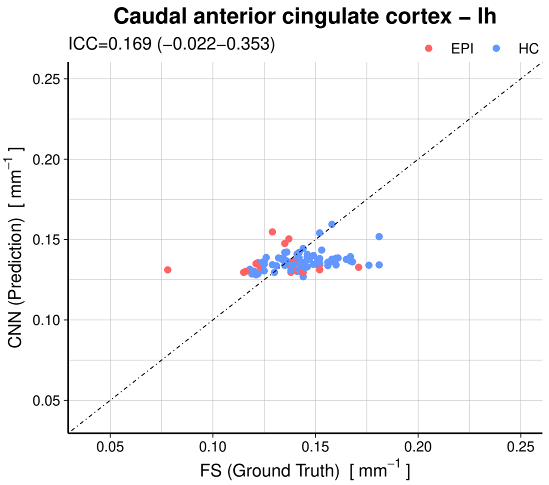
Caudal anterior cingulate cortex – lh
ICC=0.169 (−0.022–0.353)
EPI HC
CNN (Prediction) [mm^{-1}]
FS (Ground Truth) [mm^{-1}]

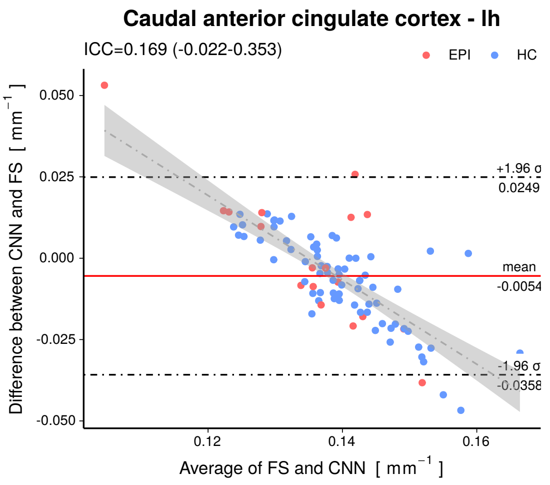
Caudal anterior cingulate cortex - lh
ICC=0.169 (-0.022-0.353)
EPI HC
+1.96 σ
0.0249
mean
-0.0054
-1.96 σ
-0.0358
Difference between CNN and FS [mm^{-1}]
Average of FS and CNN [mm^{-1}]

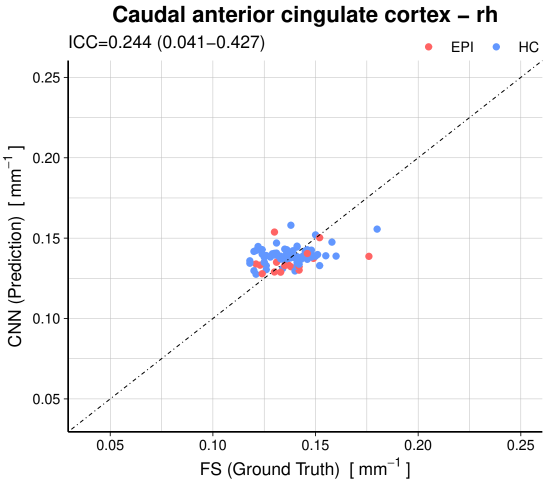
Caudal anterior cingulate cortex – rh
ICC=0.244 (0.041–0.427)
EPI HC
CNN (Prediction) [mm^{-1}]
FS (Ground Truth) [mm^{-1}]

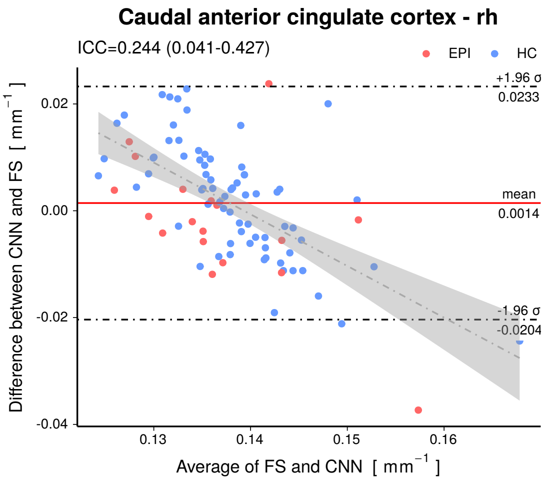
Caudal anterior cingulate cortex - rh
ICC=0.244 (0.041-0.427)
EPI HC
+1.96 σ
0.0233
mean
0.0014
-1.96 σ
-0.0204
Difference between CNN and FS [mm^{-1}]
Average of FS and CNN [mm^{-1}]

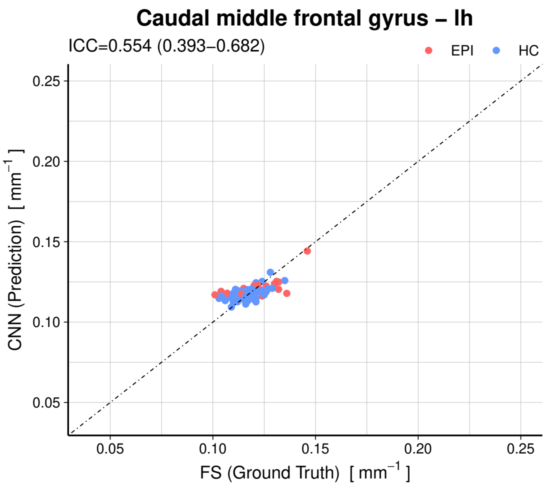
Caudal middle frontal gyrus – lh
ICC=0.554 (0.393–0.682)
EPI HC
CNN (Prediction) [mm^{-1}]
FS (Ground Truth) [mm^{-1}]

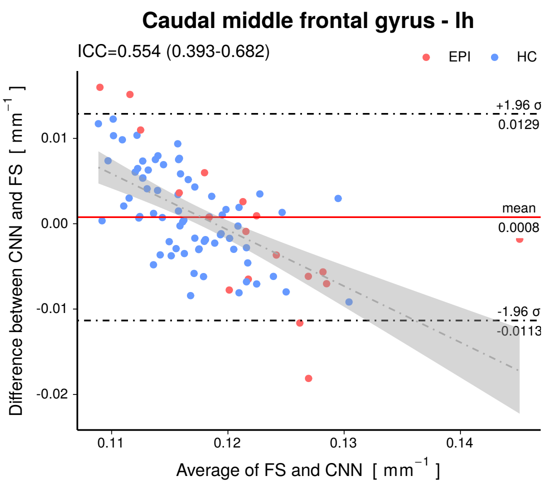
Caudal middle frontal gyrus - lh
ICC=0.554 (0.393-0.682)
EPI HC
+1.96 σ
0.0129
mean
0.0008
-1.96 σ
-0.0113
Difference between CNN and FS [mm^{-1}]
Average of FS and CNN [mm^{-1}]

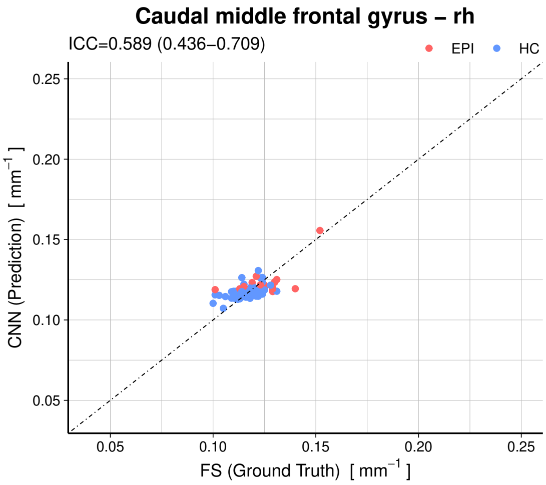
Caudal middle frontal gyrus – rh
ICC=0.589 (0.436–0.709)
EPI
HC
CNN (Prediction) [mm⁻¹]
FS (Ground Truth) [mm⁻¹]

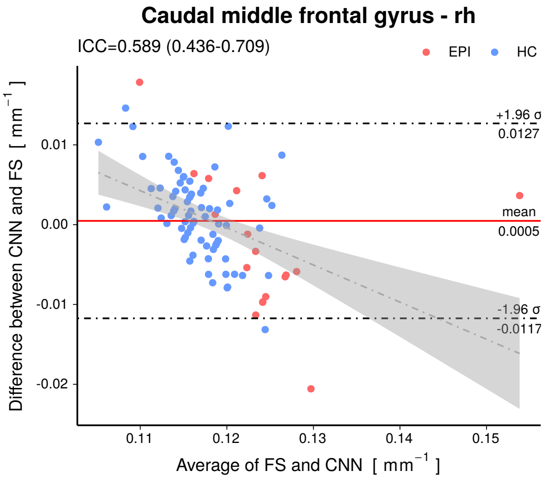
Caudal middle frontal gyrus - rh
ICC=0.589 (0.436-0.709)
EPI
HC
+1.96 σ
0.0127
mean
0.0005
-1.96 σ
-0.0117
Difference between CNN and FS [mm⁻¹]
Average of FS and CNN [mm⁻¹]

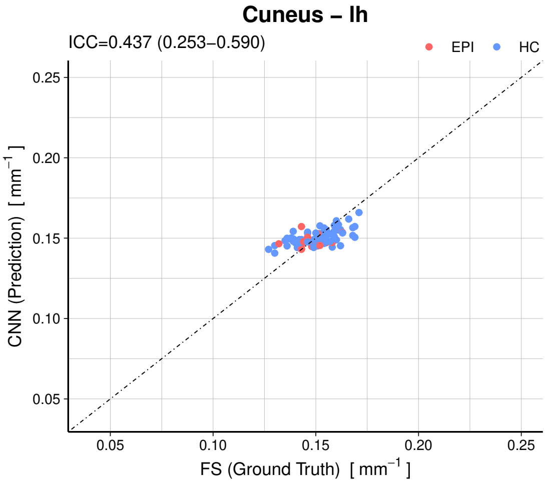
Cuneus – lh
ICC=0.437 (0.253–0.590)
EPI
HC
CNN (Prediction) [mm⁻¹]
FS (Ground Truth) [mm⁻¹]

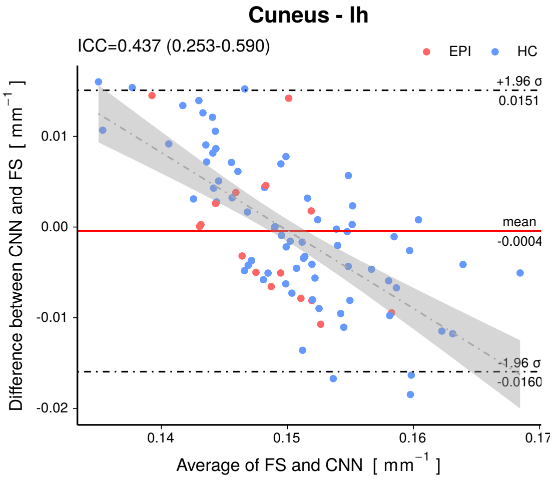
Cuneus - lh
ICC=0.437 (0.253-0.590)
EPI
HC
+1.96 σ
0.0151
mean
-0.0004
-1.96 σ
-0.0160
Difference between CNN and FS [mm⁻¹]
Average of FS and CNN [mm⁻¹]

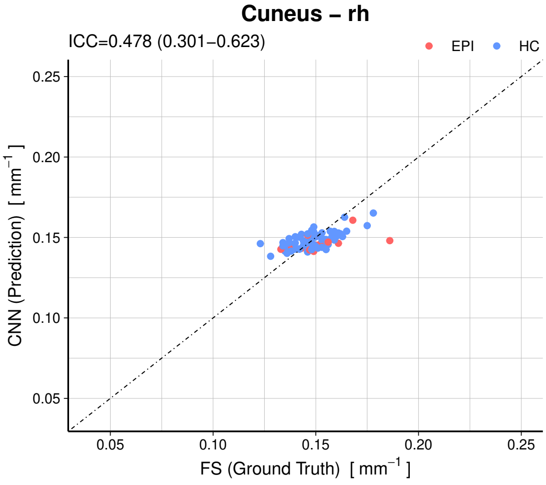
Cuneus – rh
ICC=0.478 (0.301–0.623)
EPI
HC
CNN (Prediction) [mm⁻¹]
FS (Ground Truth) [mm⁻¹]

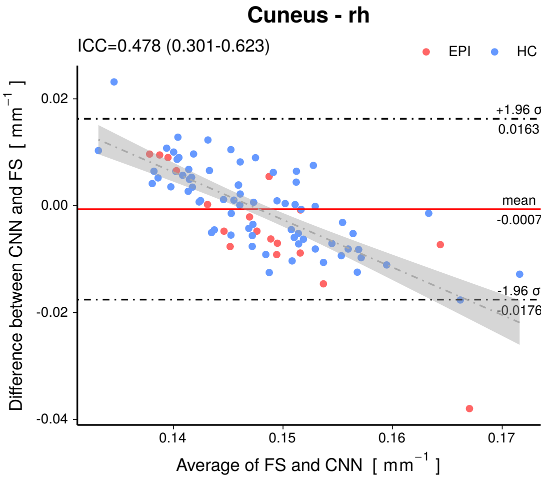
Cuneus - rh
ICC=0.478 (0.301-0.623)
EPI
HC
+1.96 σ
0.0163
mean
-0.0007
-1.96 σ
-0.0176
Difference between CNN and FS [mm⁻¹]
Average of FS and CNN [mm⁻¹]

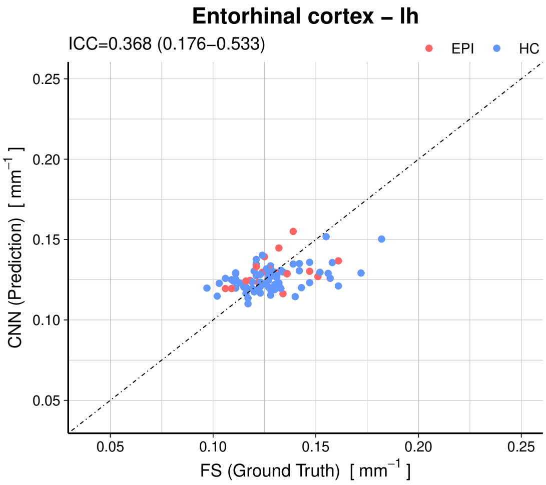
Entorhinal cortex – lh
ICC=0.368 (0.176–0.533)
EPI
HC
CNN (Prediction) [mm^−1]
FS (Ground Truth) [mm^−1]
0.05
0.10
0.15
0.20
0.25

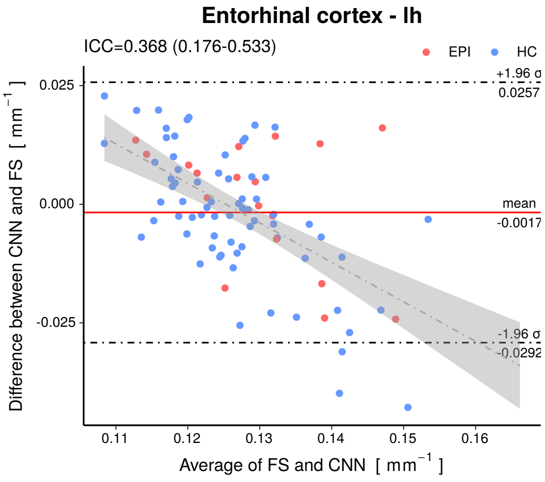
Entorhinal cortex - lh
ICC=0.368 (0.176-0.533)
EPI
HC
+1.96 σ
0.0257
mean
-0.0017
-1.96 σ
-0.0292
Difference between CNN and FS [mm^−1]
Average of FS and CNN [mm^−1]
0.025
0.000
-0.025
0.11
0.12
0.13
0.14
0.15
0.16

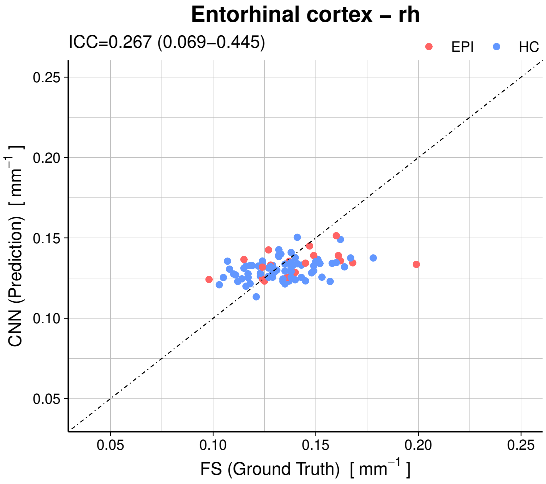
Entorhinal cortex – rh
ICC=0.267 (0.069–0.445)
EPI
HC
CNN (Prediction) [mm^−1]
FS (Ground Truth) [mm^−1]
0.05
0.10
0.15
0.20
0.25

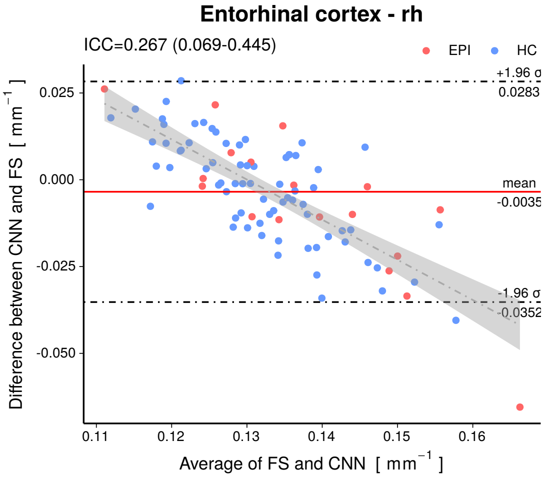
Entorhinal cortex - rh
ICC=0.267 (0.069-0.445)
EPI
HC
+1.96 σ
0.0283
mean
-0.0035
-1.96 σ
-0.0352
Difference between CNN and FS [mm^−1]
Average of FS and CNN [mm^−1]
0.025
0.000
-0.025
-0.050
0.11
0.12
0.13
0.14
0.15
0.16

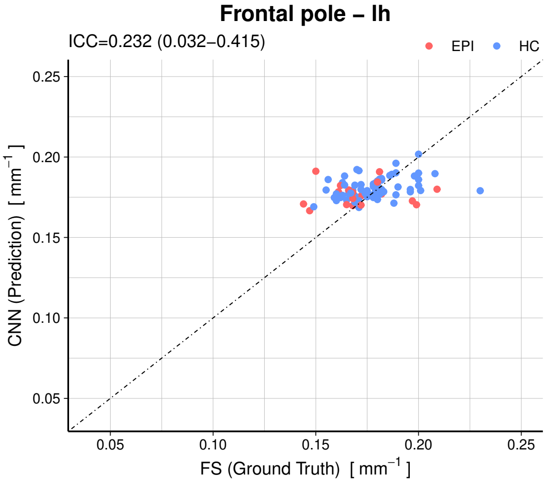
Frontal pole – lh
ICC=0.232 (0.032–0.415)
EPI
HC
CNN (Prediction) [mm^−1]
FS (Ground Truth) [mm^−1]
0.05
0.10
0.15
0.20
0.25

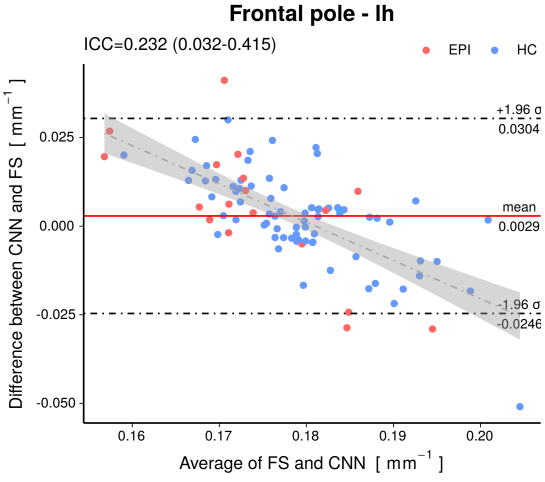
Frontal pole - lh
ICC=0.232 (0.032-0.415)
EPI
HC
+1.96 σ
0.0304
mean
0.0029
-1.96 σ
-0.0246
Difference between CNN and FS [mm^−1]
Average of FS and CNN [mm^−1]
0.025
0.000
-0.025
-0.050
0.16
0.17
0.18
0.19
0.20

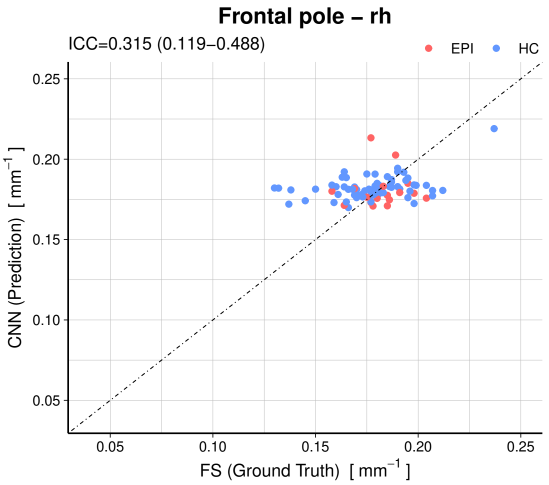
Frontal pole – rh
ICC=0.315 (0.119–0.488)
EPI
HC
CNN (Prediction) [mm^{-1}]
FS (Ground Truth) [mm^{-1}]
0.05
0.10
0.15
0.20
0.25

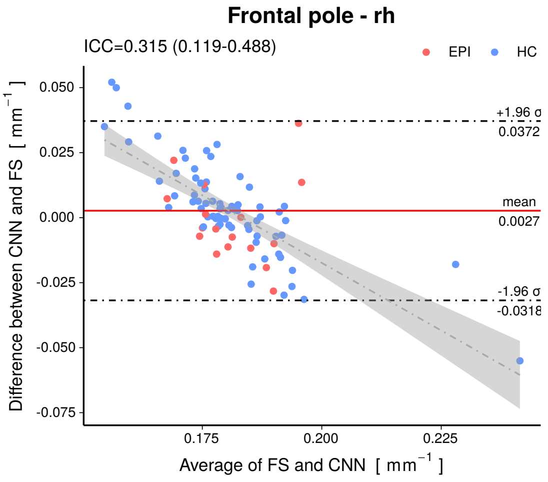
Frontal pole - rh
ICC=0.315 (0.119-0.488)
EPI
HC
+1.96 σ
0.0372
mean
0.0027
-1.96 σ
-0.0318
Difference between CNN and FS [mm^{-1}]
Average of FS and CNN [mm^{-1}]
0.050
0.025
0.000
-0.025
-0.050
-0.075
0.175
0.200
0.225

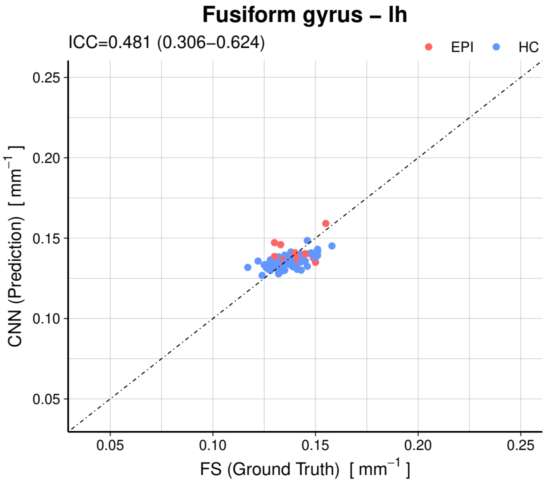
Fusiform gyrus – lh
ICC=0.481 (0.306–0.624)
EPI
HC
CNN (Prediction) [mm^{-1}]
FS (Ground Truth) [mm^{-1}]
0.05
0.10
0.15
0.20
0.25

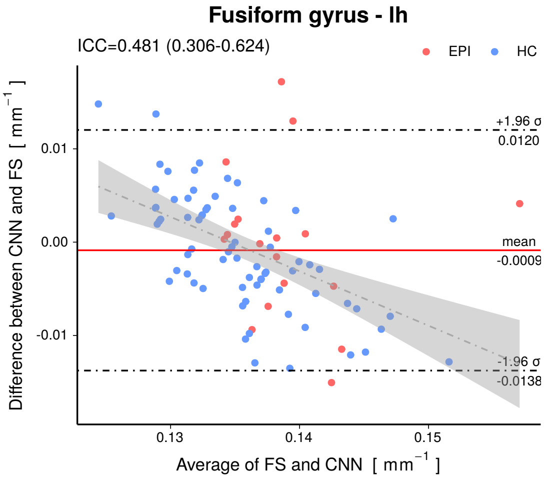
Fusiform gyrus - lh
ICC=0.481 (0.306-0.624)
EPI
HC
+1.96 σ
0.0120
mean
-0.0009
-1.96 σ
-0.0138
Difference between CNN and FS [mm^{-1}]
Average of FS and CNN [mm^{-1}]
0.01
0.00
-0.01
0.13
0.14
0.15

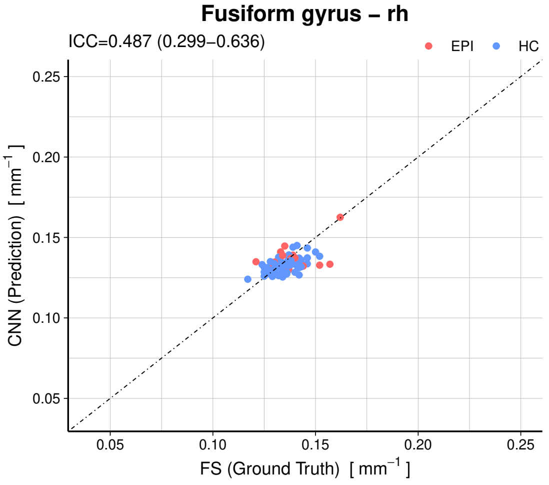
Fusiform gyrus – rh
ICC=0.487 (0.299–0.636)
EPI
HC
CNN (Prediction) [mm^{-1}]
FS (Ground Truth) [mm^{-1}]
0.05
0.10
0.15
0.20
0.25

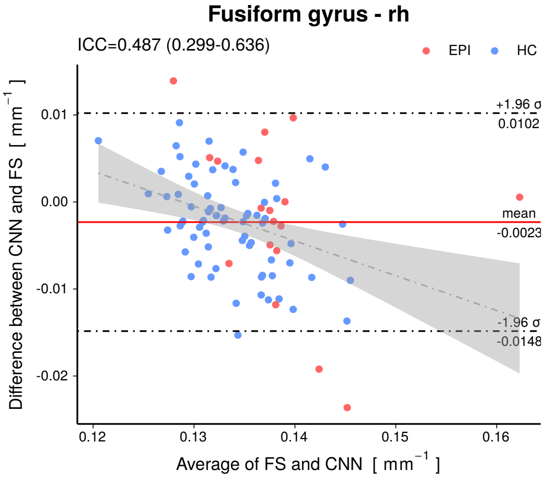
Fusiform gyrus - rh
ICC=0.487 (0.299-0.636)
EPI
HC
+1.96 σ
0.0102
mean
-0.0023
-1.96 σ
-0.0148
Difference between CNN and FS [mm^{-1}]
Average of FS and CNN [mm^{-1}]
0.01
0.00
-0.01
-0.02
0.12
0.13
0.14
0.15
0.16

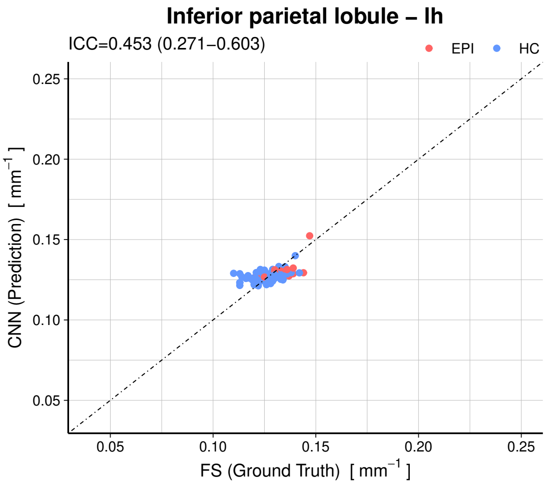
Inferior parietal lobule – lh
ICC=0.453 (0.271–0.603)
EPI
HC
CNN (Prediction) [mm^{-1}]
FS (Ground Truth) [mm^{-1}]
0.05
0.10
0.15
0.20
0.25

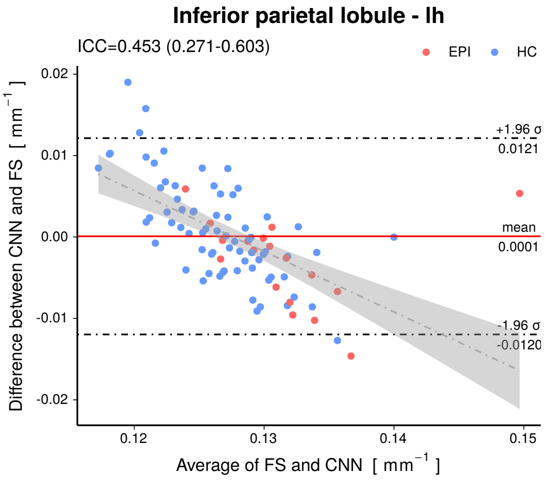
Inferior parietal lobule - lh
ICC=0.453 (0.271-0.603)
EPI
HC
+1.96 σ
0.0121
mean
0.0001
-1.96 σ
-0.0120
Difference between CNN and FS [mm^{-1}]
Average of FS and CNN [mm^{-1}]
0.02
0.01
0.00
-0.01
-0.02
0.12
0.13
0.14
0.15

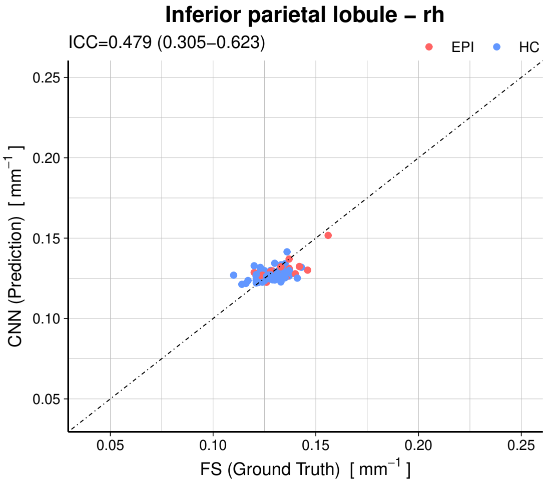
Inferior parietal lobule – rh
ICC=0.479 (0.305–0.623)
EPI
HC
CNN (Prediction) [mm^{-1}]
FS (Ground Truth) [mm^{-1}]
0.05
0.10
0.15
0.20
0.25

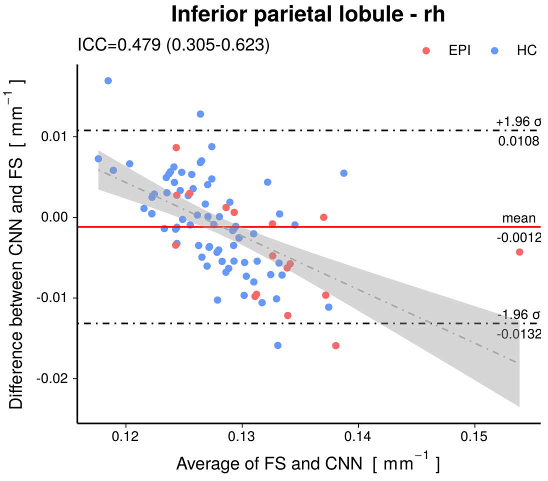
Inferior parietal lobule - rh
ICC=0.479 (0.305-0.623)
EPI
HC
+1.96 σ
0.0108
mean
-0.0012
-1.96 σ
-0.0132
Difference between CNN and FS [mm^{-1}]
Average of FS and CNN [mm^{-1}]
0.01
0.00
-0.01
-0.02
0.12
0.13
0.14
0.15

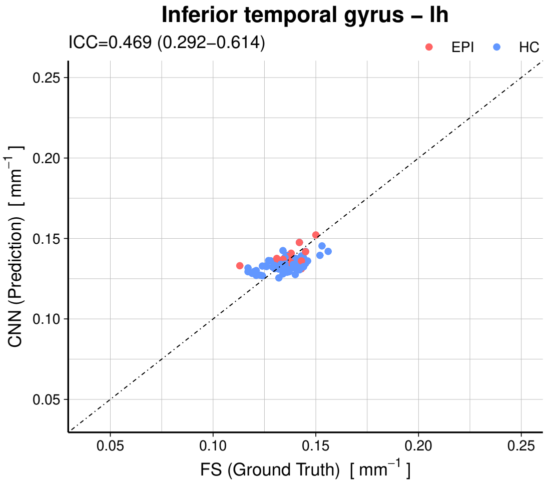
Inferior temporal gyrus – lh
ICC=0.469 (0.292–0.614)
EPI
HC
CNN (Prediction) [mm^{-1}]
FS (Ground Truth) [mm^{-1}]
0.05
0.10
0.15
0.20
0.25

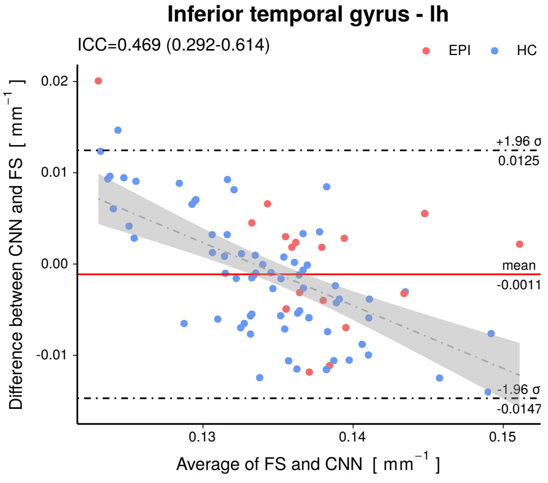
Inferior temporal gyrus - lh
ICC=0.469 (0.292-0.614)
EPI
HC
+1.96 σ
0.0125
mean
-0.0011
-1.96 σ
-0.0147
Difference between CNN and FS [mm^{-1}]
Average of FS and CNN [mm^{-1}]
0.02
0.01
0.00
-0.01
0.13
0.14
0.15

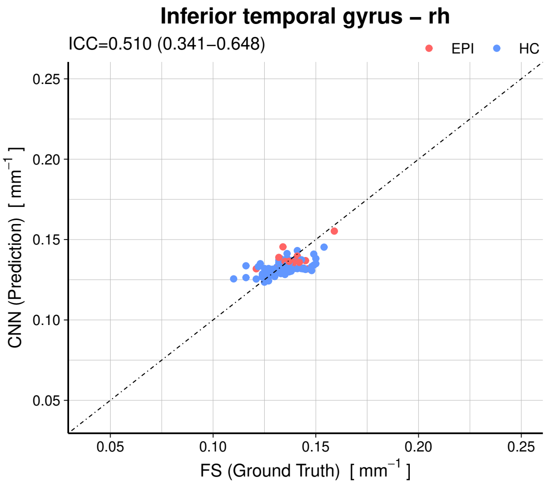
Inferior temporal gyrus – rh
ICC=0.510 (0.341–0.648)
EPI
HC
CNN (Prediction) [mm^{-1}]
FS (Ground Truth) [mm^{-1}]
0.05
0.10
0.15
0.20
0.25

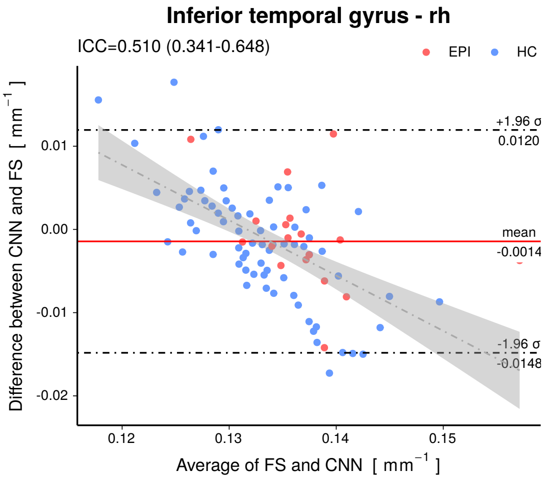
Inferior temporal gyrus - rh
ICC=0.510 (0.341-0.648)
EPI
HC
+1.96 σ
0.0120
mean
-0.0014
-1.96 σ
-0.0148
Difference between CNN and FS [mm^{-1}]
Average of FS and CNN [mm^{-1}]
0.01
0.00
-0.01
-0.02
0.12
0.13
0.14
0.15

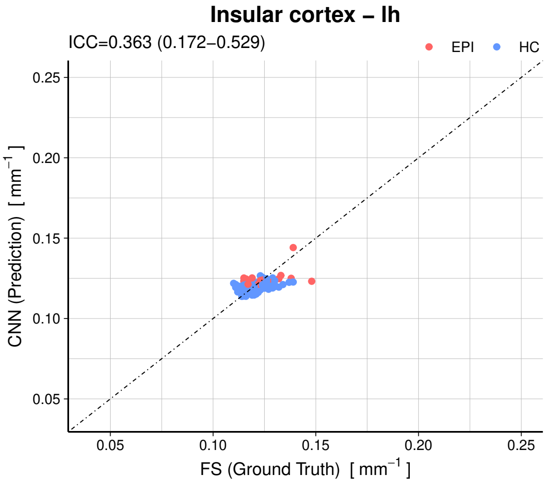
Insular cortex – lh
ICC=0.363 (0.172–0.529)
EPI
HC
CNN (Prediction) [mm^{-1}]
FS (Ground Truth) [mm^{-1}]
0.05
0.10
0.15
0.20
0.25

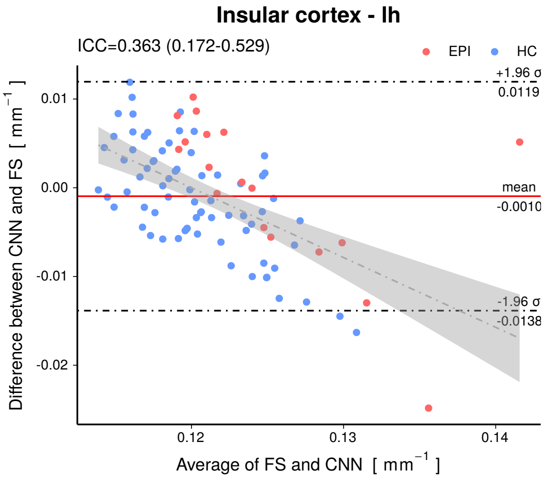
Insular cortex - lh
ICC=0.363 (0.172-0.529)
EPI
HC
+1.96 σ
0.0119
mean
-0.0010
-1.96 σ
-0.0138
Difference between CNN and FS [mm^{-1}]
Average of FS and CNN [mm^{-1}]
0.01
0.00
-0.01
-0.02
0.12
0.13
0.14

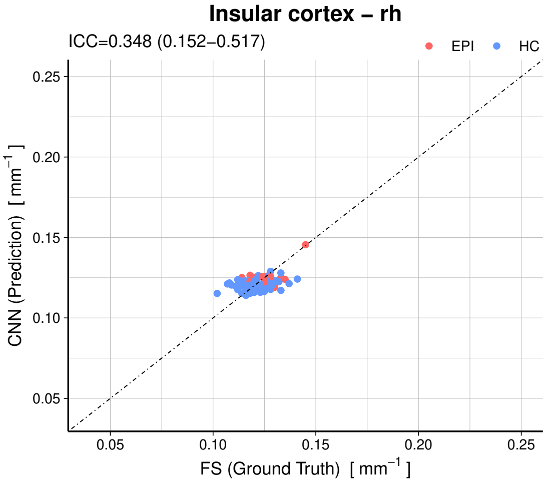
Insular cortex – rh
ICC=0.348 (0.152–0.517)
EPI
HC
CNN (Prediction) [mm^{-1}]
FS (Ground Truth) [mm^{-1}]
0.05
0.10
0.15
0.20
0.25

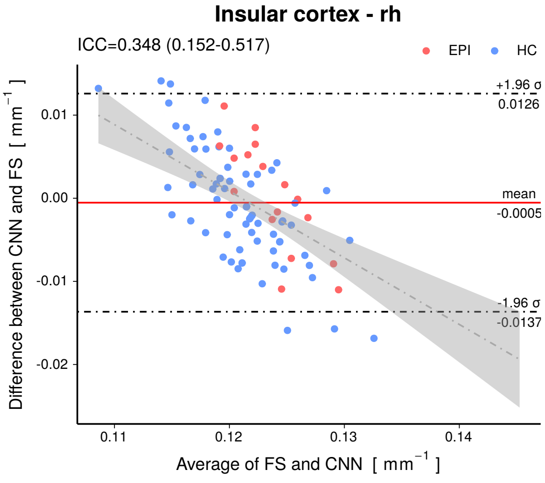
Insular cortex - rh
ICC=0.348 (0.152-0.517)
EPI
HC
+1.96 σ
0.0126
mean
-0.0005
-1.96 σ
-0.0137
Difference between CNN and FS [mm^{-1}]
Average of FS and CNN [mm^{-1}]
0.01
0.00
-0.01
-0.02
0.11
0.12
0.13
0.14

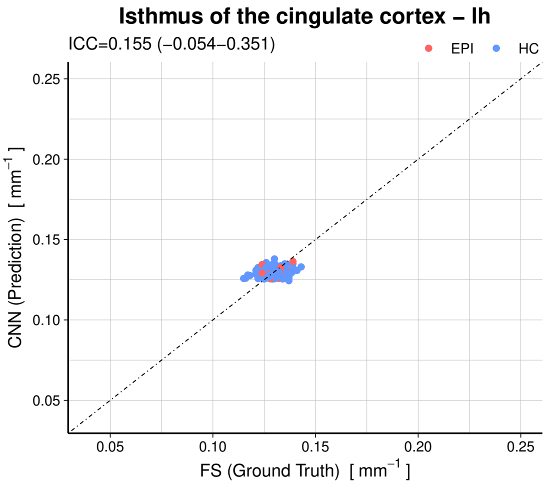
Isthmus of the cingulate cortex – lh
ICC=0.155 (–0.054–0.351)
EPI
HC
CNN (Prediction) [mm^{-1}]
FS (Ground Truth) [mm^{-1}]
0.05
0.10
0.15
0.20
0.25

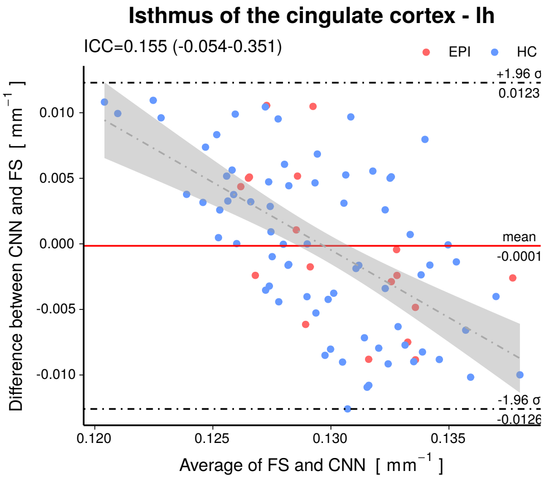
Isthmus of the cingulate cortex - lh
ICC=0.155 (-0.054-0.351)
EPI
HC
+1.96 σ
0.0123
mean
-0.0001
-1.96 σ
-0.0126
Difference between CNN and FS [mm^{-1}]
Average of FS and CNN [mm^{-1}]
0.010
0.005
0.000
-0.005
-0.010
0.120
0.125
0.130
0.135

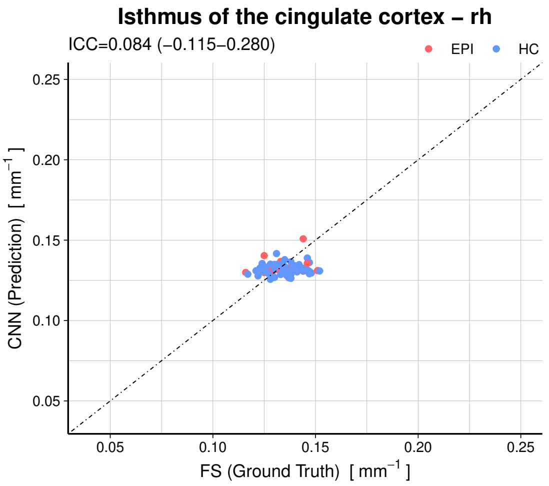
Isthmus of the cingulate cortex – rh
ICC=0.084 (–0.115–0.280)
EPI
HC
CNN (Prediction) [mm^{-1}]
FS (Ground Truth) [mm^{-1}]
0.05
0.10
0.15
0.20
0.25

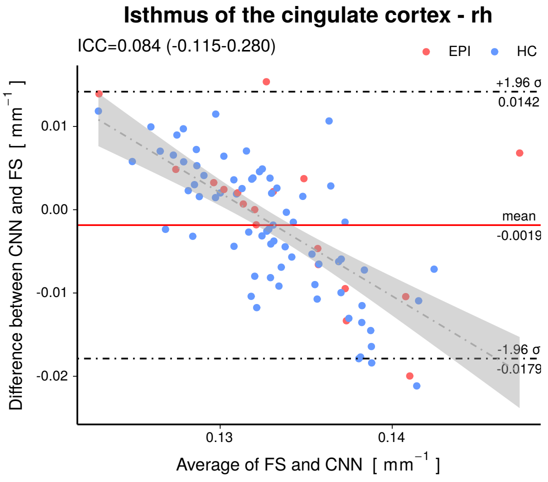
Isthmus of the cingulate cortex - rh
ICC=0.084 (-0.115-0.280)
EPI
HC
+1.96 σ
0.0142
mean
-0.0019
-1.96 σ
-0.0179
Difference between CNN and FS [mm^{-1}]
Average of FS and CNN [mm^{-1}]
0.01
0.00
-0.01
-0.02
0.13
0.14

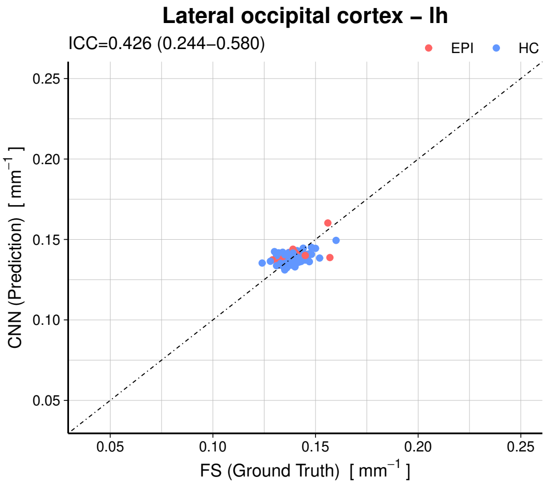
Lateral occipital cortex – lh
ICC=0.426 (0.244–0.580)
EPI
HC
CNN (Prediction) [mm^{-1}]
FS (Ground Truth) [mm^{-1}]
0.05
0.10
0.15
0.20
0.25

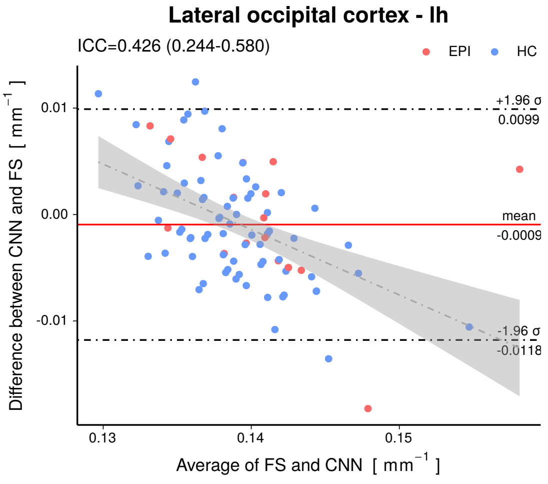
Lateral occipital cortex - lh
ICC=0.426 (0.244-0.580)
EPI
HC
+1.96 σ
0.0099
mean
-0.0009
-1.96 σ
-0.0118
Difference between CNN and FS [mm^{-1}]
Average of FS and CNN [mm^{-1}]
0.01
0.00
-0.01
0.13
0.14
0.15

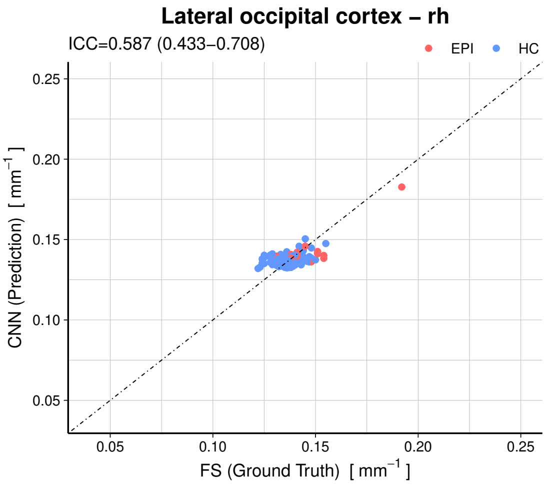
Lateral occipital cortex – rh
ICC=0.587 (0.433–0.708)
EPI
HC
CNN (Prediction) [mm^{-1}]
FS (Ground Truth) [mm^{-1}]

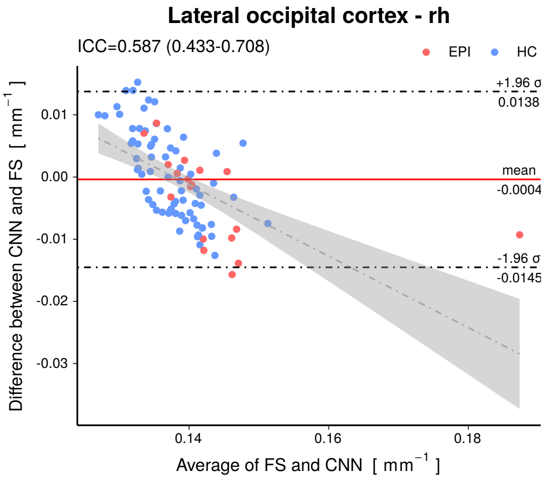
Lateral occipital cortex - rh
ICC=0.587 (0.433-0.708)
EPI
HC
+1.96 σ
0.0138
mean
-0.0004
-1.96 σ
-0.0145
Difference between CNN and FS [mm^{-1}]
Average of FS and CNN [mm^{-1}]

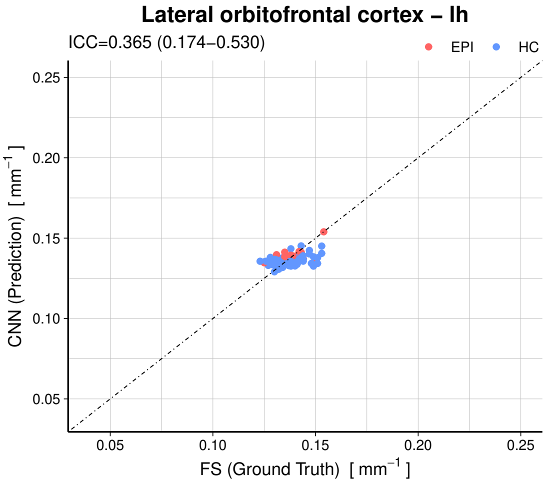
Lateral orbitofrontal cortex – lh
ICC=0.365 (0.174–0.530)
EPI
HC
CNN (Prediction) [mm^{-1}]
FS (Ground Truth) [mm^{-1}]

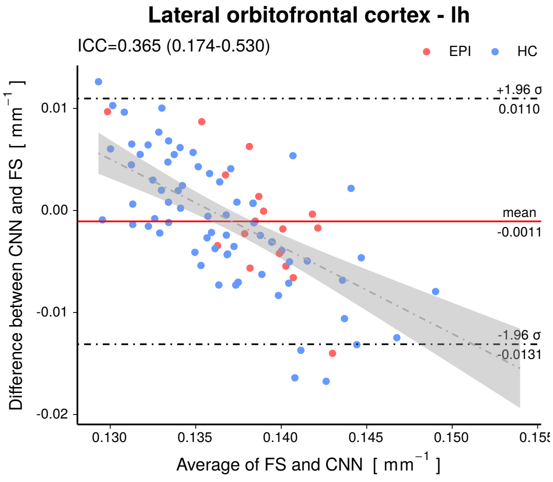
Lateral orbitofrontal cortex - lh
ICC=0.365 (0.174-0.530)
EPI
HC
+1.96 σ
0.0110
mean
-0.0011
-1.96 σ
-0.0131
Difference between CNN and FS [mm^{-1}]
Average of FS and CNN [mm^{-1}]

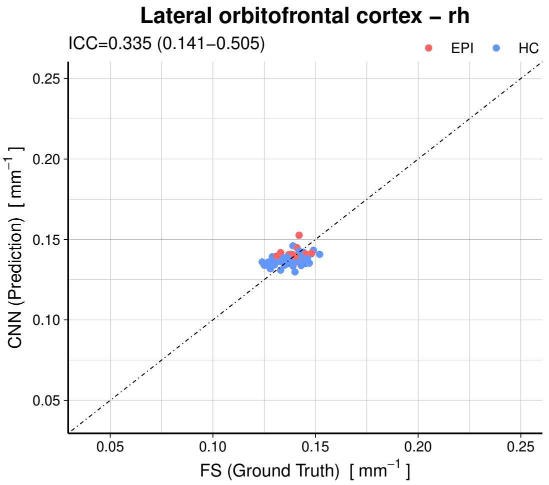
Lateral orbitofrontal cortex – rh
ICC=0.335 (0.141–0.505)
EPI
HC
CNN (Prediction) [mm^{-1}]
FS (Ground Truth) [mm^{-1}]

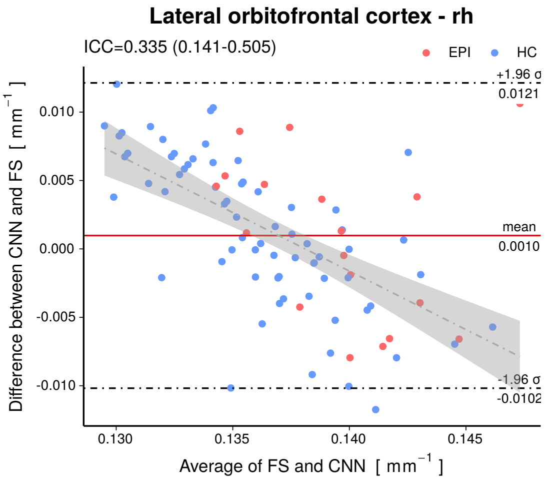
Lateral orbitofrontal cortex - rh
ICC=0.335 (0.141-0.505)
EPI
HC
+1.96 σ
0.0121
mean
0.0010
-1.96 σ
-0.0102
Difference between CNN and FS [mm^{-1}]
Average of FS and CNN [mm^{-1}]

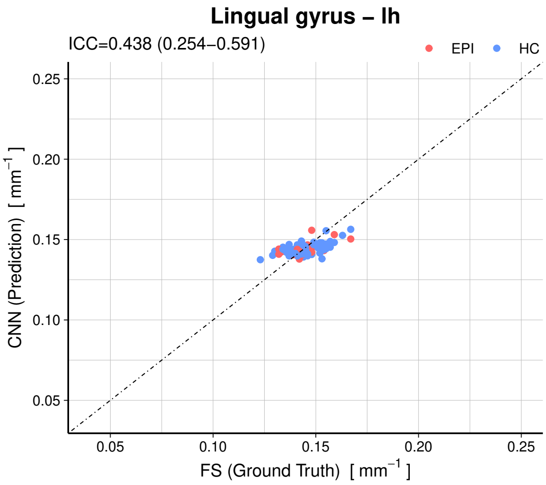
Lingual gyrus – lh
ICC=0.438 (0.254–0.591)
EPI
HC
CNN (Prediction) [mm^{-1}]
FS (Ground Truth) [mm^{-1}]

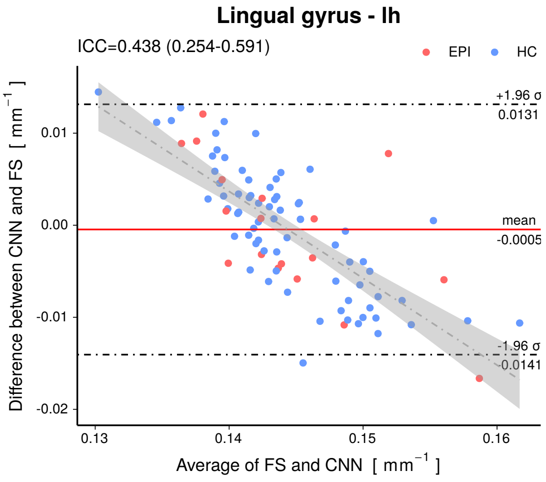
Lingual gyrus - lh
ICC=0.438 (0.254-0.591)
EPI
HC
+1.96 σ
0.0131
mean
-0.0005
-1.96 σ
-0.0141
Difference between CNN and FS [mm^{-1}]
Average of FS and CNN [mm^{-1}]

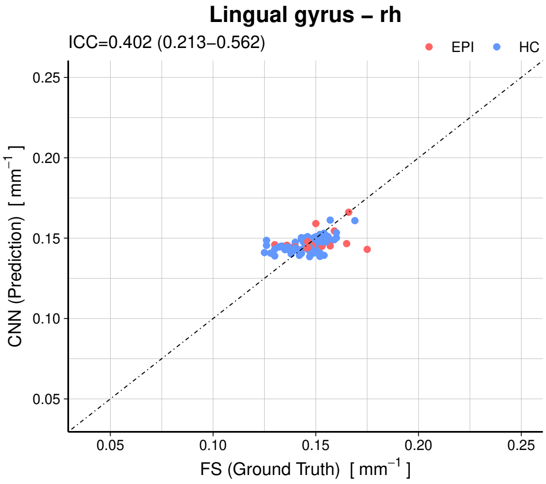
Lingual gyrus – rh
ICC=0.402 (0.213–0.562)
EPI
HC
CNN (Prediction) [mm^{-1}]
FS (Ground Truth) [mm^{-1}]

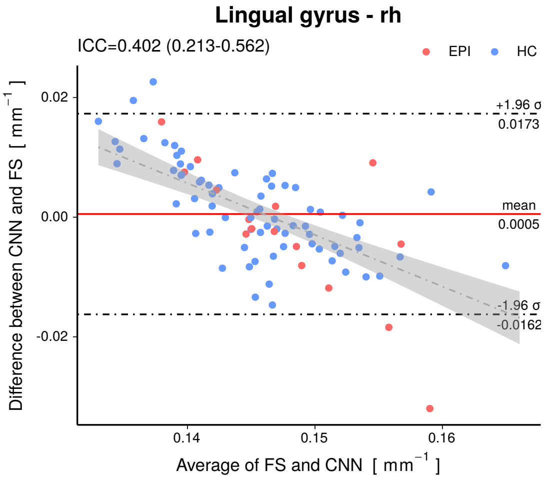
Lingual gyrus - rh
ICC=0.402 (0.213-0.562)
EPI
HC
+1.96 σ
0.0173
mean
0.0005
-1.96 σ
-0.0162
Difference between CNN and FS [mm^{-1}]
Average of FS and CNN [mm^{-1}]

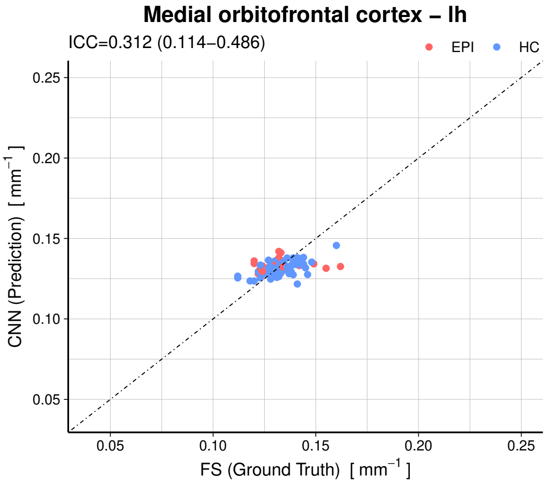
Medial orbitofrontal cortex – lh
ICC=0.312 (0.114–0.486)
EPI
HC
CNN (Prediction) [mm^{-1}]
FS (Ground Truth) [mm^{-1}]

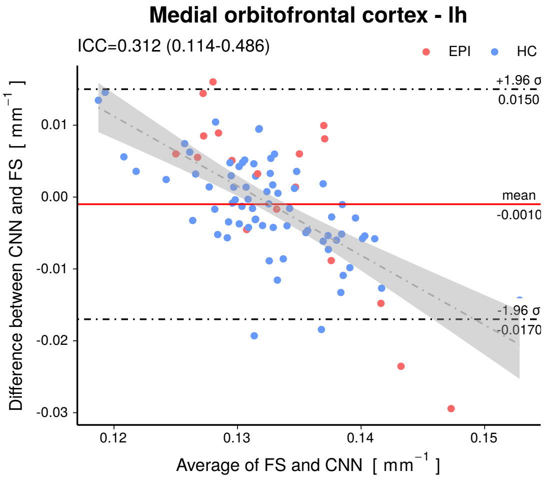
Medial orbitofrontal cortex - lh
ICC=0.312 (0.114-0.486)
EPI
HC
+1.96 σ
0.0150
mean
-0.0010
-1.96 σ
-0.0170
Difference between CNN and FS [mm^{-1}]
Average of FS and CNN [mm^{-1}]

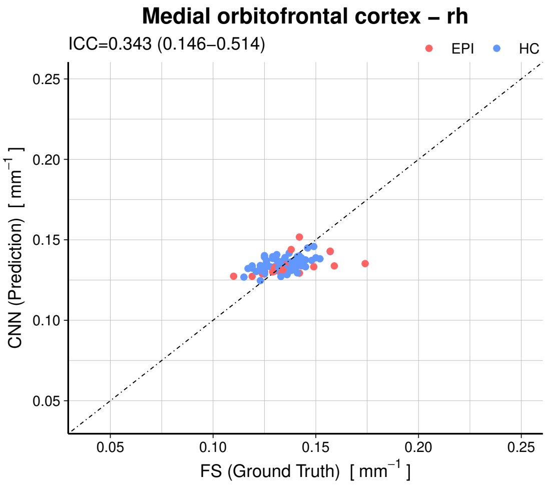
Medial orbitofrontal cortex – rh
ICC=0.343 (0.146–0.514)
EPI
HC
CNN (Prediction) [mm^{-1}]
FS (Ground Truth) [mm^{-1}]
0.05
0.10
0.15
0.20
0.25

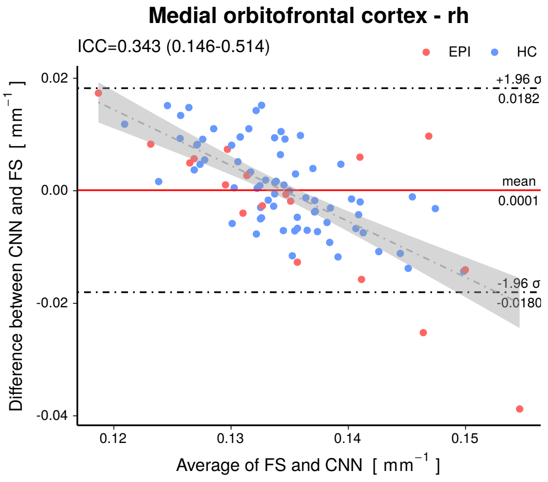
Medial orbitofrontal cortex - rh
ICC=0.343 (0.146-0.514)
EPI
HC
+1.96 σ
0.0182
mean
0.0001
-1.96 σ
-0.0180
Difference between CNN and FS [mm^{-1}]
Average of FS and CNN [mm^{-1}]
0.02
0.00
-0.02
-0.04
0.12
0.13
0.14
0.15

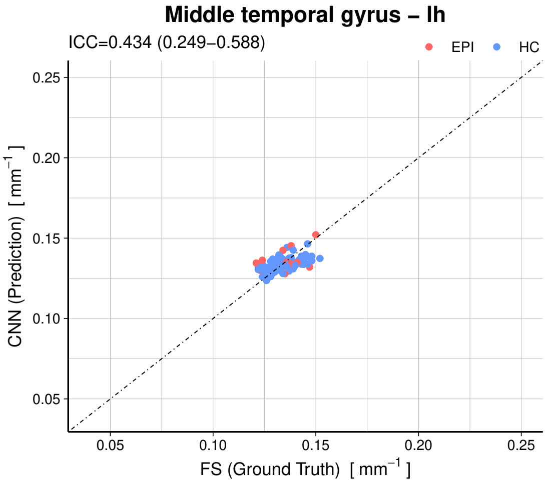
Middle temporal gyrus – lh
ICC=0.434 (0.249–0.588)
EPI
HC
CNN (Prediction) [mm^{-1}]
FS (Ground Truth) [mm^{-1}]
0.05
0.10
0.15
0.20
0.25

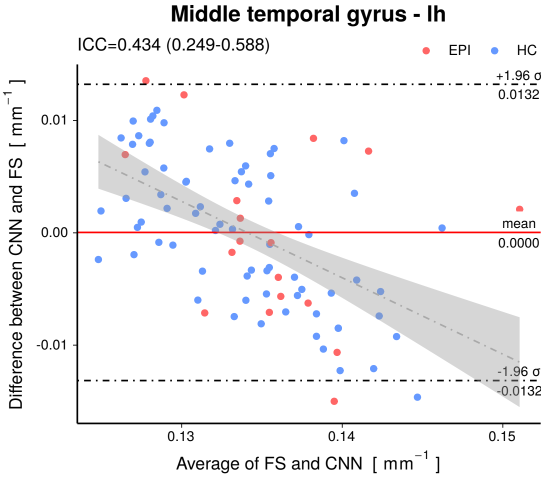
Middle temporal gyrus - lh
ICC=0.434 (0.249-0.588)
EPI
HC
+1.96 σ
0.0132
mean
0.0000
-1.96 σ
-0.0132
Difference between CNN and FS [mm^{-1}]
Average of FS and CNN [mm^{-1}]
0.01
0.00
-0.01
0.13
0.14
0.15

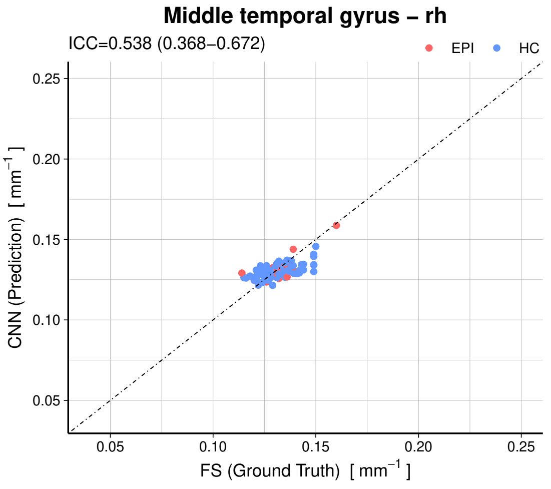
Middle temporal gyrus – rh
ICC=0.538 (0.368–0.672)
EPI
HC
CNN (Prediction) [mm^{-1}]
FS (Ground Truth) [mm^{-1}]
0.05
0.10
0.15
0.20
0.25

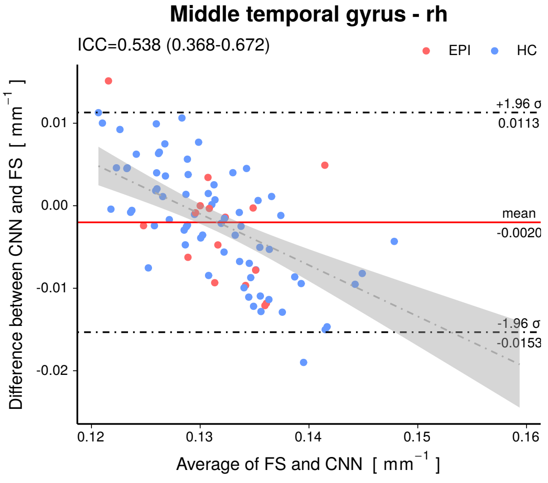
Middle temporal gyrus - rh
ICC=0.538 (0.368-0.672)
EPI
HC
+1.96 σ
0.0113
mean
-0.0020
-1.96 σ
-0.0153
Difference between CNN and FS [mm^{-1}]
Average of FS and CNN [mm^{-1}]
0.01
0.00
-0.01
-0.02
0.12
0.13
0.14
0.15
0.16

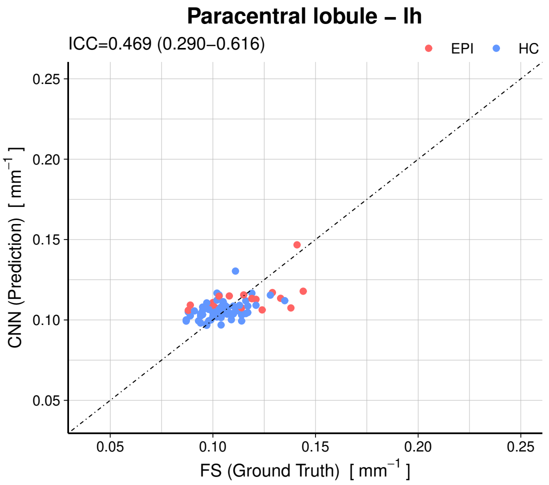
Paracentral lobule – lh
ICC=0.469 (0.290–0.616)
EPI
HC
CNN (Prediction) [mm^{-1}]
FS (Ground Truth) [mm^{-1}]
0.05
0.10
0.15
0.20
0.25

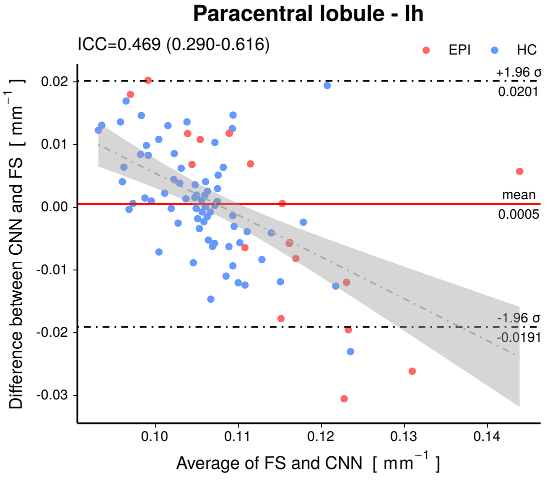
Paracentral lobule - lh
ICC=0.469 (0.290-0.616)
EPI
HC
+1.96 σ
0.0201
mean
0.0005
-1.96 σ
-0.0191
Difference between CNN and FS [mm^{-1}]
Average of FS and CNN [mm^{-1}]

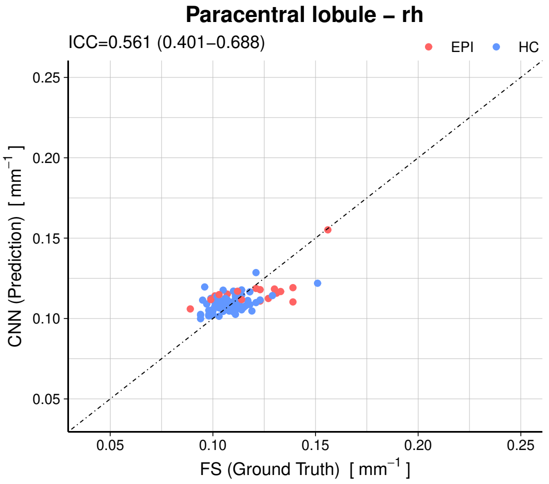
Paracentral lobule – rh
ICC=0.561 (0.401–0.688)
EPI
HC
CNN (Prediction) [mm^{-1}]
FS (Ground Truth) [mm^{-1}]

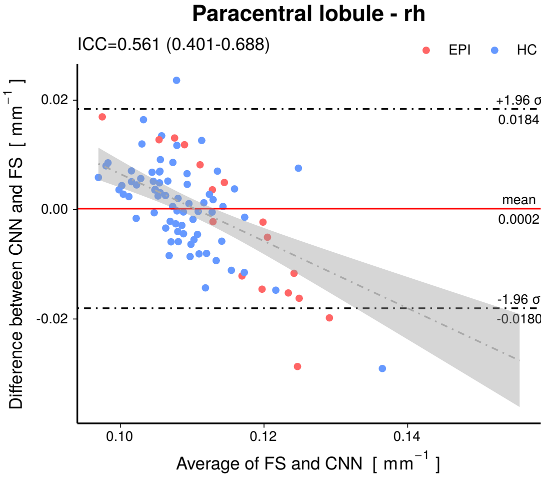
Paracentral lobule - rh
ICC=0.561 (0.401-0.688)
EPI
HC
+1.96 σ
0.0184
mean
0.0002
-1.96 σ
-0.0180
Difference between CNN and FS [mm^{-1}]
Average of FS and CNN [mm^{-1}]

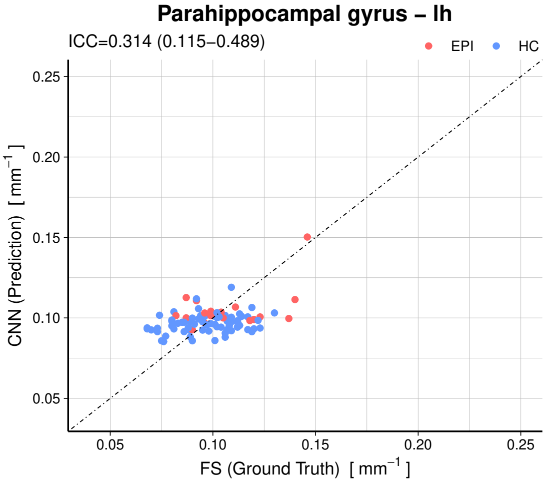
Parahippocampal gyrus – lh
ICC=0.314 (0.115–0.489)
EPI
HC
CNN (Prediction) [mm^{-1}]
FS (Ground Truth) [mm^{-1}]

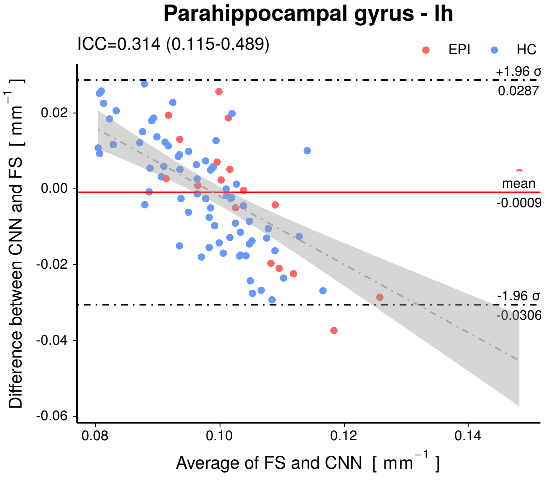
Parahippocampal gyrus - lh
ICC=0.314 (0.115-0.489)
EPI
HC
+1.96 σ
0.0287
mean
-0.0009
-1.96 σ
-0.0306
Difference between CNN and FS [mm^{-1}]
Average of FS and CNN [mm^{-1}]

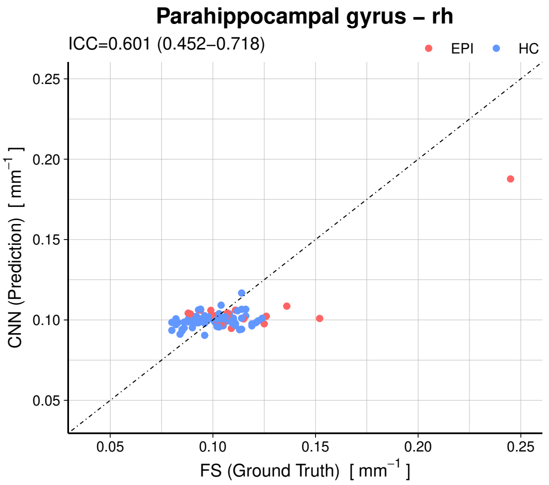
Parahippocampal gyrus – rh
ICC=0.601 (0.452–0.718)
EPI
HC
CNN (Prediction) [mm^-1]
FS (Ground Truth) [mm^-1]
0.05
0.10
0.15
0.20
0.25

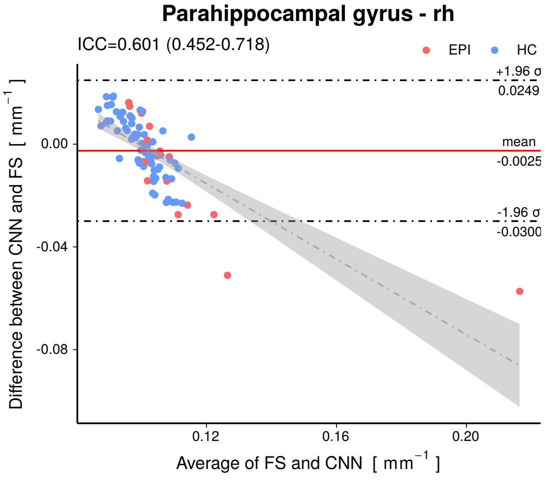
Parahippocampal gyrus - rh
ICC=0.601 (0.452-0.718)
EPI
HC
+1.96 σ
0.0249
mean
-0.0025
-1.96 σ
-0.0300
Difference between CNN and FS [mm^-1]
Average of FS and CNN [mm^-1]
0.00
-0.04
-0.08
0.12
0.16
0.20

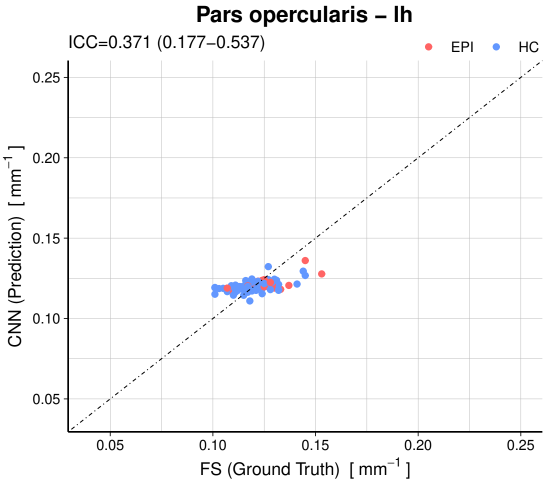
Pars opercularis – lh
ICC=0.371 (0.177–0.537)
EPI
HC
CNN (Prediction) [mm^-1]
FS (Ground Truth) [mm^-1]
0.05
0.10
0.15
0.20
0.25

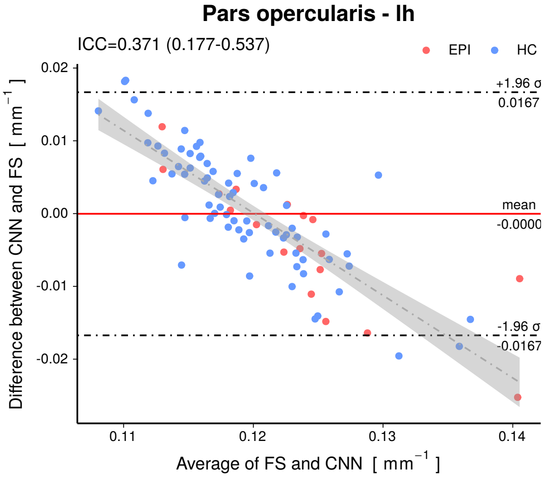
Pars opercularis - lh
ICC=0.371 (0.177-0.537)
EPI
HC
+1.96 σ
0.0167
mean
-0.0000
-1.96 σ
-0.0167
Difference between CNN and FS [mm^-1]
Average of FS and CNN [mm^-1]
0.02
0.01
0.00
-0.01
-0.02
0.11
0.12
0.13
0.14

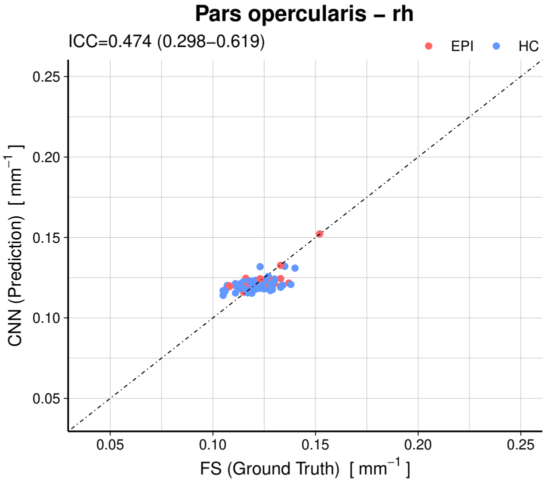
Pars opercularis – rh
ICC=0.474 (0.298–0.619)
EPI
HC
CNN (Prediction) [mm^-1]
FS (Ground Truth) [mm^-1]
0.05
0.10
0.15
0.20
0.25

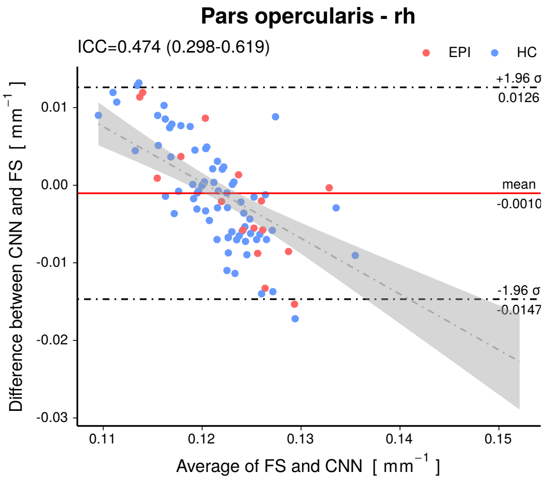
Pars opercularis - rh
ICC=0.474 (0.298-0.619)
EPI
HC
+1.96 σ
0.0126
mean
-0.0010
-1.96 σ
-0.0147
Difference between CNN and FS [mm^-1]
Average of FS and CNN [mm^-1]
0.01
0.00
-0.01
-0.02
-0.03
0.11
0.12
0.13
0.14
0.15

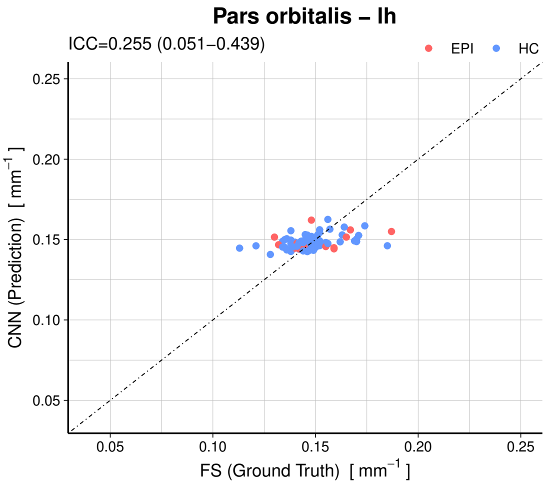
Pars orbitalis – lh
ICC=0.255 (0.051–0.439)
EPI
HC
CNN (Prediction) [mm^{-1}]
FS (Ground Truth) [mm^{-1}]

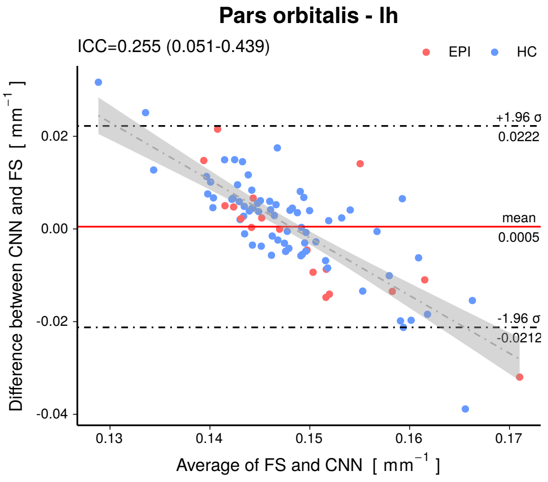
Pars orbitalis - lh
ICC=0.255 (0.051-0.439)
EPI
HC
+1.96 σ
0.0222
mean
0.0005
-1.96 σ
-0.0212
Difference between CNN and FS [mm^{-1}]
Average of FS and CNN [mm^{-1}]

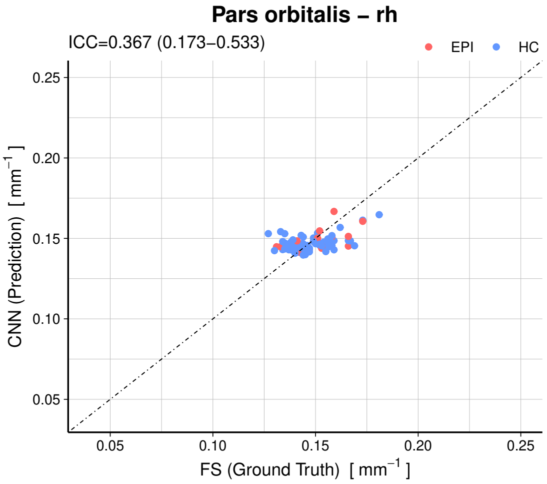
Pars orbitalis – rh
ICC=0.367 (0.173–0.533)
EPI
HC
CNN (Prediction) [mm^{-1}]
FS (Ground Truth) [mm^{-1}]

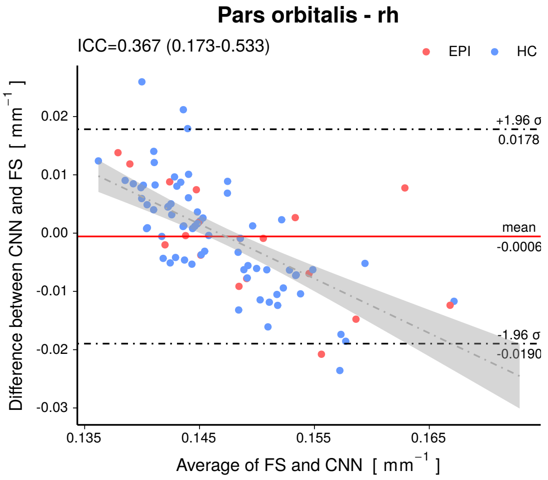
Pars orbitalis - rh
ICC=0.367 (0.173-0.533)
EPI
HC
+1.96 σ
0.0178
mean
-0.0006
-1.96 σ
-0.0190
Difference between CNN and FS [mm^{-1}]
Average of FS and CNN [mm^{-1}]

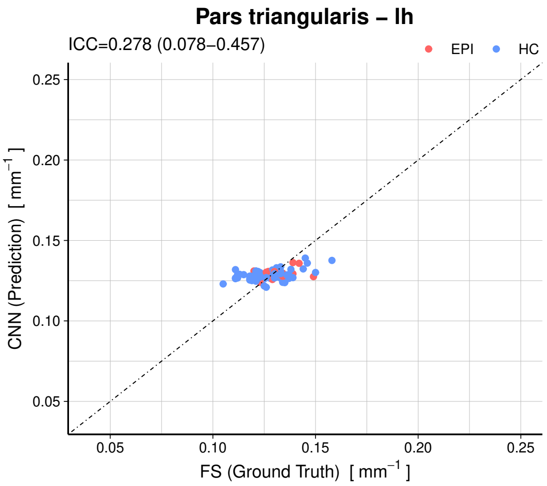
Pars triangularis – lh
ICC=0.278 (0.078–0.457)
EPI
HC
CNN (Prediction) [mm^{-1}]
FS (Ground Truth) [mm^{-1}]

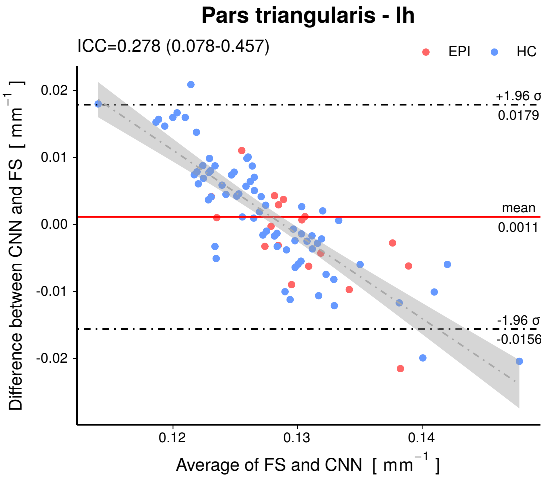
Pars triangularis - lh
ICC=0.278 (0.078-0.457)
EPI
HC
+1.96 σ
0.0179
mean
0.0011
-1.96 σ
-0.0156
Difference between CNN and FS [mm^{-1}]
Average of FS and CNN [mm^{-1}]

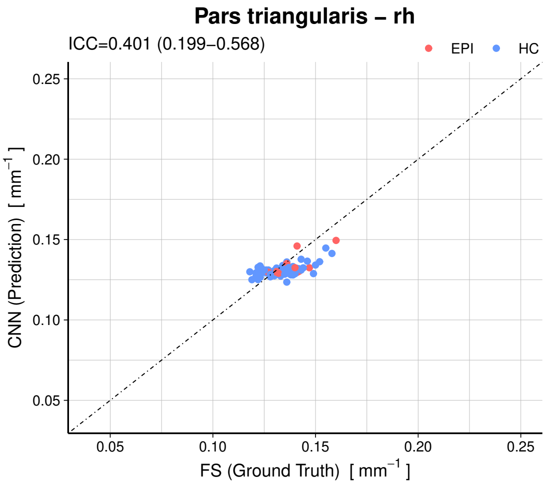
Pars triangularis – rh
ICC=0.401 (0.199–0.568)
EPI
HC
CNN (Prediction) [mm^{-1}]
FS (Ground Truth) [mm^{-1}]
0.05
0.10
0.15
0.20
0.25

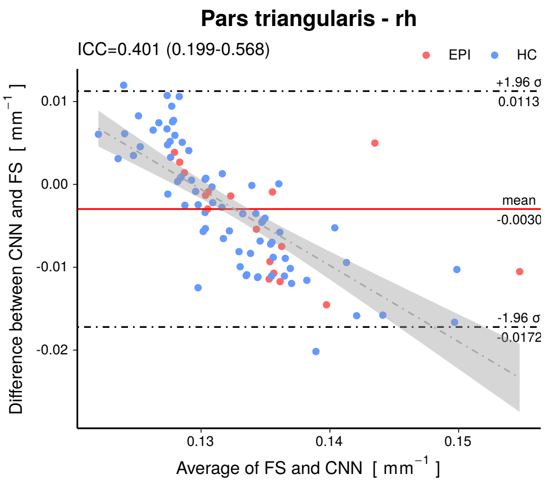
Pars triangularis - rh
ICC=0.401 (0.199-0.568)
EPI
HC
+1.96 σ
0.0113
mean
-0.0030
-1.96 σ
-0.0172
Difference between CNN and FS [mm^{-1}]
Average of FS and CNN [mm^{-1}]
0.01
0.00
-0.01
-0.02
0.13
0.14
0.15

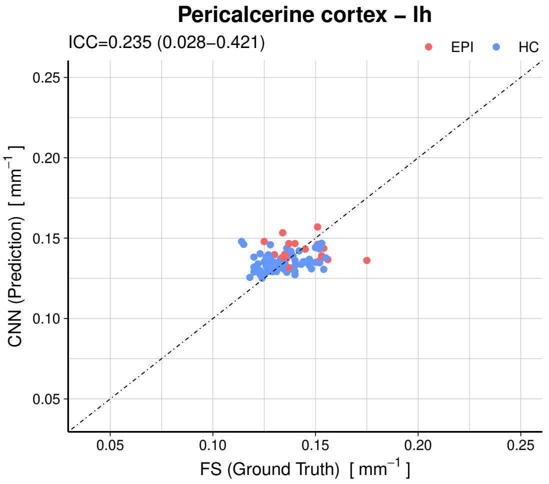
Pericalcerine cortex – lh
ICC=0.235 (0.028–0.421)
EPI
HC
CNN (Prediction) [mm^{-1}]
FS (Ground Truth) [mm^{-1}]
0.05
0.10
0.15
0.20
0.25

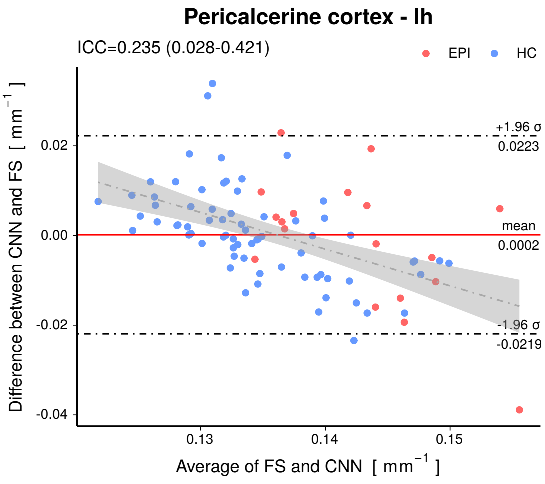
Pericalcerine cortex - lh
ICC=0.235 (0.028-0.421)
EPI
HC
+1.96 σ
0.0223
mean
0.0002
-1.96 σ
-0.0219
Difference between CNN and FS [mm^{-1}]
Average of FS and CNN [mm^{-1}]
0.02
0.00
-0.02
-0.04
0.13
0.14
0.15

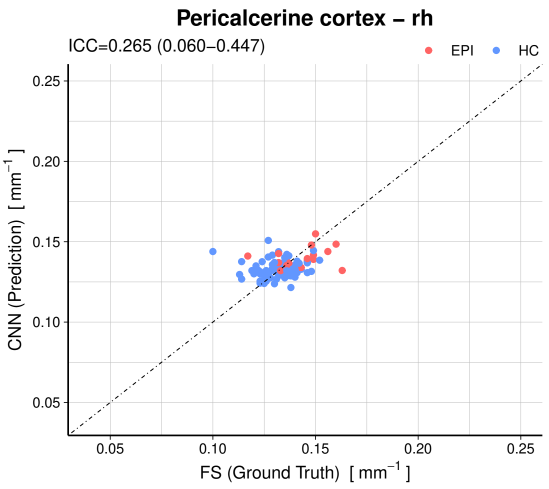
Pericalcerine cortex – rh
ICC=0.265 (0.060–0.447)
EPI
HC
CNN (Prediction) [mm^{-1}]
FS (Ground Truth) [mm^{-1}]
0.05
0.10
0.15
0.20
0.25

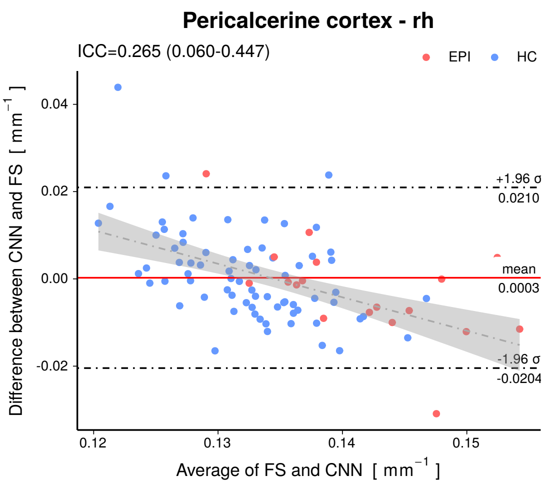
Pericalcerine cortex - rh
ICC=0.265 (0.060-0.447)
EPI
HC
+1.96 σ
0.0210
mean
0.0003
-1.96 σ
-0.0204
Difference between CNN and FS [mm^{-1}]
Average of FS and CNN [mm^{-1}]
0.04
0.02
0.00
-0.02
0.12
0.13
0.14
0.15

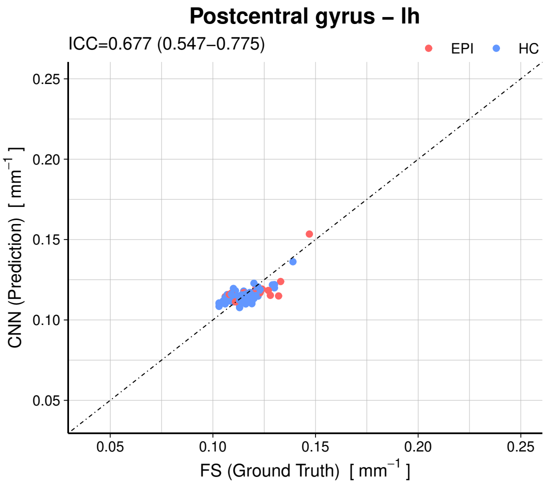
Postcentral gyrus – lh
ICC=0.677 (0.547–0.775)
EPI
HC
CNN (Prediction) [mm^{-1}]
FS (Ground Truth) [mm^{-1}]

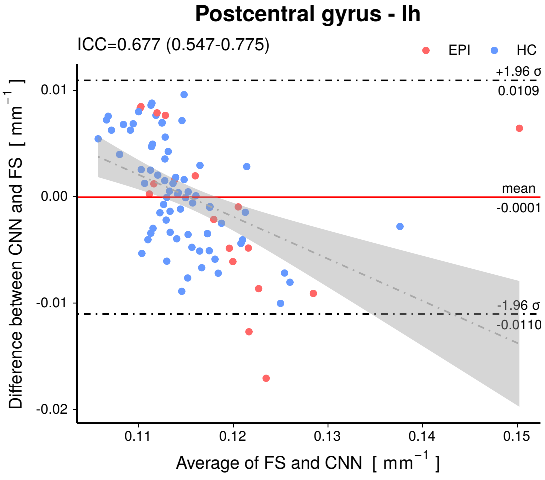
Postcentral gyrus - lh
ICC=0.677 (0.547-0.775)
EPI
HC
+1.96 σ
0.0109
mean
-0.0001
-1.96 σ
-0.0110
Difference between CNN and FS [mm^{-1}]
Average of FS and CNN [mm^{-1}]

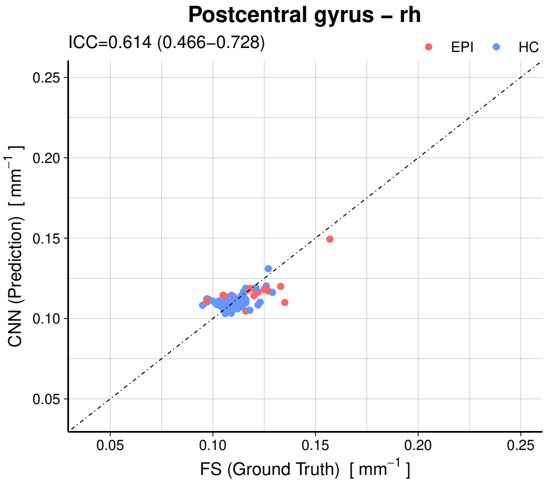
Postcentral gyrus – rh
ICC=0.614 (0.466–0.728)
EPI
HC
CNN (Prediction) [mm^{-1}]
FS (Ground Truth) [mm^{-1}]

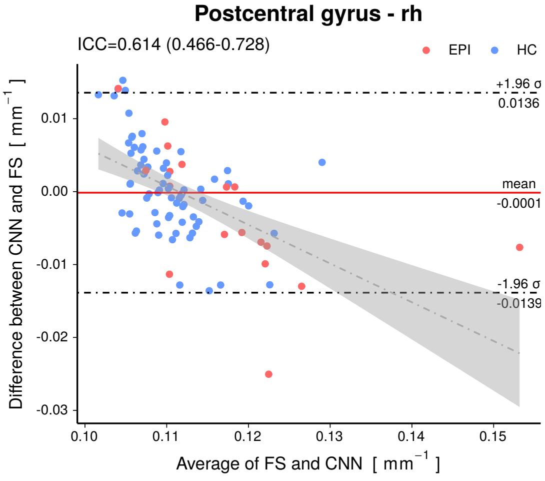
Postcentral gyrus - rh
ICC=0.614 (0.466-0.728)
EPI
HC
+1.96 σ
0.0136
mean
-0.0001
-1.96 σ
-0.0139
Difference between CNN and FS [mm^{-1}]
Average of FS and CNN [mm^{-1}]

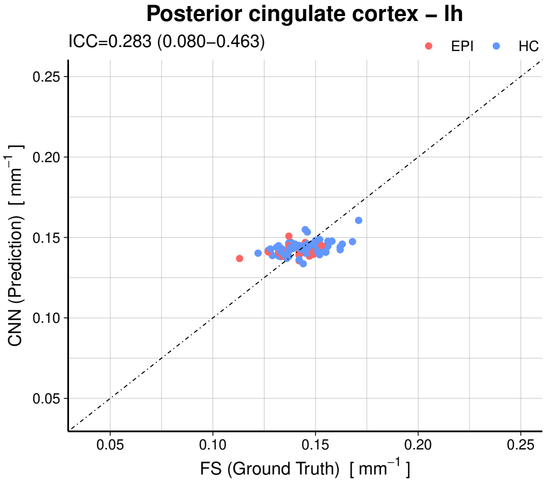
Posterior cingulate cortex – lh
ICC=0.283 (0.080–0.463)
EPI
HC
CNN (Prediction) [mm^{-1}]
FS (Ground Truth) [mm^{-1}]

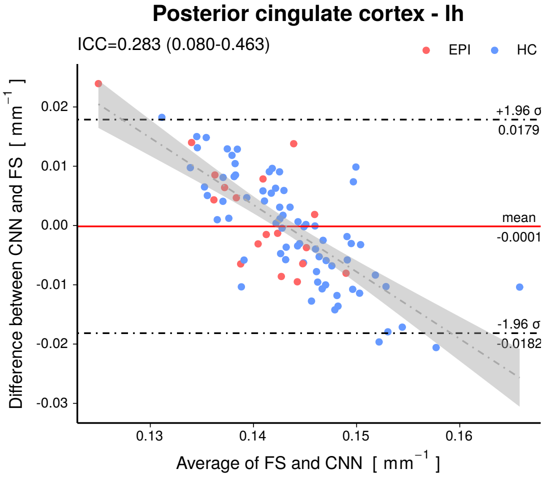
Posterior cingulate cortex - lh
ICC=0.283 (0.080-0.463)
EPI
HC
+1.96 σ
0.0179
mean
-0.0001
-1.96 σ
-0.0182
Difference between CNN and FS [mm^{-1}]
Average of FS and CNN [mm^{-1}]

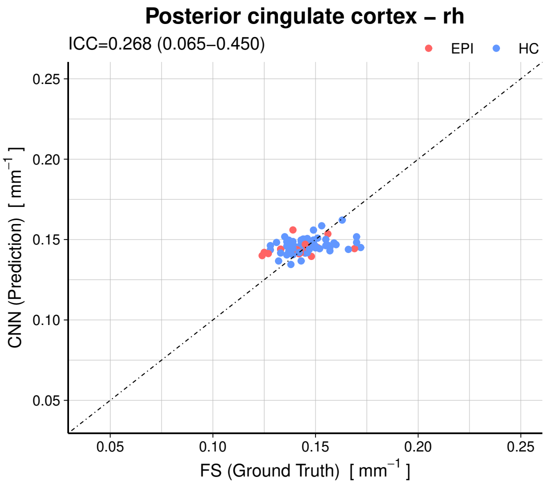
Posterior cingulate cortex – rh
ICC=0.268 (0.065–0.450)
EPI
HC
CNN (Prediction) [mm−1]
FS (Ground Truth) [mm−1]

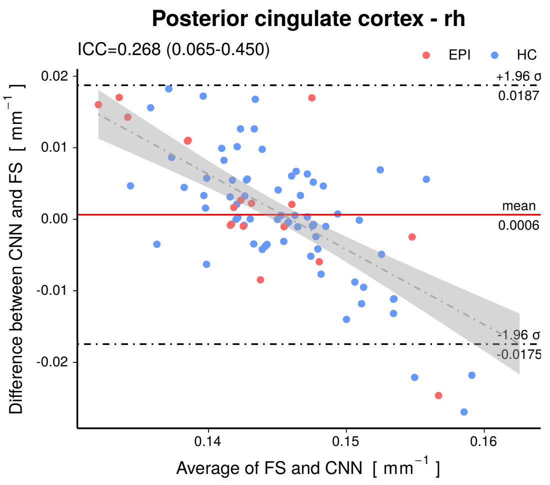
Posterior cingulate cortex - rh
ICC=0.268 (0.065-0.450)
EPI
HC
+1.96 σ
0.0187
mean
0.0006
-1.96 σ
-0.0175
Difference between CNN and FS [mm−1]
Average of FS and CNN [mm−1]

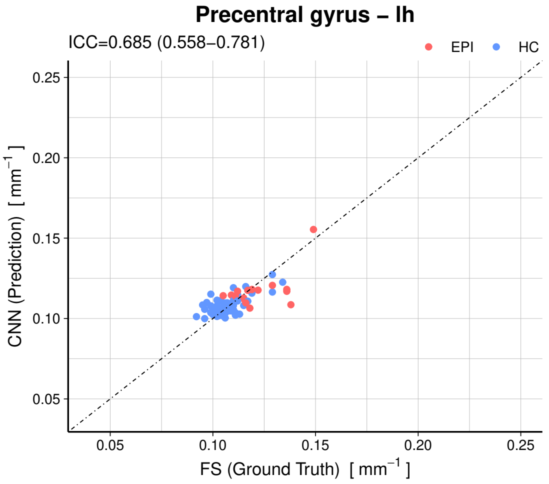
Precentral gyrus – lh
ICC=0.685 (0.558–0.781)
EPI
HC
CNN (Prediction) [mm−1]
FS (Ground Truth) [mm−1]

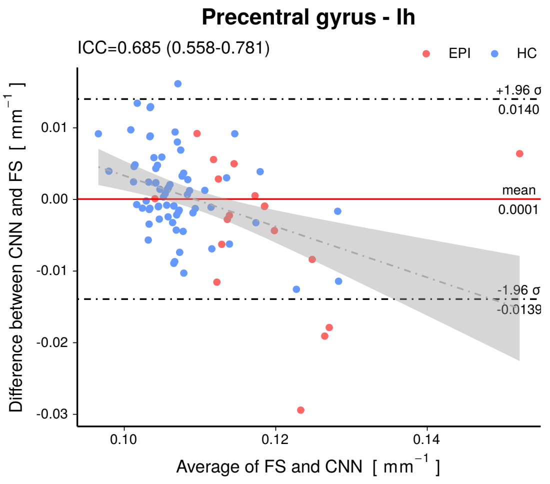
Precentral gyrus - lh
ICC=0.685 (0.558-0.781)
EPI
HC
+1.96 σ
0.0140
mean
0.0001
-1.96 σ
-0.0139
Difference between CNN and FS [mm−1]
Average of FS and CNN [mm−1]

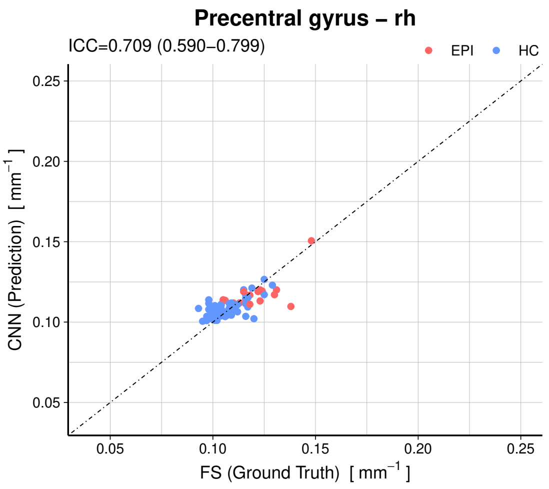
Precentral gyrus – rh
ICC=0.709 (0.590–0.799)
EPI
HC
CNN (Prediction) [mm−1]
FS (Ground Truth) [mm−1]

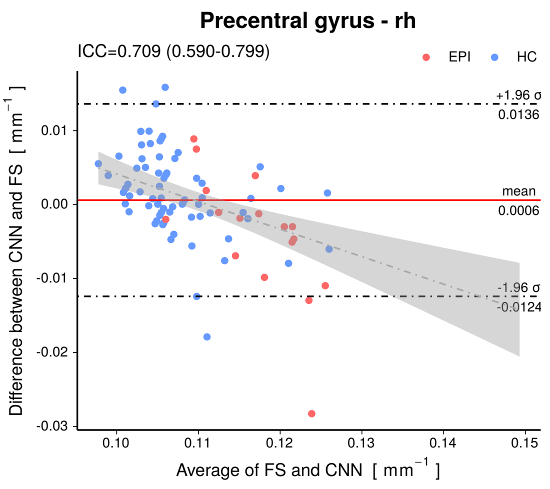
Precentral gyrus - rh
ICC=0.709 (0.590-0.799)
EPI
HC
+1.96 σ
0.0136
mean
0.0006
-1.96 σ
-0.0124
Difference between CNN and FS [mm−1]
Average of FS and CNN [mm−1]

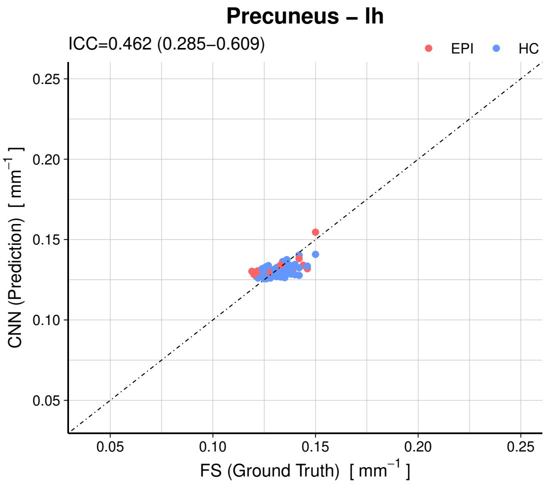
Precuneus – lh
ICC=0.462 (0.285–0.609)
EPI
HC
CNN (Prediction) [mm^{-1}]
FS (Ground Truth) [mm^{-1}]

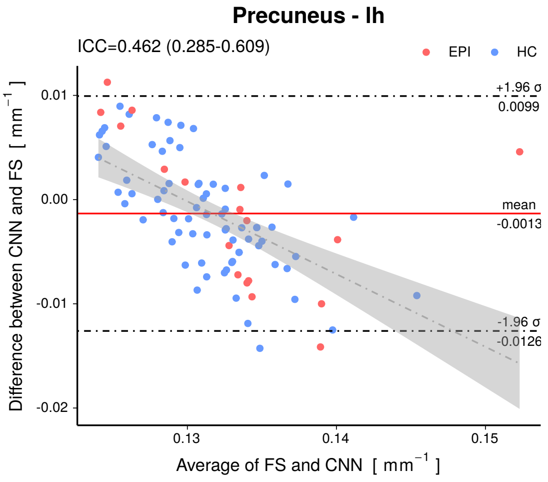
Precuneus - lh
ICC=0.462 (0.285-0.609)
EPI
HC
+1.96 σ
0.0099
mean
-0.0013
-1.96 σ
-0.0126
Difference between CNN and FS [mm^{-1}]
Average of FS and CNN [mm^{-1}]

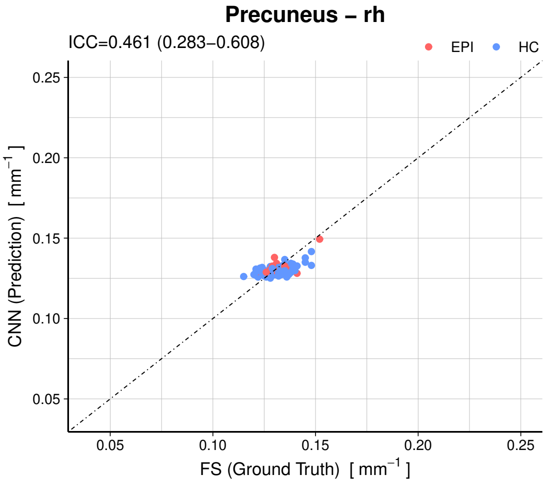
Precuneus – rh
ICC=0.461 (0.283–0.608)
EPI
HC
CNN (Prediction) [mm^{-1}]
FS (Ground Truth) [mm^{-1}]

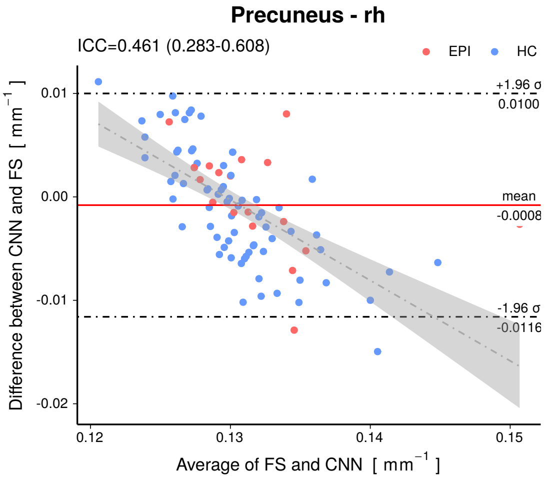
Precuneus - rh
ICC=0.461 (0.283-0.608)
EPI
HC
+1.96 σ
0.0100
mean
-0.0008
-1.96 σ
-0.0116
Difference between CNN and FS [mm^{-1}]
Average of FS and CNN [mm^{-1}]

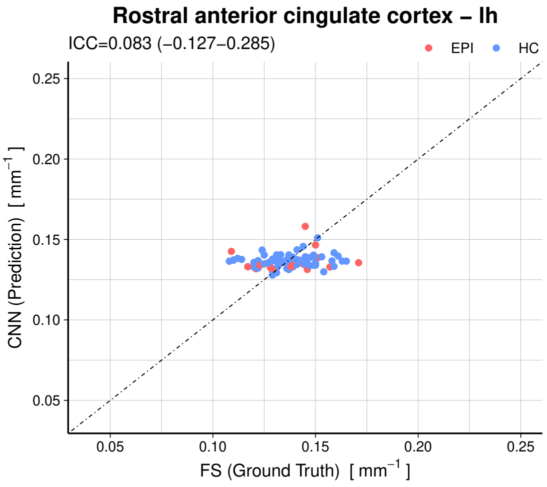
Rostral anterior cingulate cortex – lh
ICC=0.083 (–0.127–0.285)
EPI
HC
CNN (Prediction) [mm^{-1}]
FS (Ground Truth) [mm^{-1}]

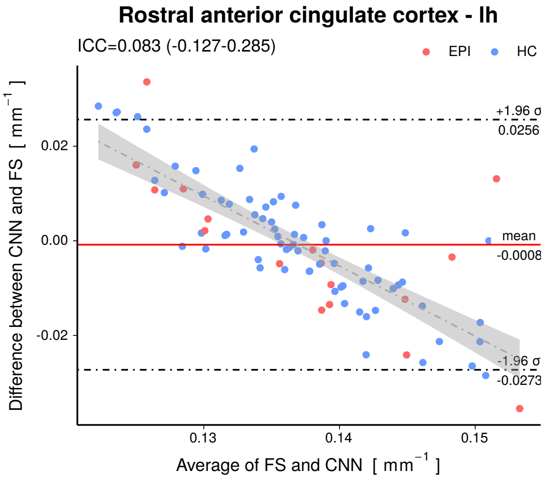
Rostral anterior cingulate cortex - lh
ICC=0.083 (-0.127-0.285)
EPI
HC
+1.96 σ
0.0256
mean
-0.0008
-1.96 σ
-0.0273
Difference between CNN and FS [mm^{-1}]
Average of FS and CNN [mm^{-1}]

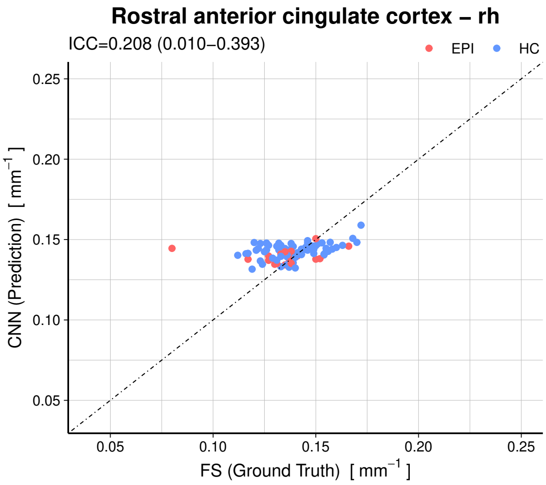
Rostral anterior cingulate cortex – rh
ICC=0.208 (0.010–0.393)
EPI
HC
CNN (Prediction) [mm^{-1}]
FS (Ground Truth) [mm^{-1}]
0.05
0.10
0.15
0.20
0.25

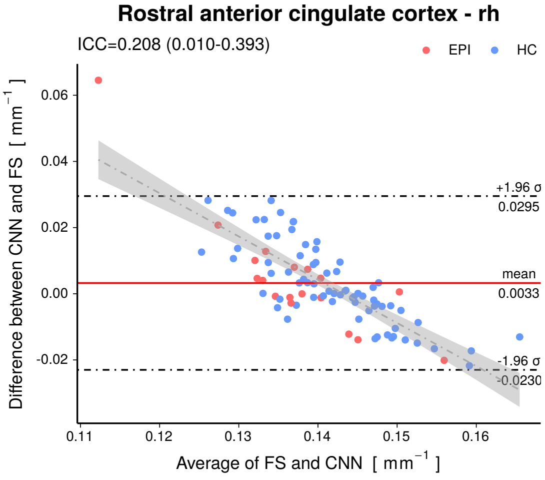
Rostral anterior cingulate cortex - rh
ICC=0.208 (0.010-0.393)
EPI
HC
Difference between CNN and FS [mm^{-1}]
Average of FS and CNN [mm^{-1}]
+1.96 σ
0.0295
mean
0.0033
-1.96 σ
-0.0230

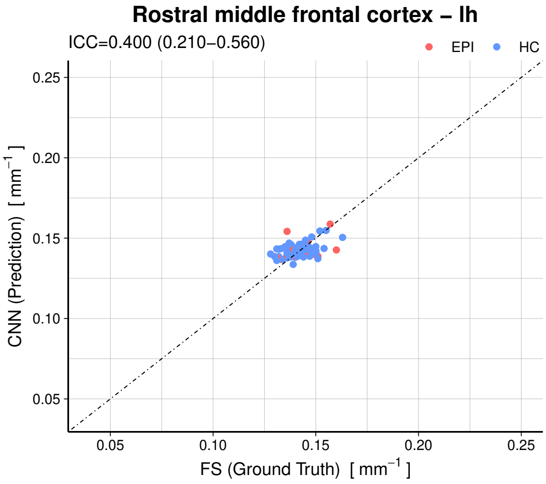
Rostral middle frontal cortex – lh
ICC=0.400 (0.210–0.560)
EPI
HC
CNN (Prediction) [mm^{-1}]
FS (Ground Truth) [mm^{-1}]

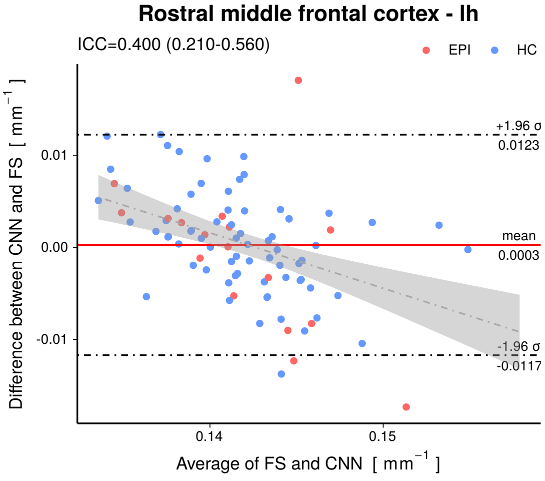
Rostral middle frontal cortex - lh
ICC=0.400 (0.210-0.560)
EPI
HC
Difference between CNN and FS [mm^{-1}]
Average of FS and CNN [mm^{-1}]
+1.96 σ
0.0123
mean
0.0003
-1.96 σ
-0.0117

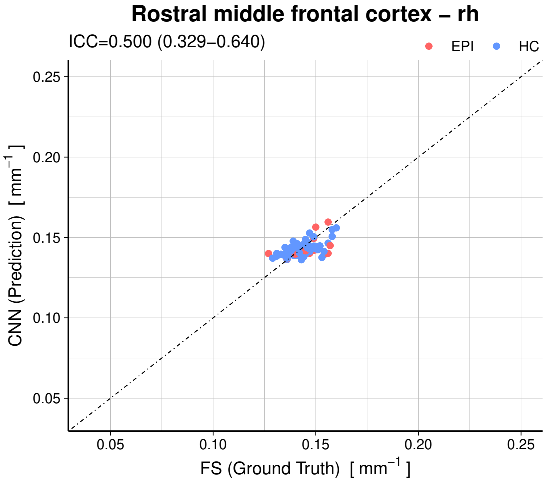
Rostral middle frontal cortex – rh
ICC=0.500 (0.329–0.640)
EPI
HC
CNN (Prediction) [mm^{-1}]
FS (Ground Truth) [mm^{-1}]

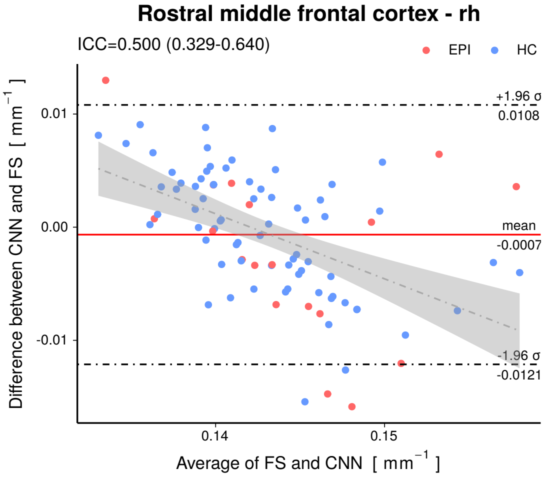
Rostral middle frontal cortex - rh
ICC=0.500 (0.329-0.640)
EPI
HC
Difference between CNN and FS [mm^{-1}]
Average of FS and CNN [mm^{-1}]
+1.96 σ
0.0108
mean
-0.0007
-1.96 σ
-0.0121

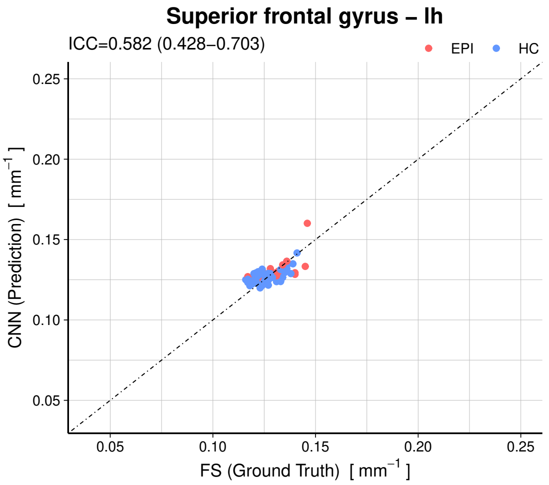
Superior frontal gyrus – lh
ICC=0.582 (0.428–0.703)
EPI
HC
CNN (Prediction) [mm^{-1}]
FS (Ground Truth) [mm^{-1}]

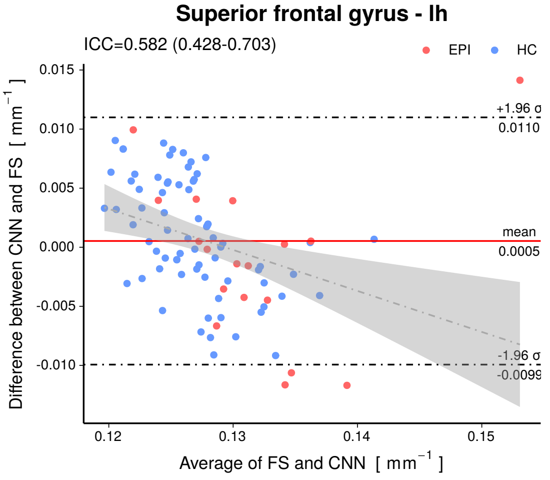
Superior frontal gyrus - lh
ICC=0.582 (0.428-0.703)
EPI
HC
+1.96 σ
0.0110
mean
0.0005
-1.96 σ
-0.0099
Difference between CNN and FS [mm^{-1}]
Average of FS and CNN [mm^{-1}]

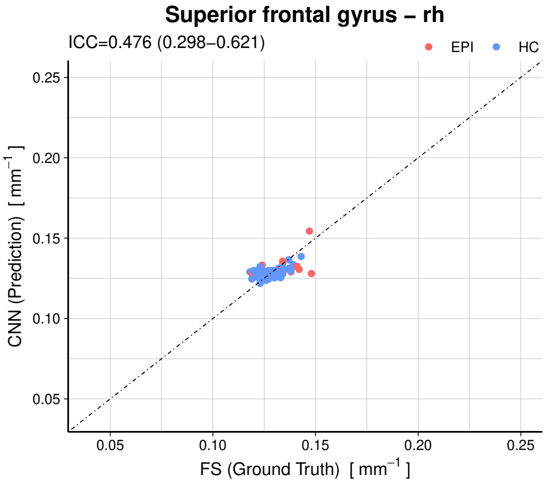
Superior frontal gyrus – rh
ICC=0.476 (0.298–0.621)
EPI
HC
CNN (Prediction) [mm^{-1}]
FS (Ground Truth) [mm^{-1}]

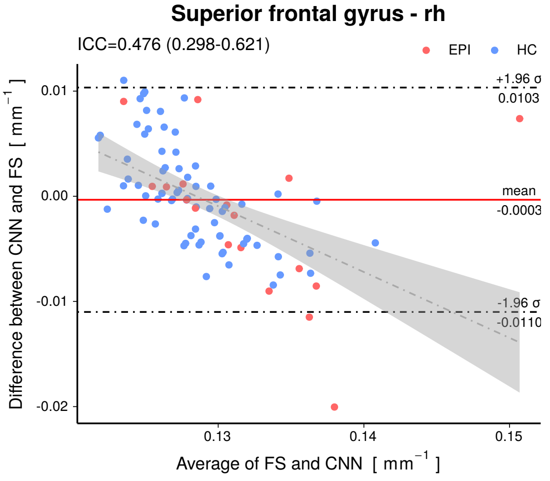
Superior frontal gyrus - rh
ICC=0.476 (0.298-0.621)
EPI
HC
+1.96 σ
0.0103
mean
-0.0003
-1.96 σ
-0.0110
Difference between CNN and FS [mm^{-1}]
Average of FS and CNN [mm^{-1}]

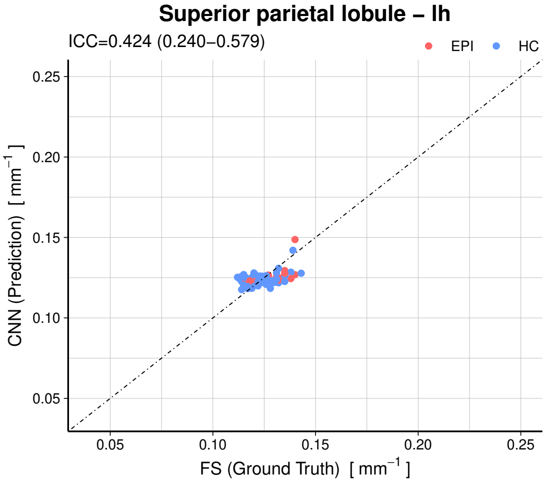
Superior parietal lobule – lh
ICC=0.424 (0.240–0.579)
EPI
HC
CNN (Prediction) [mm^{-1}]
FS (Ground Truth) [mm^{-1}]

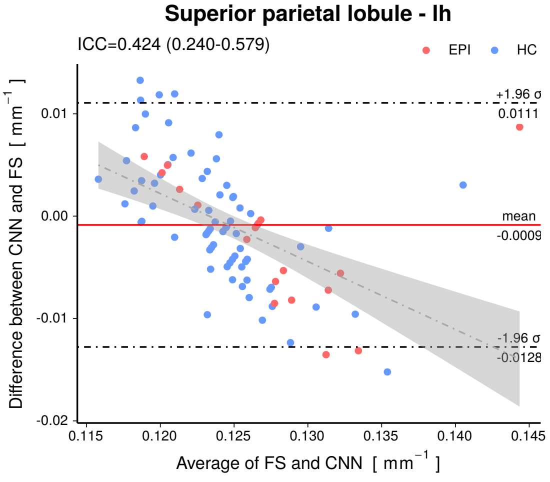
Superior parietal lobule - lh
ICC=0.424 (0.240-0.579)
EPI
HC
+1.96 σ
0.0111
mean
-0.0009
-1.96 σ
-0.0128
Difference between CNN and FS [mm^{-1}]
Average of FS and CNN [mm^{-1}]

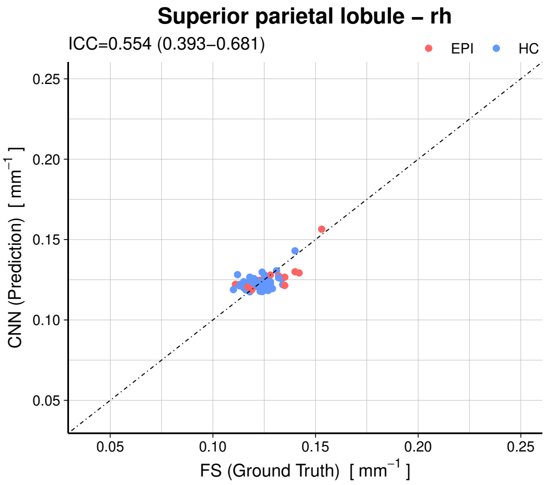
Superior parietal lobule – rh
ICC=0.554 (0.393–0.681)
EPI
HC
CNN (Prediction) [mm^{-1}]
FS (Ground Truth) [mm^{-1}]

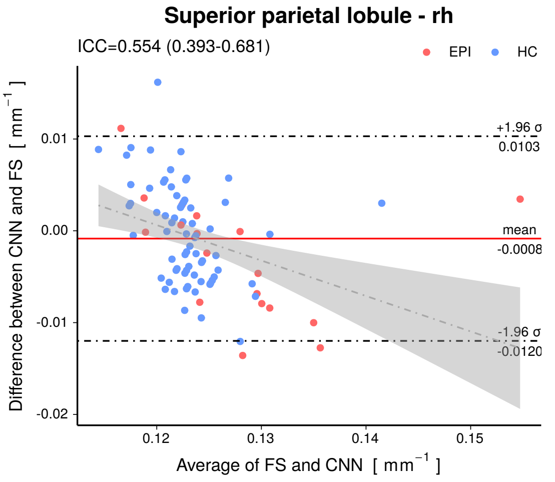
Superior parietal lobule - rh
ICC=0.554 (0.393-0.681)
EPI
HC
+1.96 σ
0.0103
mean
-0.0008
-1.96 σ
-0.0120
Difference between CNN and FS [mm^{-1}]
Average of FS and CNN [mm^{-1}]

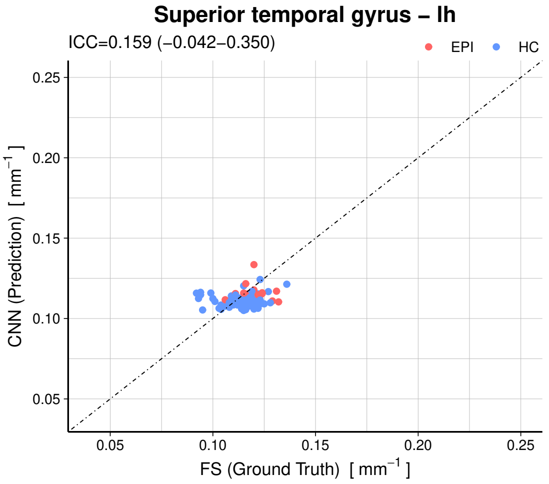
Superior temporal gyrus – lh
ICC=0.159 (–0.042–0.350)
EPI
HC
CNN (Prediction) [mm^{-1}]
FS (Ground Truth) [mm^{-1}]

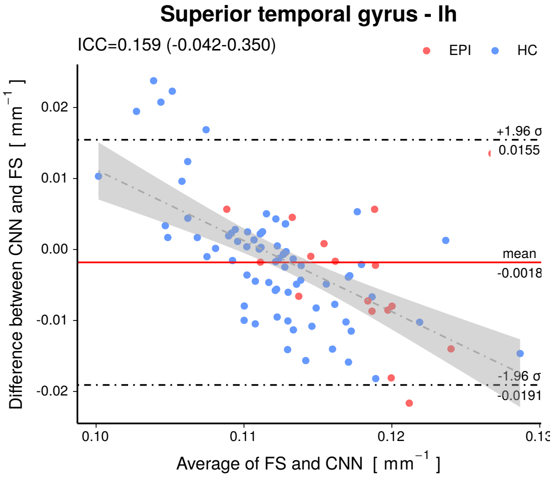
Superior temporal gyrus - lh
ICC=0.159 (-0.042-0.350)
EPI
HC
+1.96 σ
0.0155
mean
-0.0018
-1.96 σ
-0.0191
Difference between CNN and FS [mm^{-1}]
Average of FS and CNN [mm^{-1}]

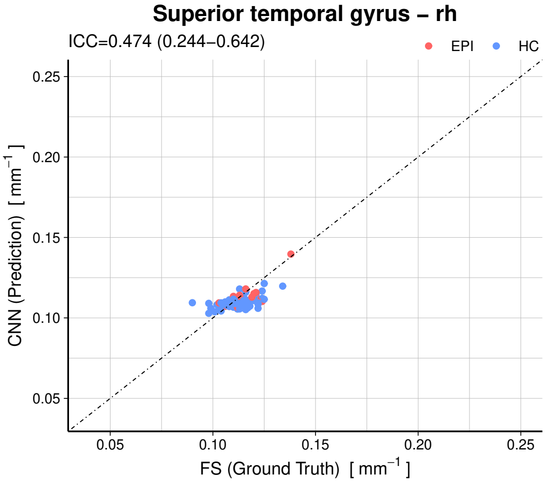
Superior temporal gyrus – rh
ICC=0.474 (0.244–0.642)
EPI
HC
CNN (Prediction) [mm^{-1}]
FS (Ground Truth) [mm^{-1}]

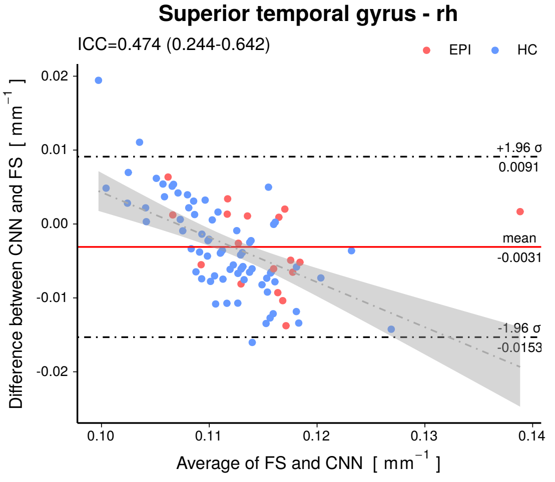
Superior temporal gyrus - rh
ICC=0.474 (0.244-0.642)
EPI
HC
+1.96 σ
0.0091
mean
-0.0031
-1.96 σ
-0.0153
Difference between CNN and FS [mm^{-1}]
Average of FS and CNN [mm^{-1}]

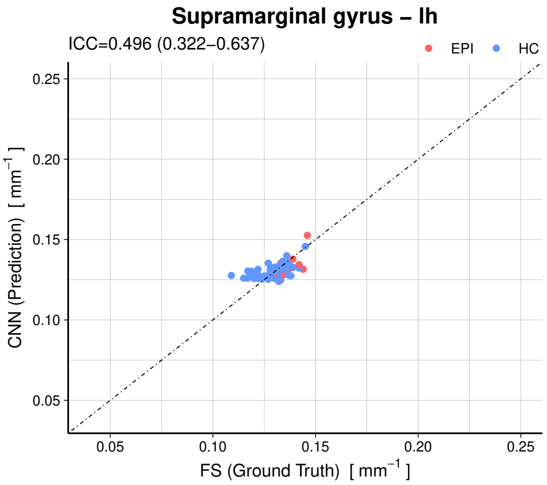
Supramarginal gyrus – lh
ICC=0.496 (0.322–0.637)
EPI
HC
CNN (Prediction) [mm^{-1}]
FS (Ground Truth) [mm^{-1}]
0.05
0.10
0.15
0.20
0.25

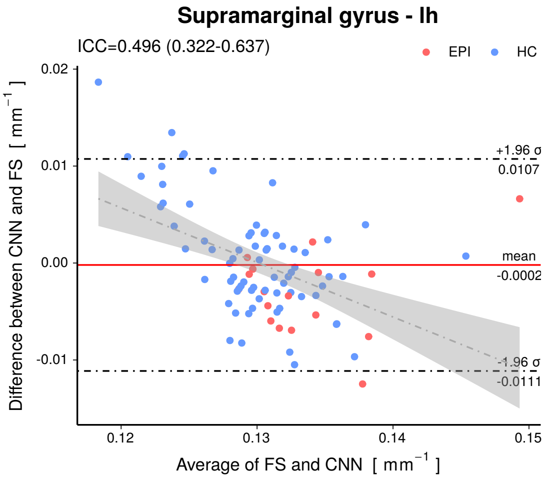
Supramarginal gyrus - lh
ICC=0.496 (0.322-0.637)
EPI
HC
+1.96 σ
0.0107
mean
-0.0002
-1.96 σ
-0.0111
Difference between CNN and FS [mm^{-1}]
Average of FS and CNN [mm^{-1}]

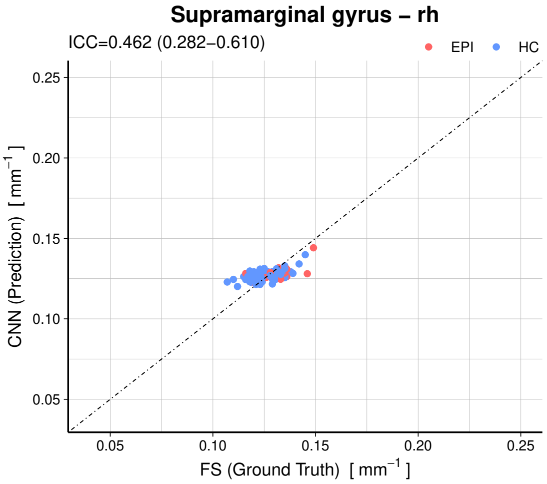
Supramarginal gyrus – rh
ICC=0.462 (0.282–0.610)
EPI
HC
CNN (Prediction) [mm^{-1}]
FS (Ground Truth) [mm^{-1}]

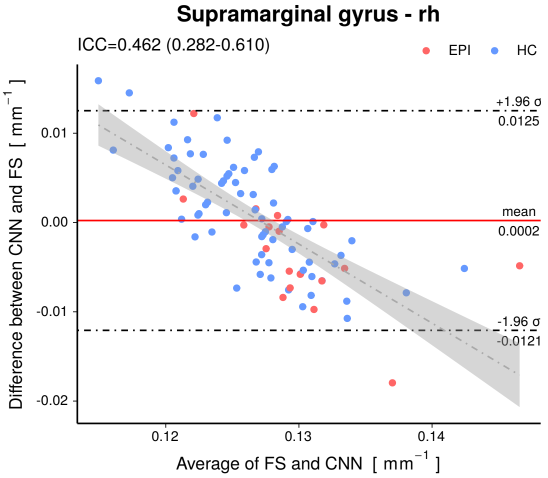
Supramarginal gyrus - rh
ICC=0.462 (0.282-0.610)
EPI
HC
+1.96 σ
0.0125
mean
0.0002
-1.96 σ
-0.0121
Difference between CNN and FS [mm^{-1}]
Average of FS and CNN [mm^{-1}]

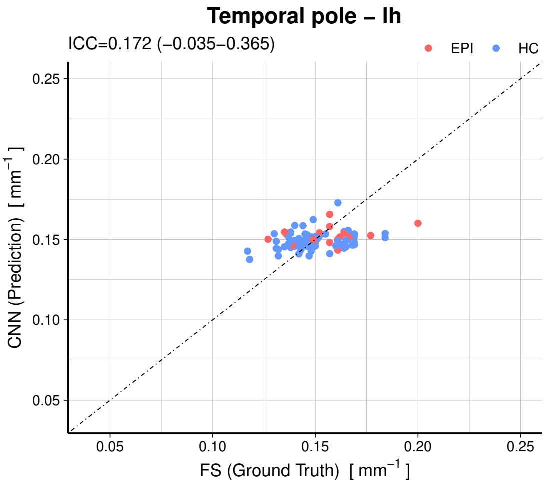
Temporal pole – lh
ICC=0.172 (–0.035–0.365)
EPI
HC
CNN (Prediction) [mm^{-1}]
FS (Ground Truth) [mm^{-1}]

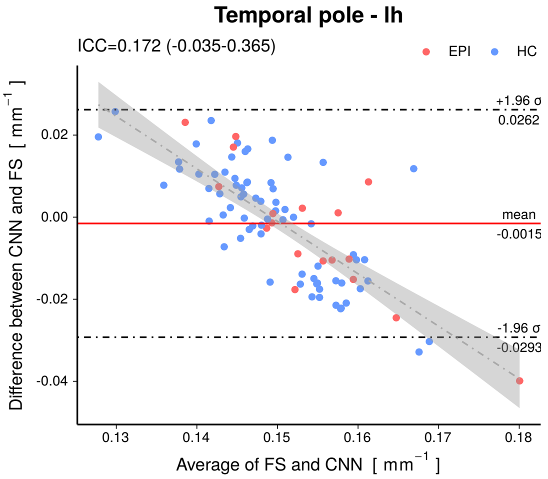
Temporal pole - lh
ICC=0.172 (-0.035-0.365)
EPI
HC
+1.96 σ
0.0262
mean
-0.0015
-1.96 σ
-0.0293
Difference between CNN and FS [mm^{-1}]
Average of FS and CNN [mm^{-1}]

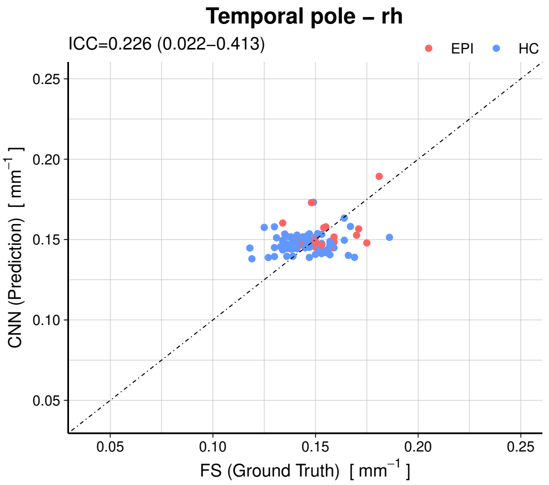
Temporal pole – rh
ICC=0.226 (0.022–0.413)
EPI
HC
CNN (Prediction) [mm^{-1}]
FS (Ground Truth) [mm^{-1}]
0.05
0.10
0.15
0.20
0.25

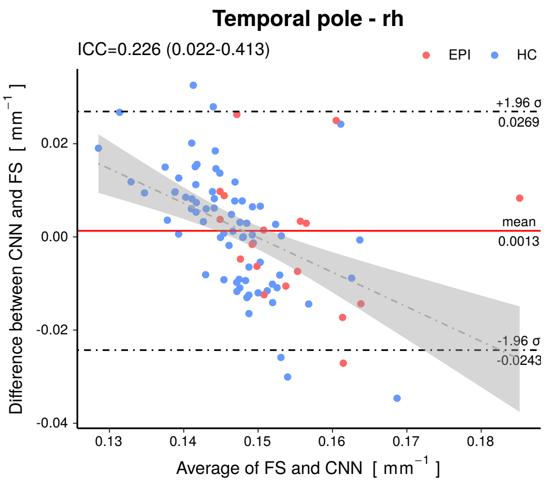
Temporal pole - rh
ICC=0.226 (0.022-0.413)
EPI
HC
+1.96 σ
0.0269
mean
0.0013
-1.96 σ
-0.0243
Difference between CNN and FS [mm^{-1}]
Average of FS and CNN [mm^{-1}]

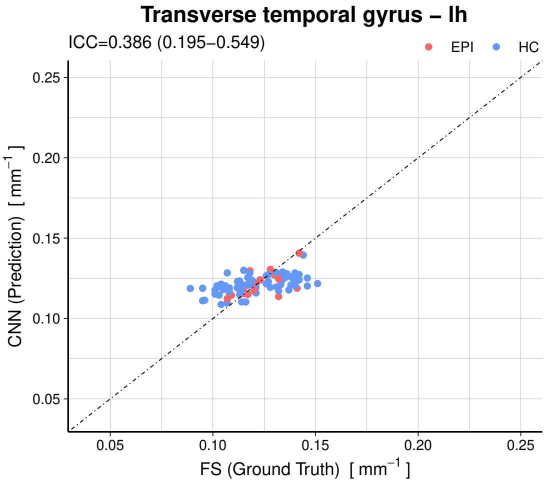
Transverse temporal gyrus – lh
ICC=0.386 (0.195–0.549)
EPI
HC
CNN (Prediction) [mm^{-1}]
FS (Ground Truth) [mm^{-1}]

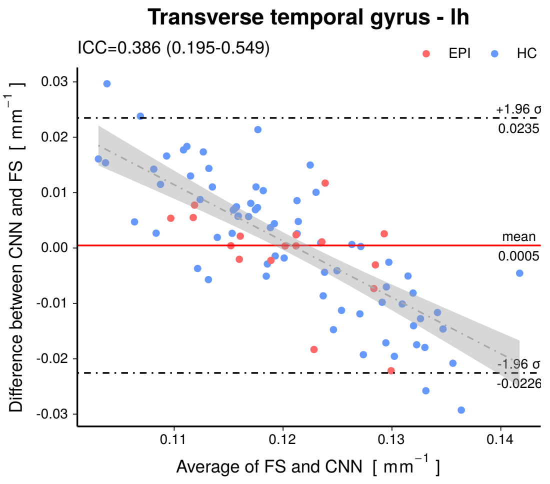
Transverse temporal gyrus - lh
ICC=0.386 (0.195-0.549)
EPI
HC
+1.96 σ
0.0235
mean
0.0005
-1.96 σ
-0.0226
Difference between CNN and FS [mm^{-1}]
Average of FS and CNN [mm^{-1}]

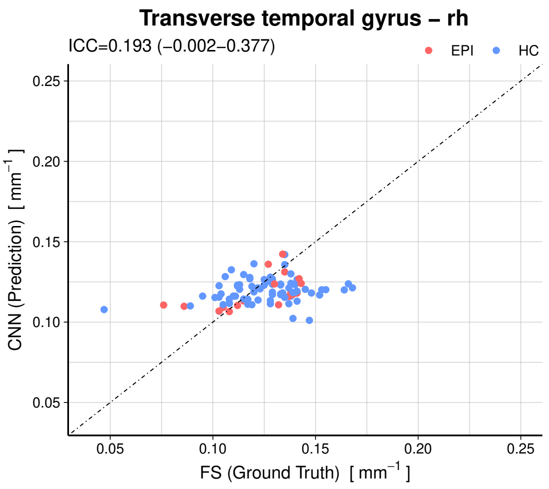
Transverse temporal gyrus – rh
ICC=0.193 (–0.002–0.377)
EPI
HC
CNN (Prediction) [mm^{-1}]
FS (Ground Truth) [mm^{-1}]

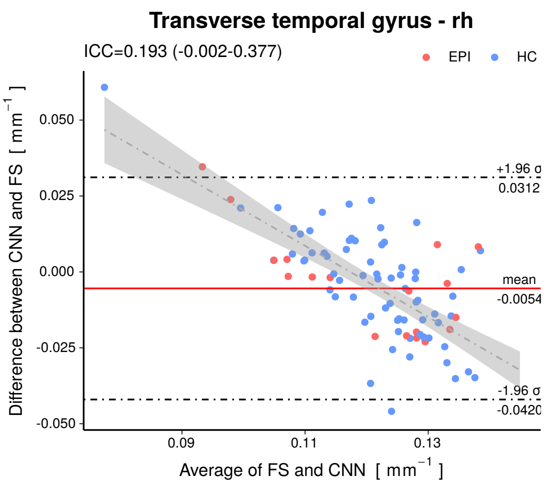
Transverse temporal gyrus - rh
ICC=0.193 (-0.002-0.377)
EPI
HC
+1.96 σ
0.0312
mean
-0.0054
-1.96 σ
-0.0420
Difference between CNN and FS [mm^{-1}]
Average of FS and CNN [mm^{-1}]

3.4 Cortical Thickness against Age Plots

Predictions of the cortical thickness together with ground truth plotted against age. The intercept and coefficient (mm/year) with associated p-value of a linear model fitted to the data points are reported in the subtitle.

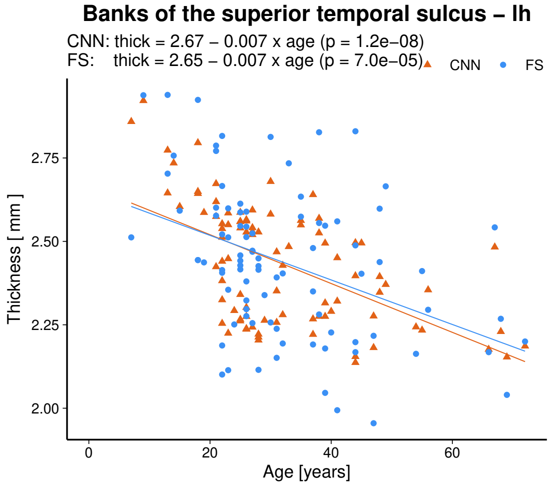

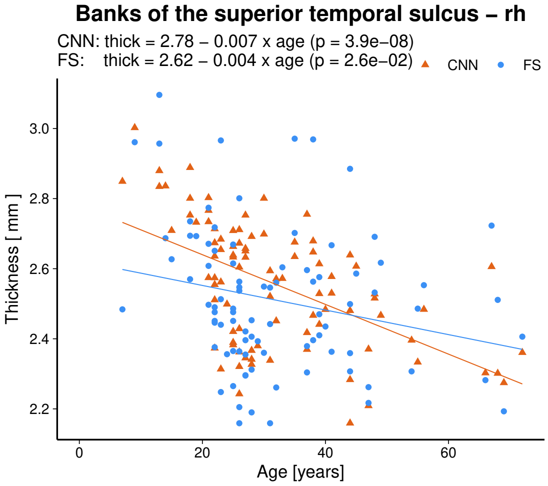

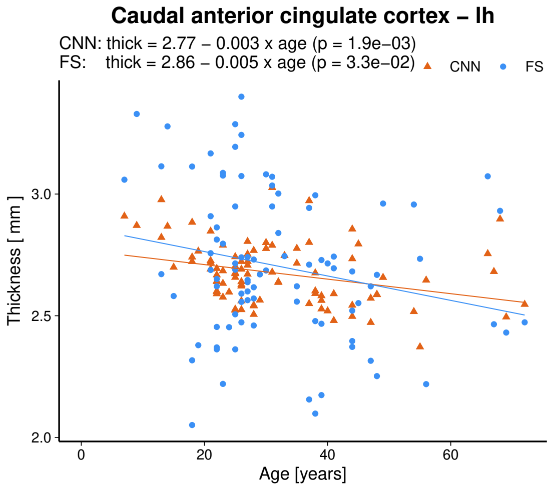

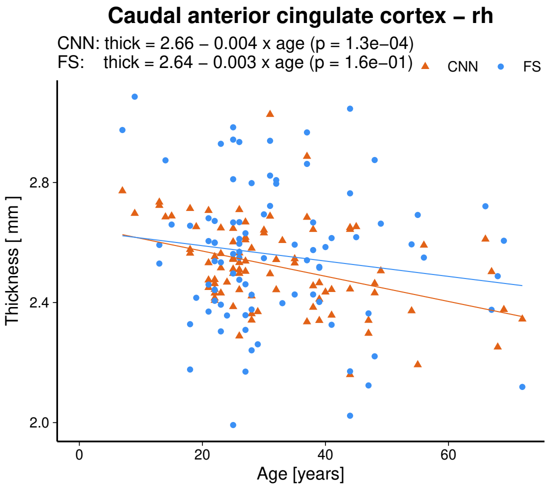

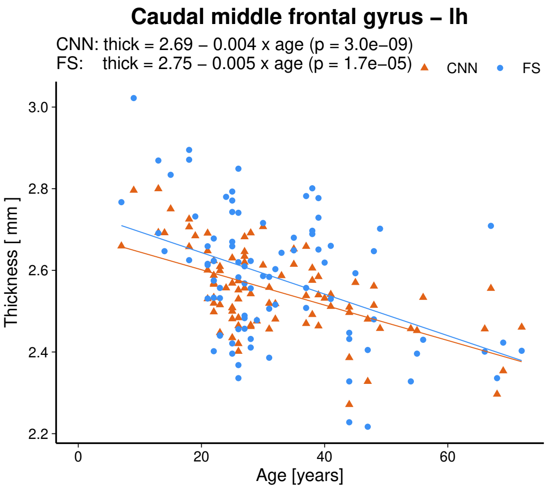
Caudal middle frontal gyrus – lh
CNN: thick = 2.69 – 0.004 x age (p = 3.0e–09)
FS: thick = 2.75 – 0.005 x age (p = 1.7e–05)
CNN
FS
Thickness [mm]
Age [years]
3.0
2.8
2.6
2.4
2.2
0
20
40
60

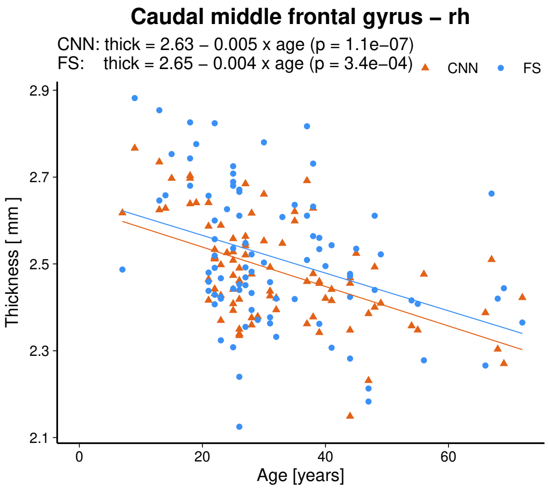
Caudal middle frontal gyrus – rh
CNN: thick = 2.63 – 0.005 x age (p = 1.1e–07)
FS: thick = 2.65 – 0.004 x age (p = 3.4e–04)
CNN
FS
Thickness [mm]
Age [years]
2.9
2.7
2.5
2.3
2.1
0
20
40
60

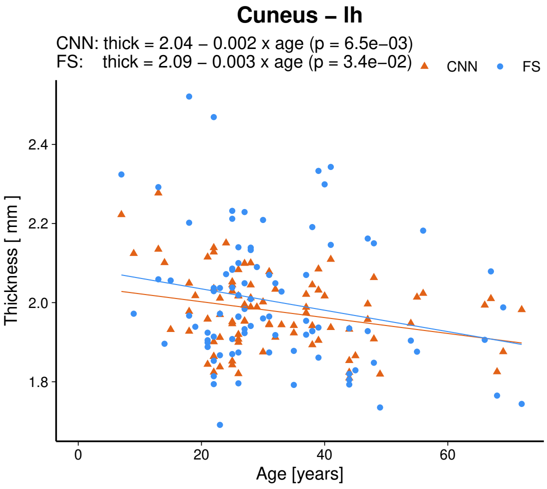
Cuneus – lh
CNN: thick = 2.04 – 0.002 x age (p = 6.5e–03)
FS: thick = 2.09 – 0.003 x age (p = 3.4e–02)
CNN
FS
Thickness [mm]
Age [years]
2.4
2.2
2.0
1.8
0
20
40
60

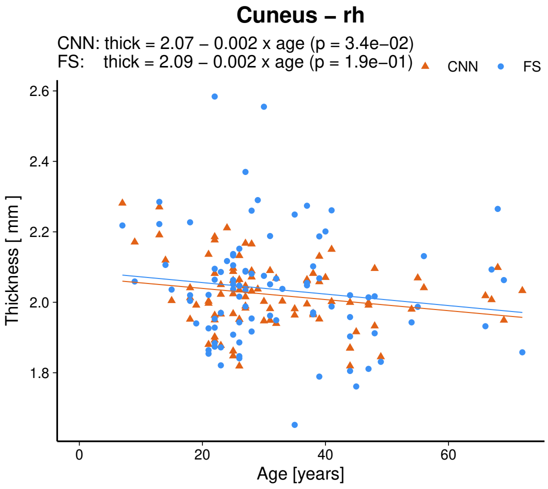
Cuneus – rh
CNN: thick = 2.07 – 0.002 x age (p = 3.4e–02)
FS: thick = 2.09 – 0.002 x age (p = 1.9e–01)
CNN
FS
Thickness [mm]
Age [years]
2.6
2.4
2.2
2.0
1.8
0
20
40
60

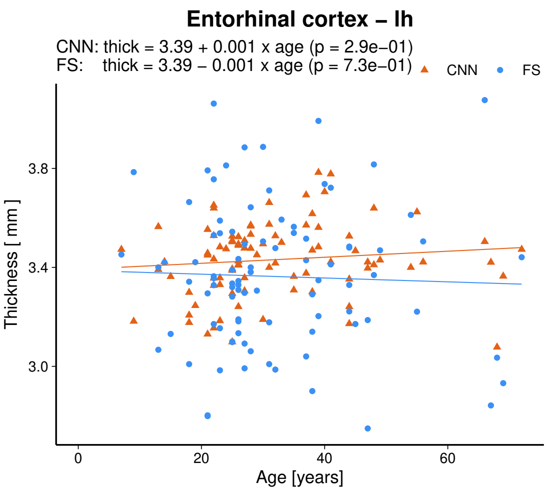
Entorhinal cortex – lh
CNN: thick = 3.39 + 0.001 x age (p = 2.9e–01)
FS: thick = 3.39 – 0.001 x age (p = 7.3e–01)
CNN
FS
Thickness [mm]
Age [years]
3.8
3.4
3.0
0
20
40
60

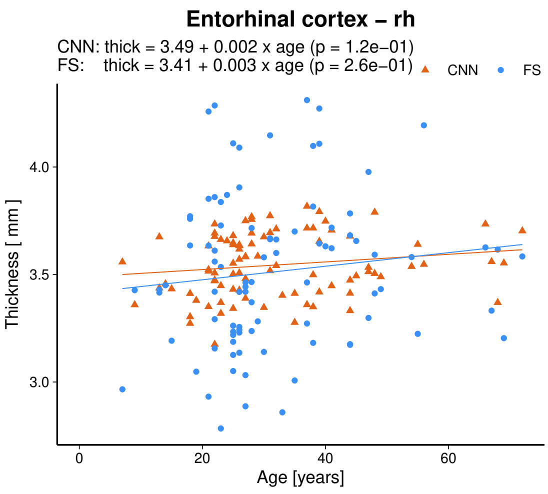
Entorhinal cortex – rh
CNN: thick = 3.49 + 0.002 x age (p = 1.2e–01)
FS: thick = 3.41 + 0.003 x age (p = 2.6e–01)
CNN
FS
Thickness [mm]
Age [years]
4.0
3.5
3.0
0
20
40
60

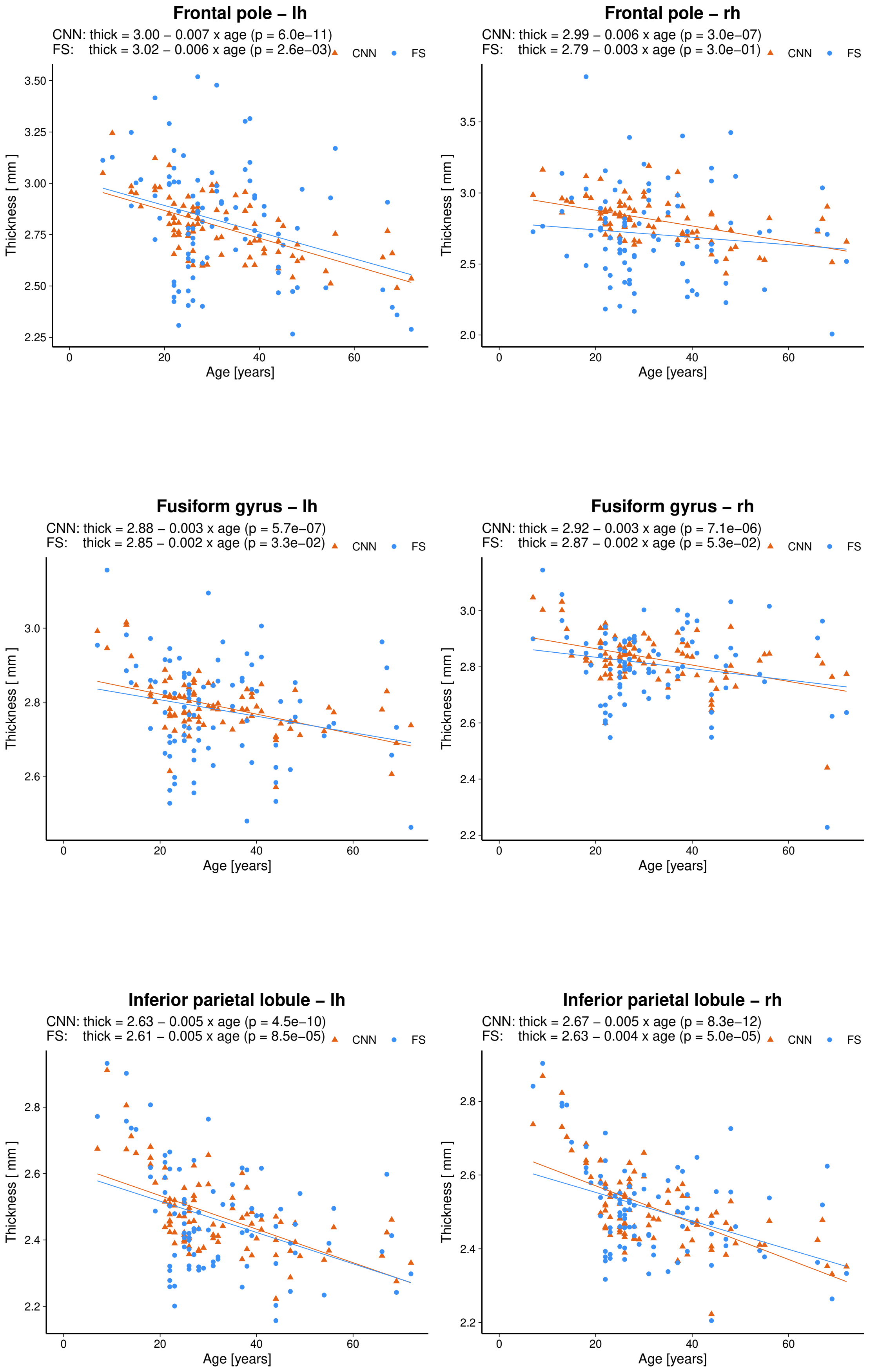

Frontal pole – lh
CNN: thick = 3.00 – 0.007 x age (p = 6.0e–11)
FS: thick = 3.02 – 0.006 x age (p = 2.6e–03)
CNN
FS
Thickness [mm]
Age [years]
Frontal pole – rh
CNN: thick = 2.99 – 0.006 x age (p = 3.0e–07)
FS: thick = 2.79 – 0.003 x age (p = 3.0e–01)
CNN
FS
Thickness [mm]
Age [years]
Fusiform gyrus – lh
CNN: thick = 2.88 – 0.003 x age (p = 5.7e–07)
FS: thick = 2.85 – 0.002 x age (p = 3.3e–02)
CNN
FS
Thickness [mm]
Age [years]
Fusiform gyrus – rh
CNN: thick = 2.92 – 0.003 x age (p = 7.1e–06)
FS: thick = 2.87 – 0.002 x age (p = 5.3e–02)
CNN
FS
Thickness [mm]
Age [years]
Inferior parietal lobule – lh
CNN: thick = 2.63 – 0.005 x age (p = 4.5e–10)
FS: thick = 2.61 – 0.005 x age (p = 8.5e–05)
CNN
FS
Thickness [mm]
Age [years]
Inferior parietal lobule – rh
CNN: thick = 2.67 – 0.005 x age (p = 8.3e–12)
FS: thick = 2.63 – 0.004 x age (p = 5.0e–05)
CNN
FS
Thickness [mm]
Age [years]

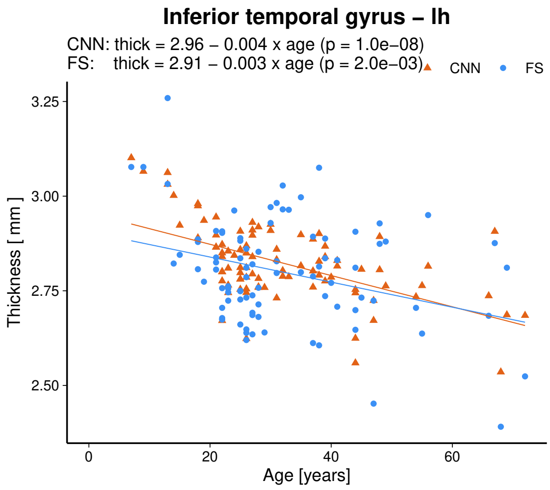
Inferior temporal gyrus – lh
CNN: thick = 2.96 – 0.004 x age (p = 1.0e–08)
FS: thick = 2.91 – 0.003 x age (p = 2.0e–03)
CNN
FS
Thickness [mm]
Age [years]
3.25
3.00
2.75
2.50
0
20
40
60

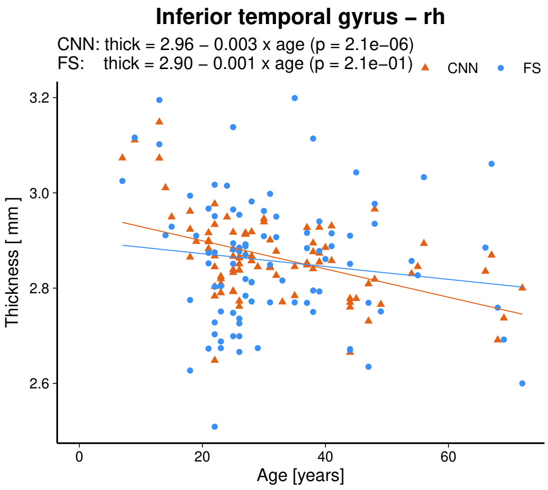
Inferior temporal gyrus – rh
CNN: thick = 2.96 – 0.003 x age (p = 2.1e–06)
FS: thick = 2.90 – 0.001 x age (p = 2.1e–01)
CNN
FS
Thickness [mm]
Age [years]
3.2
3.0
2.8
2.6
0
20
40
60

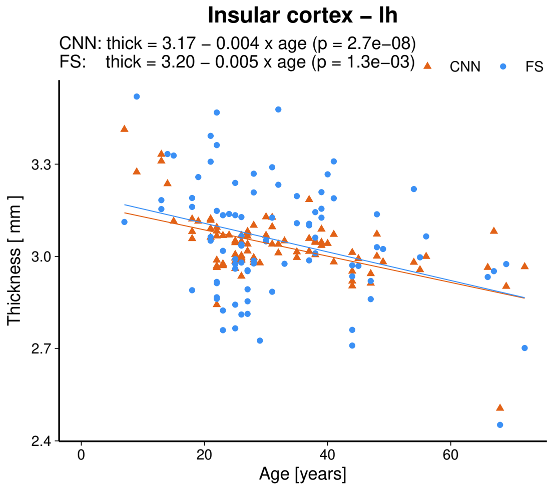
Insular cortex – lh
CNN: thick = 3.17 – 0.004 x age (p = 2.7e–08)
FS: thick = 3.20 – 0.005 x age (p = 1.3e–03)
CNN
FS
Thickness [mm]
Age [years]
3.3
3.0
2.7
2.4
0
20
40
60

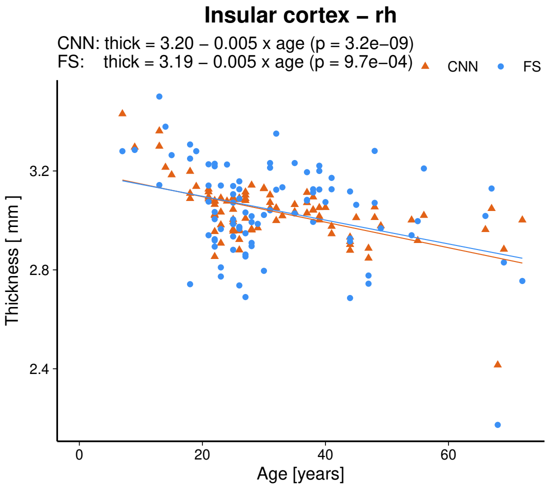
Insular cortex – rh
CNN: thick = 3.20 – 0.005 x age (p = 3.2e–09)
FS: thick = 3.19 – 0.005 x age (p = 9.7e–04)
CNN
FS
Thickness [mm]
Age [years]
3.2
2.8
2.4
0
20
40
60

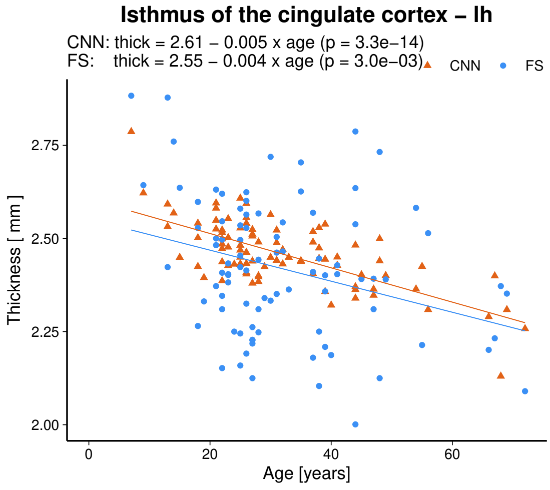
Isthmus of the cingulate cortex – lh
CNN: thick = 2.61 – 0.005 x age (p = 3.3e–14)
FS: thick = 2.55 – 0.004 x age (p = 3.0e–03)
CNN
FS
Thickness [mm]
Age [years]
2.75
2.50
2.25
2.00
0
20
40
60

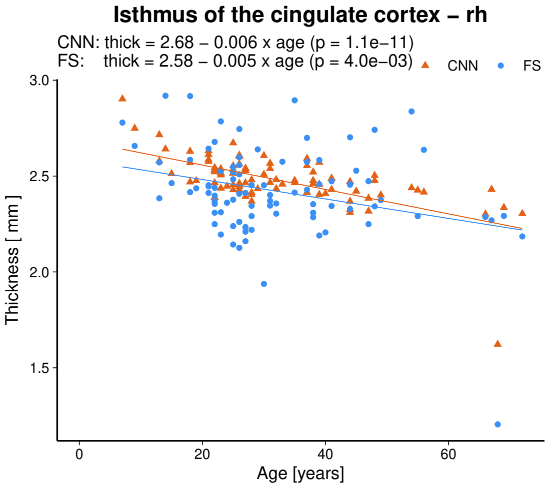
Isthmus of the cingulate cortex – rh
CNN: thick = 2.68 – 0.006 x age (p = 1.1e–11)
FS: thick = 2.58 – 0.005 x age (p = 4.0e–03)
CNN
FS
Thickness [mm]
Age [years]
3.0
2.5
2.0
1.5
0
20
40
60

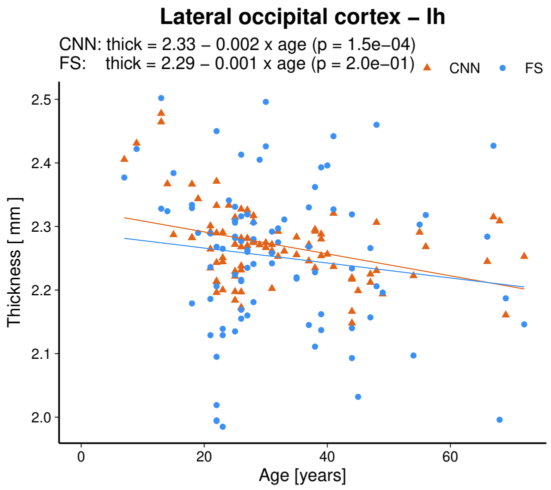
Lateral occipital cortex – lh
CNN: thick = 2.33 – 0.002 x age (p = 1.5e–04)
FS: thick = 2.29 – 0.001 x age (p = 2.0e–01)
CNN
FS
Thickness [mm]
Age [years]

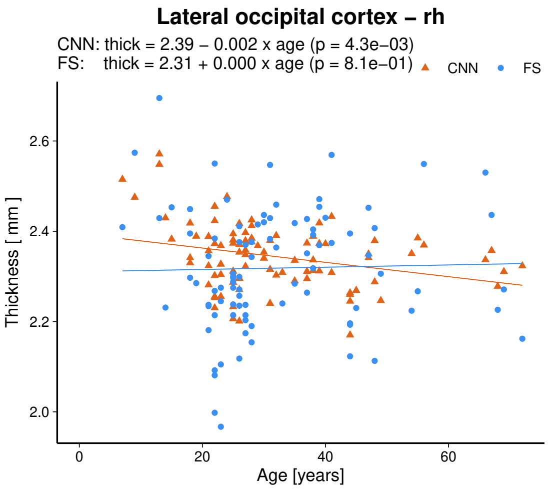
Lateral occipital cortex – rh
CNN: thick = 2.39 – 0.002 x age (p = 4.3e–03)
FS: thick = 2.31 + 0.000 x age (p = 8.1e–01)
CNN
FS
Thickness [mm]
Age [years]

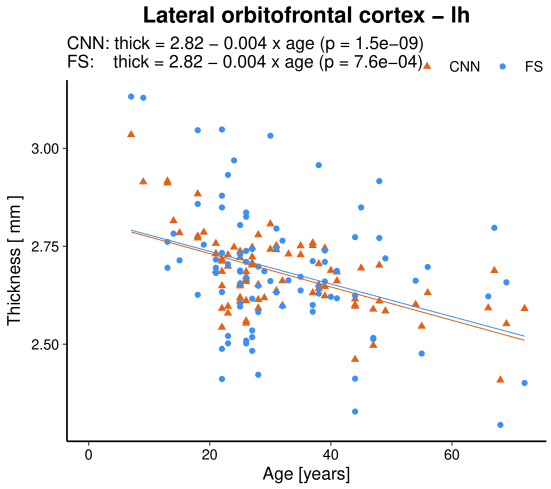
Lateral orbitofrontal cortex – lh
CNN: thick = 2.82 – 0.004 x age (p = 1.5e–09)
FS: thick = 2.82 – 0.004 x age (p = 7.6e–04)
CNN
FS
Thickness [mm]
Age [years]

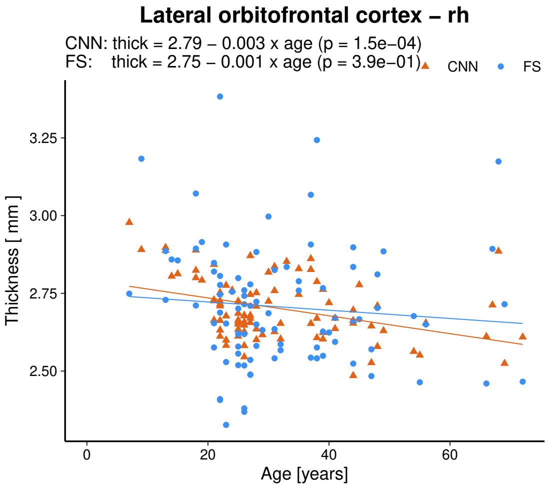
Lateral orbitofrontal cortex – rh
CNN: thick = 2.79 – 0.003 x age (p = 1.5e–04)
FS: thick = 2.75 – 0.001 x age (p = 3.9e–01)
CNN
FS
Thickness [mm]
Age [years]

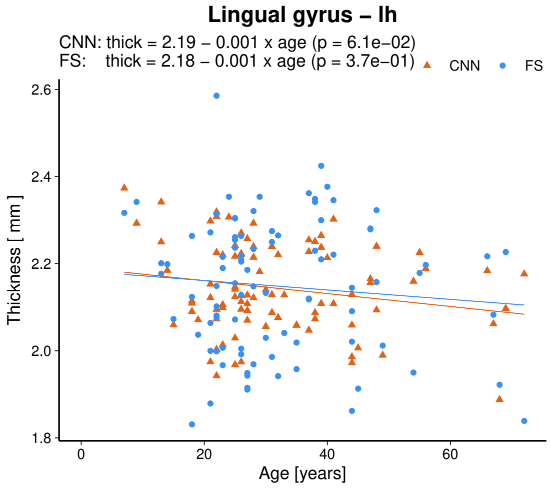
Lingual gyrus – lh
CNN: thick = 2.19 – 0.001 x age (p = 6.1e–02)
FS: thick = 2.18 – 0.001 x age (p = 3.7e–01)
CNN
FS
Thickness [mm]
Age [years]

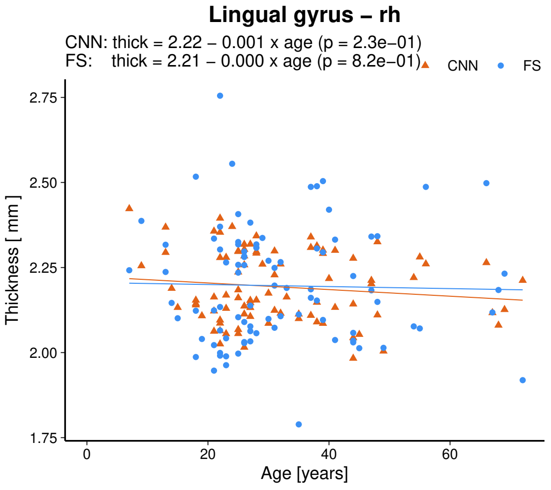
Lingual gyrus – rh
CNN: thick = 2.22 – 0.001 x age (p = 2.3e–01)
FS: thick = 2.21 – 0.000 x age (p = 8.2e–01)
CNN
FS
Thickness [mm]
Age [years]

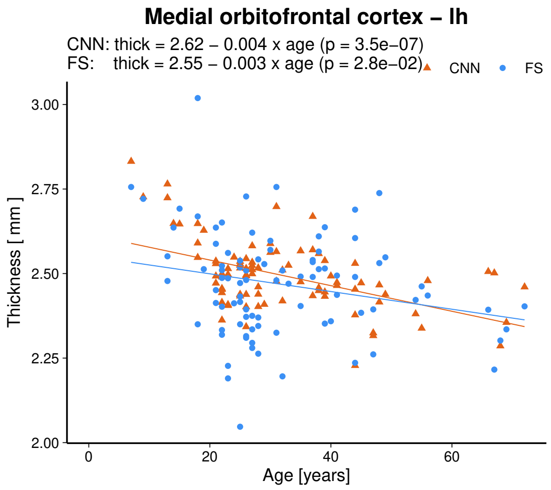

Medial orbitofrontal cortex – lh
CNN: thick = 2.62 – 0.004 x age (p = 3.5e–07)
FS: thick = 2.55 – 0.003 x age (p = 2.8e–02)
CNN
FS
Thickness [mm]
3.00
2.75
2.50
2.25
2.00
0
20
40
60
Age [years]

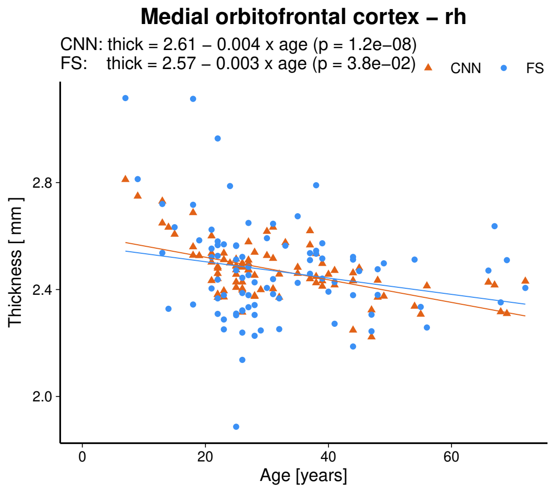

Medial orbitofrontal cortex – rh
CNN: thick = 2.61 – 0.004 x age (p = 1.2e–08)
FS: thick = 2.57 – 0.003 x age (p = 3.8e–02)
CNN
FS
Thickness [mm]
2.8
2.4
2.0
0
20
40
60
Age [years]

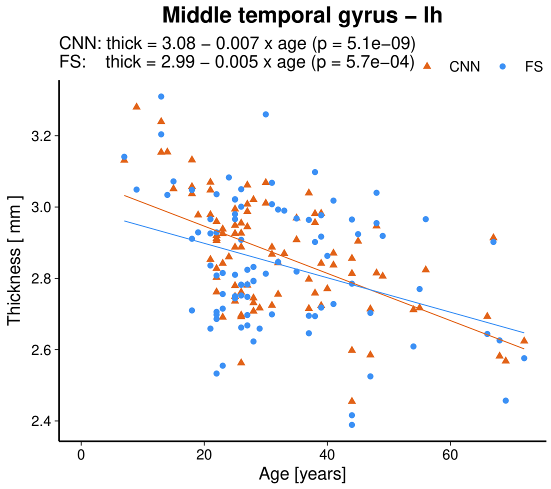

Middle temporal gyrus – lh
CNN: thick = 3.08 – 0.007 x age (p = 5.1e–09)
FS: thick = 2.99 – 0.005 x age (p = 5.7e–04)
CNN
FS
Thickness [mm]
3.2
3.0
2.8
2.6
2.4
0
20
40
60
Age [years]

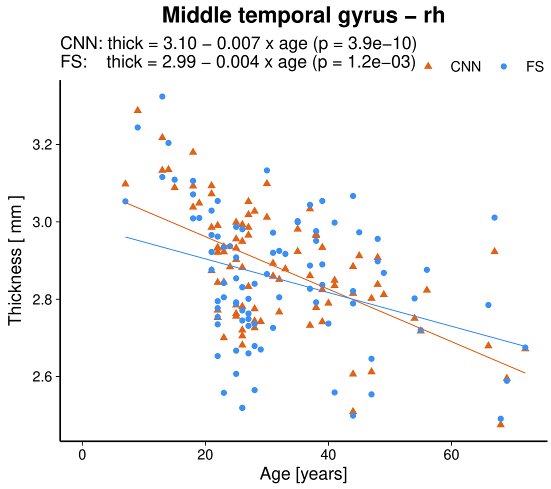

Middle temporal gyrus – rh
CNN: thick = 3.10 – 0.007 x age (p = 3.9e–10)
FS: thick = 2.99 – 0.004 x age (p = 1.2e–03)
CNN
FS
Thickness [mm]
3.2
3.0
2.8
2.6
0
20
40
60
Age [years]

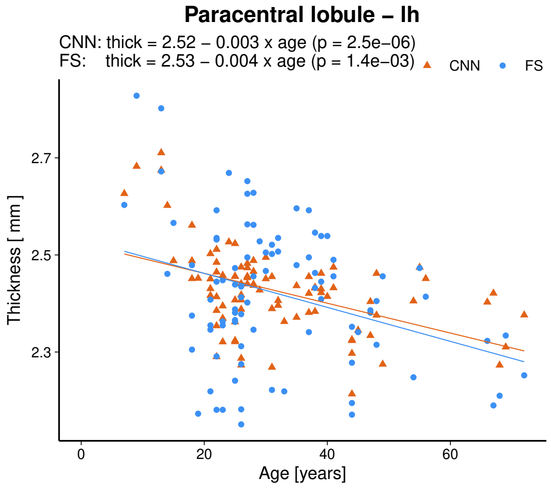

Paracentral lobule – lh
CNN: thick = 2.52 – 0.003 x age (p = 2.5e–06)
FS: thick = 2.53 – 0.004 x age (p = 1.4e–03)
CNN
FS
Thickness [mm]
2.7
2.5
2.3
0
20
40
60
Age [years]

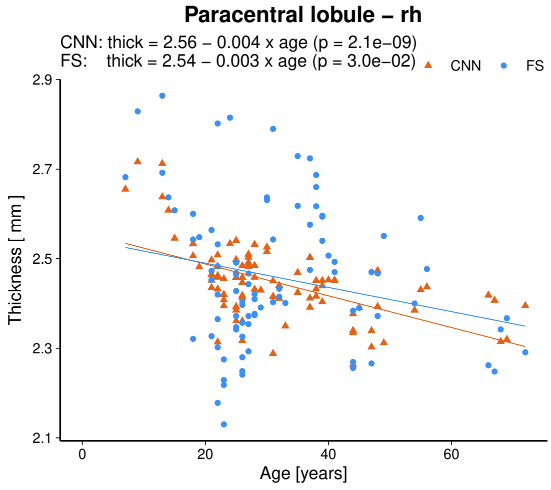

Paracentral lobule – rh
CNN: thick = 2.56 – 0.004 x age (p = 2.1e–09)
FS: thick = 2.54 – 0.003 x age (p = 3.0e–02)
CNN
FS
Thickness [mm]
2.9
2.7
2.5
2.3
2.1
0
20
40
60
Age [years]

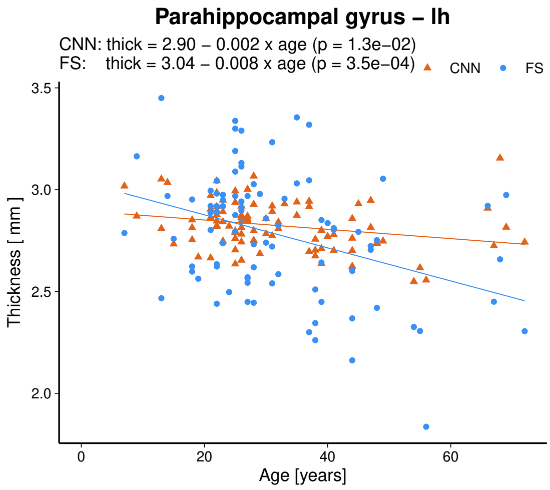
Parahippocampal gyrus – lh
CNN: thick = 2.90 – 0.002 x age (p = 1.3e–02)
FS: thick = 3.04 – 0.008 x age (p = 3.5e–04)
CNN
FS
Thickness [mm]
Age [years]

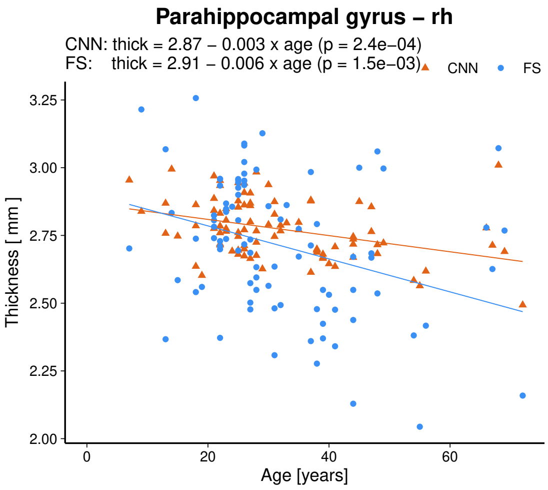
Parahippocampal gyrus – rh
CNN: thick = 2.87 – 0.003 x age (p = 2.4e–04)
FS: thick = 2.91 – 0.006 x age (p = 1.5e–03)
CNN
FS
Thickness [mm]
Age [years]

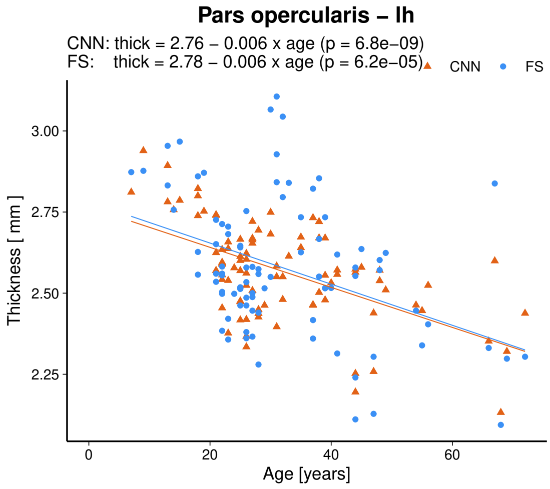
Pars opercularis – lh
CNN: thick = 2.76 – 0.006 x age (p = 6.8e–09)
FS: thick = 2.78 – 0.006 x age (p = 6.2e–05)
CNN
FS
Thickness [mm]
Age [years]

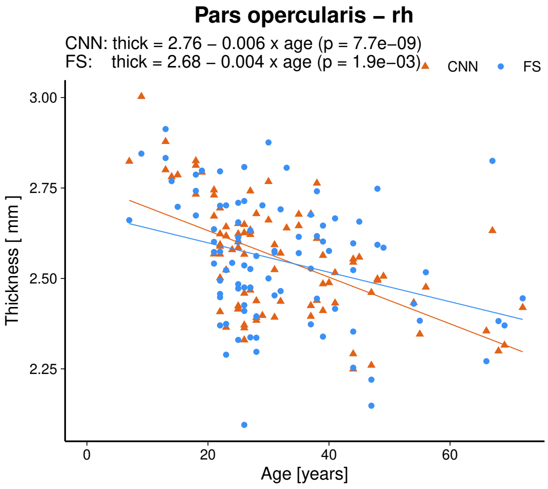
Pars opercularis – rh
CNN: thick = 2.76 – 0.006 x age (p = 7.7e–09)
FS: thick = 2.68 – 0.004 x age (p = 1.9e–03)
CNN
FS
Thickness [mm]
Age [years]

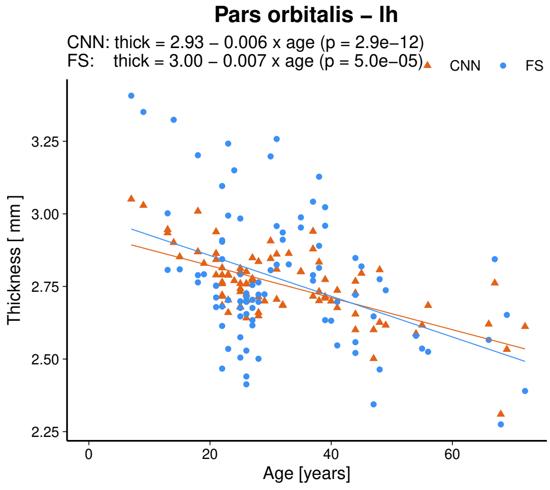
Pars orbitalis – lh
CNN: thick = 2.93 – 0.006 x age (p = 2.9e–12)
FS: thick = 3.00 – 0.007 x age (p = 5.0e–05)
CNN
FS
Thickness [mm]
Age [years]

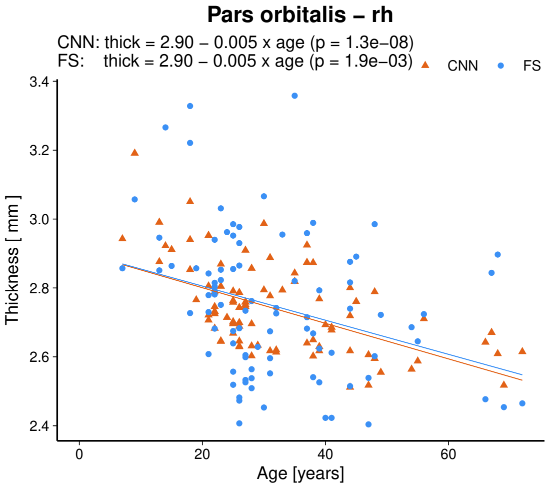
Pars orbitalis – rh
CNN: thick = 2.90 – 0.005 x age (p = 1.3e–08)
FS: thick = 2.90 – 0.005 x age (p = 1.9e–03)
CNN
FS
Thickness [mm]
Age [years]

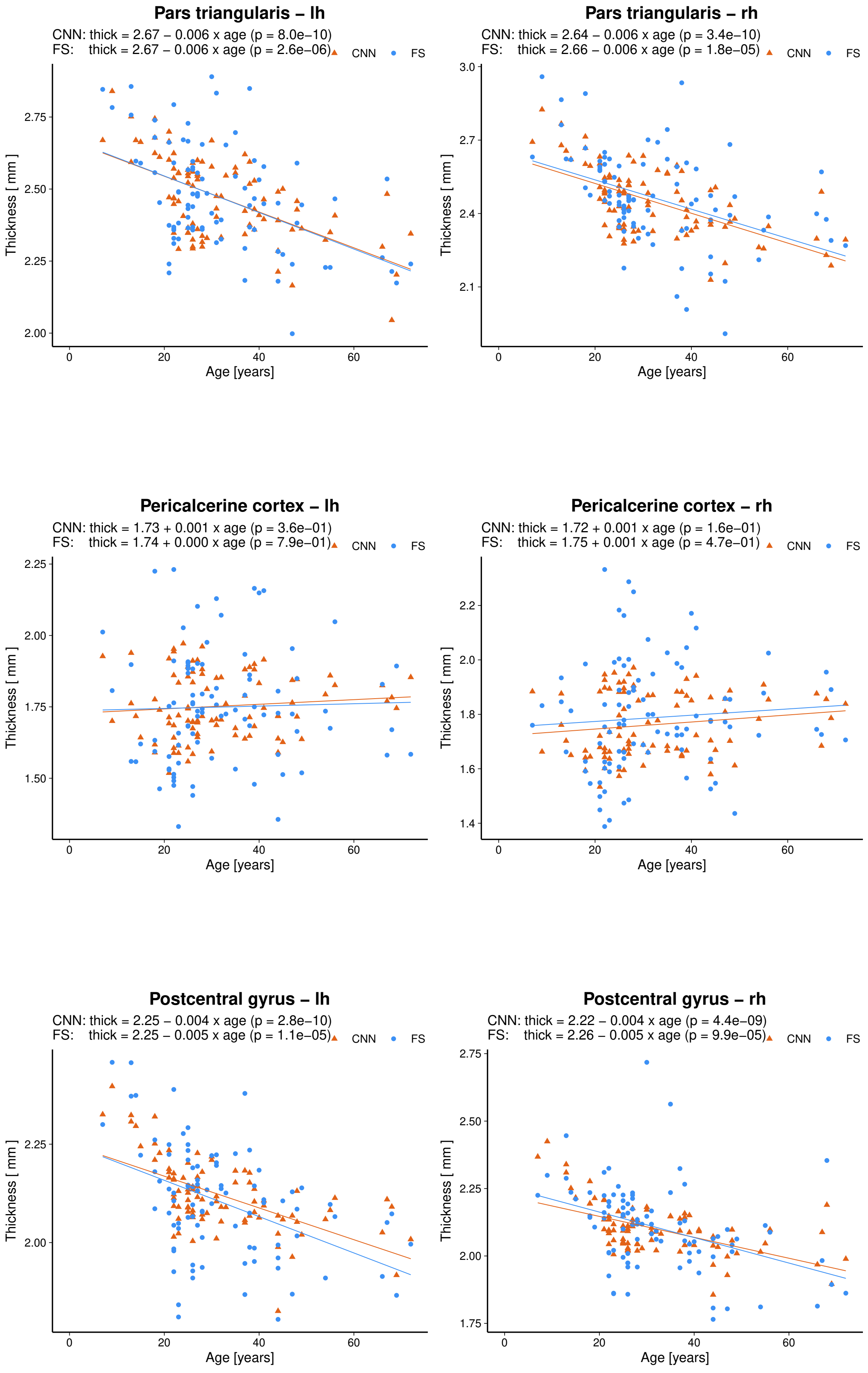

Pars triangularis – lh
CNN: thick = 2.67 – 0.006 x age (p = 8.0e–10)
FS: thick = 2.67 – 0.006 x age (p = 2.6e–06)
CNN
FS
Thickness [mm]
Age [years]
Pars triangularis – rh
CNN: thick = 2.64 – 0.006 x age (p = 3.4e–10)
FS: thick = 2.66 – 0.006 x age (p = 1.8e–05)
CNN
FS
Thickness [mm]
Age [years]
Pericalcerine cortex – lh
CNN: thick = 1.73 + 0.001 x age (p = 3.6e–01)
FS: thick = 1.74 + 0.000 x age (p = 7.9e–01)
CNN
FS
Thickness [mm]
Age [years]
Pericalcerine cortex – rh
CNN: thick = 1.72 + 0.001 x age (p = 1.6e–01)
FS: thick = 1.75 + 0.001 x age (p = 4.7e–01)
CNN
FS
Thickness [mm]
Age [years]
Postcentral gyrus – lh
CNN: thick = 2.25 – 0.004 x age (p = 2.8e–10)
FS: thick = 2.25 – 0.005 x age (p = 1.1e–05)
CNN
FS
Thickness [mm]
Age [years]
Postcentral gyrus – rh
CNN: thick = 2.22 – 0.004 x age (p = 4.4e–09)
FS: thick = 2.26 – 0.005 x age (p = 9.9e–05)
CNN
FS
Thickness [mm]
Age [years]

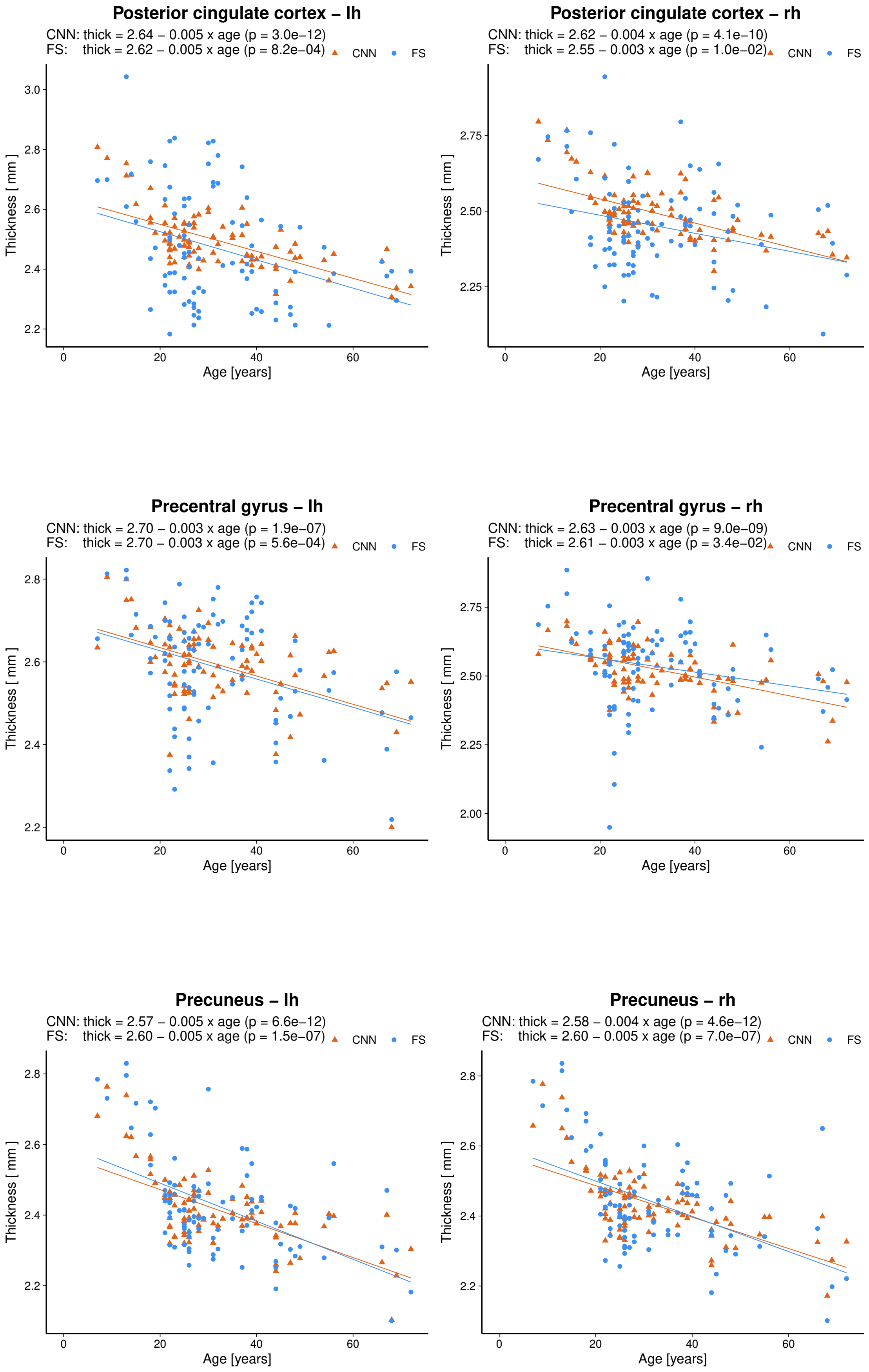
Posterior cingulate cortex – lh
CNN: thick = 2.64 – 0.005 x age (p = 3.0e–12)
FS: thick = 2.62 – 0.005 x age (p = 8.2e–04)
CNN
FS
Thickness [mm]
Age [years]
Posterior cingulate cortex – rh
CNN: thick = 2.62 – 0.004 x age (p = 4.1e–10)
FS: thick = 2.55 – 0.003 x age (p = 1.0e–02)
CNN
FS
Thickness [mm]
Age [years]
Precentral gyrus – lh
CNN: thick = 2.70 – 0.003 x age (p = 1.9e–07)
FS: thick = 2.70 – 0.003 x age (p = 5.6e–04)
CNN
FS
Thickness [mm]
Age [years]
Precentral gyrus – rh
CNN: thick = 2.63 – 0.003 x age (p = 9.0e–09)
FS: thick = 2.61 – 0.003 x age (p = 3.4e–02)
CNN
FS
Thickness [mm]
Age [years]
Precuneus – lh
CNN: thick = 2.57 – 0.005 x age (p = 6.6e–12)
FS: thick = 2.60 – 0.005 x age (p = 1.5e–07)
CNN
FS
Thickness [mm]
Age [years]
Precuneus – rh
CNN: thick = 2.58 – 0.004 x age (p = 4.6e–12)
FS: thick = 2.60 – 0.005 x age (p = 7.0e–07)
CNN
FS
Thickness [mm]
Age [years]

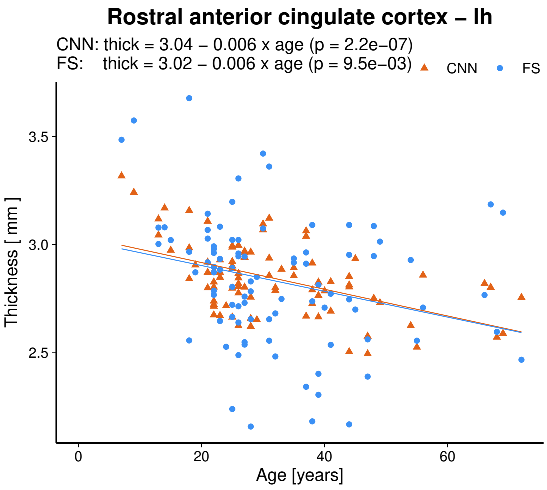
Rostral anterior cingulate cortex – lh
CNN: thick = 3.04 – 0.006 x age (p = 2.2e–07)
FS: thick = 3.02 – 0.006 x age (p = 9.5e–03)
CNN
FS
Thickness [mm]
Age [years]

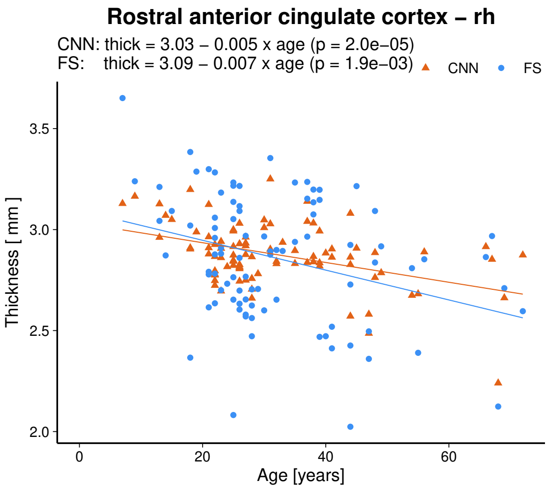
Rostral anterior cingulate cortex – rh
CNN: thick = 3.03 – 0.005 x age (p = 2.0e–05)
FS: thick = 3.09 – 0.007 x age (p = 1.9e–03)
CNN
FS
Thickness [mm]
Age [years]

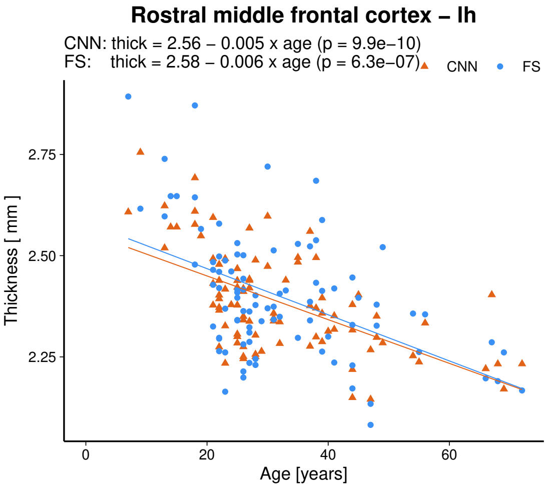
Rostral middle frontal cortex – lh
CNN: thick = 2.56 – 0.005 x age (p = 9.9e–10)
FS: thick = 2.58 – 0.006 x age (p = 6.3e–07)
CNN
FS
Thickness [mm]
Age [years]

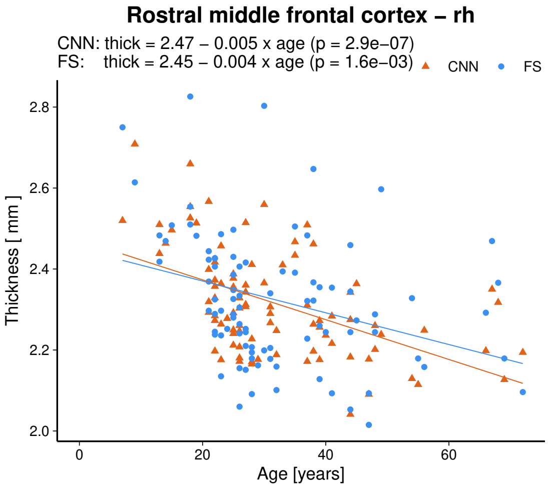
Rostral middle frontal cortex – rh
CNN: thick = 2.47 – 0.005 x age (p = 2.9e–07)
FS: thick = 2.45 – 0.004 x age (p = 1.6e–03)
CNN
FS
Thickness [mm]
Age [years]

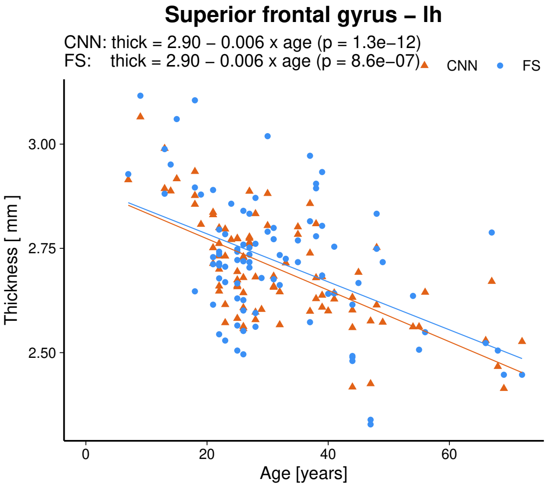
Superior frontal gyrus – lh
CNN: thick = 2.90 – 0.006 x age (p = 1.3e–12)
FS: thick = 2.90 – 0.006 x age (p = 8.6e–07)
CNN
FS
Thickness [mm]
Age [years]

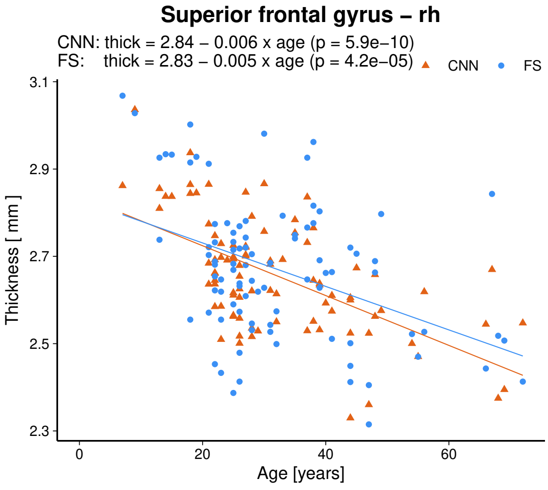
Superior frontal gyrus – rh
CNN: thick = 2.84 – 0.006 x age (p = 5.9e–10)
FS: thick = 2.83 – 0.005 x age (p = 4.2e–05)
CNN
FS
Thickness [mm]
Age [years]

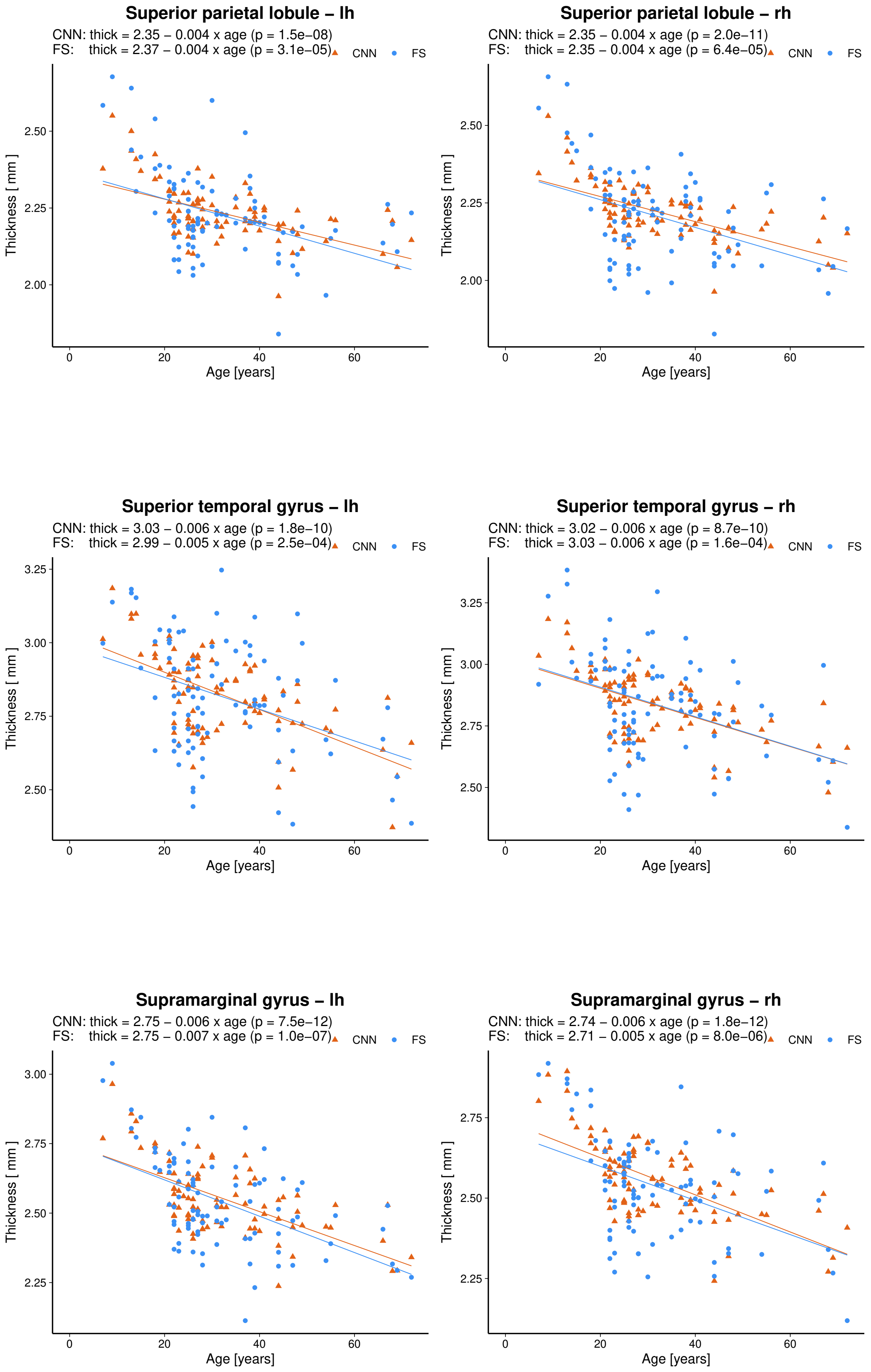

Superior parietal lobule – lh
CNN: thick = 2.35 – 0.004 x age (p = 1.5e–08)
FS: thick = 2.37 – 0.004 x age (p = 3.1e–05)
CNN
FS
Thickness [mm]
Age [years]
Superior parietal lobule – rh
CNN: thick = 2.35 – 0.004 x age (p = 2.0e–11)
FS: thick = 2.35 – 0.004 x age (p = 6.4e–05)
CNN
FS
Thickness [mm]
Age [years]
Superior temporal gyrus – lh
CNN: thick = 3.03 – 0.006 x age (p = 1.8e–10)
FS: thick = 2.99 – 0.005 x age (p = 2.5e–04)
CNN
FS
Thickness [mm]
Age [years]
Superior temporal gyrus – rh
CNN: thick = 3.02 – 0.006 x age (p = 8.7e–10)
FS: thick = 3.03 – 0.006 x age (p = 1.6e–04)
CNN
FS
Thickness [mm]
Age [years]
Supramarginal gyrus – lh
CNN: thick = 2.75 – 0.006 x age (p = 7.5e–12)
FS: thick = 2.75 – 0.007 x age (p = 1.0e–07)
CNN
FS
Thickness [mm]
Age [years]
Supramarginal gyrus – rh
CNN: thick = 2.74 – 0.006 x age (p = 1.8e–12)
FS: thick = 2.71 – 0.005 x age (p = 8.0e–06)
CNN
FS
Thickness [mm]
Age [years]

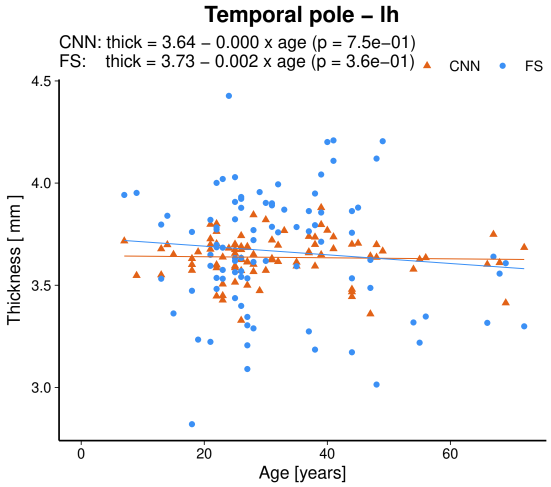
Temporal pole – lh
CNN: thick = 3.64 – 0.000 x age (p = 7.5e–01)
FS: thick = 3.73 – 0.002 x age (p = 3.6e–01)
CNN
FS
Thickness [mm]
Age [years]

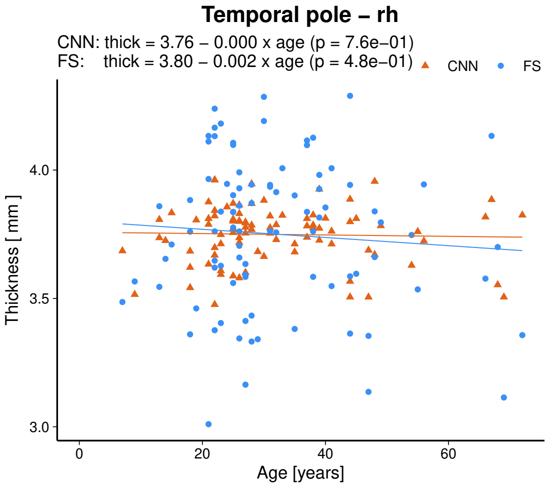
Temporal pole – rh
CNN: thick = 3.76 – 0.000 x age (p = 7.6e–01)
FS: thick = 3.80 – 0.002 x age (p = 4.8e–01)
CNN
FS
Thickness [mm]
Age [years]

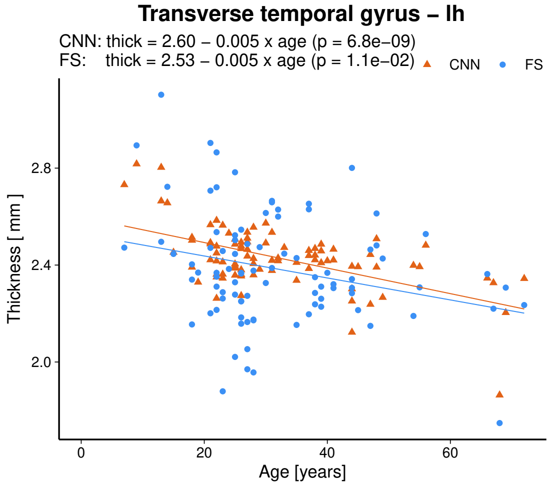
Transverse temporal gyrus – lh
CNN: thick = 2.60 – 0.005 x age (p = 6.8e–09)
FS: thick = 2.53 – 0.005 x age (p = 1.1e–02)
CNN
FS
Thickness [mm]
Age [years]

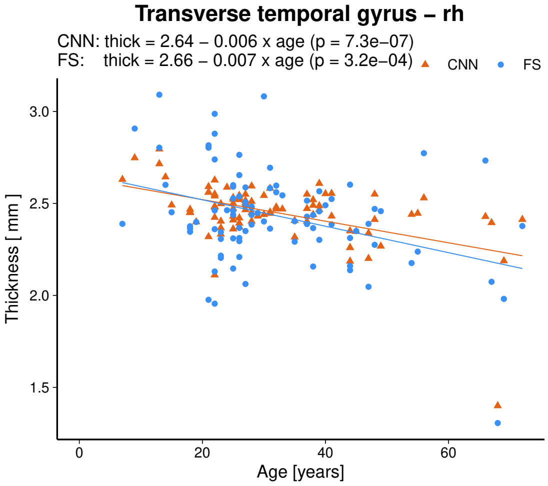
Transverse temporal gyrus – rh
CNN: thick = 2.64 – 0.006 x age (p = 7.3e–07)
FS: thick = 2.66 – 0.007 x age (p = 3.2e–04)
CNN
FS
Thickness [mm]
Age [years]

4 Reliability

Listed below are reliability plots by means of scan-rescan tests for all morphometrics. The leftmost bars depict the standard deviations (SD) across all 90 scans followed by the nine subjects with available rescans. In parenthesis: Number of scans per subject.

4.1 Reliability Plots for Subcortical Volume

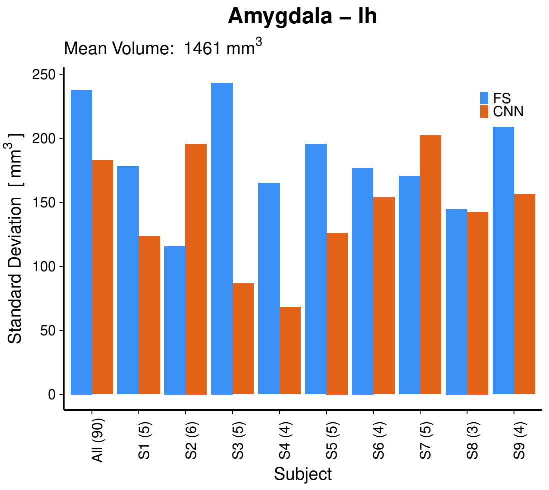

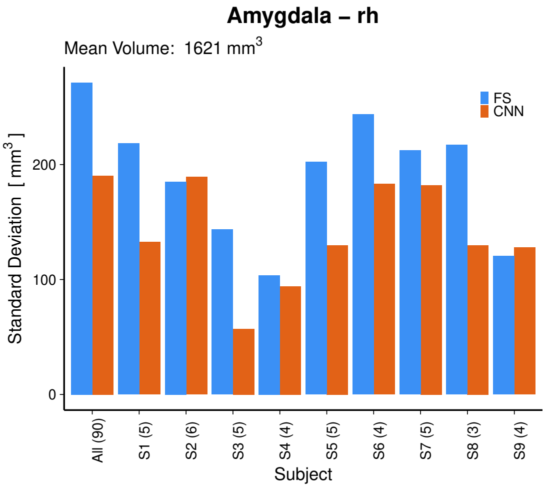

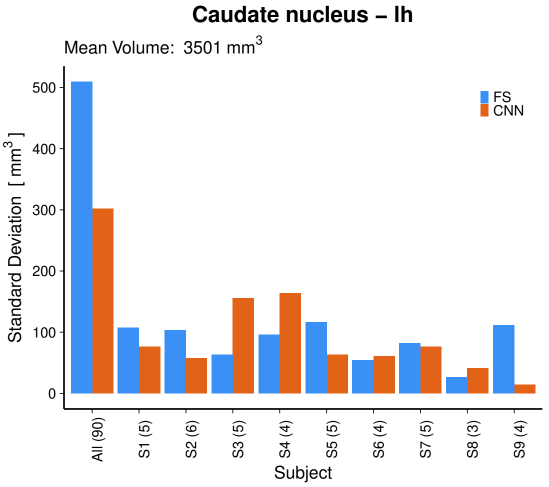

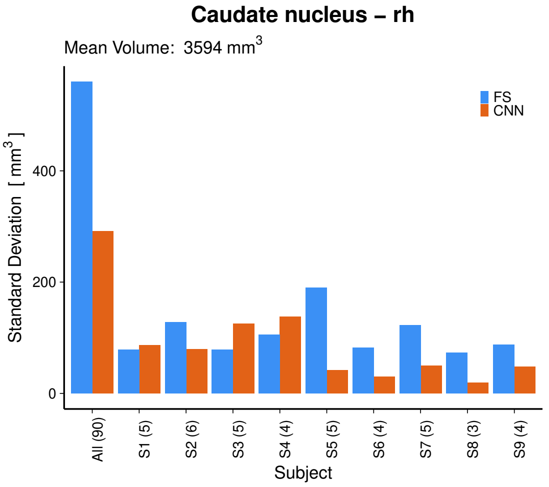

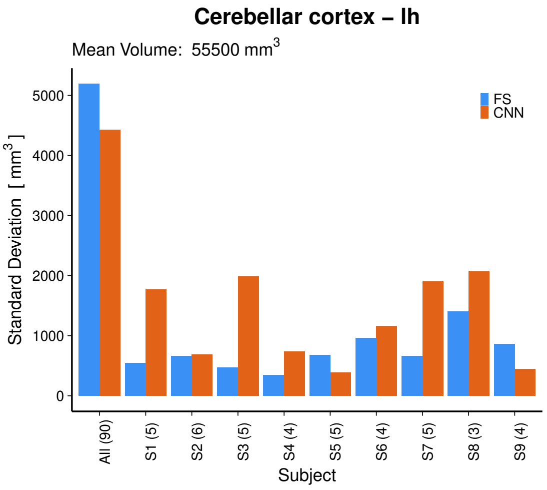
Cerebellar cortex – lh
Mean Volume: 55500 mm3
FS
CNN
Standard Deviation [mm3]
All (90)
S1 (5)
S2 (6)
S3 (5)
S4 (4)
S5 (5)
S6 (4)
S7 (5)
S8 (3)
S9 (4)
Subject

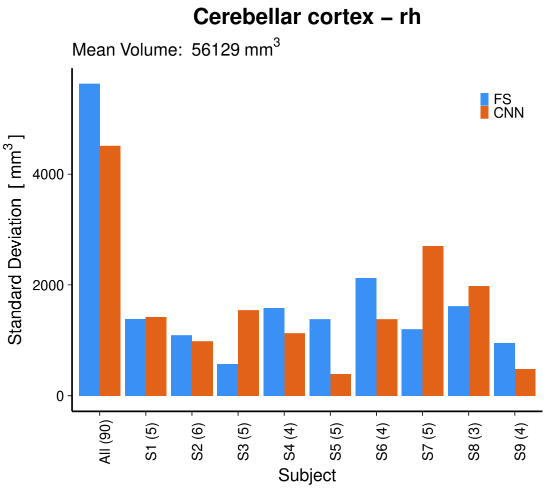
Cerebellar cortex – rh
Mean Volume: 56129 mm3
FS
CNN
Standard Deviation [mm3]
All (90)
S1 (5)
S2 (6)
S3 (5)
S4 (4)
S5 (5)
S6 (4)
S7 (5)
S8 (3)
S9 (4)
Subject

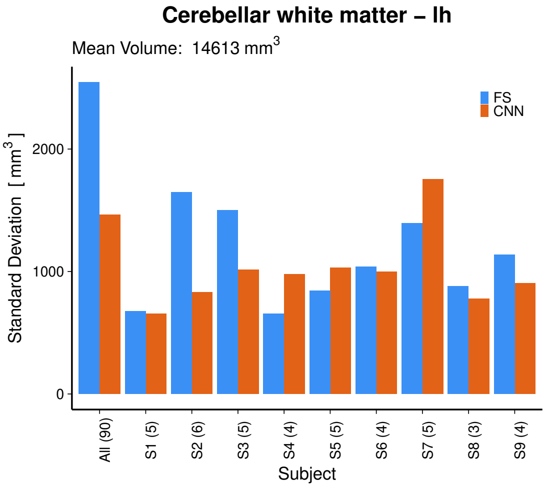
Cerebellar white matter – lh
Mean Volume: 14613 mm3
FS
CNN
Standard Deviation [mm3]
All (90)
S1 (5)
S2 (6)
S3 (5)
S4 (4)
S5 (5)
S6 (4)
S7 (5)
S8 (3)
S9 (4)
Subject

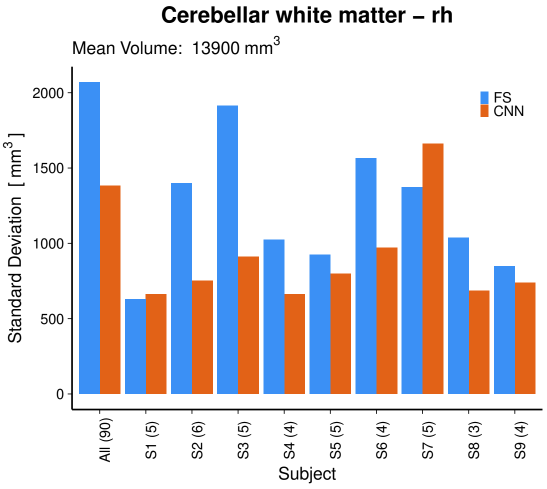
Cerebellar white matter – rh
Mean Volume: 13900 mm3
FS
CNN
Standard Deviation [mm3]
All (90)
S1 (5)
S2 (6)
S3 (5)
S4 (4)
S5 (5)
S6 (4)
S7 (5)
S8 (3)
S9 (4)
Subject

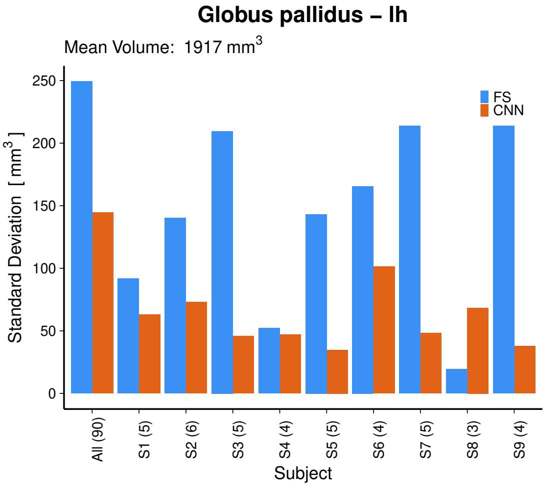
Globus pallidus – lh
Mean Volume: 1917 mm3
FS
CNN
Standard Deviation [mm3]
All (90)
S1 (5)
S2 (6)
S3 (5)
S4 (4)
S5 (5)
S6 (4)
S7 (5)
S8 (3)
S9 (4)
Subject

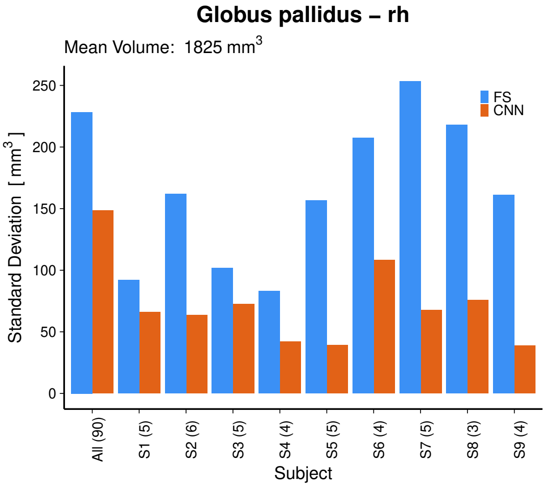
Globus pallidus – rh
Mean Volume: 1825 mm3
FS
CNN
Standard Deviation [mm3]
All (90)
S1 (5)
S2 (6)
S3 (5)
S4 (4)
S5 (5)
S6 (4)
S7 (5)
S8 (3)
S9 (4)
Subject

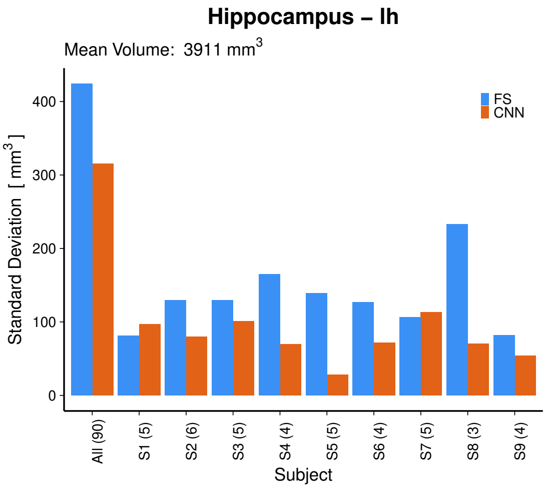
Hippocampus – lh
Mean Volume: 3911 mm3
Standard Deviation [mm3]
FS
CNN
All (90) S1 (5) S2 (6) S3 (5) S4 (4) S5 (5) S6 (4) S7 (5) S8 (3) S9 (4)
Subject

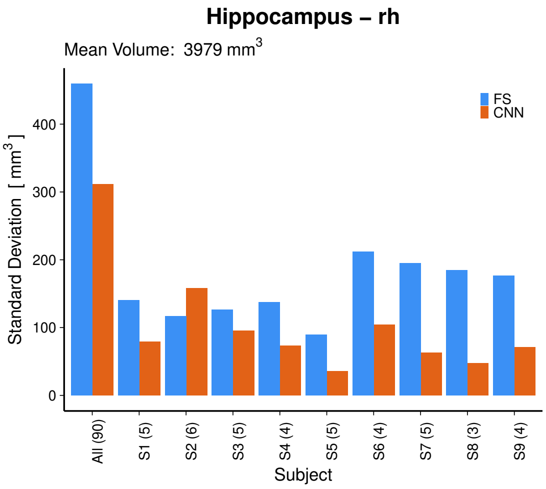
Hippocampus – rh
Mean Volume: 3979 mm3
Standard Deviation [mm3]
FS
CNN
All (90) S1 (5) S2 (6) S3 (5) S4 (4) S5 (5) S6 (4) S7 (5) S8 (3) S9 (4)
Subject

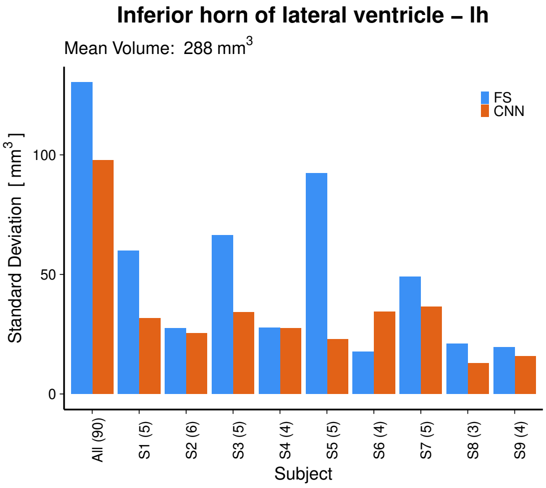
Inferior horn of lateral ventricle – lh
Mean Volume: 288 mm3
Standard Deviation [mm3]
FS
CNN
All (90) S1 (5) S2 (6) S3 (5) S4 (4) S5 (5) S6 (4) S7 (5) S8 (3) S9 (4)
Subject

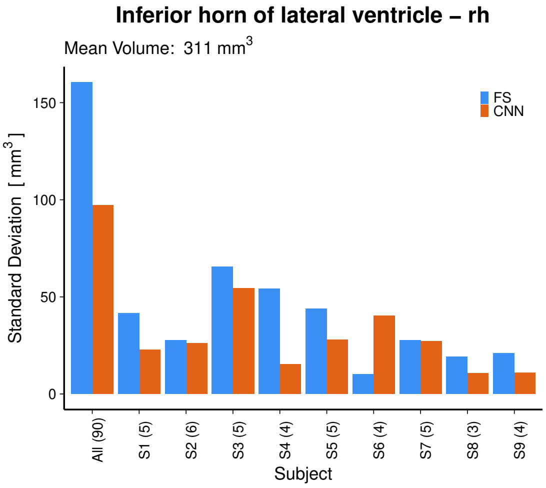
Inferior horn of lateral ventricle – rh
Mean Volume: 311 mm3
Standard Deviation [mm3]
FS
CNN
All (90) S1 (5) S2 (6) S3 (5) S4 (4) S5 (5) S6 (4) S7 (5) S8 (3) S9 (4)
Subject

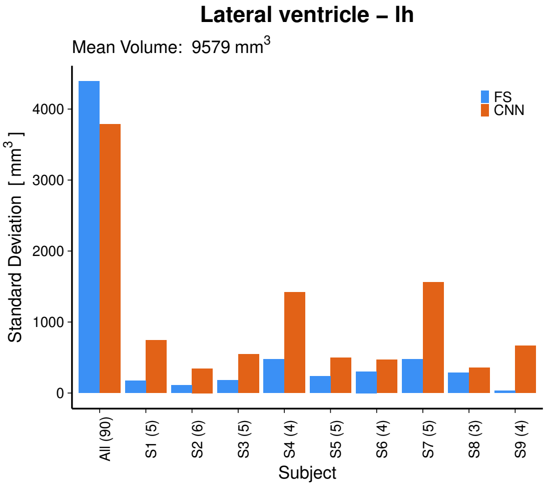
Lateral ventricle – lh
Mean Volume: 9579 mm3
Standard Deviation [mm3]
FS
CNN
All (90) S1 (5) S2 (6) S3 (5) S4 (4) S5 (5) S6 (4) S7 (5) S8 (3) S9 (4)
Subject

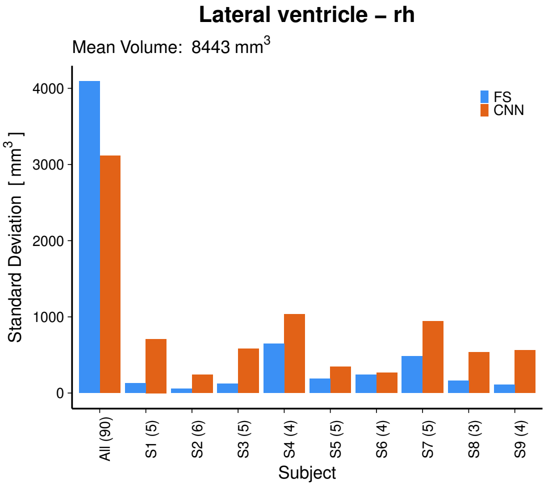
Lateral ventricle – rh
Mean Volume: 8443 mm3
Standard Deviation [mm3]
FS
CNN
All (90) S1 (5) S2 (6) S3 (5) S4 (4) S5 (5) S6 (4) S7 (5) S8 (3) S9 (4)
Subject

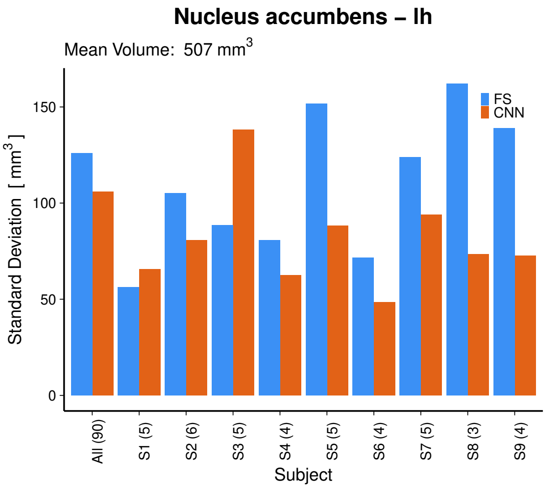

Nucleus accumbens – lh
Mean Volume: 507 mm3
Standard Deviation [mm3]
0
50
100
150
FS
CNN
All (90)
S1 (5)
S2 (6)
S3 (5)
S4 (4)
S5 (5)
S6 (4)
S7 (5)
S8 (3)
S9 (4)
Subject

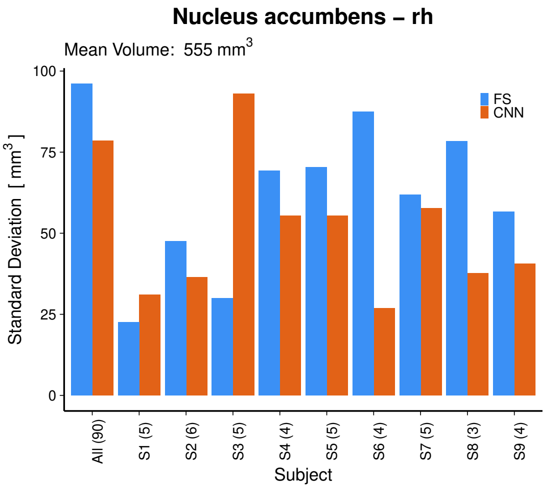

Nucleus accumbens – rh
Mean Volume: 555 mm3
Standard Deviation [mm3]
0
25
50
75
100
FS
CNN
All (90)
S1 (5)
S2 (6)
S3 (5)
S4 (4)
S5 (5)
S6 (4)
S7 (5)
S8 (3)
S9 (4)
Subject

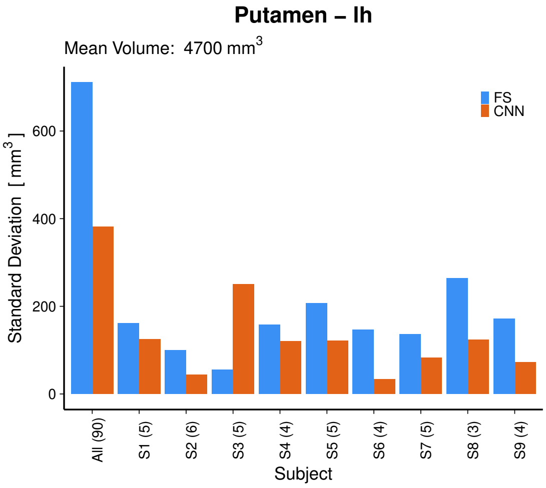

Putamen – lh
Mean Volume: 4700 mm3
Standard Deviation [mm3]
0
200
400
600
FS
CNN
All (90)
S1 (5)
S2 (6)
S3 (5)
S4 (4)
S5 (5)
S6 (4)
S7 (5)
S8 (3)
S9 (4)
Subject

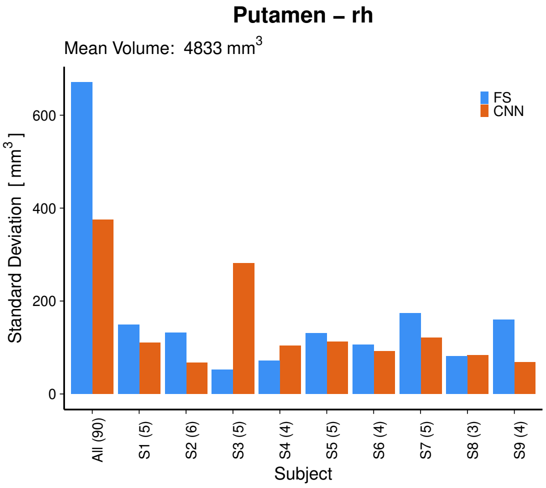

Putamen – rh
Mean Volume: 4833 mm3
Standard Deviation [mm3]
0
200
400
600
FS
CNN
All (90)
S1 (5)
S2 (6)
S3 (5)
S4 (4)
S5 (5)
S6 (4)
S7 (5)
S8 (3)
S9 (4)
Subject

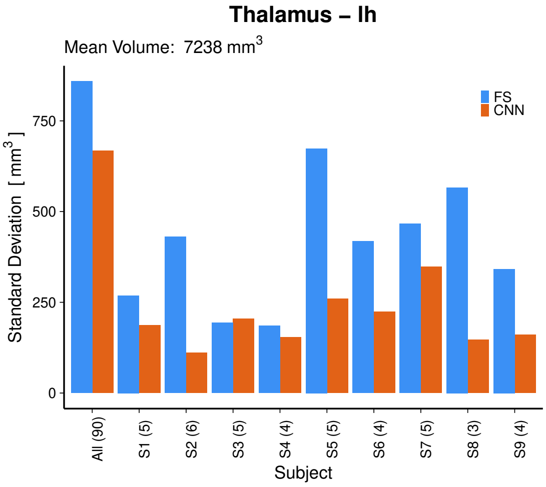

Thalamus – lh
Mean Volume: 7238 mm3
Standard Deviation [mm3]
0
250
500
750
FS
CNN
All (90)
S1 (5)
S2 (6)
S3 (5)
S4 (4)
S5 (5)
S6 (4)
S7 (5)
S8 (3)
S9 (4)
Subject

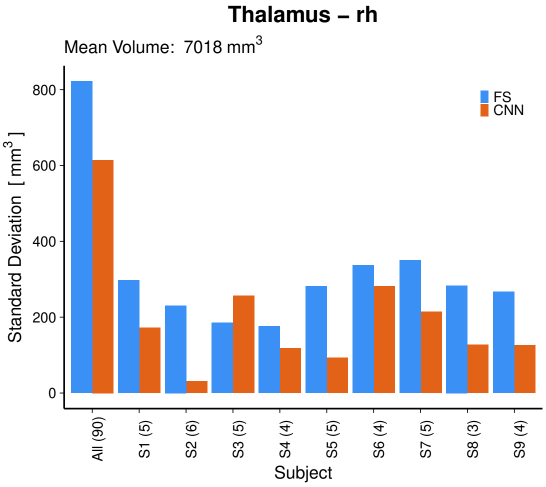

Thalamus – rh
Mean Volume: 7018 mm3
Standard Deviation [mm3]
0
200
400
600
800
FS
CNN
All (90)
S1 (5)
S2 (6)
S3 (5)
S4 (4)
S5 (5)
S6 (4)
S7 (5)
S8 (3)
S9 (4)
Subject

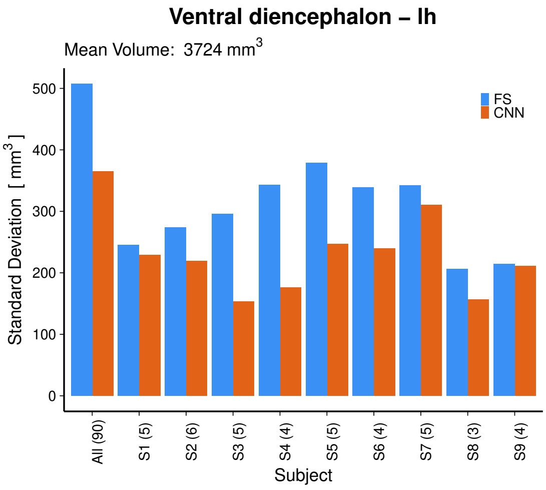
Ventral diencephalon – lh
Mean Volume: 3724 mm3
Standard Deviation [mm3]
FS
CNN
All (90)
S1 (5)
S2 (6)
S3 (5)
S4 (4)
S5 (5)
S6 (4)
S7 (5)
S8 (3)
S9 (4)
Subject

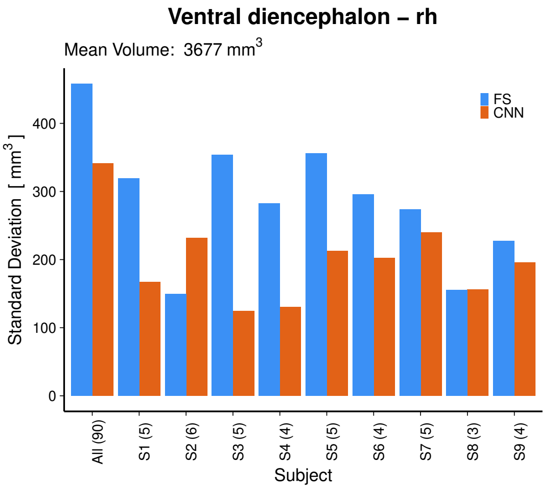
Ventral diencephalon – rh
Mean Volume: 3677 mm3
Standard Deviation [mm3]
FS
CNN
Subject

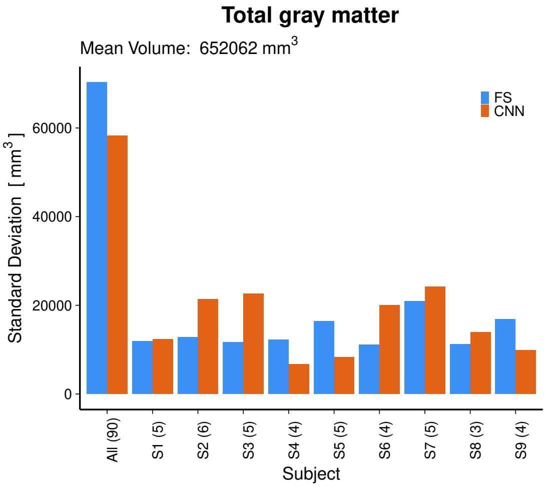
Total gray matter
Mean Volume: 652062 mm3
Standard Deviation [mm3]
FS
CNN
Subject

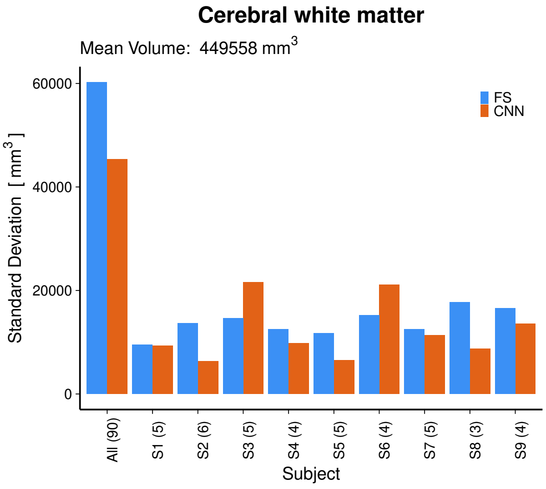
Cerebral white matter
Mean Volume: 449558 mm3
Standard Deviation [mm3]
FS
CNN
Subject

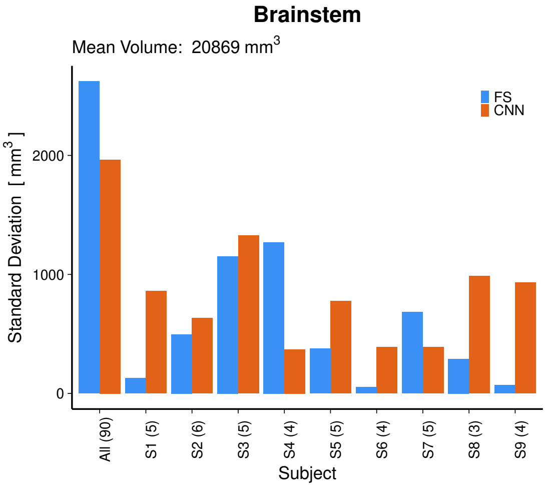
Brainstem
Mean Volume: 20869 mm3
Standard Deviation [mm3]
FS
CNN
Subject

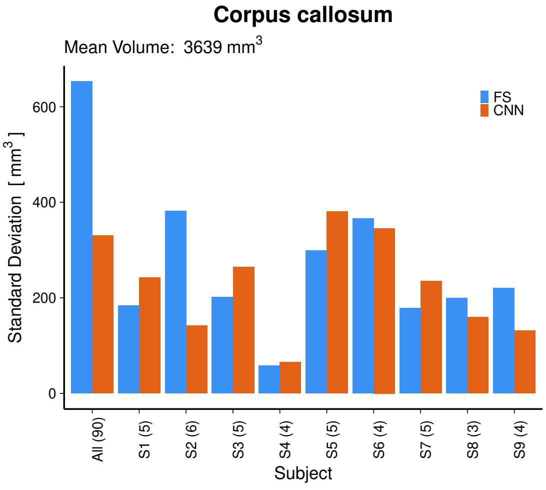
Corpus callosum
Mean Volume: 3639 mm3
Standard Deviation [mm3]
FS
CNN
Subject

4.2 Reliability Plots for Cortical Thickness

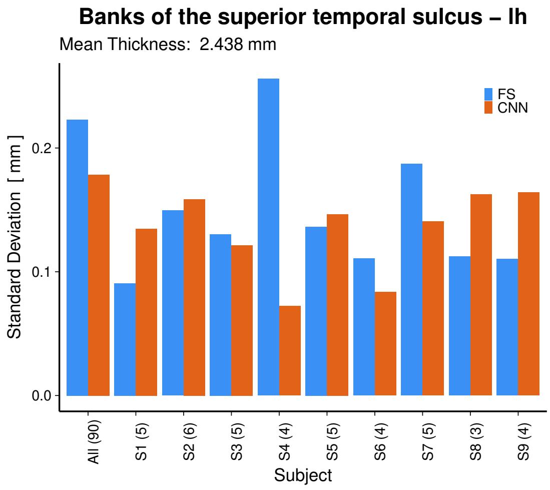

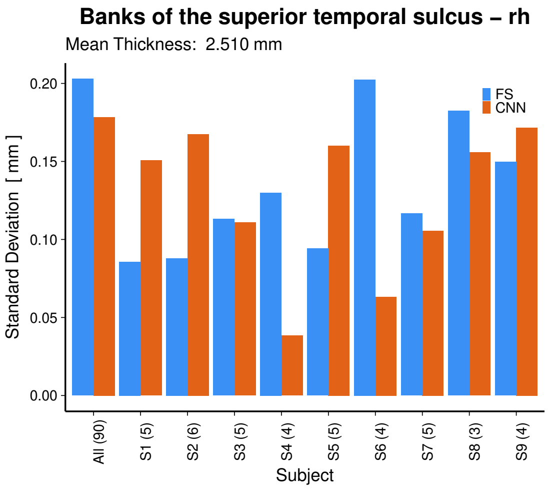

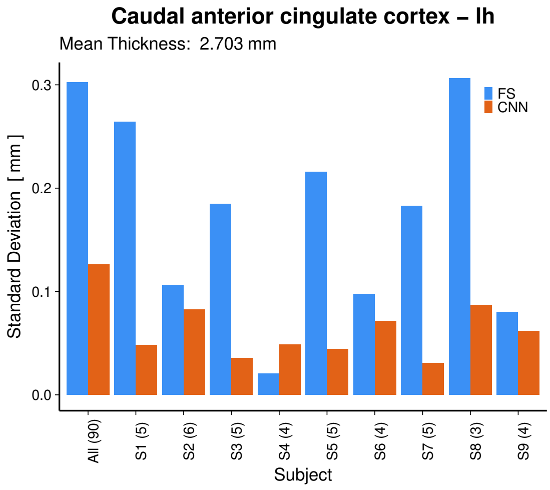

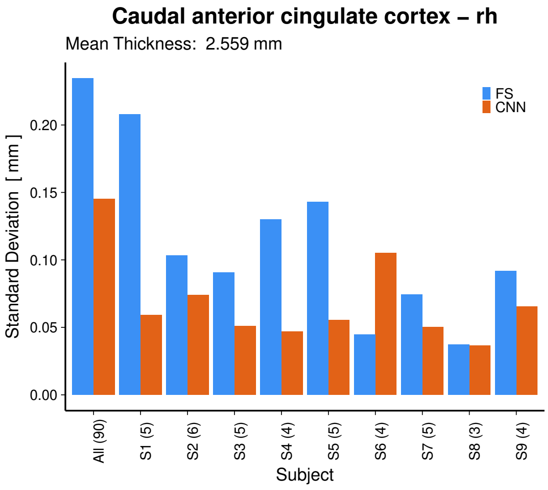

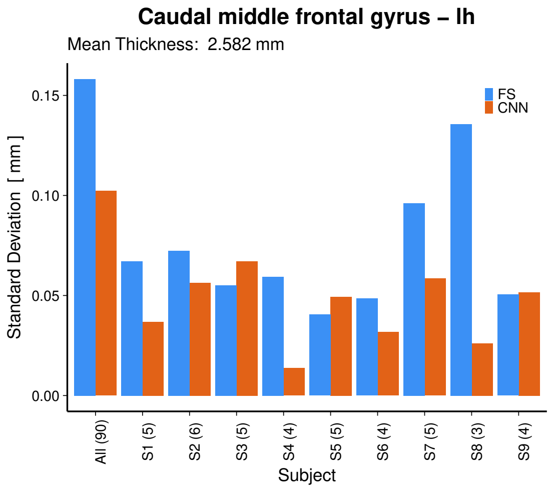
Caudal middle frontal gyrus – lh
Mean Thickness: 2.582 mm
Standard Deviation [mm]
0.00
0.05
0.10
0.15
FS
CNN
All (90)
S1 (5)
S2 (6)
S3 (5)
S4 (4)
S5 (5)
S6 (4)
S7 (5)
S8 (3)
S9 (4)
Subject

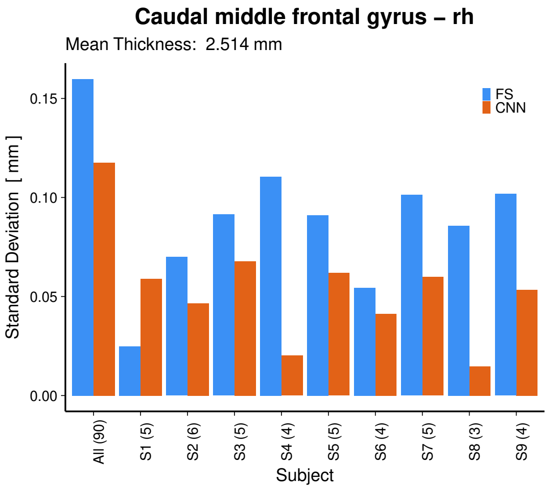
Caudal middle frontal gyrus – rh
Mean Thickness: 2.514 mm
Standard Deviation [mm]
0.00
0.05
0.10
0.15
FS
CNN
All (90)
S1 (5)
S2 (6)
S3 (5)
S4 (4)
S5 (5)
S6 (4)
S7 (5)
S8 (3)
S9 (4)
Subject

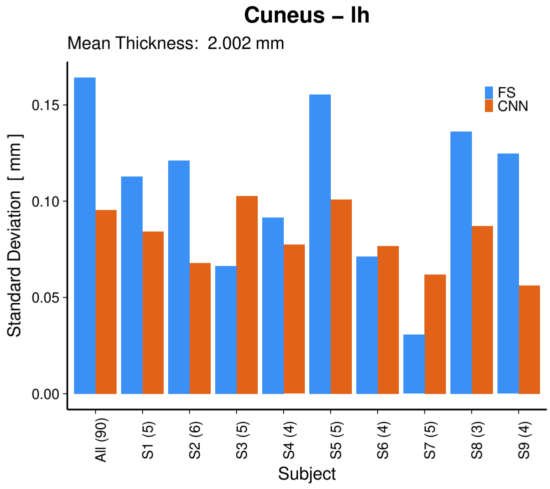
Cuneus – lh
Mean Thickness: 2.002 mm
Standard Deviation [mm]
0.00
0.05
0.10
0.15
FS
CNN
All (90)
S1 (5)
S2 (6)
S3 (5)
S4 (4)
S5 (5)
S6 (4)
S7 (5)
S8 (3)
S9 (4)
Subject

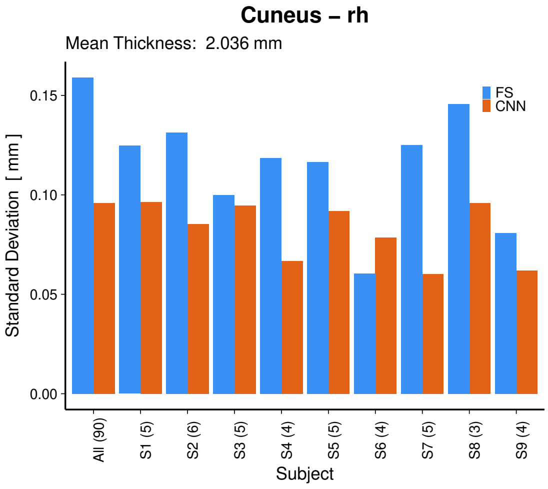
Cuneus – rh
Mean Thickness: 2.036 mm
Standard Deviation [mm]
0.00
0.05
0.10
0.15
FS
CNN
All (90)
S1 (5)
S2 (6)
S3 (5)
S4 (4)
S5 (5)
S6 (4)
S7 (5)
S8 (3)
S9 (4)
Subject

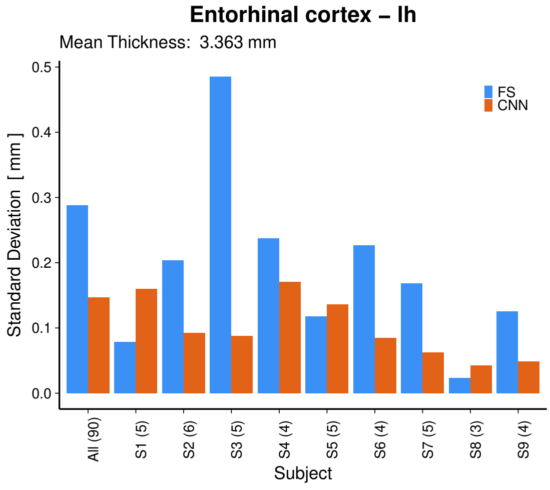
Entorhinal cortex – lh
Mean Thickness: 3.363 mm
Standard Deviation [mm]
0.0
0.1
0.2
0.3
0.4
0.5
FS
CNN
All (90)
S1 (5)
S2 (6)
S3 (5)
S4 (4)
S5 (5)
S6 (4)
S7 (5)
S8 (3)
S9 (4)
Subject

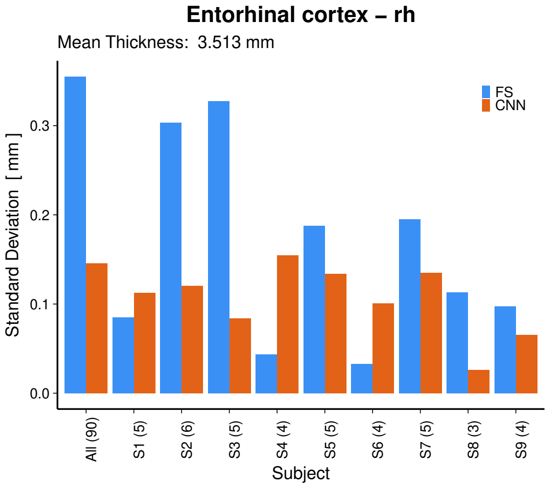
Entorhinal cortex – rh
Mean Thickness: 3.513 mm
Standard Deviation [mm]
0.0
0.1
0.2
0.3
FS
CNN
All (90)
S1 (5)
S2 (6)
S3 (5)
S4 (4)
S5 (5)
S6 (4)
S7 (5)
S8 (3)
S9 (4)
Subject

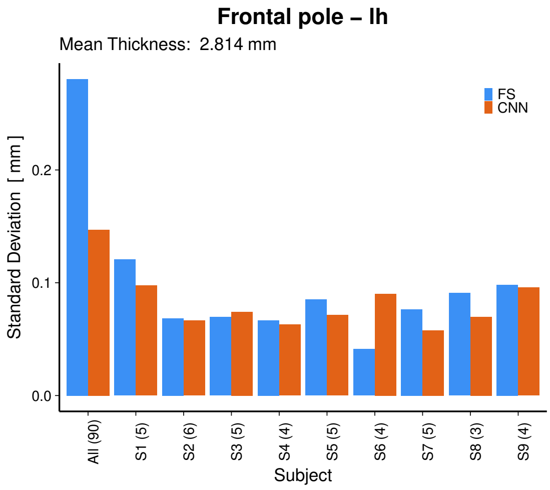

Frontal pole – lh
Mean Thickness: 2.814 mm
Standard Deviation [mm]
FS
CNN
All (90)
S1 (5)
S2 (6)
S3 (5)
S4 (4)
S5 (5)
S6 (4)
S7 (5)
S8 (3)
S9 (4)
Subject

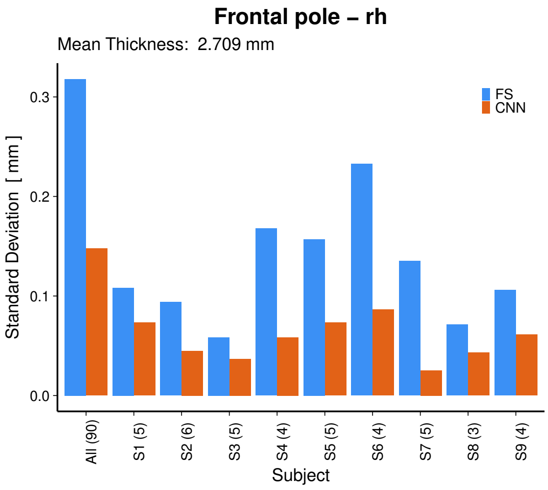

Frontal pole – rh
Mean Thickness: 2.709 mm
Standard Deviation [mm]
FS
CNN
Subject

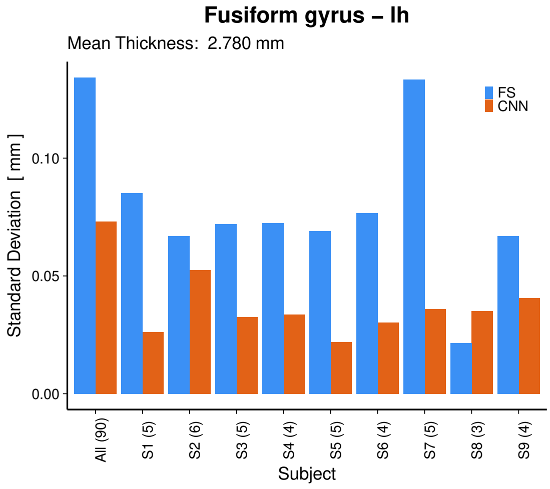

Fusiform gyrus – lh
Mean Thickness: 2.780 mm
Standard Deviation [mm]
FS
CNN
Subject

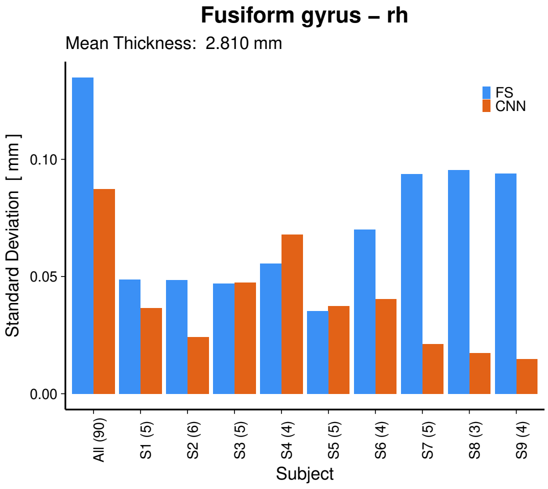

Fusiform gyrus – rh
Mean Thickness: 2.810 mm
Standard Deviation [mm]
FS
CNN
Subject

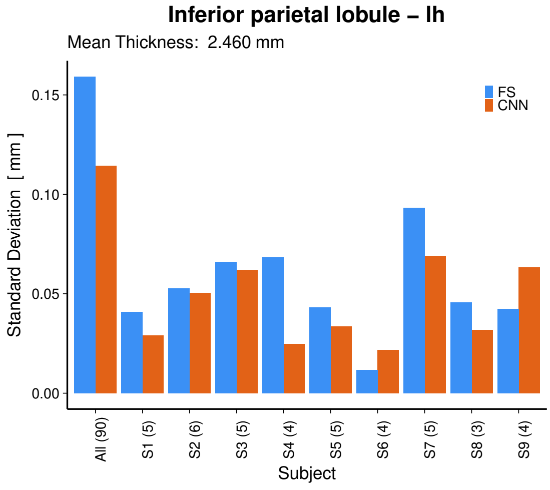

Inferior parietal lobule – lh
Mean Thickness: 2.460 mm
Standard Deviation [mm]
FS
CNN
Subject

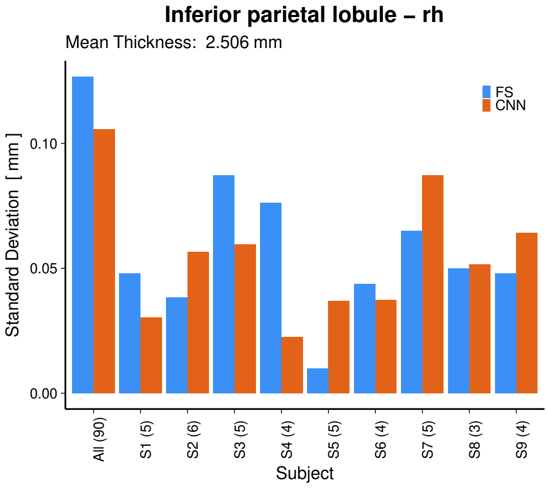

Inferior parietal lobule – rh
Mean Thickness: 2.506 mm
Standard Deviation [mm]
FS
CNN
Subject

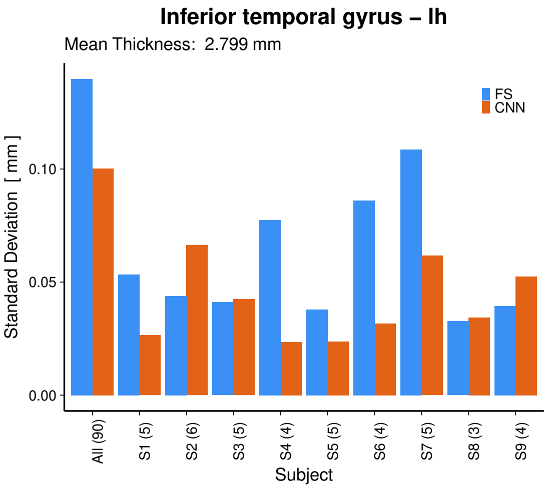
Inferior temporal gyrus – lh
Mean Thickness: 2.799 mm
FS
CNN
Standard Deviation [mm]
0.00
0.05
0.10
All (90)
S1 (5)
S2 (6)
S3 (5)
S4 (4)
S5 (5)
S6 (4)
S7 (5)
S8 (3)
S9 (4)
Subject

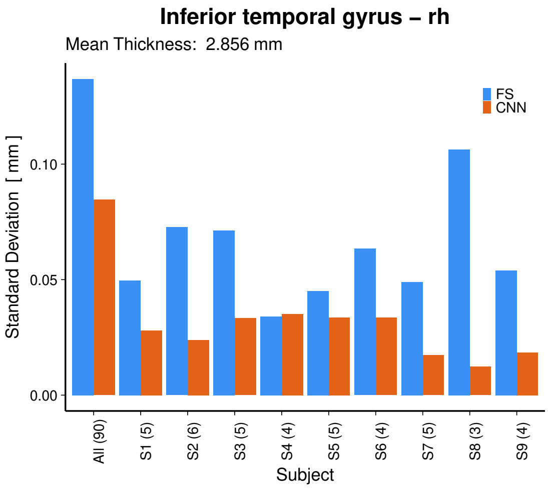
Inferior temporal gyrus – rh
Mean Thickness: 2.856 mm
FS
CNN
Standard Deviation [mm]
0.00
0.05
0.10
All (90)
S1 (5)
S2 (6)
S3 (5)
S4 (4)
S5 (5)
S6 (4)
S7 (5)
S8 (3)
S9 (4)
Subject

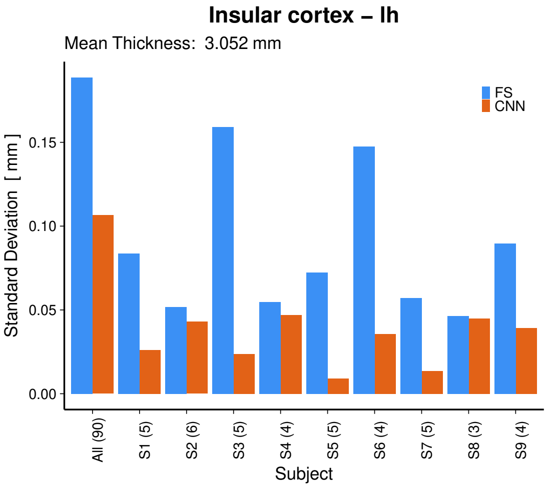
Insular cortex – lh
Mean Thickness: 3.052 mm
FS
CNN
Standard Deviation [mm]
0.00
0.05
0.10
0.15
All (90)
S1 (5)
S2 (6)
S3 (5)
S4 (4)
S5 (5)
S6 (4)
S7 (5)
S8 (3)
S9 (4)
Subject

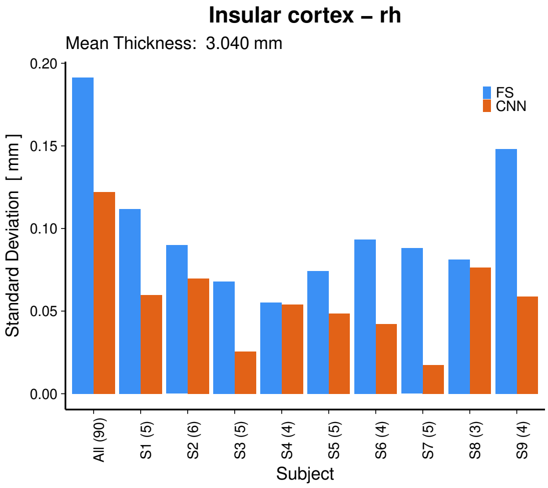
Insular cortex – rh
Mean Thickness: 3.040 mm
FS
CNN
Standard Deviation [mm]
0.00
0.05
0.10
0.15
0.20
All (90)
S1 (5)
S2 (6)
S3 (5)
S4 (4)
S5 (5)
S6 (4)
S7 (5)
S8 (3)
S9 (4)
Subject

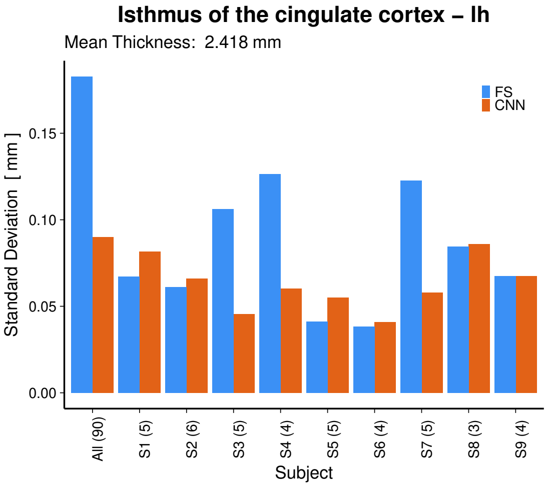
Isthmus of the cingulate cortex – lh
Mean Thickness: 2.418 mm
FS
CNN
Standard Deviation [mm]
0.00
0.05
0.10
0.15
All (90)
S1 (5)
S2 (6)
S3 (5)
S4 (4)
S5 (5)
S6 (4)
S7 (5)
S8 (3)
S9 (4)
Subject

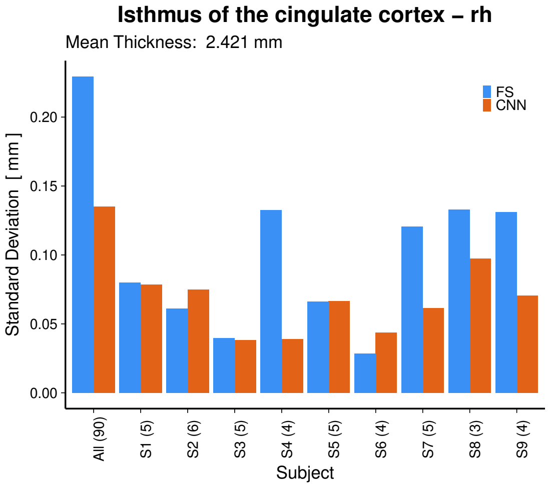
Isthmus of the cingulate cortex – rh
Mean Thickness: 2.421 mm
FS
CNN
Standard Deviation [mm]
0.00
0.05
0.10
0.15
0.20
All (90)
S1 (5)
S2 (6)
S3 (5)
S4 (4)
S5 (5)
S6 (4)
S7 (5)
S8 (3)
S9 (4)
Subject

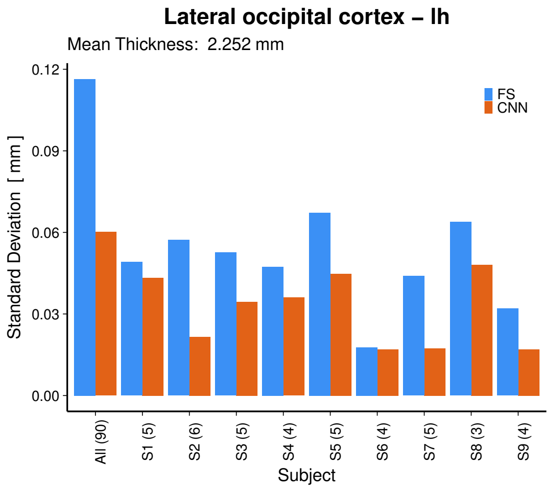
Lateral occipital cortex – lh
Mean Thickness: 2.252 mm
Standard Deviation [mm]
0.12
0.09
0.06
0.03
0.00
FS
CNN
All (90)
S1 (5)
S2 (6)
S3 (5)
S4 (4)
S5 (5)
S6 (4)
S7 (5)
S8 (3)
S9 (4)
Subject

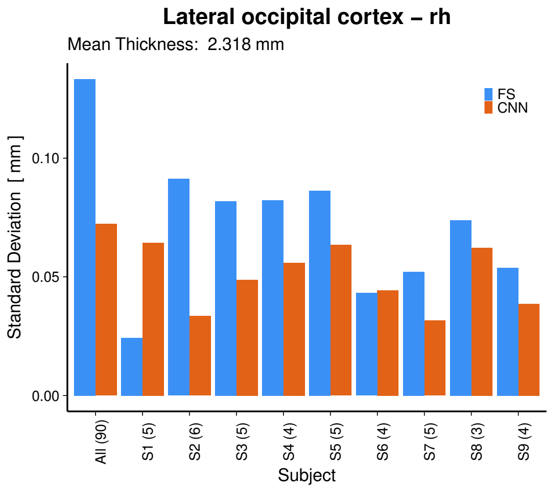
Lateral occipital cortex – rh
Mean Thickness: 2.318 mm
Standard Deviation [mm]
0.10
0.05
0.00
FS
CNN
All (90)
S1 (5)
S2 (6)
S3 (5)
S4 (4)
S5 (5)
S6 (4)
S7 (5)
S8 (3)
S9 (4)
Subject

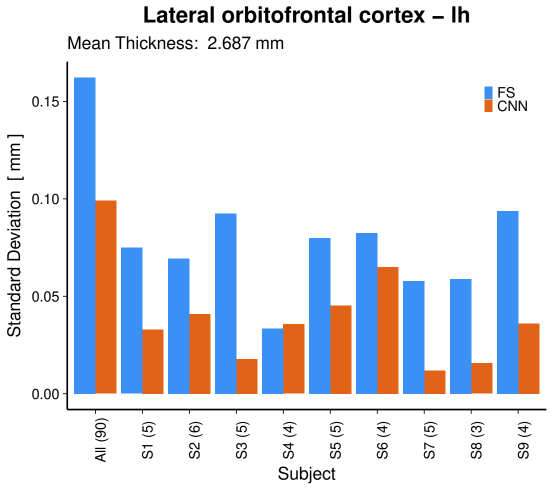
Lateral orbitofrontal cortex – lh
Mean Thickness: 2.687 mm
Standard Deviation [mm]
0.15
0.10
0.05
0.00
FS
CNN
All (90)
S1 (5)
S2 (6)
S3 (5)
S4 (4)
S5 (5)
S6 (4)
S7 (5)
S8 (3)
S9 (4)
Subject

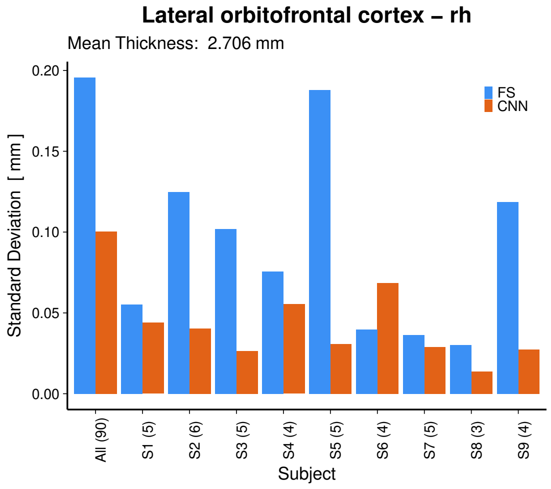
Lateral orbitofrontal cortex – rh
Mean Thickness: 2.706 mm
Standard Deviation [mm]
0.20
0.15
0.10
0.05
0.00
FS
CNN
All (90)
S1 (5)
S2 (6)
S3 (5)
S4 (4)
S5 (5)
S6 (4)
S7 (5)
S8 (3)
S9 (4)
Subject

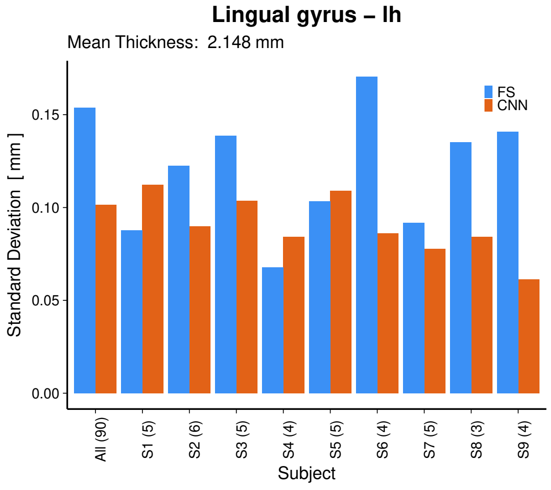
Lingual gyrus – lh
Mean Thickness: 2.148 mm
Standard Deviation [mm]
0.15
0.10
0.05
0.00
FS
CNN
All (90)
S1 (5)
S2 (6)
S3 (5)
S4 (4)
S5 (5)
S6 (4)
S7 (5)
S8 (3)
S9 (4)
Subject

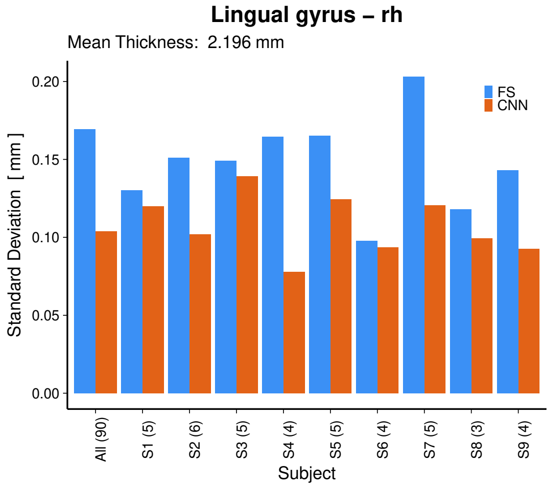
Lingual gyrus – rh
Mean Thickness: 2.196 mm
Standard Deviation [mm]
0.20
0.15
0.10
0.05
0.00
FS
CNN
All (90)
S1 (5)
S2 (6)
S3 (5)
S4 (4)
S5 (5)
S6 (4)
S7 (5)
S8 (3)
S9 (4)
Subject

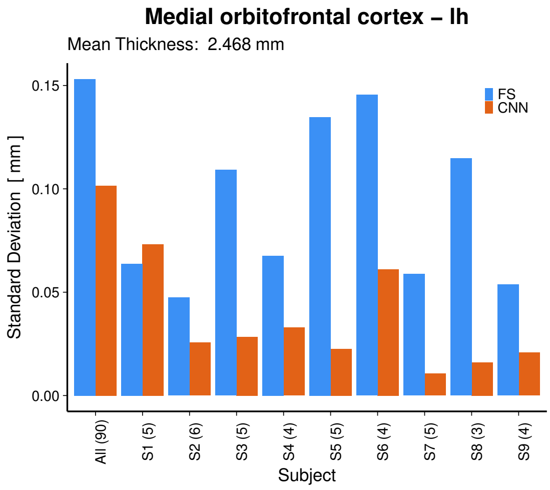
Medial orbitofrontal cortex – lh
Mean Thickness: 2.468 mm
Standard Deviation [mm]
FS
CNN
All (90)
S1 (5)
S2 (6)
S3 (5)
S4 (4)
S5 (5)
S6 (4)
S7 (5)
S8 (3)
S9 (4)
Subject

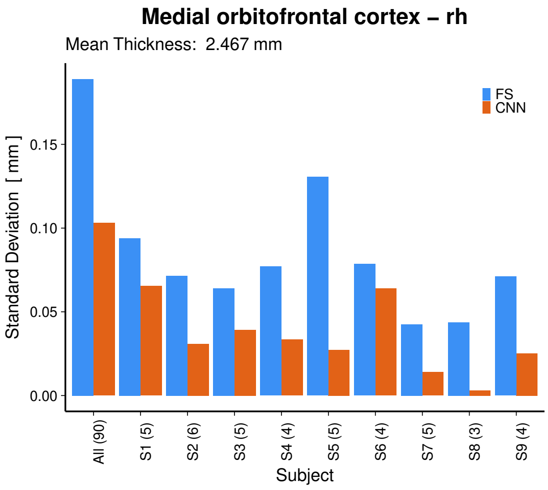
Medial orbitofrontal cortex – rh
Mean Thickness: 2.467 mm
Standard Deviation [mm]
FS
CNN
Subject

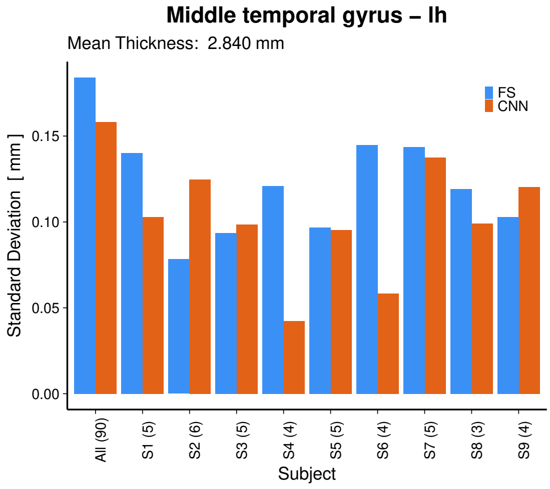
Middle temporal gyrus – lh
Mean Thickness: 2.840 mm
Standard Deviation [mm]
FS
CNN
Subject

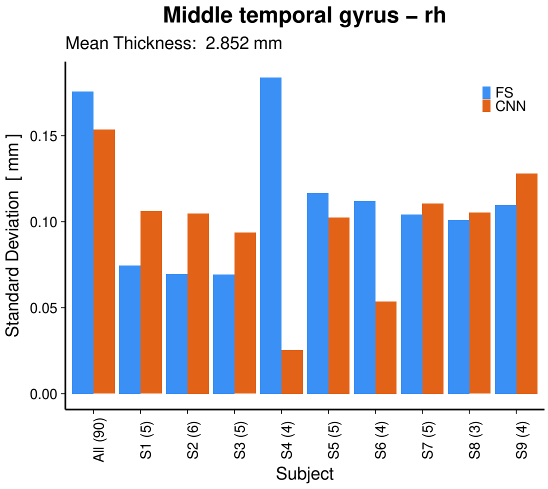
Middle temporal gyrus – rh
Mean Thickness: 2.852 mm
Standard Deviation [mm]
FS
CNN
Subject

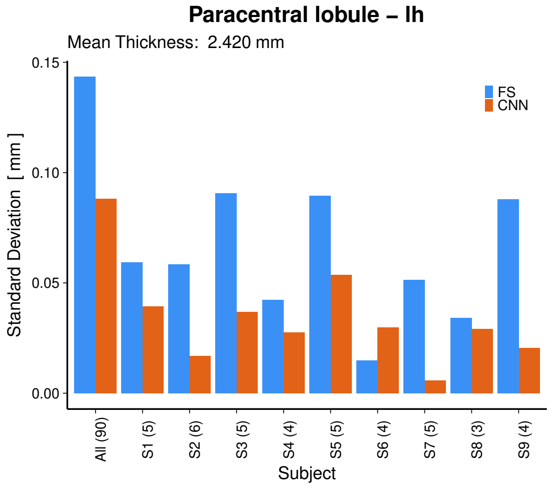
Paracentral lobule – lh
Mean Thickness: 2.420 mm
Standard Deviation [mm]
FS
CNN
Subject

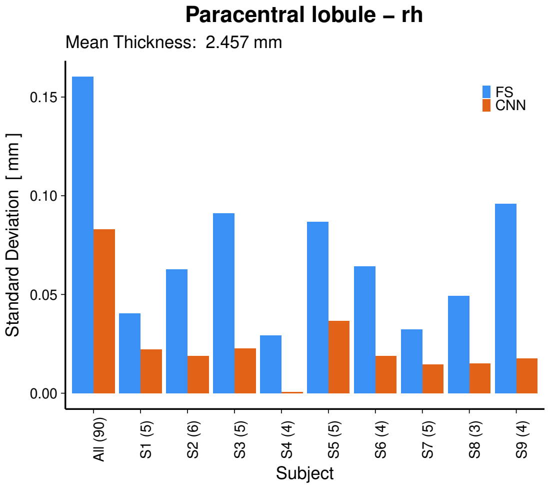
Paracentral lobule – rh
Mean Thickness: 2.457 mm
Standard Deviation [mm]
FS
CNN
Subject

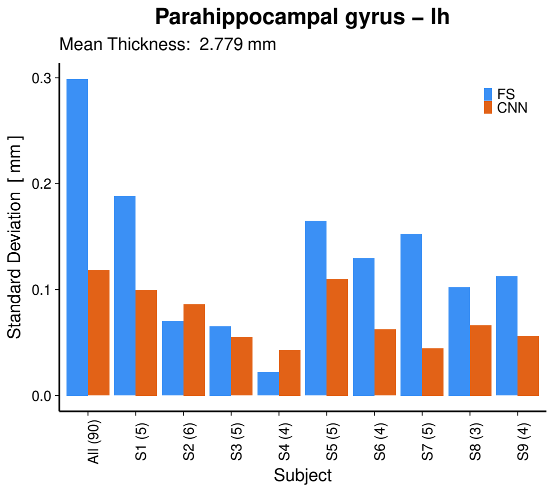
Parahippocampal gyrus – lh
Mean Thickness: 2.779 mm
Standard Deviation [mm]
Subject
FS
CNN
All (90)
S1 (5)
S2 (6)
S3 (5)
S4 (4)
S5 (5)
S6 (4)
S7 (5)
S8 (3)
S9 (4)

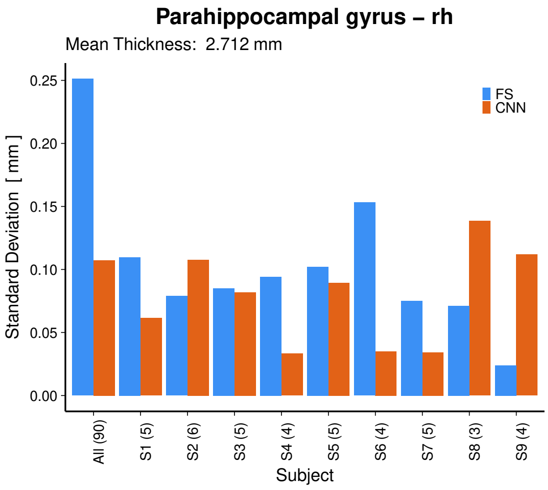
Parahippocampal gyrus – rh
Mean Thickness: 2.712 mm
Standard Deviation [mm]
Subject
FS
CNN
All (90)
S1 (5)
S2 (6)
S3 (5)
S4 (4)
S5 (5)
S6 (4)
S7 (5)
S8 (3)
S9 (4)

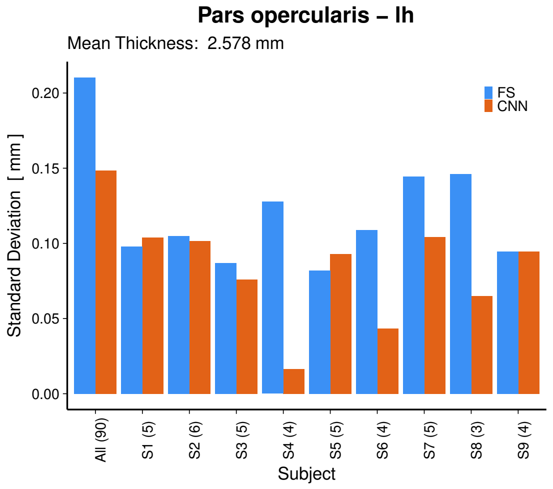
Pars opercularis – lh
Mean Thickness: 2.578 mm
Standard Deviation [mm]
Subject
FS
CNN
All (90)
S1 (5)
S2 (6)
S3 (5)
S4 (4)
S5 (5)
S6 (4)
S7 (5)
S8 (3)
S9 (4)

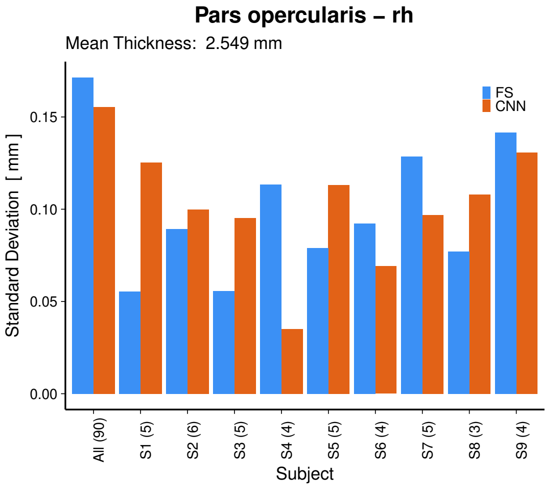
Pars opercularis – rh
Mean Thickness: 2.549 mm
Standard Deviation [mm]
Subject
FS
CNN
All (90)
S1 (5)
S2 (6)
S3 (5)
S4 (4)
S5 (5)
S6 (4)
S7 (5)
S8 (3)
S9 (4)

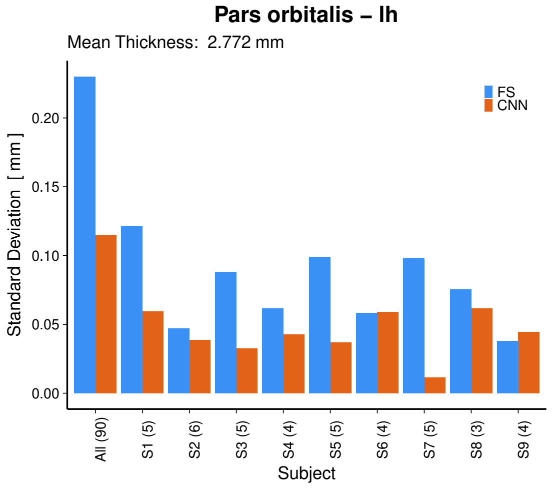
Pars orbitalis – lh
Mean Thickness: 2.772 mm
Standard Deviation [mm]
Subject
FS
CNN
All (90)
S1 (5)
S2 (6)
S3 (5)
S4 (4)
S5 (5)
S6 (4)
S7 (5)
S8 (3)
S9 (4)

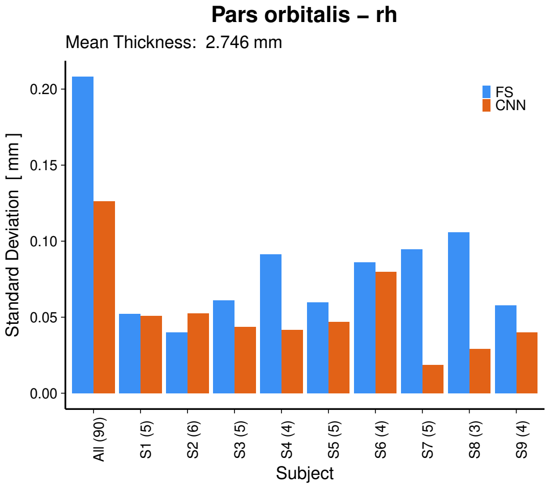
Pars orbitalis – rh
Mean Thickness: 2.746 mm
Standard Deviation [mm]
Subject
FS
CNN
All (90)
S1 (5)
S2 (6)
S3 (5)
S4 (4)
S5 (5)
S6 (4)
S7 (5)
S8 (3)
S9 (4)

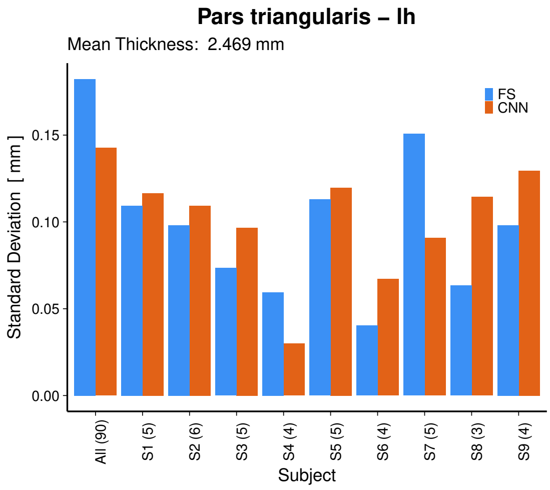
Pars triangularis – lh
Mean Thickness: 2.469 mm
FS
CNN
Standard Deviation [mm]
0.00
0.05
0.10
0.15
All (90)
S1 (5)
S2 (6)
S3 (5)
S4 (4)
S5 (5)
S6 (4)
S7 (5)
S8 (3)
S9 (4)
Subject

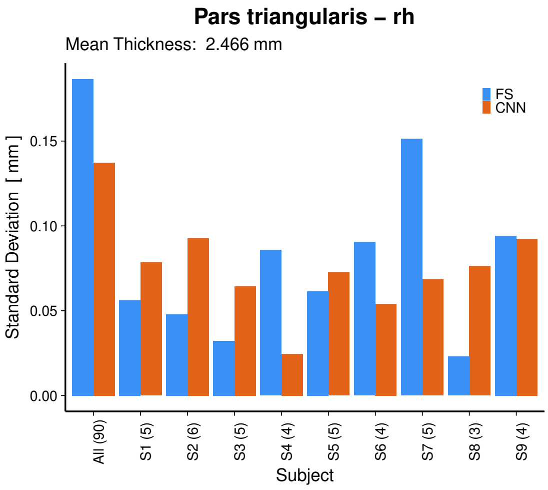
Pars triangularis – rh
Mean Thickness: 2.466 mm
FS
CNN
Standard Deviation [mm]
0.00
0.05
0.10
0.15
All (90)
S1 (5)
S2 (6)
S3 (5)
S4 (4)
S5 (5)
S6 (4)
S7 (5)
S8 (3)
S9 (4)
Subject

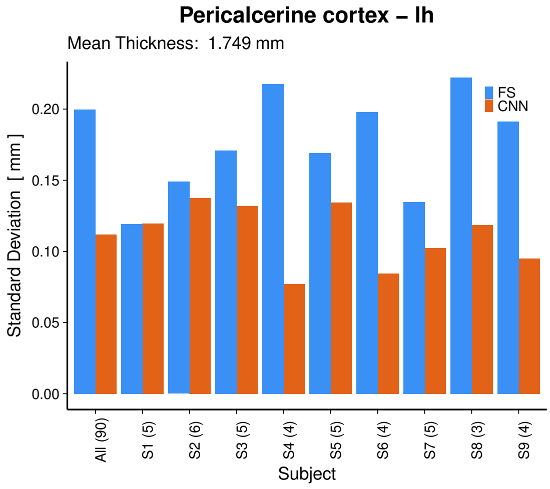
Pericalcerine cortex – lh
Mean Thickness: 1.749 mm
FS
CNN
Standard Deviation [mm]
0.00
0.05
0.10
0.15
0.20
All (90)
S1 (5)
S2 (6)
S3 (5)
S4 (4)
S5 (5)
S6 (4)
S7 (5)
S8 (3)
S9 (4)
Subject

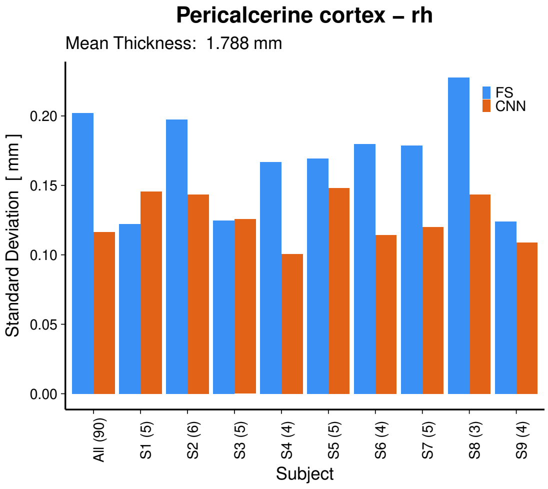
Pericalcerine cortex – rh
Mean Thickness: 1.788 mm
FS
CNN
Standard Deviation [mm]
0.00
0.05
0.10
0.15
0.20
All (90)
S1 (5)
S2 (6)
S3 (5)
S4 (4)
S5 (5)
S6 (4)
S7 (5)
S8 (3)
S9 (4)
Subject

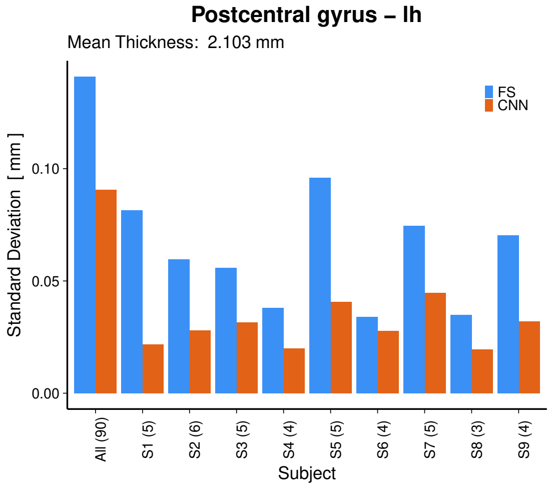
Postcentral gyrus – lh
Mean Thickness: 2.103 mm
FS
CNN
Standard Deviation [mm]
0.00
0.05
0.10
All (90)
S1 (5)
S2 (6)
S3 (5)
S4 (4)
S5 (5)
S6 (4)
S7 (5)
S8 (3)
S9 (4)
Subject

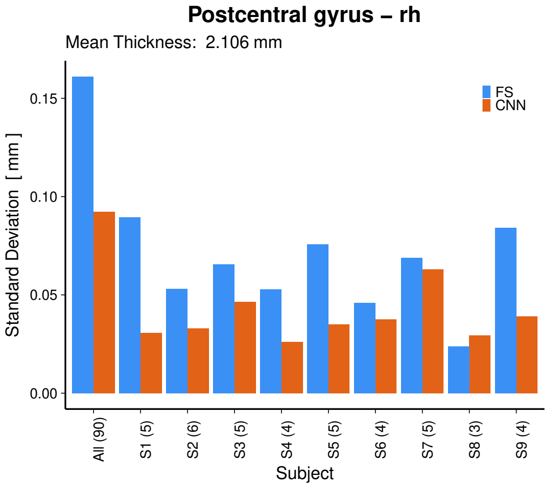
Postcentral gyrus – rh
Mean Thickness: 2.106 mm
FS
CNN
Standard Deviation [mm]
0.00
0.05
0.10
0.15
All (90)
S1 (5)
S2 (6)
S3 (5)
S4 (4)
S5 (5)
S6 (4)
S7 (5)
S8 (3)
S9 (4)
Subject

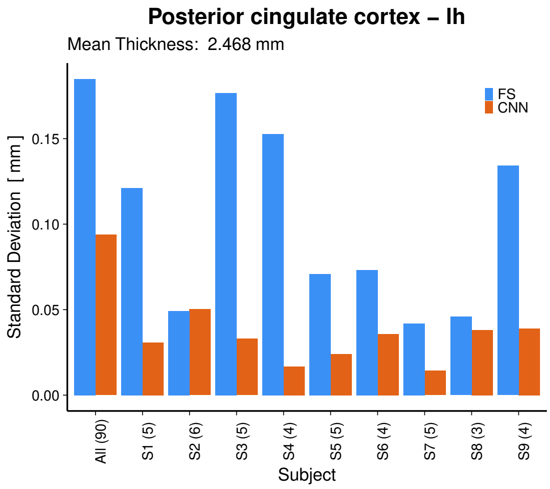
Posterior cingulate cortex – lh
Mean Thickness: 2.468 mm
Standard Deviation [mm]
0.00
0.05
0.10
0.15
FS
CNN
All (90)
S1 (5)
S2 (6)
S3 (5)
S4 (4)
S5 (5)
S6 (4)
S7 (5)
S8 (3)
S9 (4)
Subject

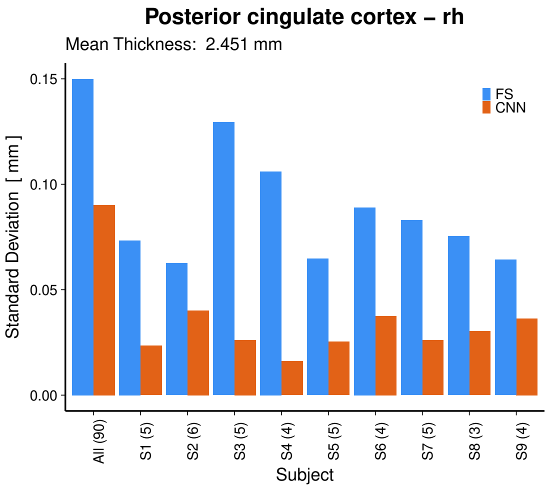
Posterior cingulate cortex – rh
Mean Thickness: 2.451 mm
Standard Deviation [mm]
0.00
0.05
0.10
0.15
FS
CNN
All (90)
S1 (5)
S2 (6)
S3 (5)
S4 (4)
S5 (5)
S6 (4)
S7 (5)
S8 (3)
S9 (4)
Subject

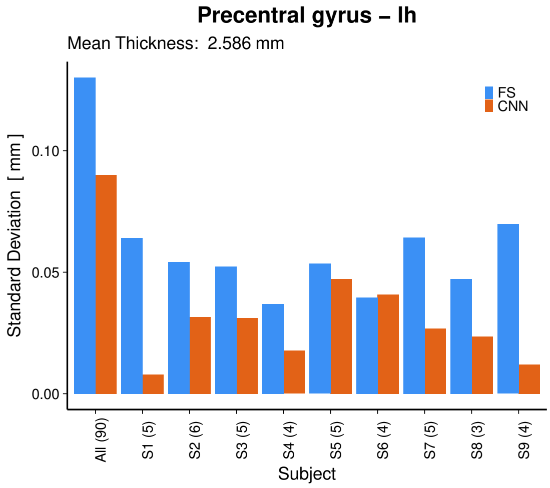
Precentral gyrus – lh
Mean Thickness: 2.586 mm
Standard Deviation [mm]
0.00
0.05
0.10
FS
CNN
All (90)
S1 (5)
S2 (6)
S3 (5)
S4 (4)
S5 (5)
S6 (4)
S7 (5)
S8 (3)
S9 (4)
Subject

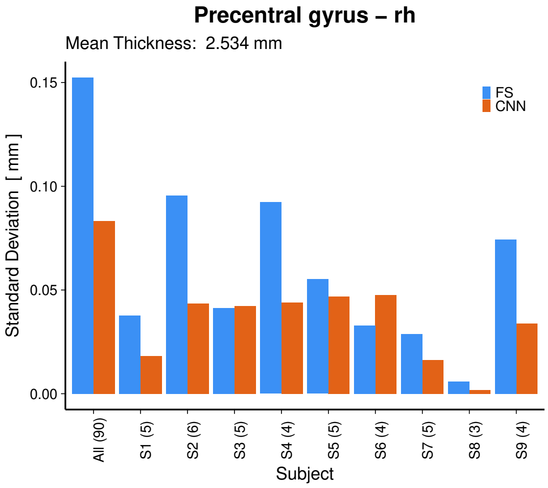
Precentral gyrus – rh
Mean Thickness: 2.534 mm
Standard Deviation [mm]
0.00
0.05
0.10
0.15
FS
CNN
All (90)
S1 (5)
S2 (6)
S3 (5)
S4 (4)
S5 (5)
S6 (4)
S7 (5)
S8 (3)
S9 (4)
Subject

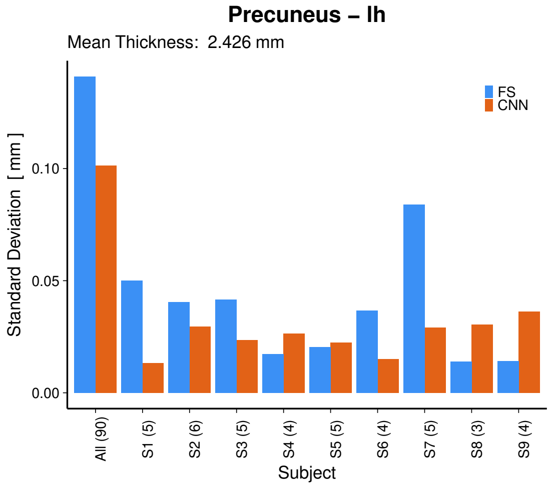
Precuneus – lh
Mean Thickness: 2.426 mm
Standard Deviation [mm]
0.00
0.05
0.10
FS
CNN
All (90)
S1 (5)
S2 (6)
S3 (5)
S4 (4)
S5 (5)
S6 (4)
S7 (5)
S8 (3)
S9 (4)
Subject

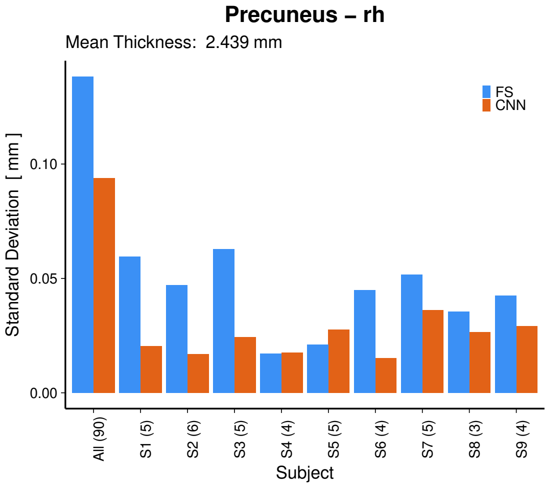
Precuneus – rh
Mean Thickness: 2.439 mm
Standard Deviation [mm]
0.00
0.05
0.10
FS
CNN
All (90)
S1 (5)
S2 (6)
S3 (5)
S4 (4)
S5 (5)
S6 (4)
S7 (5)
S8 (3)
S9 (4)
Subject

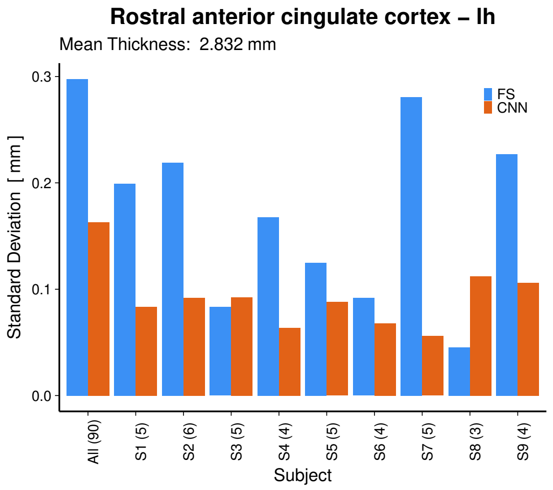
Rostral anterior cingulate cortex – lh
Mean Thickness: 2.832 mm
FS
CNN
Standard Deviation [mm]
Subject
All (90)
S1 (5)
S2 (6)
S3 (5)
S4 (4)
S5 (5)
S6 (4)
S7 (5)
S8 (3)
S9 (4)

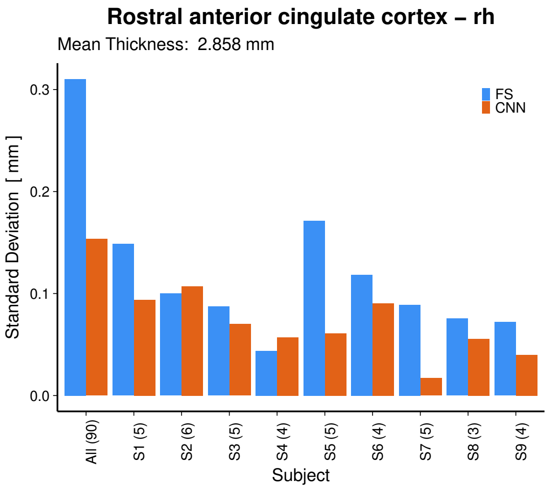
Rostral anterior cingulate cortex – rh
Mean Thickness: 2.858 mm
FS
CNN
Standard Deviation [mm]
Subject

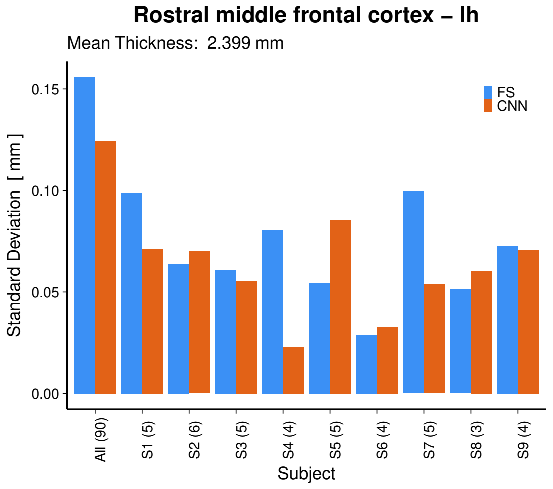
Rostral middle frontal cortex – lh
Mean Thickness: 2.399 mm
FS
CNN
Standard Deviation [mm]
Subject

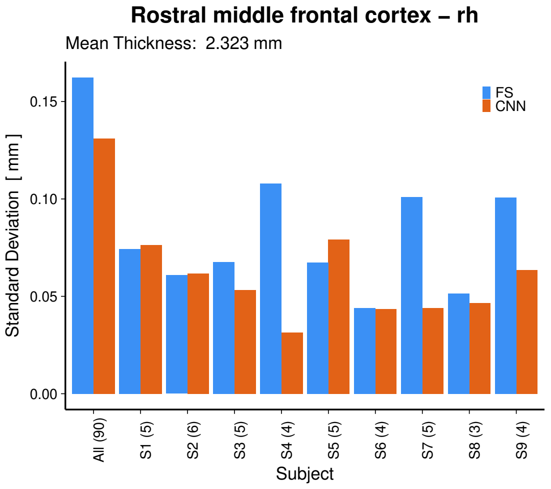
Rostral middle frontal cortex – rh
Mean Thickness: 2.323 mm
FS
CNN
Standard Deviation [mm]
Subject

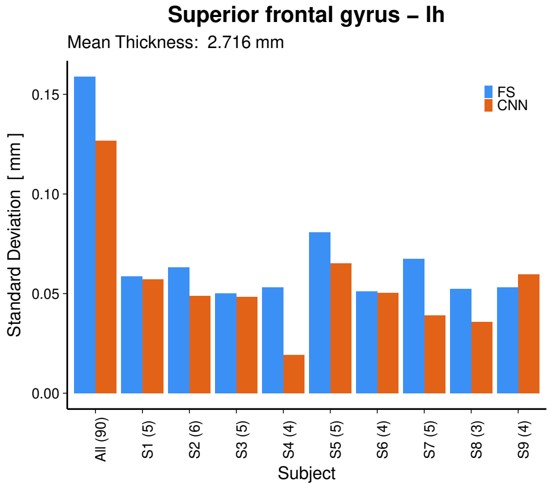
Superior frontal gyrus – lh
Mean Thickness: 2.716 mm
FS
CNN
Standard Deviation [mm]
Subject

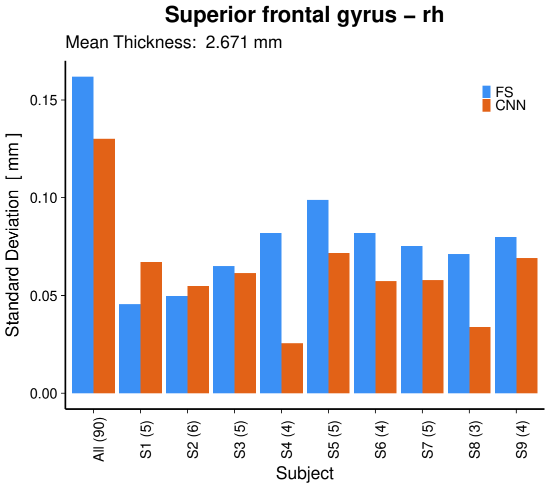
Superior frontal gyrus – rh
Mean Thickness: 2.671 mm
FS
CNN
Standard Deviation [mm]
Subject

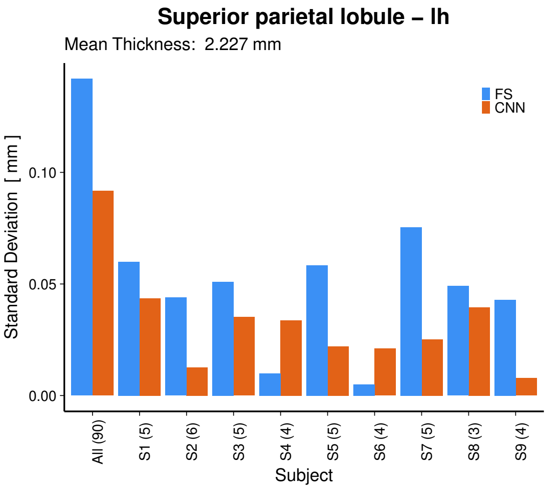
Superior parietal lobule – lh
Mean Thickness: 2.227 mm
Standard Deviation [mm]
0.00
0.05
0.10
FS
CNN
All (90)
S1 (5)
S2 (6)
S3 (5)
S4 (4)
S5 (5)
S6 (4)
S7 (5)
S8 (3)
S9 (4)
Subject

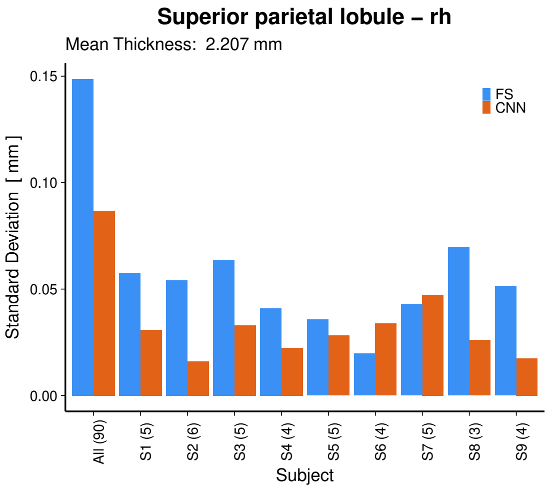
Superior parietal lobule – rh
Mean Thickness: 2.207 mm
Standard Deviation [mm]
0.00
0.05
0.10
0.15
FS
CNN
All (90)
S1 (5)
S2 (6)
S3 (5)
S4 (4)
S5 (5)
S6 (4)
S7 (5)
S8 (3)
S9 (4)
Subject

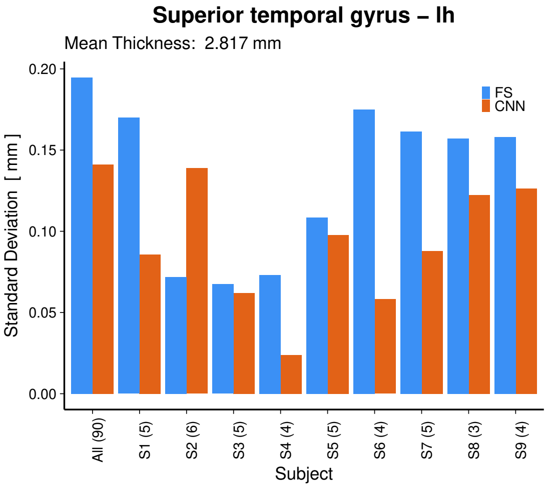
Superior temporal gyrus – lh
Mean Thickness: 2.817 mm
Standard Deviation [mm]
0.00
0.05
0.10
0.15
0.20
FS
CNN
All (90)
S1 (5)
S2 (6)
S3 (5)
S4 (4)
S5 (5)
S6 (4)
S7 (5)
S8 (3)
S9 (4)
Subject

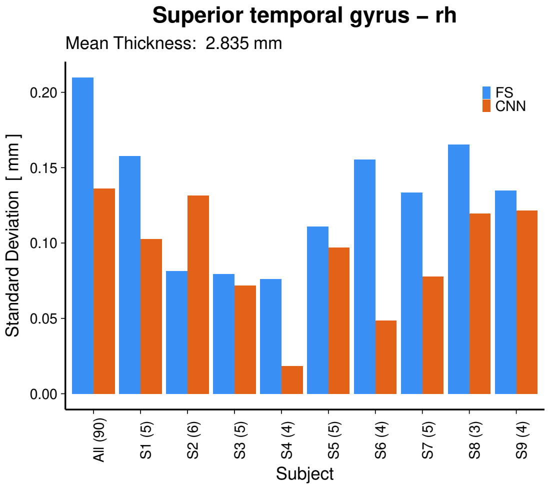
Superior temporal gyrus – rh
Mean Thickness: 2.835 mm
Standard Deviation [mm]
0.00
0.05
0.10
0.15
0.20
FS
CNN
All (90)
S1 (5)
S2 (6)
S3 (5)
S4 (4)
S5 (5)
S6 (4)
S7 (5)
S8 (3)
S9 (4)
Subject

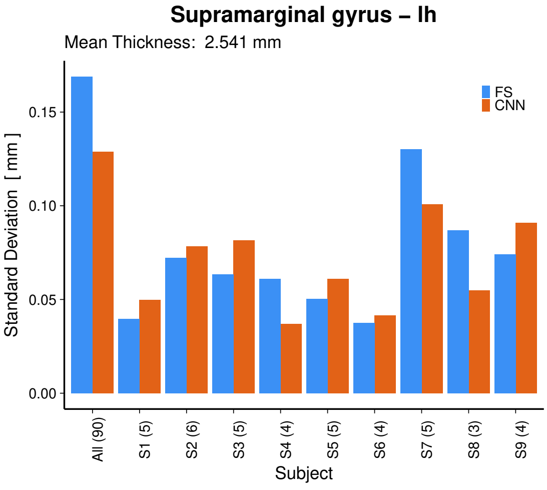
Supramarginal gyrus – lh
Mean Thickness: 2.541 mm
Standard Deviation [mm]
0.00
0.05
0.10
0.15
FS
CNN
All (90)
S1 (5)
S2 (6)
S3 (5)
S4 (4)
S5 (5)
S6 (4)
S7 (5)
S8 (3)
S9 (4)
Subject

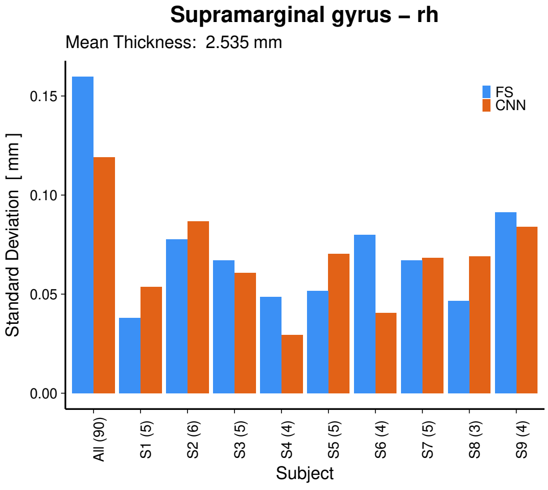
Supramarginal gyrus – rh
Mean Thickness: 2.535 mm
Standard Deviation [mm]
0.00
0.05
0.10
0.15
FS
CNN
All (90)
S1 (5)
S2 (6)
S3 (5)
S4 (4)
S5 (5)
S6 (4)
S7 (5)
S8 (3)
S9 (4)
Subject

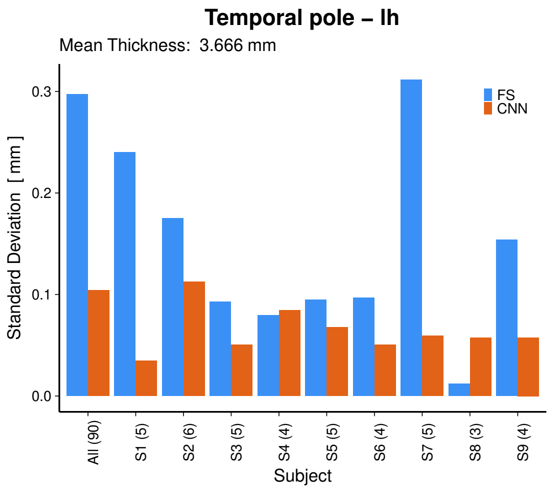
Temporal pole – lh
Mean Thickness: 3.666 mm
FS
CNN
Standard Deviation [mm]
0.0
0.1
0.2
0.3
All (90)
S1 (5)
S2 (6)
S3 (5)
S4 (4)
S5 (5)
S6 (4)
S7 (5)
S8 (3)
S9 (4)
Subject

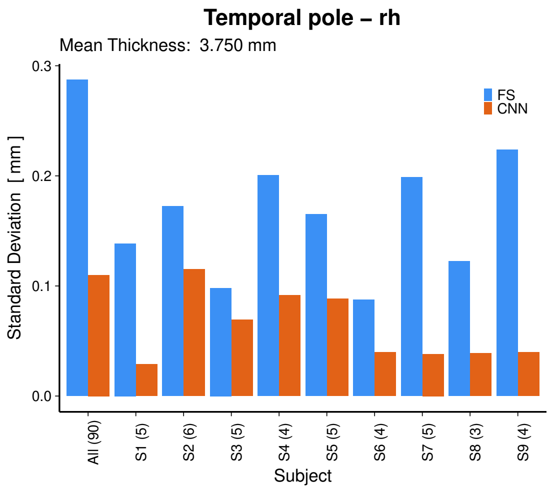
Temporal pole – rh
Mean Thickness: 3.750 mm
FS
CNN
Standard Deviation [mm]
0.0
0.1
0.2
0.3
All (90)
S1 (5)
S2 (6)
S3 (5)
S4 (4)
S5 (5)
S6 (4)
S7 (5)
S8 (3)
S9 (4)
Subject

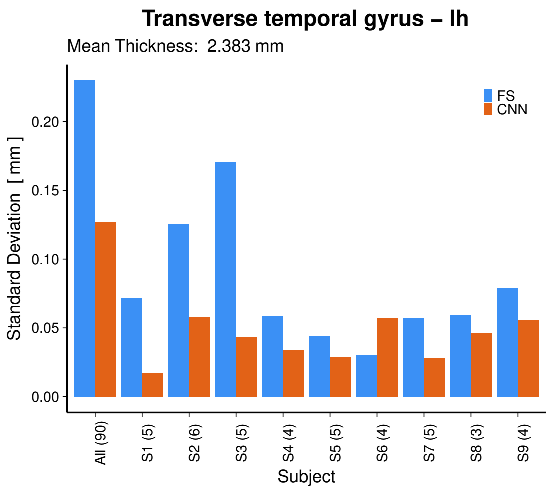
Transverse temporal gyrus – lh
Mean Thickness: 2.383 mm
FS
CNN
Standard Deviation [mm]
0.00
0.05
0.10
0.15
0.20
All (90)
S1 (5)
S2 (6)
S3 (5)
S4 (4)
S5 (5)
S6 (4)
S7 (5)
S8 (3)
S9 (4)
Subject

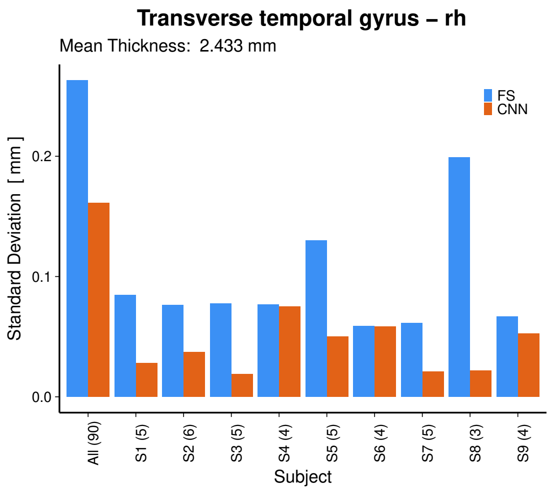
Transverse temporal gyrus – rh
Mean Thickness: 2.433 mm
FS
CNN
Standard Deviation [mm]
0.0
0.1
0.2
All (90)
S1 (5)
S2 (6)
S3 (5)
S4 (4)
S5 (5)
S6 (4)
S7 (5)
S8 (3)
S9 (4)
Subject

4.3 Reliability Plots for Cortical Curvature

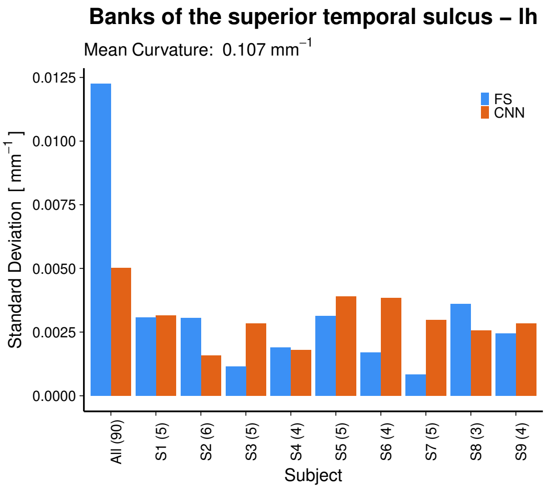

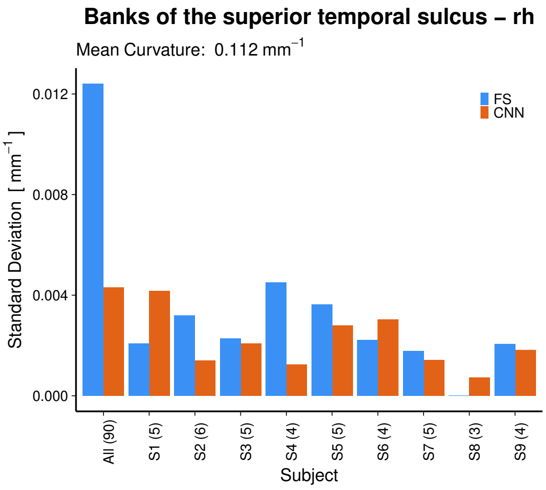

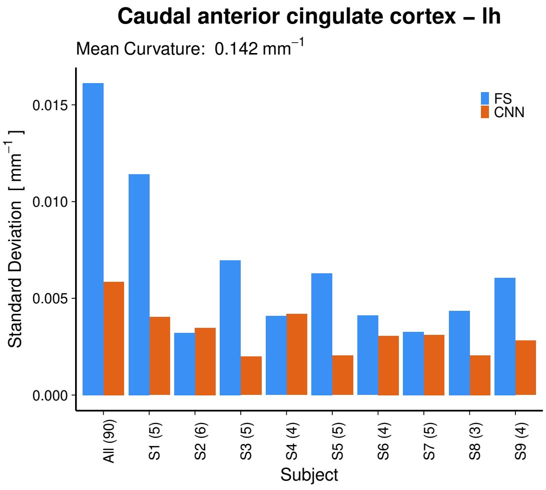

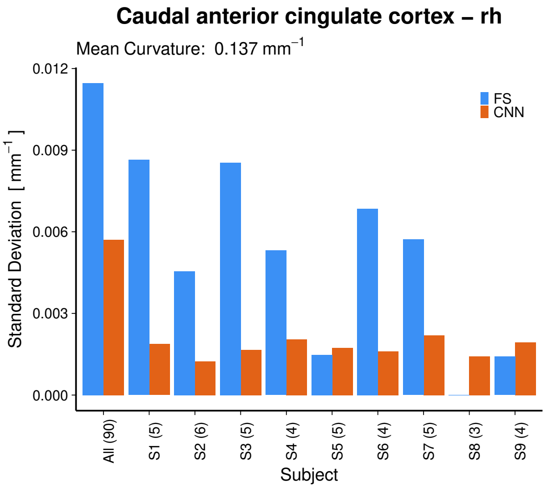

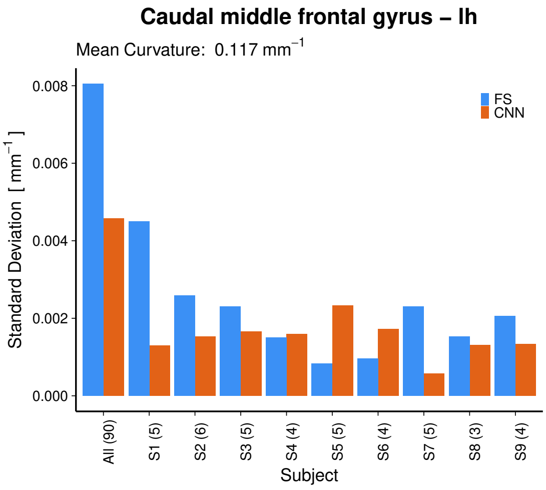
Caudal middle frontal gyrus – lh
Mean Curvature: 0.117 mm^{-1}
Standard Deviation [mm^{-1}]
0.000
0.002
0.004
0.006
0.008
FS
CNN
All (90)
S1 (5)
S2 (6)
S3 (5)
S4 (4)
S5 (5)
S6 (4)
S7 (5)
S8 (3)
S9 (4)
Subject

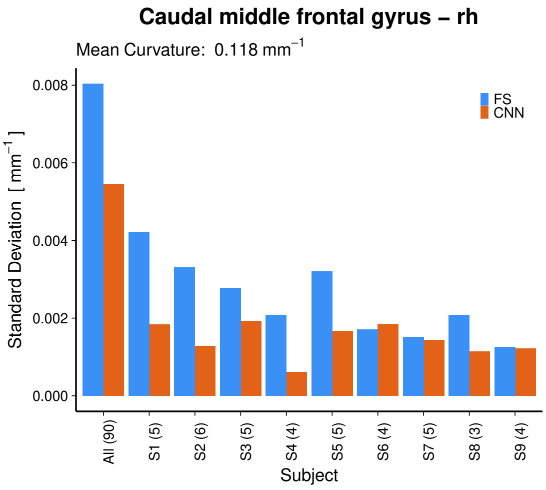
Caudal middle frontal gyrus – rh
Mean Curvature: 0.118 mm^{-1}
Standard Deviation [mm^{-1}]
0.000
0.002
0.004
0.006
0.008
FS
CNN
All (90)
S1 (5)
S2 (6)
S3 (5)
S4 (4)
S5 (5)
S6 (4)
S7 (5)
S8 (3)
S9 (4)
Subject

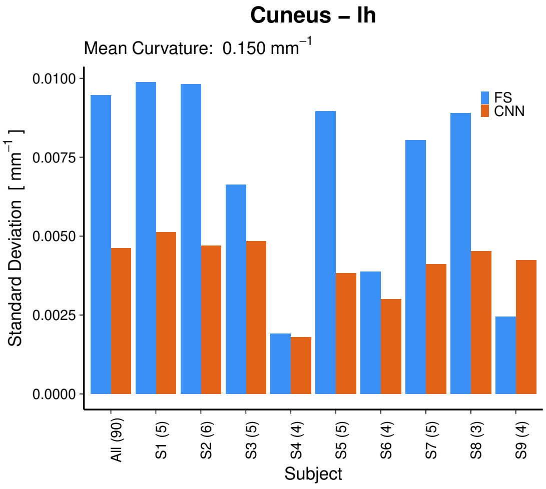
Cuneus – lh
Mean Curvature: 0.150 mm^{-1}
Standard Deviation [mm^{-1}]
0.0000
0.0025
0.0050
0.0075
0.0100
FS
CNN
All (90)
S1 (5)
S2 (6)
S3 (5)
S4 (4)
S5 (5)
S6 (4)
S7 (5)
S8 (3)
S9 (4)
Subject

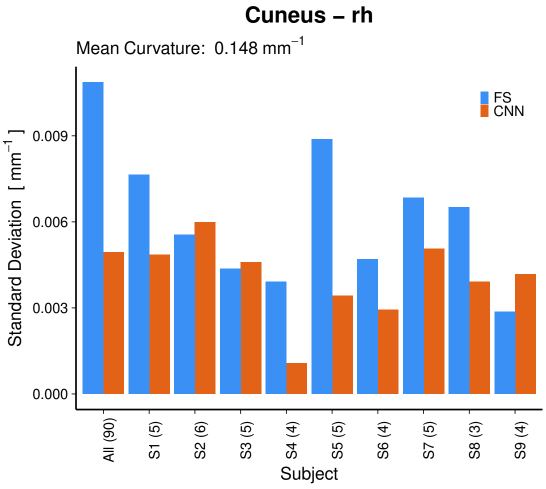
Cuneus – rh
Mean Curvature: 0.148 mm^{-1}
Standard Deviation [mm^{-1}]
0.000
0.003
0.006
0.009
FS
CNN
All (90)
S1 (5)
S2 (6)
S3 (5)
S4 (4)
S5 (5)
S6 (4)
S7 (5)
S8 (3)
S9 (4)
Subject

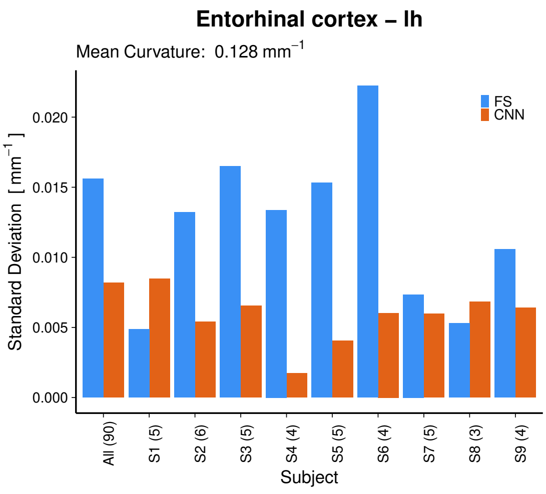
Entorhinal cortex – lh
Mean Curvature: 0.128 mm^{-1}
Standard Deviation [mm^{-1}]
0.000
0.005
0.010
0.015
0.020
FS
CNN
All (90)
S1 (5)
S2 (6)
S3 (5)
S4 (4)
S5 (5)
S6 (4)
S7 (5)
S8 (3)
S9 (4)
Subject

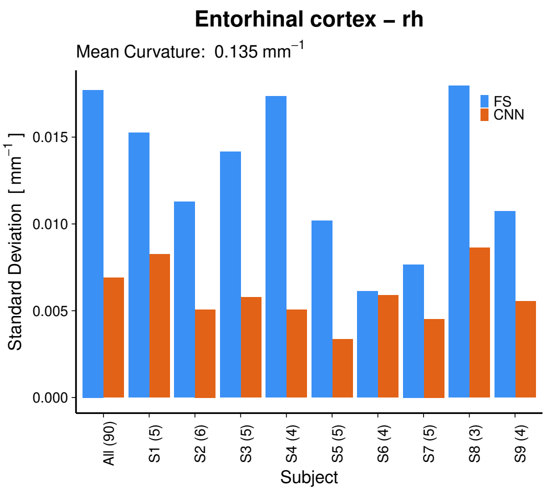
Entorhinal cortex – rh
Mean Curvature: 0.135 mm^{-1}
Standard Deviation [mm^{-1}]
0.000
0.005
0.010
0.015
FS
CNN
All (90)
S1 (5)
S2 (6)
S3 (5)
S4 (4)
S5 (5)
S6 (4)
S7 (5)
S8 (3)
S9 (4)
Subject

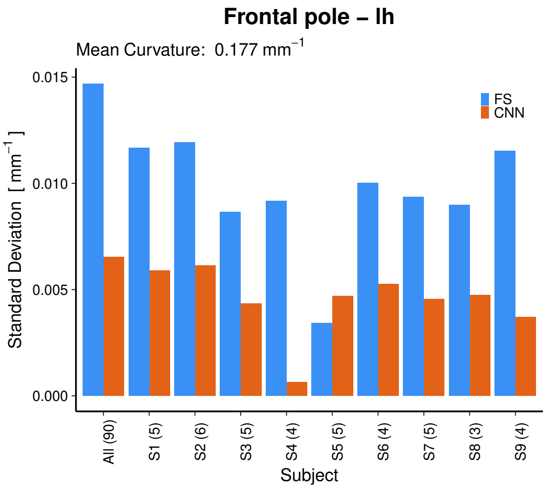
Frontal pole – lh
Mean Curvature: 0.177 mm−1
Standard Deviation [mm−1]
0.015
0.010
0.005
0.000
FS
CNN
All (90)
S1 (5)
S2 (6)
S3 (5)
S4 (4)
S5 (5)
S6 (4)
S7 (5)
S8 (3)
S9 (4)
Subject

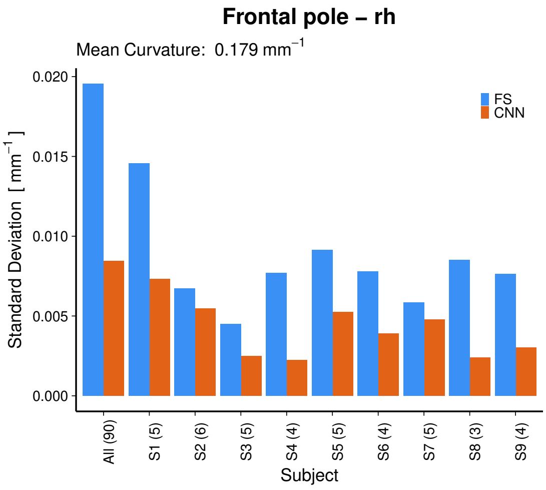
Frontal pole – rh
Mean Curvature: 0.179 mm−1
Standard Deviation [mm−1]
0.020
0.015
0.010
0.005
0.000
FS
CNN
All (90)
S1 (5)
S2 (6)
S3 (5)
S4 (4)
S5 (5)
S6 (4)
S7 (5)
S8 (3)
S9 (4)
Subject

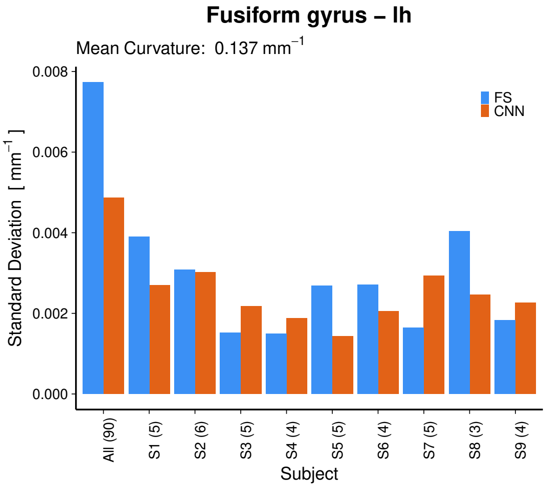
Fusiform gyrus – lh
Mean Curvature: 0.137 mm−1
Standard Deviation [mm−1]
0.008
0.006
0.004
0.002
0.000
FS
CNN
All (90)
S1 (5)
S2 (6)
S3 (5)
S4 (4)
S5 (5)
S6 (4)
S7 (5)
S8 (3)
S9 (4)
Subject

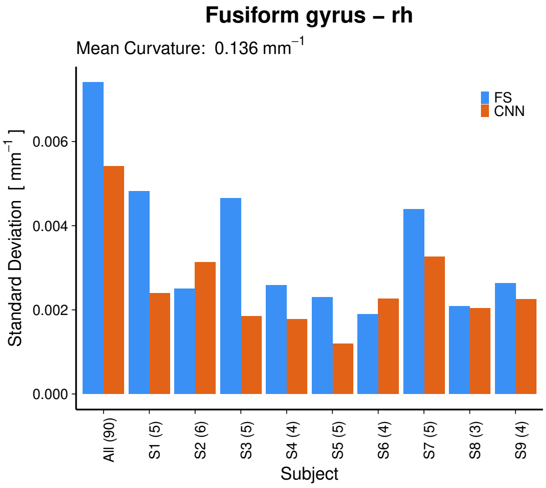
Fusiform gyrus – rh
Mean Curvature: 0.136 mm−1
Standard Deviation [mm−1]
0.006
0.004
0.002
0.000
FS
CNN
All (90)
S1 (5)
S2 (6)
S3 (5)
S4 (4)
S5 (5)
S6 (4)
S7 (5)
S8 (3)
S9 (4)
Subject

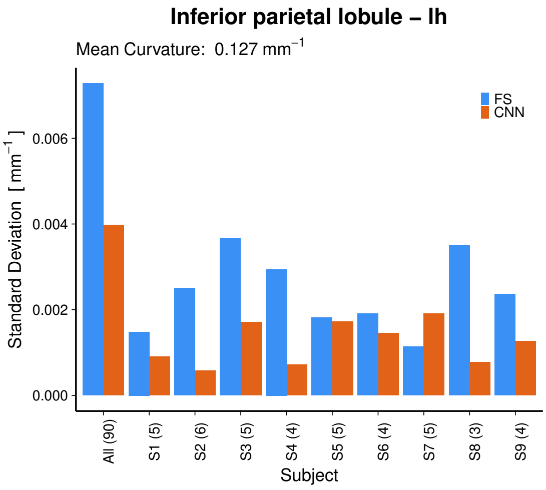
Inferior parietal lobule – lh
Mean Curvature: 0.127 mm−1
Standard Deviation [mm−1]
0.006
0.004
0.002
0.000
FS
CNN
All (90)
S1 (5)
S2 (6)
S3 (5)
S4 (4)
S5 (5)
S6 (4)
S7 (5)
S8 (3)
S9 (4)
Subject

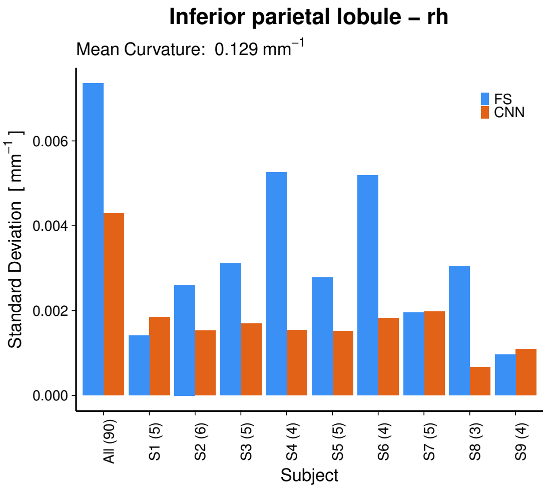
Inferior parietal lobule – rh
Mean Curvature: 0.129 mm−1
Standard Deviation [mm−1]
0.006
0.004
0.002
0.000
FS
CNN
All (90)
S1 (5)
S2 (6)
S3 (5)
S4 (4)
S5 (5)
S6 (4)
S7 (5)
S8 (3)
S9 (4)
Subject

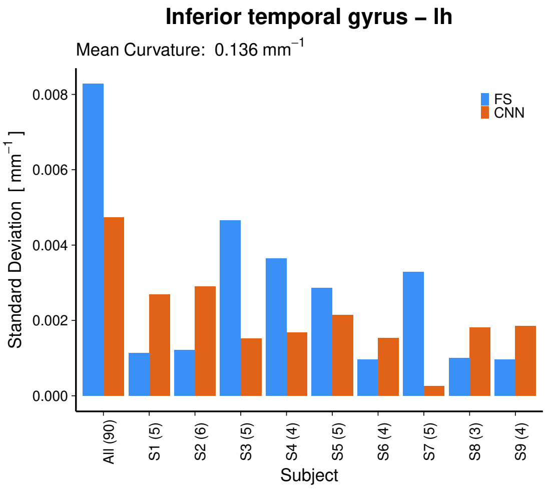
Inferior temporal gyrus – lh
Mean Curvature: 0.136 mm^{-1}
Standard Deviation [mm^{-1}]
0.008
0.006
0.004
0.002
0.000
FS
CNN
All (90)
S1 (5)
S2 (6)
S3 (5)
S4 (4)
S5 (5)
S6 (4)
S7 (5)
S8 (3)
S9 (4)
Subject

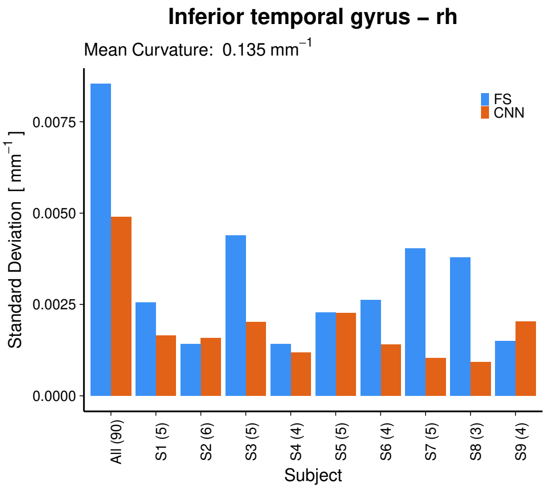
Inferior temporal gyrus – rh
Mean Curvature: 0.135 mm^{-1}
Standard Deviation [mm^{-1}]
0.0075
0.0050
0.0025
0.0000
FS
CNN
All (90)
S1 (5)
S2 (6)
S3 (5)
S4 (4)
S5 (5)
S6 (4)
S7 (5)
S8 (3)
S9 (4)
Subject

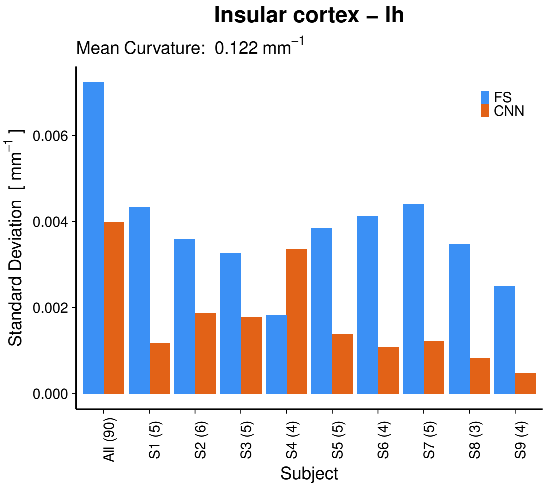
Insular cortex – lh
Mean Curvature: 0.122 mm^{-1}
Standard Deviation [mm^{-1}]
0.006
0.004
0.002
0.000
FS
CNN
All (90)
S1 (5)
S2 (6)
S3 (5)
S4 (4)
S5 (5)
S6 (4)
S7 (5)
S8 (3)
S9 (4)
Subject

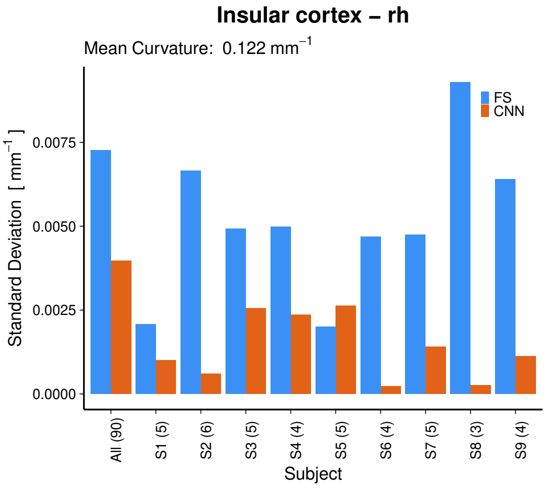
Insular cortex – rh
Mean Curvature: 0.122 mm^{-1}
Standard Deviation [mm^{-1}]
0.0075
0.0050
0.0025
0.0000
FS
CNN
All (90)
S1 (5)
S2 (6)
S3 (5)
S4 (4)
S5 (5)
S6 (4)
S7 (5)
S8 (3)
S9 (4)
Subject

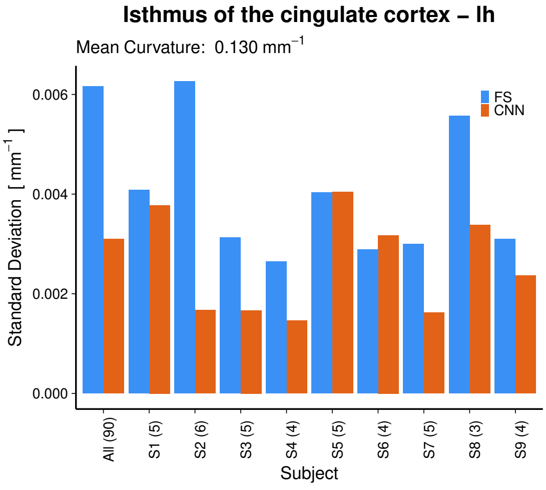
Isthmus of the cingulate cortex – lh
Mean Curvature: 0.130 mm^{-1}
Standard Deviation [mm^{-1}]
0.006
0.004
0.002
0.000
FS
CNN
All (90)
S1 (5)
S2 (6)
S3 (5)
S4 (4)
S5 (5)
S6 (4)
S7 (5)
S8 (3)
S9 (4)
Subject

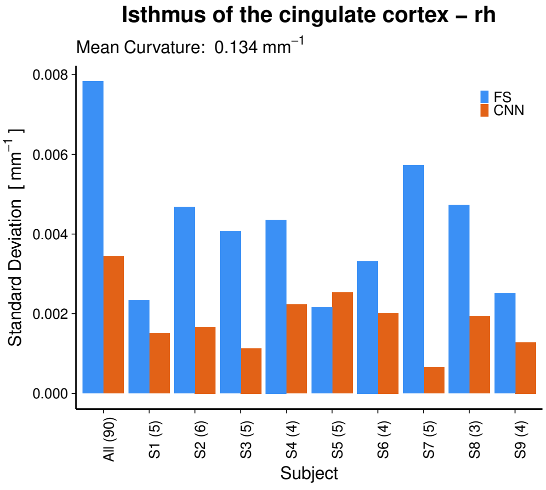
Isthmus of the cingulate cortex – rh
Mean Curvature: 0.134 mm^{-1}
Standard Deviation [mm^{-1}]
0.008
0.006
0.004
0.002
0.000
FS
CNN
All (90)
S1 (5)
S2 (6)
S3 (5)
S4 (4)
S5 (5)
S6 (4)
S7 (5)
S8 (3)
S9 (4)
Subject

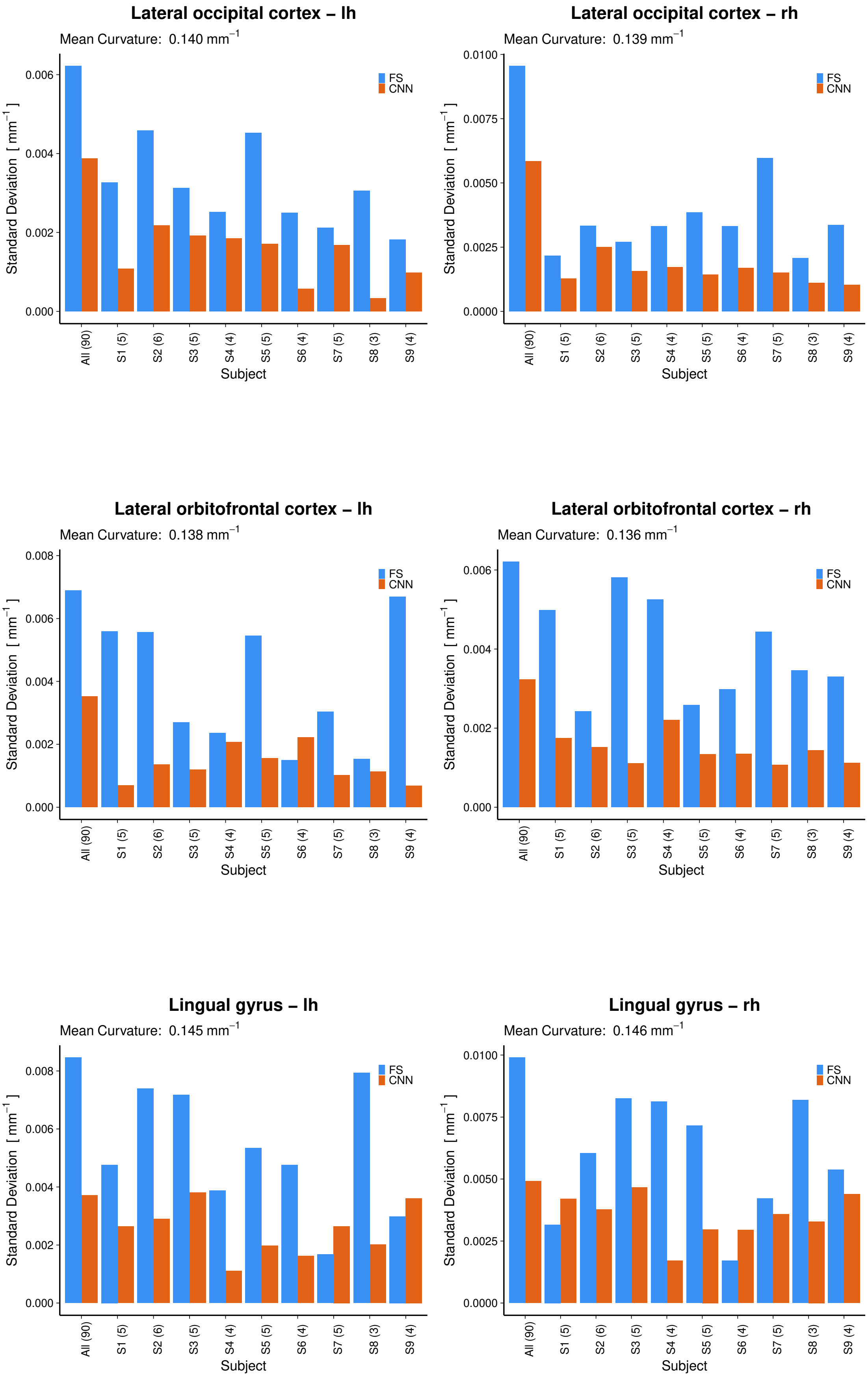

Lateral occipital cortex – lh
Mean Curvature: 0.140 mm⁻¹
Lateral occipital cortex – rh
Mean Curvature: 0.139 mm⁻¹
Lateral orbitofrontal cortex – lh
Mean Curvature: 0.138 mm⁻¹
Lateral orbitofrontal cortex – rh
Mean Curvature: 0.136 mm⁻¹
Lingual gyrus – lh
Mean Curvature: 0.145 mm⁻¹
Lingual gyrus – rh
Mean Curvature: 0.146 mm⁻¹
Standard Deviation [mm⁻¹]
Subject
All (90)
S1 (5)
S2 (6)
S3 (5)
S4 (4)
S5 (5)
S6 (4)
S7 (5)
S8 (3)
S9 (4)
FS
CNN

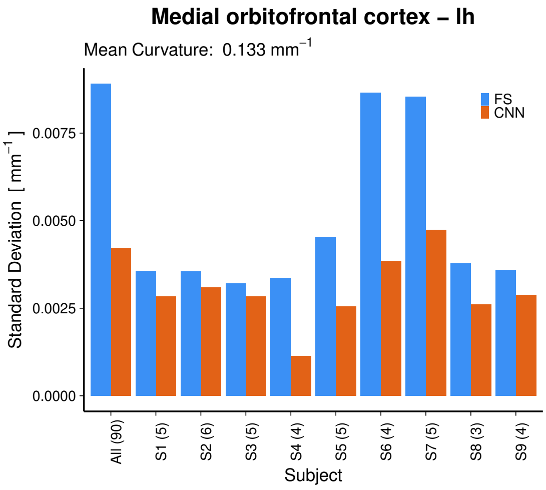
Medial orbitofrontal cortex – lh
Mean Curvature: 0.133 mm⁻¹
Standard Deviation [mm⁻¹]
0.0000
0.0025
0.0050
0.0075
FS
CNN
All (90)
S1 (5)
S2 (6)
S3 (5)
S4 (4)
S5 (5)
S6 (4)
S7 (5)
S8 (3)
S9 (4)
Subject

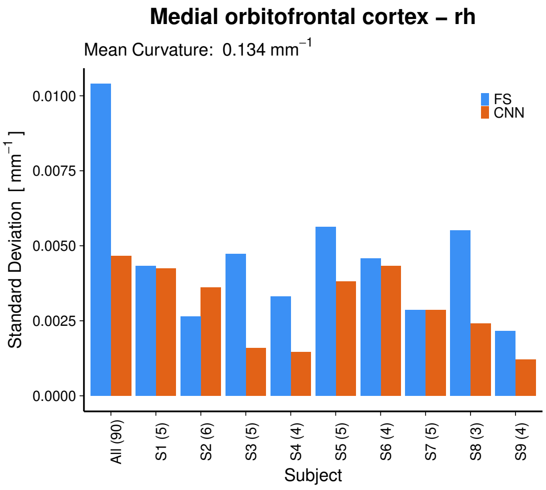
Medial orbitofrontal cortex – rh
Mean Curvature: 0.134 mm⁻¹
Standard Deviation [mm⁻¹]
0.0000
0.0025
0.0050
0.0075
0.0100
FS
CNN
All (90)
S1 (5)
S2 (6)
S3 (5)
S4 (4)
S5 (5)
S6 (4)
S7 (5)
S8 (3)
S9 (4)
Subject

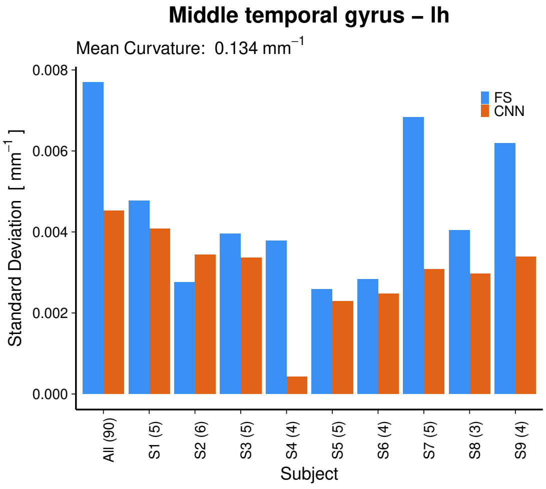
Middle temporal gyrus – lh
Mean Curvature: 0.134 mm⁻¹
Standard Deviation [mm⁻¹]
0.000
0.002
0.004
0.006
0.008
FS
CNN
All (90)
S1 (5)
S2 (6)
S3 (5)
S4 (4)
S5 (5)
S6 (4)
S7 (5)
S8 (3)
S9 (4)
Subject

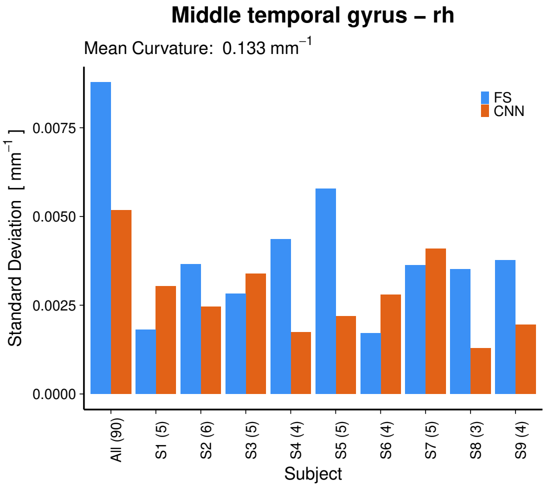
Middle temporal gyrus – rh
Mean Curvature: 0.133 mm⁻¹
Standard Deviation [mm⁻¹]
0.0000
0.0025
0.0050
0.0075
FS
CNN
All (90)
S1 (5)
S2 (6)
S3 (5)
S4 (4)
S5 (5)
S6 (4)
S7 (5)
S8 (3)
S9 (4)
Subject

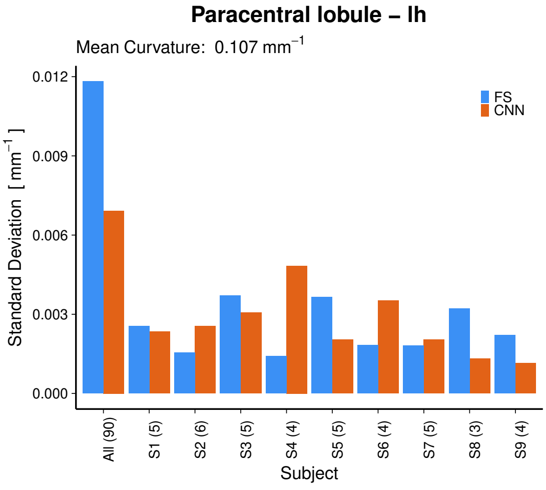
Paracentral lobule – lh
Mean Curvature: 0.107 mm⁻¹
Standard Deviation [mm⁻¹]
0.000
0.003
0.006
0.009
0.012
FS
CNN
All (90)
S1 (5)
S2 (6)
S3 (5)
S4 (4)
S5 (5)
S6 (4)
S7 (5)
S8 (3)
S9 (4)
Subject

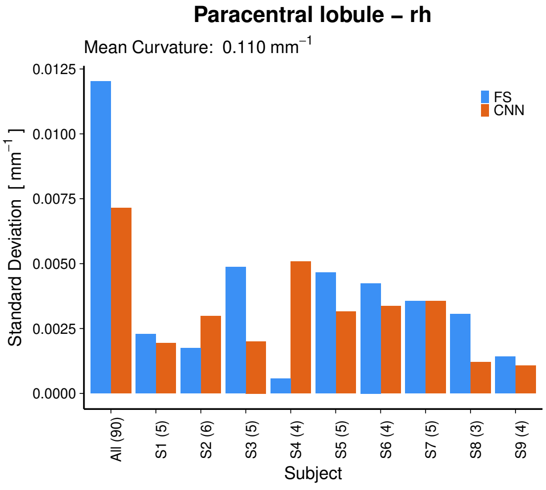
Paracentral lobule – rh
Mean Curvature: 0.110 mm⁻¹
Standard Deviation [mm⁻¹]
0.0000
0.0025
0.0050
0.0075
0.0100
0.0125
FS
CNN
All (90)
S1 (5)
S2 (6)
S3 (5)
S4 (4)
S5 (5)
S6 (4)
S7 (5)
S8 (3)
S9 (4)
Subject

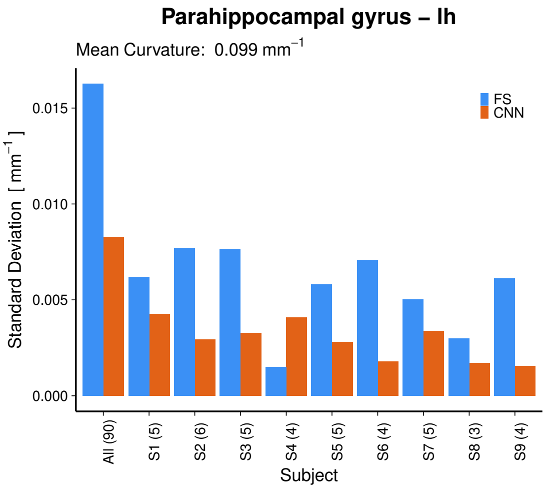
Parahippocampal gyrus – lh
Mean Curvature: 0.099 mm⁻¹
Standard Deviation [mm⁻¹]
0.000
0.005
0.010
0.015
FS
CNN
All (90)
S1 (5)
S2 (6)
S3 (5)
S4 (4)
S5 (5)
S6 (4)
S7 (5)
S8 (3)
S9 (4)
Subject

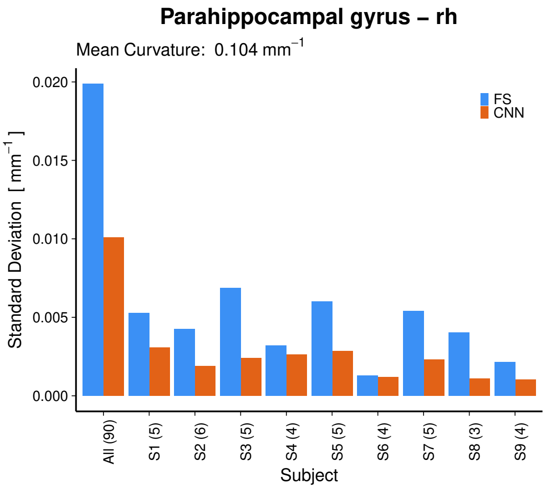
Parahippocampal gyrus – rh
Mean Curvature: 0.104 mm⁻¹
Standard Deviation [mm⁻¹]
0.000
0.005
0.010
0.015
0.020
FS
CNN
All (90)
S1 (5)
S2 (6)
S3 (5)
S4 (4)
S5 (5)
S6 (4)
S7 (5)
S8 (3)
S9 (4)
Subject

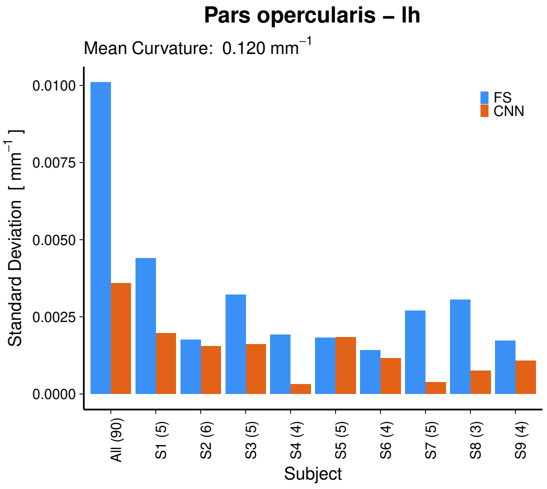
Pars opercularis – lh
Mean Curvature: 0.120 mm⁻¹
Standard Deviation [mm⁻¹]
0.0000
0.0025
0.0050
0.0075
0.0100
FS
CNN
All (90)
S1 (5)
S2 (6)
S3 (5)
S4 (4)
S5 (5)
S6 (4)
S7 (5)
S8 (3)
S9 (4)
Subject

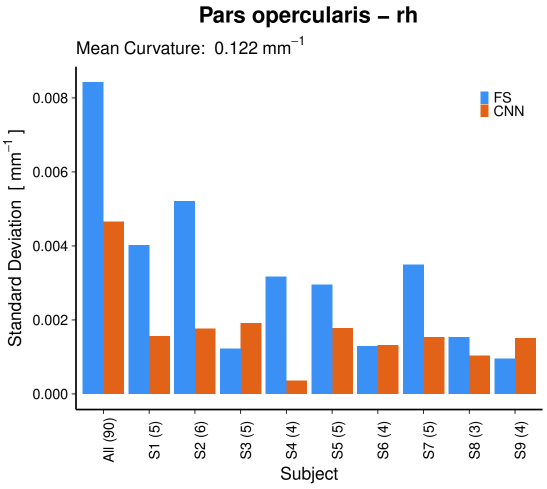
Pars opercularis – rh
Mean Curvature: 0.122 mm⁻¹
Standard Deviation [mm⁻¹]
0.000
0.002
0.004
0.006
0.008
FS
CNN
All (90)
S1 (5)
S2 (6)
S3 (5)
S4 (4)
S5 (5)
S6 (4)
S7 (5)
S8 (3)
S9 (4)
Subject

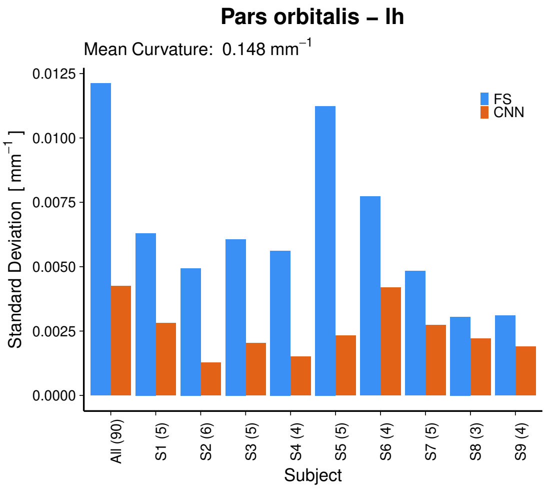
Pars orbitalis – lh
Mean Curvature: 0.148 mm⁻¹
Standard Deviation [mm⁻¹]
0.0000
0.0025
0.0050
0.0075
0.0100
0.0125
FS
CNN
All (90)
S1 (5)
S2 (6)
S3 (5)
S4 (4)
S5 (5)
S6 (4)
S7 (5)
S8 (3)
S9 (4)
Subject

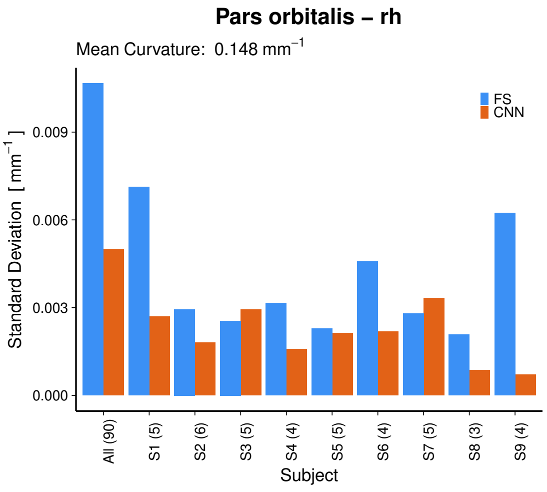
Pars orbitalis – rh
Mean Curvature: 0.148 mm⁻¹
Standard Deviation [mm⁻¹]
0.000
0.003
0.006
0.009
FS
CNN
All (90)
S1 (5)
S2 (6)
S3 (5)
S4 (4)
S5 (5)
S6 (4)
S7 (5)
S8 (3)
S9 (4)
Subject

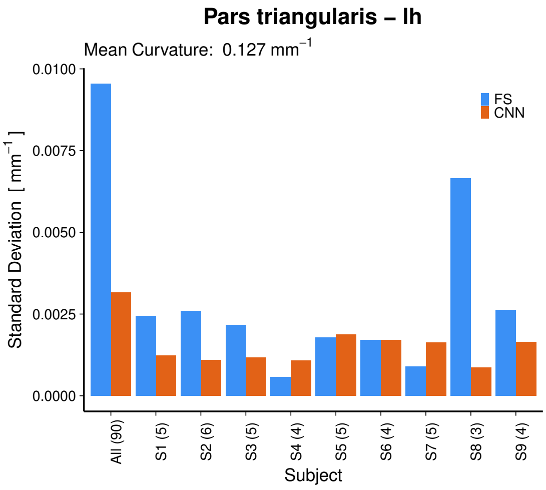
Pars triangularis – lh
Mean Curvature: 0.127 mm^{-1}
Standard Deviation [mm^{-1}]
0.0100
0.0075
0.0050
0.0025
0.0000
FS
CNN
All (90)
S1 (5)
S2 (6)
S3 (5)
S4 (4)
S5 (5)
S6 (4)
S7 (5)
S8 (3)
S9 (4)
Subject

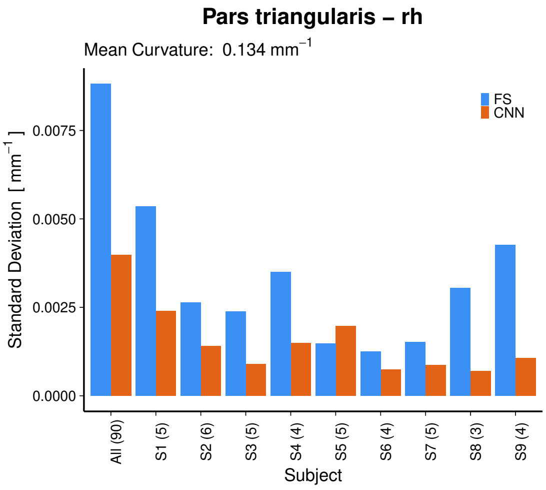
Pars triangularis – rh
Mean Curvature: 0.134 mm^{-1}
Standard Deviation [mm^{-1}]
0.0075
0.0050
0.0025
0.0000
FS
CNN
All (90)
S1 (5)
S2 (6)
S3 (5)
S4 (4)
S5 (5)
S6 (4)
S7 (5)
S8 (3)
S9 (4)
Subject

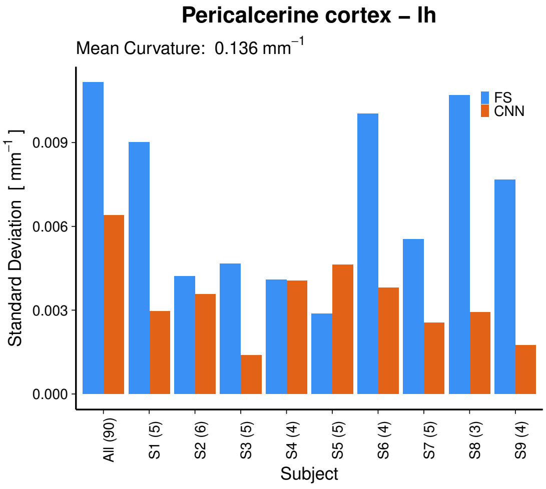
Pericalcerine cortex – lh
Mean Curvature: 0.136 mm^{-1}
Standard Deviation [mm^{-1}]
0.009
0.006
0.003
0.000
FS
CNN
All (90)
S1 (5)
S2 (6)
S3 (5)
S4 (4)
S5 (5)
S6 (4)
S7 (5)
S8 (3)
S9 (4)
Subject

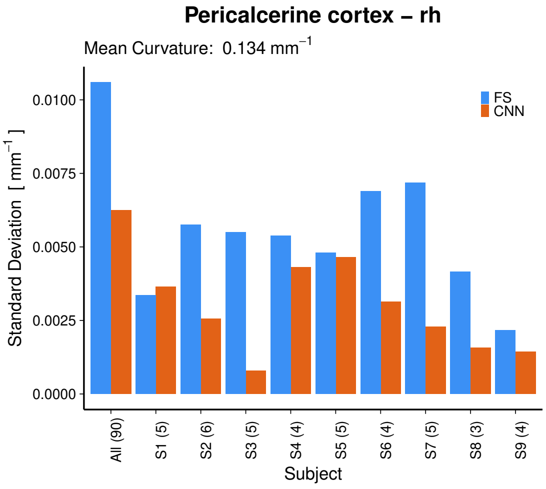
Pericalcerine cortex – rh
Mean Curvature: 0.134 mm^{-1}
Standard Deviation [mm^{-1}]
0.0100
0.0075
0.0050
0.0025
0.0000
FS
CNN
All (90)
S1 (5)
S2 (6)
S3 (5)
S4 (4)
S5 (5)
S6 (4)
S7 (5)
S8 (3)
S9 (4)
Subject

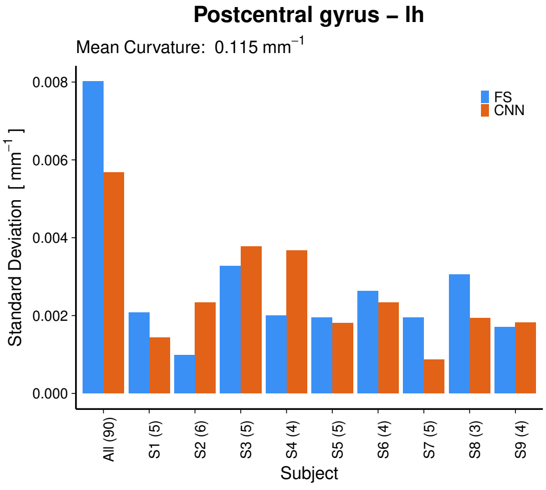
Postcentral gyrus – lh
Mean Curvature: 0.115 mm^{-1}
Standard Deviation [mm^{-1}]
0.008
0.006
0.004
0.002
0.000
FS
CNN
All (90)
S1 (5)
S2 (6)
S3 (5)
S4 (4)
S5 (5)
S6 (4)
S7 (5)
S8 (3)
S9 (4)
Subject

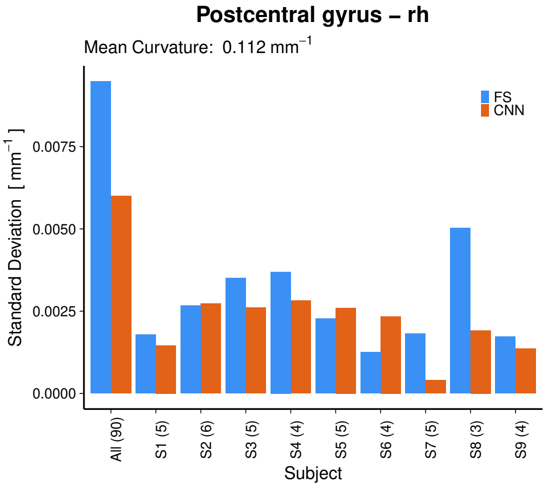
Postcentral gyrus – rh
Mean Curvature: 0.112 mm^{-1}
Standard Deviation [mm^{-1}]
0.0075
0.0050
0.0025
0.0000
FS
CNN
All (90)
S1 (5)
S2 (6)
S3 (5)
S4 (4)
S5 (5)
S6 (4)
S7 (5)
S8 (3)
S9 (4)
Subject

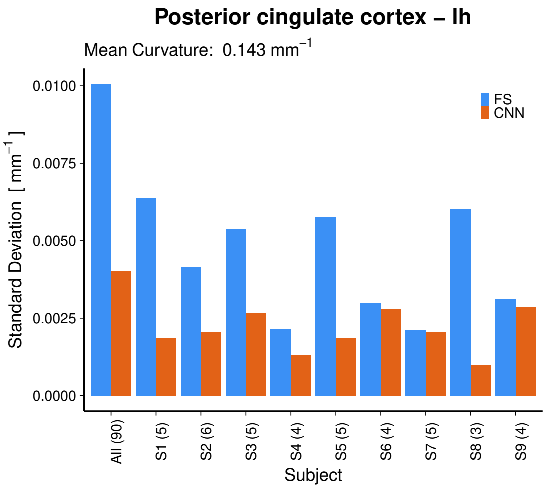
Posterior cingulate cortex – lh
Mean Curvature: 0.143 mm⁻¹
Standard Deviation [mm⁻¹]
0.0100
0.0075
0.0050
0.0025
0.0000
FS
CNN
All (90)
S1 (5)
S2 (6)
S3 (5)
S4 (4)
S5 (5)
S6 (4)
S7 (5)
S8 (3)
S9 (4)
Subject

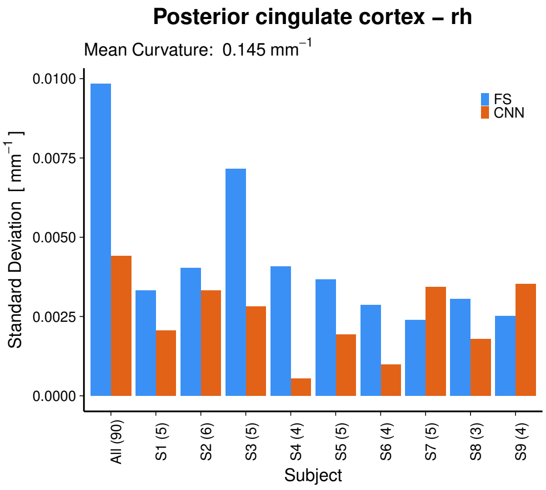
Posterior cingulate cortex – rh
Mean Curvature: 0.145 mm⁻¹
Standard Deviation [mm⁻¹]
0.0100
0.0075
0.0050
0.0025
0.0000
FS
CNN
All (90)
S1 (5)
S2 (6)
S3 (5)
S4 (4)
S5 (5)
S6 (4)
S7 (5)
S8 (3)
S9 (4)
Subject

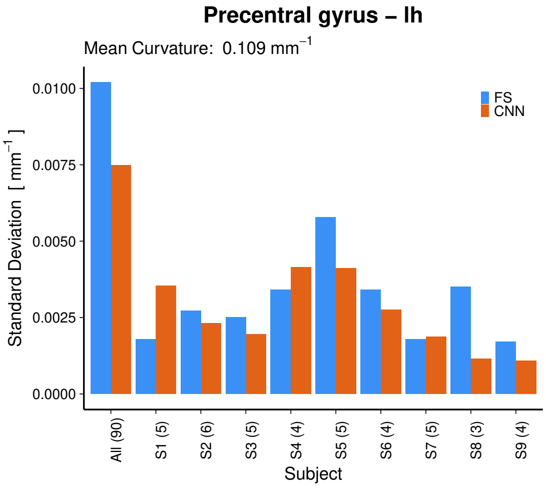
Precentral gyrus – lh
Mean Curvature: 0.109 mm⁻¹
Standard Deviation [mm⁻¹]
0.0100
0.0075
0.0050
0.0025
0.0000
FS
CNN
All (90)
S1 (5)
S2 (6)
S3 (5)
S4 (4)
S5 (5)
S6 (4)
S7 (5)
S8 (3)
S9 (4)
Subject

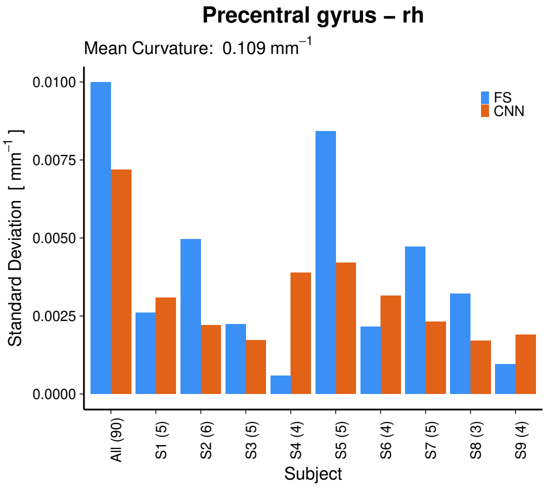
Precentral gyrus – rh
Mean Curvature: 0.109 mm⁻¹
Standard Deviation [mm⁻¹]
0.0100
0.0075
0.0050
0.0025
0.0000
FS
CNN
All (90)
S1 (5)
S2 (6)
S3 (5)
S4 (4)
S5 (5)
S6 (4)
S7 (5)
S8 (3)
S9 (4)
Subject

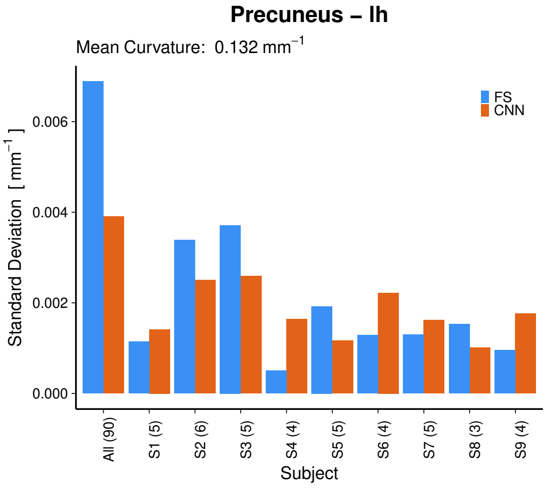
Precuneus – lh
Mean Curvature: 0.132 mm⁻¹
Standard Deviation [mm⁻¹]
0.006
0.004
0.002
0.000
FS
CNN
All (90)
S1 (5)
S2 (6)
S3 (5)
S4 (4)
S5 (5)
S6 (4)
S7 (5)
S8 (3)
S9 (4)
Subject

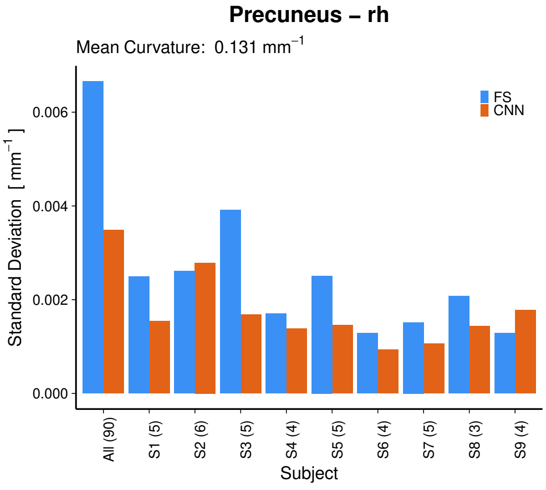
Precuneus – rh
Mean Curvature: 0.131 mm⁻¹
Standard Deviation [mm⁻¹]
0.006
0.004
0.002
0.000
FS
CNN
All (90)
S1 (5)
S2 (6)
S3 (5)
S4 (4)
S5 (5)
S6 (4)
S7 (5)
S8 (3)
S9 (4)
Subject

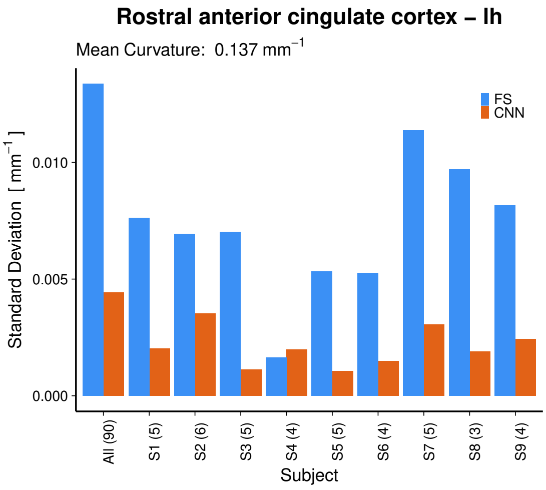
Rostral anterior cingulate cortex – lh
Mean Curvature: 0.137 mm⁻¹
Standard Deviation [mm⁻¹]
0.000
0.005
0.010
FS
CNN
All (90)
S1 (5)
S2 (6)
S3 (5)
S4 (4)
S5 (5)
S6 (4)
S7 (5)
S8 (3)
S9 (4)
Subject

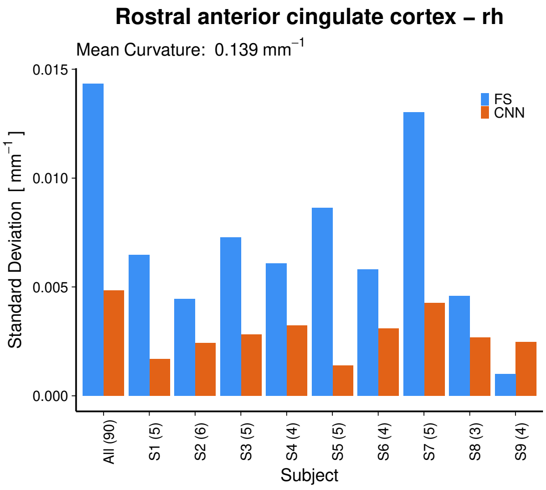
Rostral anterior cingulate cortex – rh
Mean Curvature: 0.139 mm⁻¹
Standard Deviation [mm⁻¹]
0.000
0.005
0.010
0.015
FS
CNN
All (90)
S1 (5)
S2 (6)
S3 (5)
S4 (4)
S5 (5)
S6 (4)
S7 (5)
S8 (3)
S9 (4)
Subject

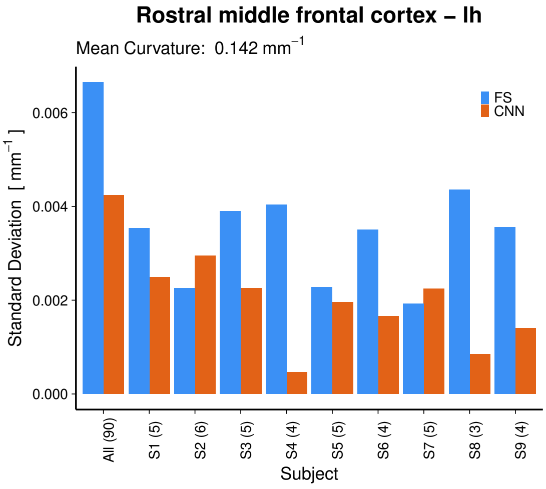
Rostral middle frontal cortex – lh
Mean Curvature: 0.142 mm⁻¹
Standard Deviation [mm⁻¹]
0.000
0.002
0.004
0.006
FS
CNN
All (90)
S1 (5)
S2 (6)
S3 (5)
S4 (4)
S5 (5)
S6 (4)
S7 (5)
S8 (3)
S9 (4)
Subject

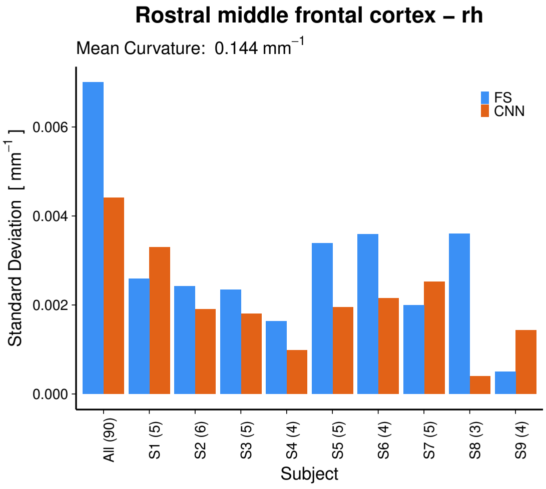
Rostral middle frontal cortex – rh
Mean Curvature: 0.144 mm⁻¹
Standard Deviation [mm⁻¹]
0.000
0.002
0.004
0.006
FS
CNN
All (90)
S1 (5)
S2 (6)
S3 (5)
S4 (4)
S5 (5)
S6 (4)
S7 (5)
S8 (3)
S9 (4)
Subject

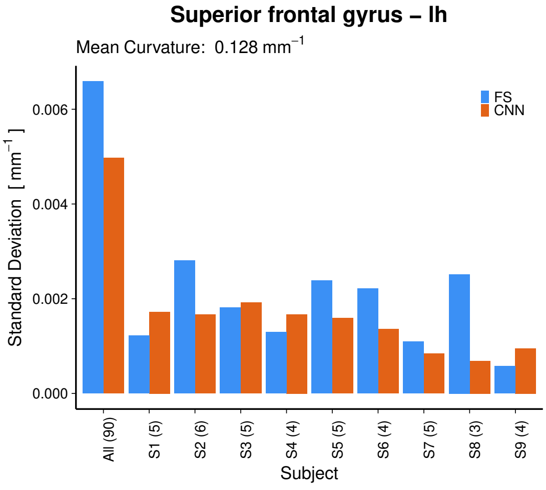
Superior frontal gyrus – lh
Mean Curvature: 0.128 mm⁻¹
Standard Deviation [mm⁻¹]
0.000
0.002
0.004
0.006
FS
CNN
All (90)
S1 (5)
S2 (6)
S3 (5)
S4 (4)
S5 (5)
S6 (4)
S7 (5)
S8 (3)
S9 (4)
Subject

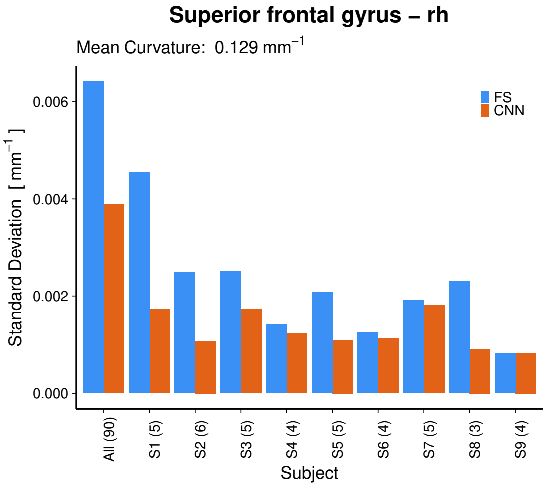
Superior frontal gyrus – rh
Mean Curvature: 0.129 mm⁻¹
Standard Deviation [mm⁻¹]
0.000
0.002
0.004
0.006
FS
CNN
All (90)
S1 (5)
S2 (6)
S3 (5)
S4 (4)
S5 (5)
S6 (4)
S7 (5)
S8 (3)
S9 (4)
Subject

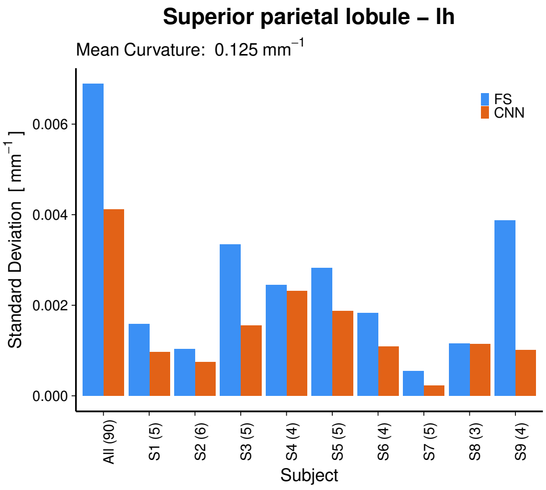
Superior parietal lobule – lh
Mean Curvature: 0.125 mm⁻¹
Standard Deviation [mm⁻¹]
0.000
0.002
0.004
0.006
FS
CNN
All (90)
S1 (5)
S2 (6)
S3 (5)
S4 (4)
S5 (5)
S6 (4)
S7 (5)
S8 (3)
S9 (4)
Subject

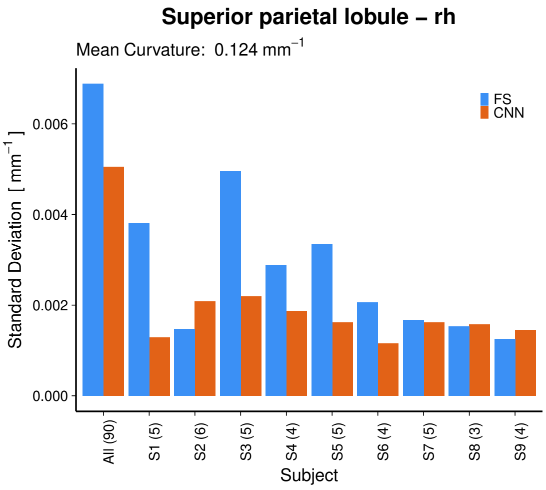
Superior parietal lobule – rh
Mean Curvature: 0.124 mm⁻¹
Standard Deviation [mm⁻¹]
0.000
0.002
0.004
0.006
FS
CNN
All (90)
S1 (5)
S2 (6)
S3 (5)
S4 (4)
S5 (5)
S6 (4)
S7 (5)
S8 (3)
S9 (4)
Subject

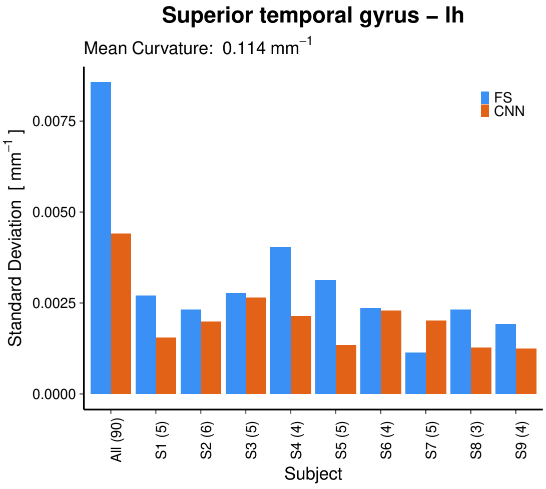
Superior temporal gyrus – lh
Mean Curvature: 0.114 mm⁻¹
Standard Deviation [mm⁻¹]
0.0000
0.0025
0.0050
0.0075
FS
CNN
All (90)
S1 (5)
S2 (6)
S3 (5)
S4 (4)
S5 (5)
S6 (4)
S7 (5)
S8 (3)
S9 (4)
Subject

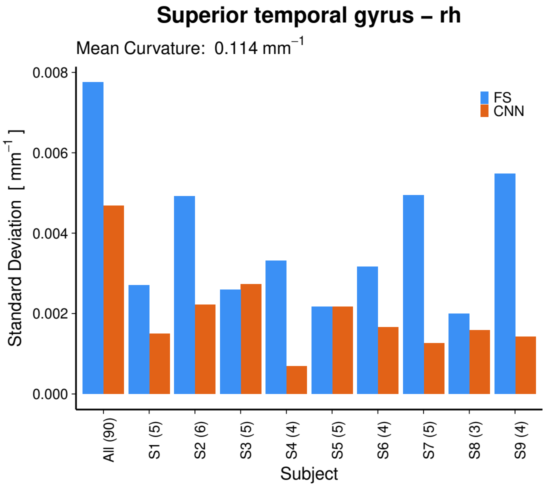
Superior temporal gyrus – rh
Mean Curvature: 0.114 mm⁻¹
Standard Deviation [mm⁻¹]
0.000
0.002
0.004
0.006
0.008
FS
CNN
All (90)
S1 (5)
S2 (6)
S3 (5)
S4 (4)
S5 (5)
S6 (4)
S7 (5)
S8 (3)
S9 (4)
Subject

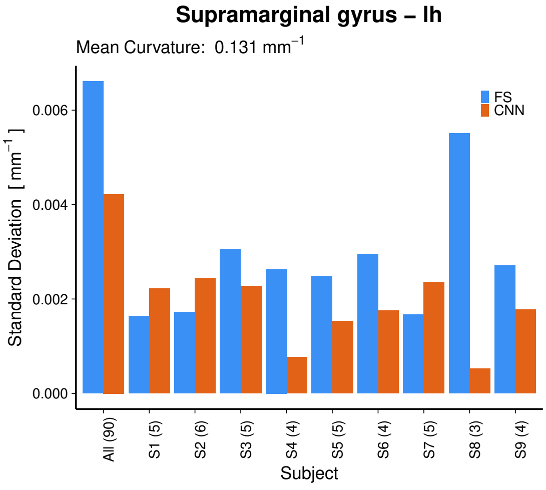
Supramarginal gyrus – lh
Mean Curvature: 0.131 mm⁻¹
Standard Deviation [mm⁻¹]
0.000
0.002
0.004
0.006
FS
CNN
All (90)
S1 (5)
S2 (6)
S3 (5)
S4 (4)
S5 (5)
S6 (4)
S7 (5)
S8 (3)
S9 (4)
Subject

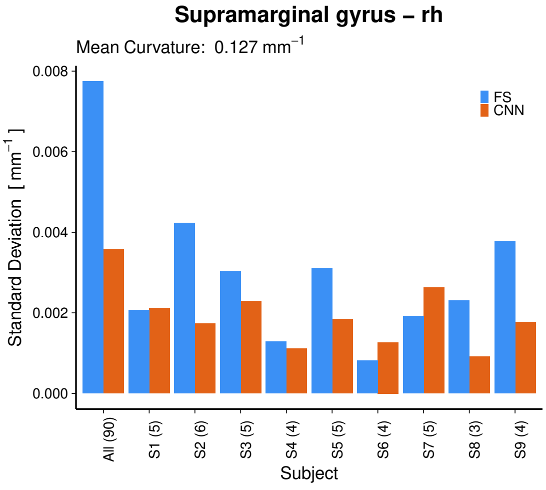
Supramarginal gyrus – rh
Mean Curvature: 0.127 mm⁻¹
Standard Deviation [mm⁻¹]
0.000
0.002
0.004
0.006
0.008
FS
CNN
All (90)
S1 (5)
S2 (6)
S3 (5)
S4 (4)
S5 (5)
S6 (4)
S7 (5)
S8 (3)
S9 (4)
Subject

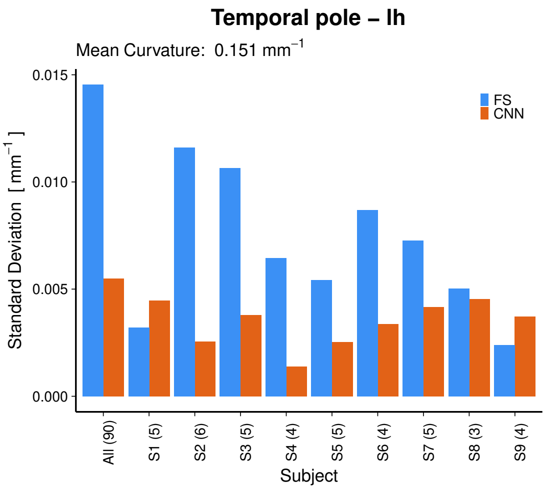
Temporal pole – lh
Mean Curvature: 0.151 mm−1
Standard Deviation [mm−1]
0.015
0.010
0.005
0.000
FS
CNN
All (90)
S1 (5)
S2 (6)
S3 (5)
S4 (4)
S5 (5)
S6 (4)
S7 (5)
S8 (3)
S9 (4)
Subject

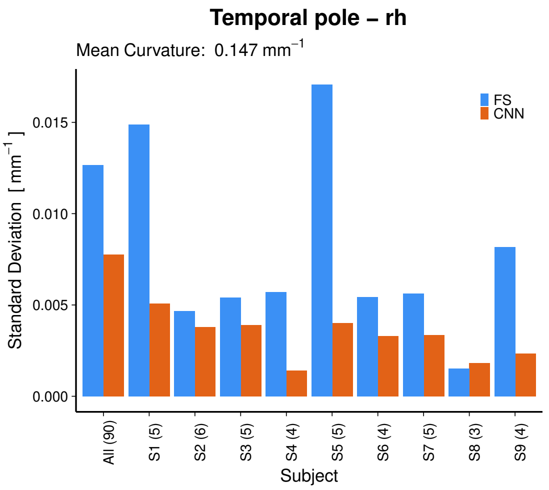
Temporal pole – rh
Mean Curvature: 0.147 mm−1
Standard Deviation [mm−1]
0.015
0.010
0.005
0.000
FS
CNN
All (90)
S1 (5)
S2 (6)
S3 (5)
S4 (4)
S5 (5)
S6 (4)
S7 (5)
S8 (3)
S9 (4)
Subject

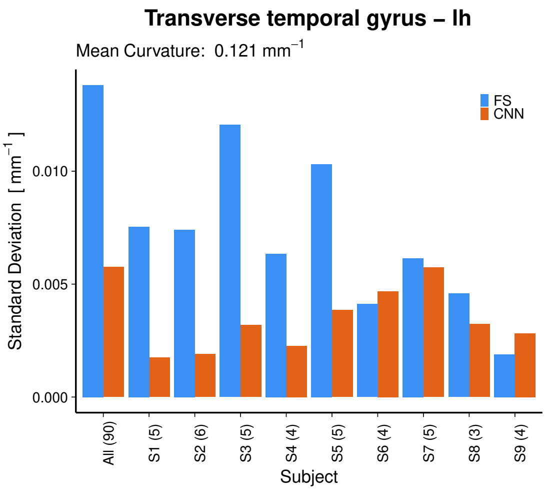
Transverse temporal gyrus – lh
Mean Curvature: 0.121 mm−1
Standard Deviation [mm−1]
0.010
0.005
0.000
FS
CNN
All (90)
S1 (5)
S2 (6)
S3 (5)
S4 (4)
S5 (5)
S6 (4)
S7 (5)
S8 (3)
S9 (4)
Subject

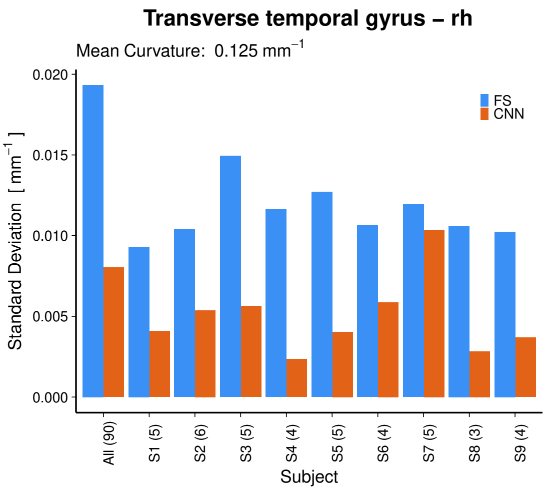
Transverse temporal gyrus – rh
Mean Curvature: 0.125 mm−1
Standard Deviation [mm−1]
0.020
0.015
0.010
0.005
0.000
FS
CNN
All (90)
S1 (5)
S2 (6)
S3 (5)
S4 (4)
S5 (5)
S6 (4)
S7 (5)
S8 (3)
S9 (4)
Subject
